# Design, Synthesis, and Evaluation of *B*-(Trifluoromethyl)phenyl Phosphine–Borane Derivatives as Novel Progesterone Receptor Antagonists

**DOI:** 10.3390/molecules29071587

**Published:** 2024-04-02

**Authors:** Yu Miyajima, Kotaro Ochiai, Shinya Fujii

**Affiliations:** Institute of Biomaterials and Bioengineering, Tokyo Medical and Dental University, 2-3-10 Kanda-Surugadai, Chiyoda-ku, Tokyo 101-0062, Japan

**Keywords:** phosphorus, phosphine–borane, hydrophobicity, progesterone receptor, antagonist, structural optimization

## Abstract

We previously revealed that phosphine–boranes can function as molecular frameworks for biofunctional molecules. In the present study, we exploited the diversity of available phosphines to design and synthesize a series of *B*-(trifluoromethyl)phenyl phosphine–borane derivatives as novel progesterone receptor (PR) antagonists. We revealed that the synthesized phosphine–borane derivatives exhibited Log*P* values in a predictable manner and that the P–H group in the phosphine–borane was almost nonpolar. Among the synthesized phosphine–boranes, which exhibited PR antagonistic activity, *B*-(4-trifluoromethyl)phenyl tricyclopropylphosphine–borane was the most potent with an IC_50_ value of 0.54 μM. A docking simulation indicated that the tricyclopropylphosphine moiety plays an important role in ligand–receptor interactions. These results support the idea that phosphine–boranes are versatile structural options in drug discovery, and the developed compounds are promising lead compounds for further structural development of next-generation PR antagonists.

## 1. Introduction

**Figure 1:**
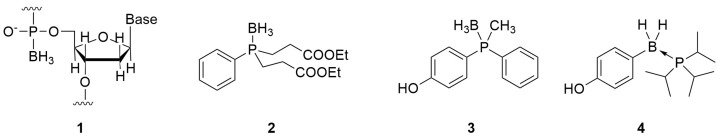
Examples of P–B bond-containing biofunctional molecules.

Phosphine–boranes are complexes of phosphines and boranes bearing P–B bonds [1,2,3]. Owing to the stability of the P–B bond in the adducts, phosphine–boranes are utilized as synthetic intermediates of phosphines: the borane moiety functions as a protective group for the oxidation-susceptible lone pair of trivalent phosphorus [4,5,6]. P–B adducts have also been investigated for biomedical applications such as boranophosphate-type nucleic acids (**1**), in which the P=O double bond of the phosphate moiety is replaced by a P–B bond [7,8]. Levin et al. developed phosphine–boranes bearing a P–BH_3_ moiety, such as compound **2**, as neuroprotective agents (Figure 1) [9,10]. From a structural arrangement perspective, the P–B substructures in phosphine–boranes can be regarded as isosteric with alkanes because of their tetrahedral sp^3^–sp^3^ character. Based on this consideration, we recently developed phosphine–borane-containing estrogen receptor (ER) modulators such as **3** and **4** [11,12]. Subsequently, we had revealed that the P–BH_2_–Ph moiety as well as P–BH_3_ moieties are useful structural options for the development of biologically active compounds, particularly for the optimization of hydrophobic substructures by controlling the hydrophobicity of the compounds. In the current study, to investigate the general usefulness of phosphine–boranes for other biological targets, we aimed to investigate the development of novel progesterone receptor (PR) antagonists based on a phosphine–borane framework.

**Figure 2:**
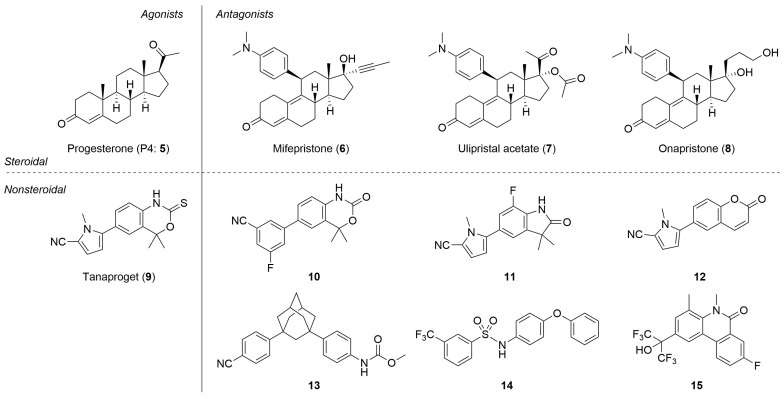
Structures of the endogenous PR agonist **5** and representative synthetic PR agonists and antagonists.

The progesterone receptor (PR) is a member of the nuclear receptor superfamily of ligand-dependent transcription factors and plays important roles in multiple physiological processes including the female reproductive system, such as in uterine cell proliferation and differentiation, ovulation cycle, and mammary gland growth and differentiation [13,14]. The PR is regulated by the endogenous steroidal agonist progesterone (P4, **5**, Figure 2). Various steroidal PR agonists have been developed and are clinically used for the treatment of gynecological disorders, contraception, and hormone replacement therapy [15,16]. In addition to PR agonists, PR antagonists have attracted considerable attention as drug candidates. Although the representative, approved PR antagonist mifepristone (**6**) is currently in limited clinical use as an abortifacient, ulipristal acetate (**7**) is used not only as a contraceptive agent but also as a treatment for uterine fibroids [17,18,19,20]. Studies using PR antagonists, such as **6** and the investigational drug onapristone (**8**), indicated that PR antagonists might be effective not only for contraception and uterine fibroids but also for the treatment of endometriosis, breast cancer, ovarian cancer, uterine cancer, and some psychiatric disorders [21,22,23,24,25,26,27]. In contrast, all PR antagonists used clinically, including **6** and **7**, are steroidal compounds. Therefore, the development of nonsteroidal PR antagonists is preferred to avoid side effects related to target selectivity and metabolic pathways. Indeed, a few steroidal PR antagonists, including **6**, demonstrate potent activities against other steroid receptors, such as the glucocorticoid receptor (GR) [28,29,30]. Thus, various nonsteroidal PR antagonists such as **10**–**13** [31,32,33,34,35,36,37] have been synthesized based on the structure of the nonsteroidal PR agonist tanaproget (**9**) [38]. These compounds have a common cyanoaryl moiety as the pharmacophore motif, and minor structural modifications of PR ligands bearing this motif can cause agonist/antagonist activity switching [32,33,38,39]. We have been investigating the development of new-generation PR antagonists and have reported that PR antagonists such as **14** and **15** are structurally distinct from conventional nonsteroidal PR antagonists (Figure 2) [40,41]. Regarding PR antagonists and ligands of other nuclear receptors, hydrophobic interactions are essential for ligand activity. Thus, we hypothesized that the application of the phosphine–borane moiety is advantageous for the structural development of the hydrophobic moiety in PR antagonists. Consequently, we developed novel phosphine–borane framework-based PR antagonists.

## 2. Results and Discussion

### 2.1. Molecular Design

**Figure 3:**
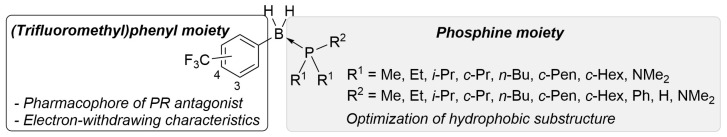
Design scheme of the *B*-(trifluoromethyl)phenyl phosphine–borane derivatives as PR antagonists.

To develop a novel nonsteroidal PR antagonist, we adopted the (trifluoromethyl)phenyl group as the key structural motif, which has been found to function as a pharmacophore for PR antagonists such as **14**. Our recent results indicated that the electronic profile of the *B*-substituents of phosphine–boranes partly influences the stability of the compounds, and an increase in the electrophilicity of the boron atom can increase the stability of the phosphine–borane adducts [12]. Thus, the introduction of an electron-withdrawing trifluoromethyl group onto the *B*-phenyl moiety is a reasonable approach for designing novel biologically active phosphine–borane derivatives. In addition, phosphine–borane frameworks are less hydrophobic than the corresponding carbon-based frameworks [11,12]; therefore, the introduction of a highly hydrophobic trifluoromethyl group on the phosphine–borane framework can provide appropriate hydrophobicity to the compounds. Regarding the phosphine moiety, various phosphines with diverse shapes and bulkiness are available because of their utility as phosphine ligands in catalysts. We assumed that the structural diversity of phosphines is particularly useful for the optimization of a hydrophobic motif in PR antagonists. Hence, we designed a series of *B*-(trifluoromethyl)phenyl phosphine–borane derivatives bearing a wide variety of phosphine moieties (Figure 3).

### 2.2. Synthesis

**Scheme 1:**
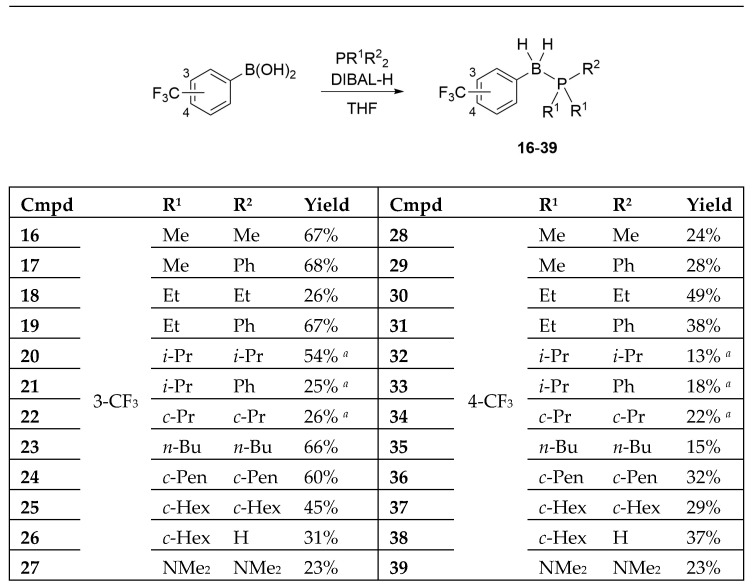
Synthesis of the designed *B*-(trifluoromethyl)phenyl phosphine–borane derivatives **16**–**39**. *^a^* The yield was calculated in two steps: (1) synthesis of phosphines without purification, (2) reductive P–B bond formation using DIBAL-H.

The designed *B*-(trifluoromethyl)phenyl phosphine–borane derivatives **16**–**39** were synthesized from the corresponding phosphines and 3- or 4-(trifluoromethyl)phenyl boronic acid under reductive conditions (Scheme 1) [12,42,43]. Interestingly, we could obtain phosphine–borane derivatives **26** and **38** containing the P–H moiety under normal preparation conditions using an aqueous workup. We also obtained tris(dimethylamino)phosphine-borane derivatives **27** and **39**.

### 2.3. Hydrophobicity

Hydrophobicity is a key determinant of the activity and pharmacokinetics of biologically active compounds, and the octanol–water partition coefficient (*P*) and logarithm value (Log*P*) are widely used hydrophobicity parameters [44]. The Log*P* values of the synthesized compounds were determined using HPLC [45,46]. Table 1 summarizes the experimentally determined Log*P* values and calculated values. The trimethylphosphine–borane derivatives **16** and **28** exhibited a Log*P* value of 3.65. In our previous study, the corresponding phenol derivative *B*-4-hydroxyphenyl trimethylphosphine–borane exhibited a Log*P* value of 2.44 [12]. The reported substituent constants of hydrophobicity of the CF_3_ and phenolic OH groups were 0.88 and −0.67, respectively [47], and therefore, the difference between the Log*P* values of trifluoride and the corresponding phenol derivative moderately agreed with the reported difference. From the viewpoint of the phosphine structure, an increase in the number of carbon atoms increased hydrophobicity in a predictable manner. Compounds **26** and **38** bearing a P–H group and two cyclohexyl groups exhibited Log*P* values of 7.28 and 7.34, respectively. These Log*P* values were similar to those of the tri-*n*-butyl derivatives **23** (Log*P* = 7.34) and **35** (Log*P* = 7.50) possessing the same number of carbon atoms (C_12_), indicating that the P–H group in the phosphine–borane derivative was almost nonpolar and did not significantly reduce the hydrophobicity of the compounds.

**Figure 4:**
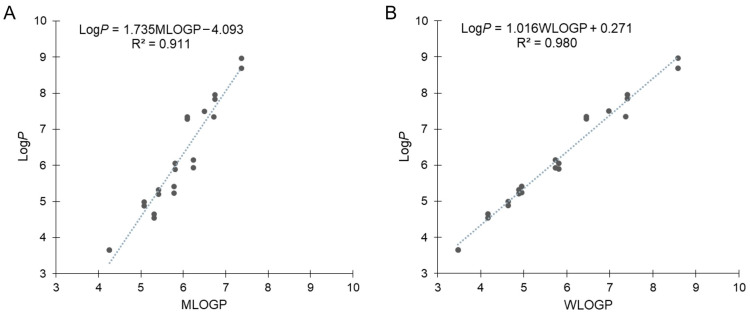
Correlation (**A**) between experimentally measured Log*P* and calculated MLOGP values and (**B**) between experimentally measured Log*P* and calculated WLOGP values with the exception of amide derivatives **27** and **39**.

Our previous results for *B*-hydroxyphenyl phosphine–borane derivatives indicated that some of these compounds showed Log*P* values with non-negligible differences from the calculated values. Therefore, we calculated the hydrophobicity parameters using SwissADME [48,49]. MLOGP is one of the most representative calculation methods of Log*P* defined by Moriguchi et al. [50,51], which is referred to as Lipinski’s Rule of Five [52,53]. Figure 4A shows the correlation between the measured Log*P* and MLOGP values, with the exception of amide compounds **27** and **39**, which is expressed by the following equation: Log*P* = 1.735MOLGP − 4.093 (R^2^ = 0.911). The correlation coefficient was above 0.9 but not sufficiently large, and moreover, the slope and intercept were significantly large. The difference between the calculated and measured values of each compound ranged from –0.77 (**17**) to +1.60 (**37**). MLOGP is a calculation method based on the topology of chemical structures using 13 molecular descriptors that are correlated with Log*P* values. Thereafter, we used the WLOGP calculation method, defined by Wildman and Crippen based on the classification of constituent atoms using 68 atomic descriptors [54]. Figure 4B shows the correlation between the measured Log*P* and WLOGP values, which was expressed by the following equation: Log*P* = 1.016WLOGP + 0.271 (R^2^ = 0.980). The correlation coefficient was close to 1.0, and the slope and intercept were smaller than those of MLOGP. These results suggest that WLOGP was more suitable for predicting the hydrophobicity of phosphine–borane derivatives.

### 2.4. PR Antagonistic Activity

**Figure 5:**
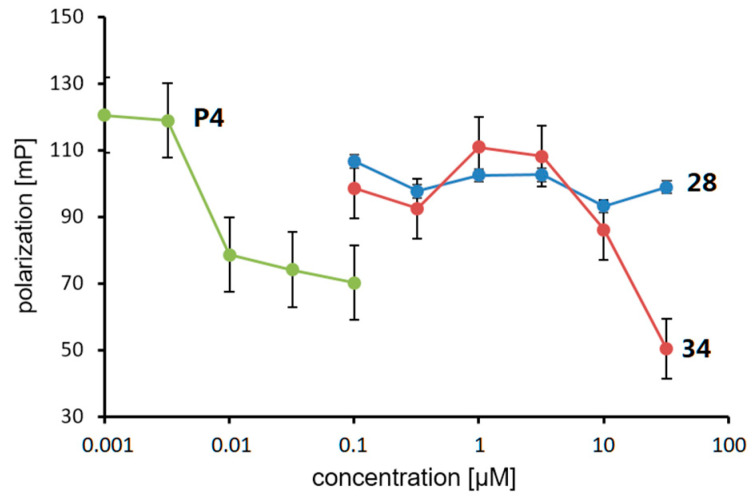
Competitive binding assay using a fluorescence polarization system.

The PR agonistic and antagonistic activities of the synthesized phosphine–borane derivatives were evaluated using an alkaline phosphatase assay with the T47D human breast cancer cell line [55]. None of the test compounds induced alkaline phosphatase activity alone, indicating that they do not act as PR agonists. The basal alkaline phosphatase activity was also not affected by the tested compounds alone. The results also indicated that these compounds exhibited no significant cytotoxicity (Figure S1). The PR antagonistic activities of the compounds were assessed in the presence of 1 nM of P4 (**5**). We also calculated the molecular volume of each compound to investigate the structure–activity relationship (SAR). Table 2 summarizes the PR antagonistic activity and calculated volumes of the synthesized compounds. All tested compounds exhibited PR antagonistic activity. Among the 3-CF_3_ derivatives **16**–**27**, the trimethylphosphine derivative **16**, tri-*n*-butylphosphine derivative **23**, and tricyclohexylphosphine derivative **25** exhibited only weak potency, and the triethylphosphine derivative **18**, diisopropyl(phenyl)phosphine derivative **21**, and tricyclopentylphosphine derivative **24** exhibited moderate PR antagonistic activity. The dimethyl(phenyl)phosphine derivative **17**, diethyl(phenyl)phosphine derivative **19**, triisopropylphosphine derivative **20**, and tricyclopropylphosphine derivative **22**, as well as the amide derivative **27**, exhibited potent activities. These results indicate that the trimethylphosphine moiety cannot sufficiently fill the hydrophobic cavity of the ligand-binding pocket of PR and that the bulky phosphine moiety is too large to enter the ligand-binding pocket. The findings also indicated that phosphines bearing 8–10 carbon atoms are suitable for the hydrophobic substructure of the designed PR antagonists, with the tricyclopropylphosphine substructure as the most suitable hydrophobic motif. The SAR of the 4-CF_3_ derivatives **28**–**39** were similar to those of 3-CF_3_ derivatives, and among the synthesized compounds, *B*-(4-trifluoromethyl)phenyl tricyclopropylphosphine–borane (**34**) was the most potent with an IC_50_ value of 0.54 μM. Interestingly, tricyclopropylphosphine derivative **34** was approximately three times more potent than triisopropylphosphine derivative **32** (IC_50_ = 1.51 μM). The differences in the molecular volumes of these compounds resulted in large differences in potency. We investigated the binding affinity of the compounds toward the PR ligand-binding domain (LBD) using a fluorescence polarization assay system. Compound **34** exhibited significant affinity toward PR LBD, whereas the less potent compound **28** did not show the affinity, indicating that the potent antagonistic activity of **34** in the alkaline phosphatase assay was mediated by the binding to PR (Figure 5). We also investigated the activity of compounds **28** and **34** toward the androgen receptor (AR) by means of cell-proliferation promoting/inhibitory activity toward androgen-dependent SC-3 cells [56]. These compounds did not affect cell growth both in the presence or absence of dihydrotestosterone, indicating that these compounds did not have AR agonistic or antagonistic activity (Tabel S1).

### 2.5. Docking Simulations

**Figure 6:**
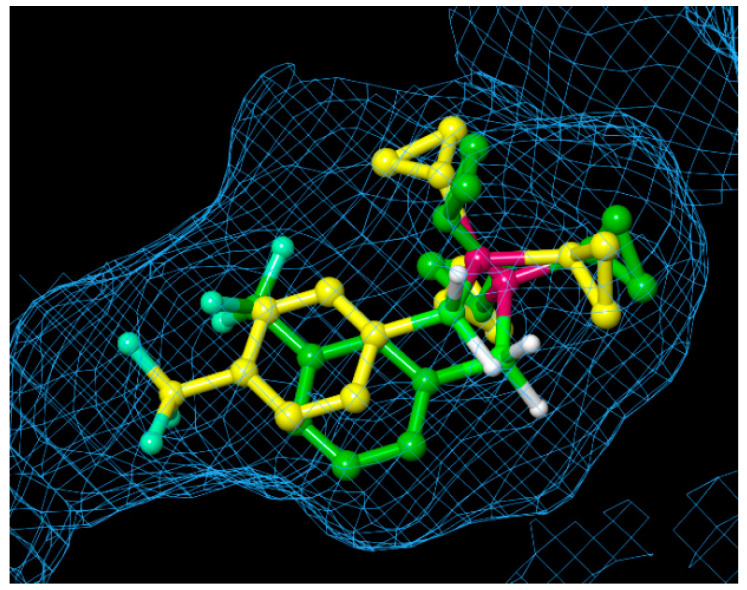
Superimposition of the docked structures of *B*-(3-trifluoromethyl)phenyl tricyclopropylphosphine–borane (**22**: carbon in green) and *B*-(4-trifluoromethyl)phenyl tricyclopropylphosphine–borane (**34**: carbon in yellow), with hPR LBD, calculated using the docking program AutoDock. The protein surface is displayed as a blue mesh.

To estimate the binding mode of the developed phosphine–borane-based PR antagonists, we conducted docking simulations of tricyclopropylphosphine derivatives **22** and **34** with the X-ray crystal structure of the nonsteroidal ligand-bound form of the hPR LBD (PDB ID: 3G8O) using AutoDock 4.2 [57,58]. Figure 6 shows the docking models of phosphine–boranes **22** and **34** in hPR LBD. In the docked structure of *B*-(4-trifluoromethyl)phenyl tricyclopropylphosphine–borane (**34**), the tricyclopropylphosphine moiety occupied the hydrophobic cavity of the ligand-binding pocket, and the 4-(trifluoromethyl)phenyl group was located in the hydrophobic cavity on the contralateral side of the phosphine moiety. In the docked structure of the 3-trifluoromethyl isomer **22**, the tricyclopropylphosphine moiety occupied the hydrophobic cavity in the same manner as that in **34**, and the 3-(trifluoromethyl)phenyl group was located near the center of the pocket. These results indicated that the tricyclopropylphosphine moiety played an important role in ligand–receptor interactions and that the optimization of the phenyl moiety could lead to the development of novel PR antagonists with improved potency. Overall, a wide variety of phosphine structures, which are difficult to construct using carbon-based functionalities, could enable fine tuning of the hydrophobic interactions, and phosphine–boranes could be a versatile option for the structural optimization of diverse drug candidates.

## 3. Materials and Methods

### 3.1. Chemistry

All the reagents were purchased from Sigma-Aldrich Inc., St. Louis, MO, USA, Tokyo Chemical Industry Co., Ltd., Tokyo, Japan, Fujifilm Wako Pure Chemical Corporation, Osaka, Japan, or Kanto Chemical Co., Inc., Tokyo, Japan, and used without further purification. Thin-layer chromatography (TLC) was performed using a silica gel coated with the fluorescent indicator F254 (Merck, Boston, MA, USA, #1.05715.0001). Silica gel column chromatography was performed using a neutral silica gel (60 Å, 40–50 μm) purchased from Kanto Chemical Co., Inc. NMR spectra were recorded using Bruker Avance 400 (^1^H: 400 MHz, ^13^C: 100 MHz, ^11^B: 128 MHz, ^19^F: 376 MHz, and ^31^P: 161 MHz) and Bruker Avance 500 (^1^H: 500 MHz and ^13^C: 125 MHz) spectrometers. High-resolution mass (HRMS) spectra were recorded on a Bruker Daltonics micrOTOF-2 focus using electron spray ionization time-of-flight (ESI-TOF).

### 3.2. General Procedure for the Synthesis of Phosphine–Borane Derivatives **16**–**39**

A dry round-bottomed flask, equipped with a magnetic stirring bar, sealed with a septum, and protected with an Ar balloon, was charged with 4- or 3-(trifluoromethyl)phenyl boronic acid (190 mg, 1.0 mmol or 570 mg, 3.0 mmol) in 1.0 mL of THF. A solution of 1.0 M diisobutyl aluminium hydride (DIBAL) in toluene (3.0 or 5.0 mmol) was added to the mixture, and the corresponding phosphine was added at 0 °C. The mixture was stirred at room temperature and monitored using TLC. Once completed, the reaction was quenched by the addition of a saturated aqueous potassium sodium tartrate solution and extracted with AcOEt. The organic layer was washed with brine and dried over Na_2_SO_4_. The solvent was evaporated, and the residue was purified using silica gel column chromatography (eluent = hexane/AcOEt) to generate the desired phosphine–borane derivatives. For compounds **20**–**22** and **32**–**34**, the corresponding phosphines were prepared immediately before addition, without purification. The details of the characterization and spectra of the compounds are presented in the Supporting Information.

#### 3.2.1. *B*-3-(Trifluoromethyl)phenyl Trimethylphosphine–Borane (**16**)

Colorless oil, Yield 67%, 154 mg; ^1^H NMR (400 MHz, DMSO-*d*_6_): δ 7.49–7.46 (m, 2H), 7.37–7.31 (m, 2H), 2.23–1.41 (br, 2H), 1.23 (d, *J* = 11.0 Hz, 9H); ^11^B NMR (128 MHz, DMSO-*d*_6_): δ −23.7 (d, *J_BP_* = 59.3 Hz); ^13^C NMR (125 MHz, CDCl_3_): δ 138.9 (d, *J_CP_* = 6.8 Hz), 131.74–131.66 (m), 129.1 (qd, *J_CF_* = 30.8 Hz, *J_CP_* = 3.3 Hz), 127.2 (d, *J_CP_* = 3.4 Hz), 125.0 (q, *J_CF_* = 272.2 Hz), 121.5–121.4 (m), 10.2 (d, *J_CP_* = 37.5 Hz); ^19^F NMR (376 MHz, DMSO-*d*_6_): δ −61.1 (s); ^31^P NMR (161 MHz, DMSO-*d*_6_): δ −6.1 (s); HRMS (ESI) *m/z* calcd. for C_10_H_15_BF_3_NaP [M + Na]^+^ 257.0849, found 257.0852.

#### 3.2.2. *B*-(3-Trifluoromethyl)phenyl Dimethyl(phenyl)phosphine–Borane (**17**)

White solid; Yield 68%, 177 mg; MP 75.0–75.8 °C; ^1^H NMR (400 MHz, DMSO-*d*_6_) δ 7.65 (t, *J* = 8.7 Hz, 2H), 7.57–7.43 (m, 4H), 7.36–7.26 (m, 3H), 2.35–1.72 (br, 2H), 1.53 (d, *J* = 10.7 Hz, 6H); ^11^B NMR (128 MHz, DMSO-*d*_6_) δ −23.8 (s); ^13^C NMR (125 MHz, CDCl_3_) δ 139.2 (d, *J_CP_* = 7.0 Hz), 132.1–132.0 (m), 131.3 (d, *J_CP_* = 2.4 Hz), 130.8 (d, *J_CP_* = 8.4 Hz), 129.3 (d, *J_CP_* = 53.5 Hz), 129.1 (qd, *J_CF_* = 31.0 Hz, *J_CP_* = 3.3 Hz), 128.9 (d, *J_CP_* = 10 Hz), 127.2 (d, *J_CP_* = 3.4 Hz), 124.9 (q, *J_CF_* = 272.1 Hz), 121.6–121.5 (m), 10.0 (d, *J_CP_* = 38.2 Hz; ^19^F NMR (376 MHz, DMSO-*d_6_*) δ −61.1 (s); ^31^P NMR (161 MHz, DMSO-*d*_6_) δ −1.2 (s); HRMS (ESI) *m/z* calcd. for C_15_H_17_BF_3_NaP [M + Na]^+^ 319.1005, found 319.0998.

#### 3.2.3. *B*-3-(Trifluoromethyl)phenyl Triethylphosphine–Borane (**18**)

Colorless oil; Yield 26%, 71 mg; ^1^H NMR (400 MHz, DMSO-*d*_6_) δ 7.50–7.48 (m, 2H), 7.35–7.29 (m, 2H), 1.63–1.54 (m, 6H), 1.04–0.96 (m, 9H); ^11^B NMR (128 MHz, DMSO-*d*_6_) δ −27.4 (d, *J_BP_* = 50.1 Hz); ^13^C NMR (125 MHz, CDCl_3_) δ 139.1 (d, *J_CP_* = 6.4 Hz), 132.0–131.9 (m), 129.0 (qd, *J_CF_* = 30.9 Hz, *J_CP_* = 3.0 Hz), 127.1 (d, *J_CP_* = 2.9 Hz), 125.0 (q, *J_CF_* = 272.3 Hz), 121.3–121.2 (m), 12.8 (d, *J_CP_* = 34.1 Hz), 6.4 (d, *J_CP_* = 3.9 Hz); ^19^F NMR (376 MHz, DMSO-*d*_6_) δ −61.1 (s); ^31^P NMR (161 MHz, DMSO-*d*_6_) δ 12.5 (s); HRMS (ESI) *m*/*z* calcd. for C_13_H_21_BF_3_NaP [M + Na]^+^ 299.1318, found 299.1325.

#### 3.2.4. *B*-3-(Trifluoromethyl)phenyl Diethyl(phenyl)phosphine–Borane (**19**)

White solid; Yield 68%, 215 mg; MP 40.0–40.5 °C; ^1^H NMR (400 MHz, CD_2_Cl_2_) δ 7.62–7.58 (m, 2H), 7.56–7.46 (m, 5H), 7.30 (d, *J* = 7.5 Hz, 1H), 7.23 (t, *J* = 7.5 Hz, 1H), 1.89–1.76 (m, 4H), 1.09–1.01 (m, 6H); ^11^B NMR (128 MHz, DMSO-*d*_6_) δ −27.1 (s); ^13^C NMR (100 MHz, DMSO-*d*_6_): δ 139.8 (d, *J_CP_* = 6.4 Hz), 132.5 (d, *J_CP_* = 7.6 Hz), 131.9–131.7 (m), 131.7 (d, *J_CP_* = 2.2 Hz), 129.3 (d, *J_CP_* = 9.3 Hz), 128.2 (qd, *J_CF_* = 30.3 Hz, *J_CP_* = 3.1 Hz), 127.9 (d, *J_CP_* = 3.0 Hz), 126.4 (d, *J_CP_* = 51.1 Hz), 125.3 (q, *J_CF_* = 272.2 Hz), 121.5–121.4 (m), 14.5 (d, *J_CP_* = 36.0 Hz), 6.9 (d, *J_CP_* = 3.0 Hz); ^19^F NMR (376 MHz, DMSO-*d*_6_) δ −61.2 (s); ^31^P NMR (161 MHz, DMSO-*d*_6_) δ 13.1 (s); ESMS (ESI) *m*/*z* calcd. for C_17_H_21_BF_3_NaP [M + Na]^+^ 347.1318, found 347.1310.

#### 3.2.5. *B*-3-(Trifluoromethyl)phenyl Triisopropylphosphine–Borane (**20**)

Colorless oil; Yield 54%, 514 mg (2 steps); ^1^H NMR (400 MHz, DMSO-*d*_6_) δ= 7.57–7.55 (m, 2H), 7.34–7.28 (m, 2H), 2.28–1.50 (br, 2H), 2.26–2.16 (m, 3H), 1.17–1.12 (m, 18H); ^11^B NMR (128 MHz, DMSO-*d*_6_) δ −28.8 (s); ^13^C NMR (125 MHz, DMSO-*d*_6_) δ 140.2 (d, *J_CP_* = 5.5 Hz), 132.3–132.2 (m), 128.0 (qd, *J_CF_* = 30.2 Hz, *J_CP_* = 3.4 Hz), 127.9 (d, *J_CP_* = 3.0 Hz), 125.4 (q, *J_CF_* = 272.2 Hz), 121.31–121.25 (m), 20.6 (d, *J_CP_* = 29.5 Hz), 18.1 (d, *J_CP_* = 1.5 Hz); ^19^F NMR (376 MHz, DMSO-*d*_6_) δ −61.2 (s); ^31^P NMR (161 MHz, DMSO-*d*_6_) δ 24.6 (s); HRMS (ESI) *m/z* calcd. for C_16_H_27_BF_3_NaP [M + Na]^+^ 341.1788, found 341.1800.

#### 3.2.6. *B*-3-(Trifluoromethyl)phenyl Diisopropyl(phenyl)phosphine–Borane (**21**)

White solid; Yield 25%, 263 mg (2 step); MP 118.7–119.1 °C; ^1^H NMR (400 MHz, CD_2_Cl_2_) δ 7.80–7.76 (m, 2H), 7.69 (s, 1H), 7.62 (d, *J* = 6.9 Hz, 1H), 7.59–7.50 (m, 3H), 7.29 (d, *J* = 7.3 Hz, 1H), 7.22 (t, *J* = 7.5 Hz, 1H), 2.72–1.90 (br, 2H), 2.49–2.39 (m, 2H), 1.08–0.95 (m, 12H); ^11^B NMR (128 MHz, DMSO-*d*_6_) δ −28.5 (s); ^13^C NMR (100 MHz, DMSO-*d*_6_) δ 140.2 (d, *J_CP_* = 6.8 Hz), 134.1 (d, *J_CP_* = 6.7 Hz), 132.3–132.2 (m), 132.0 (d, *J_CP_* = 2.2 Hz), 129.2 (d, *J_CP_* = 8.9 Hz), 128.2 (qd, *J_CF_* = 27.6 Hz, *J_CP_* = 2.5 Hz), 127.9 (d, *J_CP_* = 2.2 Hz), 125.4 (q, *J_CF_* = 272.2 Hz), 123.5 (d, *J_CP_* = 46.9 Hz), 121.53–121.46 (m), 20.7 (d, *J_CP_* = 32.7 Hz), 16.8–16.7 (m); ^19^F NMR (376 MHz, DMSO-*d*_6_) δ −61.2 (s); ^31^P NMR (161 MHz, DMSO-*d*_6_) δ 22.1 (s); HRMS (ESI) *m/z* calcd. for C_19_H_25_BF_3_NaP [M + Na]^+^ 375.1631, found 375.1653.

#### 3.2.7. *B*-3-(Trifluoromethyl)phenyl Tricyclopropylphosphine–Borane (**22**)

Colorless oil; Yield 26%, 80 mg (2 steps); ^1^H NMR (400 MHz, DMSO-*d*_6_) δ 7.54–7.52 (m, 2H), 7.34–7.26 (m, 2H), 2.00–1.05 (br, 2H), 0.80–0.62 (m, 15H); ^11^B NMR (128 MHz, DMSO-*d*_6_) δ −29.1 (s); ^13^C NMR (125 MHz, CDCl_3_) δ 139.5 (d, *J_CP_* = 6.9 Hz), 132.5–132.4 (m), 128.7 (q, *J_CF_* = 31.4 Hz), 126.9 (d, *J_CP_* = 2.5 Hz), 125.0 (q, *J_CF_* = 272.3 Hz), 121.1–121.0 (m), 2.1 (d, *J_CP_* = 3.0 Hz), 1.6 (d, *J_CP_* = 58.3 Hz); ^19^F NMR (376 MHz, DMSO-*d*_6_) δ −61.1 (s); ^31^P NMR (161 MHz, DMSO-*d*_6_) δ 17.3 (s); HRMS (ESI) *m/z* calcd. for C_16_H_21_BF_3_NaP [M + Na]^+^ 335.1318, found 335.1323.

#### 3.2.8. *B*-3-(Trifluoromethyl)phenyl Tri-*n*-Butylphosphine–Borane (**23**)

Colorless oil; Yield 66%, 238 mg; ^1^H NMR (400 MHz, DMSO-*d*_6_) δ 7.47 (s, 2H), 7.36–7.30 (m, 2H), 1.57–1.51 (m, 6H), 1.38–1.29 (m, 12H), 0.85 (t, *J* = 7.0 Hz, 9H); ^11^B NMR (128 MHz, DMSO-*d*_6_) δ −26.8 (s); ^13^C NMR (125 MHz, CDCl_3_) δ 139.0 (d, *J_CP_* = 6.4 Hz), 132.0–131.9 (m), 128.9 (qd, *J_CF_* = 30.8 Hz, *J_CP_* = 3.4 Hz), 127.1 (d, *J_CP_* = 3.4 Hz), 125.0 (q, *J_CF_* = 272.2 Hz), 121.2–121.1 (m), 24.4 (d, *J_CP_* = 2.8 Hz), 24.3 (d, *J_CP_* = 12.5 Hz), 20.2 (d, *J_CP_* = 33.0 Hz), 13.4 (s); ^19^F NMR (376 MHz, DMSO-*d*_6_) δ −61.2 (s); ^31^P NMR (161 MHz, DMSO-*d*_6_) δ 7.03 (s); ESMS (ESI) *m*/*z* calcd. for C_19_H_33_BF_3_NaP [M + Na]^+^ 383.2257, found 383.2276.

#### 3.2.9. *B*-3-(Trifluoromethyl)phenyl Tricyclopentylphosphine–Borane (**24**)

Colorless oil; Yield 60%, 239 mg; ^1^H NMR (400 MHz, DMSO-*d*_6_) δ 7.56–7.53 (m, 2H), 7.32–7.26 (m, 2H), 2.23–1.69 (br, 2H), 2.19–2.10 (m, 3H), 1.81–1.79 (m, 6H), 1.58–1.50 (m, 18H); ^11^B NMR (128 MHz, DMSO-*d*_6_) δ −28.4 (s); ^13^C NMR (125 MHz, CDCl_3_) δ 139.6 (d, *J_CP_* = 6.0 Hz), 132.8–132.7 (m), 128.7 (qd, *J_CF_* = 30.7 Hz, *J_CP_* = 2.9 Hz), 126.8 (d, *J_CP_* = 2.8 Hz), 125.0 (q, *J_CF_* = 272.3 Hz), 121.0–120.9 (m), 32.4 (d, *J_CP_* = 31.9 Hz), 28.3(s), 26.0 (d, *J_CP_* = 8.8 Hz); ^19^F NMR (376 MHz, DMSO-*d*_6_) δ −61.2 (s); ^31^P NMR (161 MHz, DMSO-*d*_6_) δ 19.1 (s); HRMS (ESI) *m/z* calcd. for C_22_H_33_BF_3_NaP [M + Na]^+^ 419.2257, found 419.2265.

#### 3.2.10. *B*-3-(Trifluoromethyl)phenyl Tricyclohexylphosphine–Borane (**25**)

White solid; Yield:45%, 213 mg; MP 100.0–100.5 °C; ^1^H NMR (400 MHz, DMSO-*d*_6_) δ 7.53 (s, 2H), 7.35–7.30 (m, 2H), 2.08–1.10 (br, 2H),1.94–1.65 (m, 18H), 1.32–1.14 (m, 15H); ^11^B NMR (128 MHz, DMSO-*d*_6_) δ −28.8 (s); ^13^C NMR (125 MHz, DMSO-*d*_6_) δ 140.2 (d, *J_CP_* = 5.2 Hz), 132.4–132.3 (m), 128.0 (qd, *J_CF_* = 30.1 Hz, *J_CP_* = 2.6 Hz), 128.0 (d, *J_CP_* = 2.3 Hz), 125.5 (q, *J_CF_* = 272.6 Hz), 121.33–121.27 (m), 30.3 (d, *J_CP_* = 28.7 Hz), 27.8 (d, *J_CP_* = 1.4 Hz), 27.2 (d, *J_CP_* = 10.0 Hz), 26.1 (s); ^19^F NMR (376 MHz, DMSO-*d*_6_) δ −61.2 (s); ^31^P NMR (161 MHz, DMSO-*d*_6_) δ 15.7 (s); HRMS (ESI) *m/z* calcd. for C_25_H_39_BF_3_NaP [M + Na]^+^ 461.2727, found 461.2739.

#### 3.2.11. *B*-3-(Trifluoromethyl)phenyl Dicyclohexylphosphine–Borane (**26**)

Colorless oil; Yield 31%, 109 mg; ^1^H NMR (400 MHz, acetone-*d*_6_) δ 7.65–7.60 (m, 2H), 7.35–7.28 (m, 2H), 4.26 (d, *J* = 357.0 Hz, 1H), 2.82–1.66 (br, 2H), 2.12–2.01 (m, 2H), 1.85–1.66 (m, 10H), 1.47–1.15 (m, 10H); ^11^B NMR (128 MHz, DMSO-*d*_6_) δ −29.18 (s); ^13^C NMR (100 MHz, acetone-*d*_6_) δ 140.4 (d, *J_CP_* = 7.0 Hz), 132.8–132.7 (m), 129.5 (qd, *J_CF_* = 30.3 Hz, *J_CP_* = 2.9 Hz), 128.3 (d, *J_CP_* = 2.6 Hz), 126.2 (q, *J_CF_* = 271.5 Hz), 121.9–121.8 (m), 30.7 (s), 29.8 (d, *J_CP_* = 31.1 Hz), 29.1 (s), 27.4 (d, *J_CP_* = 10.3 Hz), 27.2 (d, *J_CP_* = 11.5 Hz), 26.5 (s); ^19^F NMR (376 MHz, DMSO-*d*_6_): δ −61.12 (s); ^31^P NMR (161 MHz, DMSO-*d*_6_) δ 10.0 (d, *J* = 353.3 Hz); HRMS (ESI) *m/z* calcd. for C_19_H_29_BF_3_NaP [M + Na]^+^ 379.1944, found 379.1929.

#### 3.2.12. *B*-3-(Trifluoromethyl)phenyl Tris(dimethylamino)phosphine–Borane (**27**)

Colorless oil; Yield:23%, 75 mg; ^1^H NMR (400 MHz, DMSO-*d*_6_) δ 7.57–7.53 (m, 2H), 7.33–7.527 (m, 2H), 2.52 (d, *J* = 9.1 Hz, 18H), 2.30–1.56 (br, 2H); ^11^B NMR (128 MHz, DMSO-*d*_6_) δ −27.1 (d, *J* = 104.8 Hz); ^13^C NMR (125 MHz, DMSO-*d*_6_): δ 140.3 (d, *J_CP_* = 8.1 Hz), 132.3–132.2 (m), 127.8 (qd, *J_C__F_* = 30.3 Hz, *J_CP_* = 2.9 Hz), 127.7 (s), 125.5 (q, *J_CF_* = 272.2 Hz), 121.18–121.12 (m), 37.3 (d, *J_CP_* = 3.4 Hz); ^19^F NMR (376 MHz, DMSO-*d*_6_) δ −61.1 (s); ^31^P NMR (161 MHz, DMSO-*d*_6_) δ 92.3 (s); HRMS (ESI) *m/z* calcd. for C_13_H_24_BF_3_N_3_NaP [M + Na]^+^ 344.1645, found 344.1640.

#### 3.2.13. *B*-4-(Trifluoromethyl)phenyl Trimethylphosphine–Borane (**28**)

Colorless oil; Yield: 24%, 55 mg; ^1^H NMR (400 MHz, DMSO-*d*_6_) δ 7.41 (s, 4H), 2.29–1.42 (br, 2H), 1.23 (d, *J* = 10.9 Hz, 9H); ^11^B NMR (128 MHz, DMSO-*d*_6_): δ −23.8 (d, *J_BP_* = 63.1 Hz); ^13^C NMR (125 MHz, CDCl_3_) δ 135.6 (d, *J_CP_* = 7.0 Hz), 126.8 (qd, *J_CF_* = 31.5 Hz, *J_CP_* = 4.7 Hz), 125.0 (qd, *J_CF_* = 271.3 Hz, *J_CP_* = 1.7 Hz), 123.7 (s), 10.3 (d, *J_CP_* = 37.5 Hz); ^19^F NMR (376 MHz, DMSO-*d*_6_) δ −55.9 (s); ^31^P NMR (161 MHz, DMSO-*d*_6_) δ −6.2 (s); HRMS (ESI) *m/z* calcd. for C_10_H_15_BF_3_NaP [M + Na]^+^ 257.0849, found 257.0842.

#### 3.2.14. *B*-4-(Trifluoromethyl)phenyl Dimethyl(phenyl)phosphine–Borane (**29**)

White solid; Yield:28%, 73 mg; MP 78.0–78.3 °C; ^1^H NMR (400 MHz, DMSO-*d*_6_) δ 7.69–7.66 (m, 2H), 7.58–7.49 (m, 3H), 7.38 (s, 4H), 2.30–1.72 (br, 2H), 1.54 (d, *J* = 10.7 Hz, 6H); ^11^B NMR (128 MHz, DMSO-*d*_6_) δ −23.8 (s); ^13^C NMR (100 MHz, DMSO-*d*_6_) δ 136.3 (d, *J_CP_* = 7.2 Hz), 131.6 (d, *J_CP_* = 2.1 Hz), 131.4 (d, *J_CP_* = 8.8 Hz), 130.1 (d, *J_CP_* = 54.4 Hz), 129.3 (d, *J_CP_* = 9.6 Hz), 125.7 (qd, *J_CF_* = 31.2 Hz, *J_CP_* = 4.3 Hz), 125.5 (qd, *J_CF_* = 270.9 Hz, *J_CP_* = 1.3 Hz), 123.67–13.60 (m), 9.7 (d, *J_CP_* = 38.8 Hz); ^19^F NMR (376 MHz, DMSO-*d*_6_) δ −60.8 (d, *J* = 4.1 Hz); ^31^P NMR (161 MHz, DMSO-*d*_6_) δ −1.4 (s); HRMS (ESI) *m/z* calcd. for C_15_H_17_BF_3_NaP [M + Na]^+^ 319.1005, found 319.1013.

#### 3.2.15. *B*-4-(Trifluoromethyl)phenyl Triethylphosphine–Borane (**30**)

Colorless oil; Yield:49%, 135 mg; ^1^H NMR (400 MHz, DMSO-*d*_6_) δ 7.41 (s, 4H), 2.24–1.43 (br, 2H), 1.63–1.55 (m, 6H), 1.05–0.97 (m, 9H); ^11^B NMR (128 MHz, DMSO-*d*_6_) δ −27.6 (d, *J_BP_* = 53.0 Hz); ^13^C NMR (125 MHz, CDCl_3_) δ 135.8 (d, *J_CP_* = 6.6 Hz), 126.5 (qd, *J_CF_* = 31.7 Hz, *J_CP_* = 4.2 Hz), 125.0 (qd, *J_CF_* = 271.6 Hz), 123.6–123.5 (m), 12.8 (d, *J_CP_* = 34.1 Hz), 6.4 (d, *J_CP_* = 3.8 Hz); ^19^F NMR (376 MHz, DMSO-*d*_6_) δ −60.7 (d, *J_FP_* = 3.0 Hz); ^31^P NMR (161 MHz, DMSO-*d*_6_) δ 12.8 (s); HRMS (ESI) *m/z* calcd. for C_13_H_21_BF_3_NaP [M + Na]^+^ 299.1318, found 299.1319.

#### 3.2.16. *B*-4-(Trifluoromethyl)phenyl Diethyl(phenyl)phosphine–Borane (**31**)

White solid; Yield:38%, 121 mg; MP 62.0–62.3 °C; ^1^H NMR (400 MHz, DMSO-*d*_6_) δ 7.75–7.70 (m, 2H), 7.61–7.52 (m, 3H), 7.39 (s,4H), 2.45–1.76 (br, 2H), 2.02–1.77 (m, 4H), 0.98–0.90 (m, 6H); ^11^B NMR (128 MHz, DMSO-*d*_6_) δ −27.3 (s); ^13^C NMR (125 MHz, CDCl_3_) δ 135.9 (d, *J_CP_* = 7.1 Hz), 132.1 (d, *J_CP_* = 7.5 Hz), 131.3 (d, *J_CP_* = 2.5 Hz), 128.9 (d, *J_CP_* = 9.4 Hz), 126.8 (qd, *J_CF_* = 31.7 Hz, *J_CP_* = 4.2 Hz), 126.1 (d, *J_CP_* = 50.3 Hz), 125.0 (q, *J_CF_* = 271.5 Hz), 123.61–123.56 (m), 15.1 (d, *J_CP_* = 35.6 Hz), 6.7 (d, *J_CP_* = 3.0 Hz); ^19^F NMR (376 MHz, DMSO-*d*_6_) δ −60.7 (d, *J* = 3.8 Hz); ^31^P NMR (161 MHz, DMSO-*d_6_*) δ 13.3 (s); ESMS (ESI) *m*/*z* calcd. for C_17_H_21_BF_3_PNa [M + Na]^+^ 347.1318, found 347.1317.

#### 3.2.17. *B*-4-(Trifluoromethyl)phenyl Triisopropylphosphine–Borane (**32**)

White solid; Yield:13%, 123 mg (2 steps); MP 59.0–59.4 °C; ^1^H NMR (400 MHz, DMSO-*d*_6_) δ 7.49 (d, *J* = 7.4 Hz, 2H), 7.39 (d, *J* = 8.0 Hz, 2H), 2.30–1.52 (br, 2H), 2.28–2.18 (m, 3H), 1.17–1.12 (m, 18H); ^11^B NMR (128 MHz, DMSO-*d*_6_) δ −28.7 (d, *J* = 37.6 Hz,); ^13^C NMR (125 MHz, CDCl_3_) δ 136.3 (d, *J_CP_* = 6.2 Hz), 126.5 (q, *J_CF_* = 30.0 Hz), 125.1 (q, *J_CF_* = 271.4 Hz), 123.4 (s), 20.8 (d, *J_CP_* = 29.2 Hz), 18.0 (s); ^19^F NMR (376 MHz, DMSO-*d*_6_) δ −60.6 (d, *J* = 3.5 Hz); ^31^P NMR (161 MHz, DMSO-*d*_6_) δ 24.9 (s); HRMS (ESI) *m/z* calcd. for C_16_H_27_BF_3_NaP [M + Na]^+^ 341.1788, found 341.1791.

#### 3.2.18. *B*-4-(Trifluoromethyl)phenyl Diisopropyl(phenyl)phosphine–Borane (**33**)

White solid; Yield 18%, 186 mg (2 steps); MP 94.8–95.2 °C; ^1^H NMR (400 MHz, DMSO-*d*_6_) δ 7.87–7.65 (m, 2H), 7.65–7.57 (m, 3H), 7.52 (d, *J* = 7.1 Hz, 2H), 7.39 (d, *J* = 8.0 Hz, 2H), 2.65–2.00 (br, 2H), 2.63–2.53 (m, 2H), 1.01–0.86 (m, 12H); ^11^B NMR (128 MHz, DMSO-*d*_6_) δ −28.9 (s); ^13^C NMR (125 MHz, CDCl_3_) δ 136.3 (d, *J_CP_* = 7.0 Hz), 133.6 (d, *J_CP_* = 6.5 Hz), 131.3 (d, *J_CP_* = 2.4 Hz), 128.6 (d, *J_CP_* = 8.7 Hz), 126.7 (qd, *J_CF_* = 31.8 Hz, *J_CP_* = 3.8 Hz), 125.0 (q, *J_CF_* = 271.2 Hz), 123.9 (s), 123.53–123.48 (m), 21.2 (d, *J_CP_* = 31.8 Hz), 16.7–16.5 (m); ^19^F NMR (376 MHz, DMSO-*d*_6_) δ −60.7 (d, *J* = 2.8 Hz); ^31^P NMR (161 MHz, DMSO-*d*_6_) δ 22.5 (s); HRMS (ESI) *m/z* calcd. for C_19_H_25_BF_3_NaP [M + Na]^+^ 375.1631, found 375.1632.

#### 3.2.19. *B*-4-(Trifluoromethyl)phenyl Tricyclopropylphosphine–Borane (**34**)

White solid; Yield:22%, 68 mg (2 steps); MP 72.6–72.8 °C; ^1^H NMR (400 MHz, DMSO-*d*_6_) δ 7.46 (d, *J* = 6.9 Hz, 2H), 7.38 (d, *J* = 8.1 Hz, 2H), 1.99–1.12 (br, 2H), 0.81–0.66 (m, 15H); ^11^B NMR (128 MHz, DMSO-*d*_6_) δ −29.4 (s); ^13^C NMR (125 MHz, DMSO-*d*_6_) δ 136.6 (d, *J_CP_* = 6.8 Hz), 125.5 (qd, *J_CF_* = 271.4 Hz), 125.4 (qd, *J_CF_* = 31.1 Hz, *J_CP_* = 4.1 Hz), 123.42–123.39 (m), 2.37 (d, *J_CP_* = 3.3 Hz), 1.65 (d, *J_CP_* = 59.3 Hz); ^19^F NMR (376 MHz, DMSO-*d*_6_) δ −60.6 (d, *J* = 4.0 Hz); ^31^P NMR (161 MHz, DMSO-*d*_6_) δ 17.7 (s); HRMS (ESI) *m/z* calcd. for C_16_H_21_BF_3_NaP [M + Na]^+^ 335.1318, found 335.1321.

#### 3.2.20. *B*-4-(Trifluoromethyl)phenyl Tri-n-butylphosphine–Borane (**35**)

White solid; Yield:15%, 54 mg; MP 33.2–33.8 °C; ^1^H NMR (400 MHz, DMSO-*d*_6_) δ 7.41 (s, 4H), 1.59–1.52 (m, 6H), 2.10–1.48 (br, 2H), 1.38–1.29 (m, 12H), 0.85 (t, *J* = 7.0 Hz, 9H); ^11^B NMR (128 MHz, DMSO-*d*_6_) δ −26.8 (s); ^13^C NMR (125 MHz, DMSO-*d*_6_) δ 136.2 (d, *J_CP_* = 6.6 Hz), 125.54 (q, *J_CF_* = 270.9 Hz), 125.48 (qd, *J_CF_* = 31.1 Hz, *J_CP_* = 4.0 Hz), 123.7–123.6 (m), 24.43 (d, *J_CP_* = 2.7 Hz), 24.37 (d, *J_CP_* = 12.5 Hz), 20.1 (d, *J_CP_* = 33.8 Hz), 13.9 (s); ^19^F NMR (376 MHz, DMSO-*d*_6_) δ −60.7 (s); ^31^P NMR (161 MHz, DMSO-*d*_6_) δ 7.4 (s); ESMS (ESI) calcd. for C_19_H_33_BF_3_PNa [M + Na]^+^ 383.2257, found 383.2270.

#### 3.2.21. *B*-4-(Trifluoromethyl)phenyl Tricyclopentylphosphine–Borane (**36**)

White solid; Yield:32%, 126 mg; MP 88.0–88.6 °C; ^1^H NMR (400 MHz, DMSO-*d*_6_) δ 7.48 (d, *J* = 7.0 Hz, 2H), 7.38 (d, *J* = 7.8 Hz, 2H), 2.24–1.73 (br, 2H), 2.20–2.12 (m, 3H), 1.82–1.80 (m, 6H), 1.58–1.50 (m, 18H); ^11^B NMR (128 MHz, DMSO-*d*_6_) δ −28.9 (s); ^13^C NMR (125 MHz, CDCl_3_) δ 136.4 (d, *J_CP_* = 6.0 Hz), 126.4 (qd, *J_CF_* = 31.6 Hz, *J_CP_* = 4.0 Hz), 125.1 (q, *J_CF_* = 271.1 Hz), 123.31–123.26 (m), 34.3 (d, *J_CP_* = 32.0 Hz), 28.4 (s), 26.1 (d, *J_CP_* = 8.4 Hz); ^19^F NMR (376 MHz, DMSO-*d*_6_) δ −60.9 (t, *J* = 2.3 Hz); ^31^P NMR (161 MHz, DMSO-*d*_6_) δ 19.4 (s); HRMS (ESI) *m/z* calcd. for C_22_H_33_BF_3_NaP [M + Na]^+^ 419.2257, found 419.2247.

#### 3.2.22. *B*-4-(Trifluoromethyl)phenyl Tricyclohexylphosphine–Borane (**37**)

White solid; Yield:29%, 140 mg; MP 126.7–127.0 °C; ^1^H NMR (400 MHz, DMSO-*d*_6_) δ 7.47–7.40 (m, 4H), 1.98–1.10 (br, 2H), 1.93–1.65 (m, 18H), 1.32–1.20 (m, 15H); ^11^B NMR (128 MHz, DMSO-*d*_6_) δ −28.8 (s); ^13^C NMR (125 MHz, DMSO-*d*_6_): δ 136.8 (d, *J_CP_* = 6.3 Hz), 125.5 (d, *J_CF_* = 271.7 Hz), 125.4 (qd, *J_CF_* = 31.1 Hz, *J_CP_* = 3.6 Hz), 123.6 (s), 30.4 (d, *J_CP_* = 28.7 Hz), 27.8 (s), 27.2 (d, *J_CP_* = 10.0 Hz), 26.2 (s); ^19^F NMR (376 MHz, DMSO-*d*_6_) δ −69.6 (d, *J* = 2.8 Hz); ^31^P NMR (161 MHz, DMSO-*d*_6_) δ 16.2 (s); HRMS (ESI) *m/z* calcd. for C_25_H_39_BF_3_ClP [M + Cl]^−^ 473.2529, found 473.2529.

#### 3.2.23. *B*-4-(Trifluoromethyl)phenyl Dicyclohexylphosphine–Borane (**38**)

White solid; Yield 37%, 132 mg, MP 52.7–53.7 °C; ^1^H NMR (400 MHz, acetone-*d*_6_) δ 7.54 (d, *J* = 7.1 Hz, 2H), 7.40 (d, *J* = 8.0 Hz, 2H), 4.27 (d, *J* = 357.0 Hz, 1H), 2.45–1.63 (br, 2H), 2.13–2.03 (m, 2H), 1.86–1.66 (m, 10H), 1.46–1.25 (m, 10H); ^11^B NMR (128 MHz, DMSO-*d*_6_) δ −29.4 (s); ^13^C NMR (100 MHz, acetone-*d*_6_) δ 137.0 (d, *J_CP_* = 7.7 Hz), 126.9 (qd, *J_CF_* = 31.4 Hz, *J_CP_* = 4.1 Hz), 126.3 (qd, *J_CF_* = 270.8 Hz, *J_CP_* = 1.3 Hz), 124.3–124.2 (m), 30.1 (s), 29.8 (d, *J_CP_* = 31.3 Hz), 29.1 (s), 27.4 (d, *J_CP_* = 10.4 Hz), 27.2 (d, *J_CP_* = 11.7 Hz), 26.5 (d, *J_CP_* = 1.1 Hz); ^19^F NMR (376 MHz, DMSO-*d*_6_) δ −60.7 (d, *J_FP_* = 3.4 Hz); ^31^P NMR (161 MHz, DMSO-*d*_6_) δ 10.5 (d, *J* = 368.2 Hz); HRMS (ESI) *m/z* calcd. for C_19_H_29_BF_3_NaP [M + Na]^+^ 379.1944, found 379.1964.

#### 3.2.24. *B*-4-(Trifluoromethyl)phenyl Tris(dimethylamino)phosphine–Borane (**39**)

White solid; Yield:23%, 73 mg; MP 41.1–41.6 °C; ^1^H NMR (400 MHz, DMSO-*d*_6_) δ 7.48 (d, *J* = 7.1 Hz, 2H), 7.39 (d, *J* = 8.1 Hz, 2H), 2.53 (d, *J* = 9.1 Hz, 18H), 2.30–1.56 (br, 2H); ^11^B NMR (128 MHz, DMSO-*d*_6_) δ −27.2 (d, *J* = 102.7 Hz); ^13^C NMR (125 MHz, DMSO-*d*_6_): δ 136.8 (d, *J_CP_* = 7.8 Hz), 125.6 (q, *J_CF_* = 271.7 Hz), 125.3 (qd, *J_CF_* = 31.2 Hz, *J_CP_* = 3.9 Hz), 123.42–123.37 (m), 37.4 (d, *J_CP_* = 3.3 Hz); ^19^F NMR (376 MHz, DMSO-*d*_6_) δ −60.6 (d, *J* = 3.9 Hz); ^31^P NMR (161 MHz, DMSO-*d*_6_) δ 92.5 (s); HRMS (ESI) *m/z* calcd. for C_13_H_24_BF_3_N_3_KP [M + K]^+^ 360.1385, found 360.1398.

### 3.3. Determination of Hydrophobicity (LogP)

The 1-octanol/water partition coefficient *P* was determined using HPLC based on the OECD Guideline for Testing Chemicals. A COSMOSIL Packed Column 5C18-MS-II (5 μm, 150 mm × 4.6 mm id, Nacalai Tesque, Inc., Kyoto, Japan) was fitted on an HPLC instrument (Multiwavelength Detector, MD-2010 Plus. JASCO, Tokyo, Japan) equipped with a pump (PU-2080, JASCO) and an oven (SSC-2120, Senshu Scientific Co., Ltd., Tokyo, Japan). The injection volume was 20 µL; the mobile phase was methanol–water 75% (*v*/*v*); and the flow rate was 1.0 mL/min in all cases. Compounds were detected by measuring the UV absorption at 240 nm. The temperature of the column was kept at 40.0 (± 0.1) °C during the measurement. The measurement was performed in triplicate, and the mean value was calculated. The dead time t_0_ was measured with thiourea as the unretained compound, and the capacity factor *k* was calculated using the equation log*k* = log (t_r_ − t_0_/t_0_), where t_r_ represents the retention time of the compound. A calibration graph was determined experimentally using reference compounds (4-methylphenol, 4-chlorophenol, 4-phenylphenol, diphenylether, fluoranthene, and dichlorodiphenyltrichloroethane) with known log*P* values. The Log*P* values of phosphine–boranes were calculated based on a calibration graph (Log*P* = 2.6269 log*k*_w_ + 3.059, R^2^ = 0.9915).

### 3.4. T47D Alkaline Phosphatase Assay for the Evaluation of PR Antagonistic Activity

T47D alkaline phosphatase assays were performed as previously described, with minor modifications [40]. Briefly, the human breast cancer cell line T47D (HMS LINCS database Accession Numbers: 50541) was routinely cultivated in RPMI 1640 medium with 10% FBS at 37 °C in a 5% CO_2_ humidified incubator. The cells were plated in 96-well plates and incubated overnight in a 5% CO_2_ humidified incubator at 37 °C. The next day, the cells were treated with a fresh medium containing the test compound in the presence of 1 nM of progesterone and further incubated for 48 h. The medium was aspirated, and the cells were fixed with 100 μL of 1.8% formalin–PBS. The fixed cells were washed with PBS, and 100 μL of an assay buffer (1 mg/mL *p*-nitrophenol phosphate in diethanolamine water solution, pH 9.0) was added. The mixture was incubated at room temperature for 2 h under light-shielded conditions. The absorbance was measured at 405 nm using a DTX 880 Multimode Detector (Beckman Coulter, Brea, CA, USA). All data points were measured in triplicate, and IC_50_ values were calculated from three independent experiments.

### 3.5. PR LBD Competitive Binding Assay

PR binding affinity was assessed using a PolarScreen Progesterone Receptor Competitor Assay Kit, Green (Invitrogen, Waltham, MA, USA, A15905) based on the manufacturer’s instruction. Briefly, PR-LBD(GST)/Fluormone PR Green Complex (final concentration: 6.5 nM) and test compounds (final concentration: 100 nM to 32 μM for the phosphine-boranes and 1 nM–320 nM for P4) in the assay buffer (final volume: 32 μM) were mixed on a 384-well plate (black, polypropylene). Then, the mixture was incubated at room temperature for 1 h under shade. The fluorescence polarization value (mP) was measured at 485 nm/535 nm (excitation/emission) on a DTX 880 Multimode Detector (Beckman Coulter).

### 3.6. Docking Simulation

The structure of the LBD of hPR was prepared from the Protein Data Bank accession number 3G8O [56]. Polar hydrogen atoms and partial atomic charges were assigned using AutoDockTools (ADT). Molecular docking was performed using AutoDock 4.2 with the genetic algorithm. The AutoDock parameters for boron atoms were Rii = 4.08 and eii = 0.180.

## 4. Conclusions

To expand the utility of phosphine–boranes in medicinal chemistry, we designed and synthesized a series of *B*-(trifluoromethyl)phenyl phosphine–borane derivatives and investigated their hydrophobic characteristics and biological activity toward the PR. The synthesized *B*-(trifluoromethyl)phenyl phosphine–borane derivatives exhibited predictable Log*P* values. We also demonstrated that the P–H group in phosphine–borane was nonpolar. Biological evaluation revealed that all synthesized phosphine–boranes, except for the secondary phosphine derivatives, exhibited PR antagonistic activity. The potency of the compounds depended on the bulkiness of the phosphine moiety, and the tricyclopropylphosphine substructure was found to be the most suitable for the designed PR antagonists. Docking simulations suggested that the tricyclopropylphosphine moiety plays an important role in ligand–receptor interactions and that optimization of the phenyl moiety could lead to the development of novel PR antagonists with improved potency. These results support the idea that phosphine–boranes are versatile structural options in medicinal chemistry for a wide variety of drug candidates, and the developed compounds, including **22** and **34**, are promising lead compounds for further structural development of next-generation PR antagonists.

## Figures and Tables

**Table 1:** Determined Log*P* values and calculated hydrophobicity parameters MLOGP and WLOGP of the synthesized phosphine–borane derivatives **16**–**39**.

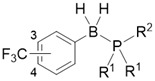								
Cmpd		R^1^	R^2^	Log*P*	MLOGP	Δ(Log*P*−MLOGP)	WLOGP	Δ(Log*P*−WLOGP)
**16**	3-CF_3_	Me	Me	3.65	4.26	−0.61	3.47	0.18
**17**		Me	Ph	4.54	5.31	−0.77	4.17	0.37
**18**		Et	Et	4.88	5.07	−0.19	4.64	0.24
**19**		Et	Ph	5.24	5.78	−0.54	4.95	0.29
**20**		*i*-Pr	*i*-Pr	5.89	5.81	0.08	5.81	0.08
**21**		*i*-Pr	Ph	5.93	6.24	−0.31	5.73	0.20
**22**		*c*-Pr	*c*-Pr	5.21	5.41	−0.20	4.88	0.33
**23**		*n*-Bu	*n*-Bu	7.34 ^a^	6.72	0.62	7.37	−0.03
**24**		*c*-Pen	*c*-Pen	7.84 ^a^	6.75	1.09	7.41	0.43
**25**		*c*-Hex	*c*-Hex	8.69 ^a^	7.37	1.32	8.58	0.11
**26**		*c*-Hex	H	7.28 ^a^	6.10	1.18	6.45	0.83
**27**		NMe_2_	NMe_2_	5.39	3.00	2.39	3.01	2.38
**28**	4-CF_3_	Me	Me	3.65	4.26	−0.61	3.47	0.18
**29**		Me	Ph	4.65	5.31	−0.66	4.17	0.48
**30**		Et	Et	4.99	5.07	−0.08	4.64	0.35
**31**		Et	Ph	5.41	5.78	−0.37	4.95	0.46
**32**		*i*-Pr	*i*-Pr	6.06	5.81	0.25	5.81	0.25
**33**		*i*-Pr	Ph	6.15	6.24	−0.09	5.73	0.42
**34**		*c*-Pr	*c*-Pr	5.32	5.41	−0.09	4.88	0.44
**35**		*n*-Bu	*n*-Bu	7.50 ^a^	6.50	1.00	6.98	0.52
**36**		*c*-Pen	*c*-Pen	7.95 ^a^	6.75	1.20	7.41	0.54
**37**		*c*-Hex	*c*-Hex	8.97 ^a^	7.37	1.60	8.58	0.39
**38**		*c*-Hex	H	7.34 ^a^	6.10	1.24	6.45	0.89
**39**		NMe_2_	NMe_2_	5.64	3.00	2.64	3.01	2.63

^a^ These Log*P* values are tentative because the hydrophobicity was beyond the range of the reference compounds (1.9 < Log*P* < 6.5).

**Table 2:** PR antagonistic activity assessed using a T47D alkaline phosphatase assay and the calculated molecular volumes of synthesized phosphine–borane derivatives **16**–**39**.

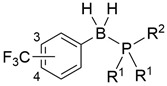						
Cmpd		R^1^	R^2^	Log*P*	Volume (Å^3^) ^a^	IC_50_ (μM) ^b^
**16**	3-CF_3_	Me	Me	3.65	238	>10
**17**		Me	Ph	4.54	304	1.16 ± 0.60
**18**		Et	Et	4.88	294	3.67 ± 3.34
**19**		Et	Ph	5.24	341	1.12 ± 0.22
**20**		*i*-Pr	*i*-Pr	5.89	348	1.35 ± 0.47
**21**		*i*-Pr	Ph	5.93	389	3.35 ± 1.49
**22**		*c*-Pr	*c*-Pr	5.21	328	0.65 ± 0.17
**23**		*n*-Bu	*n*-Bu	7.34	404	>10
**24**		*c*-Pen	*c*-Pen	7.84	424	6.64 ± 0.87
**25**		*c*-Hex	*c*-Hex	8.69	474	>10
**26**		*c*-Hex	H	7.28	376	N.D. ^c^
**27**		NMe_2_	NMe_2_	5.39	332	1.20 ± 0.63
**28**	4-CF_3_	Me	Me	3.65	238	>10
**29**		Me	Ph	4.65	304	1.61 ± 0.87
**30**		Et	Et	4.99	294	1.76 ± 1.44
**31**		Et	Ph	5.41	341	1.78 ± 0.97
**32**		*i*-Pr	*i*-Pr	6.06	347	1.51 ± 0.53
**33**		*i*-Pr	Ph	6.15	377	2.01 ± 0.58
**34**		*c*-Pr	*c*-Pr	5.32	328	0.54 ± 0.09
**35**		*n*-Bu	*n*-Bu	7.50	404	>10
**36**		*c*-Pen	*c*-Pen	7.95	424	2.63 ± 0.72
**37**		*c*-Hex	*c*-Hex	8.97	474	>10
**38**		*c*-Hex	H	7.34	376	N.D. ^c^
**39**		NMe_2_	NMe_2_	5.64	332	1.40 ± 0.88

^a^ These values were calculated for structures optimized at the B3LYP/6-311 + G** level using the Spartan’ 18 program (Wavefunction, Inc.). ^b^ The IC_50_ values are shown as the mean ± SD from three independent experiments. The P4 concentration was 1.0 nM. ^c^ Not determined.

## Data Availability

The data are contained within the article and Supplementary Materials.

## Supplementary Materials

The following supporting information can be downloaded at: https://www.mdpi.com/article/10.3390/molecules29071587/s1, ^1^H, ^13^C, ^11^B, ^19^F and ^31^P NMR Spectra of synthesized compounds.
