# Supplementary material for: Design, Synthesis, and Evaluation of B-(Trifluoromethyl)phenyl Phosphine–Borane Derivatives as Novel Progesterone Receptor Antagonists
Source: Molecules. 2024 Apr 2;29(7):1587. doi: 10.3390/molecules29071587 (PMC11013038; doi:10.3390/molecules29071587)
Supplement: Supplementary file 1 [file molecules-29-01587-s001.zip › molecules-2897421-supplementary.pdf]

Design, Synthesis, and Evaluation of *B*-(Trifluoromethyl)phenyl Phosphine–Borane Derivatives as Novel Progesterone Receptor Antagonists

Yu Miyajima, Kotaro Ochiai, Shinya Fujii

Supplementary Materials

1. Supplementary Figures
   - Figure S1
   - Table S1

2. ^{1}H, ^{13}C, ^{11}B, ^{19}F and ^{31}P NMR Spectra of synthesized compounds

Figure S1. T47D Alkaline phosphatase assay of test compounds alone. None of the synthesized compounds alone affected the alkaline phosphatase activity.

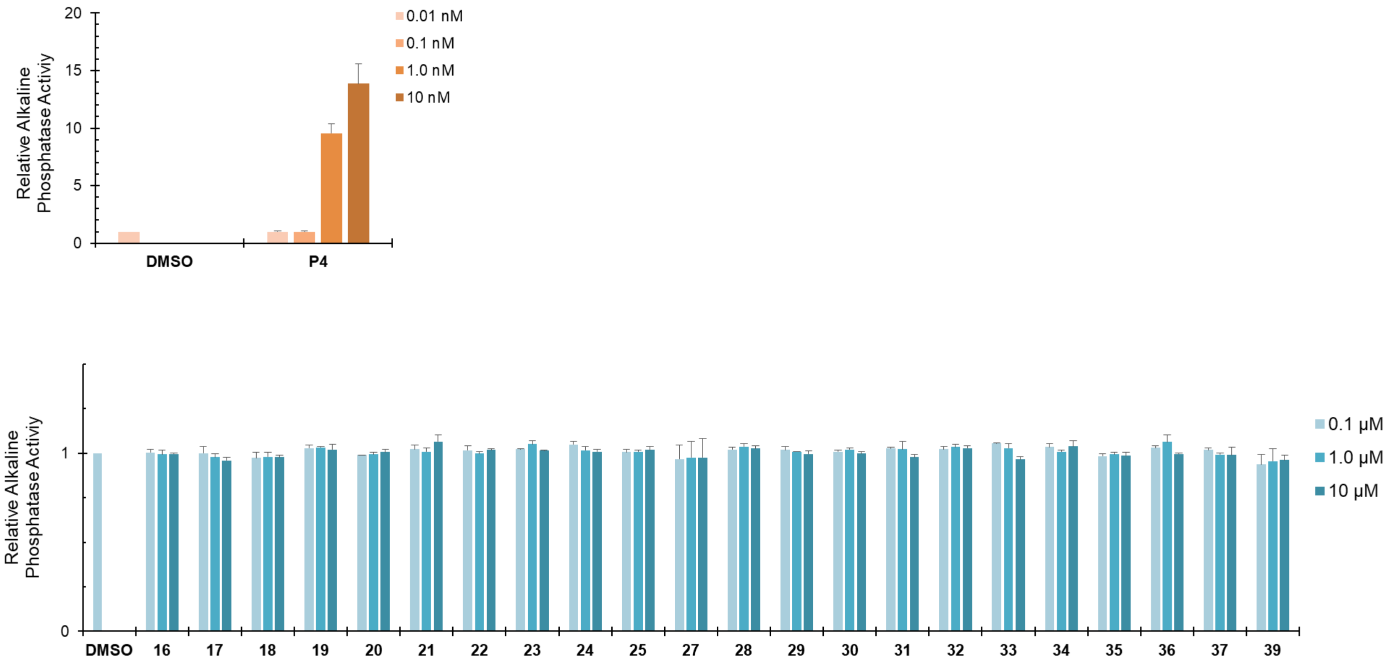

Table S1. Cell proliferation-promoting/inhibitory activity of compounds **28** and **34** toward androgen-dependent SC-3 cells. Both of the compounds did not exhibit cell proliferation promoting activity as well as inhibitory activity toward androgen-dependent cell proliferation.

Cmpd	EC_{50} (agonist) [a]	IC_{50} (antagonist) [b]
Dihydrotestosterone	0.12 nM	N.D.
Hydroxyflutamide	N.A.	0.37 μM
**28**	N.A.	N.A.
**34**	N.A.	N.A.

[a] Agonistic activity was evaluated by induction of cell-proliferation by single a treatment of the compound.
[b] Antagonistic activity was evaluated by inhibitory activity toward 1 nM-DHT promoted cell proliferation.
N.A. no activity in the concentration range of 0.01 μM to 10 μM.
N.D. not determined.

Procedure of SC-3 assay. SC-3 cells were cultured in MEM*α* (Fujifilm Wako Co.) supplemented with 2 % FBS and 1 nM dihydrotestosterone (DHT) at 37 °C under 5 % CO_2. All experiments were per-formed in triplicate or more. Cells were trypsinized and diluted to 20,000 cells/mL with DMEM supplemented with 2 % charcoal-stripped FBS. This cell suspension was seeded in 96-well plates at a volume of 90 μL per well and incubated at 24 h. Then, 10 μL of the drug solution, which was supplemented with serial dilutions of the test compounds or DMSO as a dilution control in the presence of 1 nM DHT, was added. The plates were incubated at 37 °C under 5 % CO_2 for 3 days, and the cell number was determined using a Cell Counting Kit-8 (DOJINDO). A 10 μL aliquot of WST-8 was added to each well, and the cells were further incubated for 2 h, then the absorbance at 450 nm was determined with a microplate reader as a measure of the number of living cells in the culture.

3CF3PhBH2PMe3

B-3-(Trifluoromethyl)phenyl trimethylphosphine–borane (16)

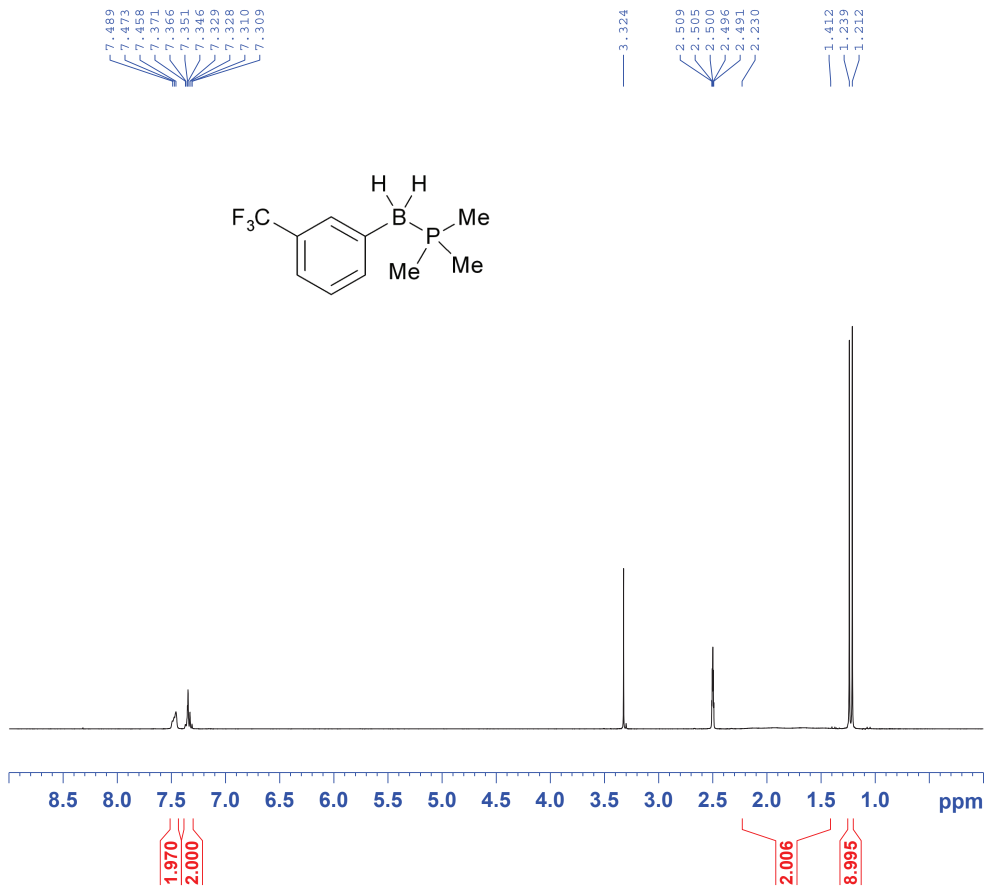

IBB-nmr Analysis

NAME	Mar12-2022
EXPNO	1
PROCNO	1
Date_	20220312
Time	21.51
INSTRUM	av400
PROBHD	5 mm PABBO BB-
PULPROG	zg30
TD	65536
SOLVENT	DMSO
NS	16
DS	2
SWH	8223.685 Hz
FIDRES	0.125483 Hz
AQ	3.9846387 sec
RG	203
DW	60.800 usec
DE	6.50 usec
TE	296.7 K
D1	1.00000000 sec
TD0	1
======== CHANNEL f1 ========	
NUC1	1H
P1	14.00 usec
PL1	-1.80 dB
PL1W	14.82738590 W
SFO1	400.1324710 MHz
SI	32768
SF	400.1300034 MHz
WDW	EM
SSB	0
LB	0.30 Hz
GB	0
PC	1.00

3CF3PhBH2PMe3

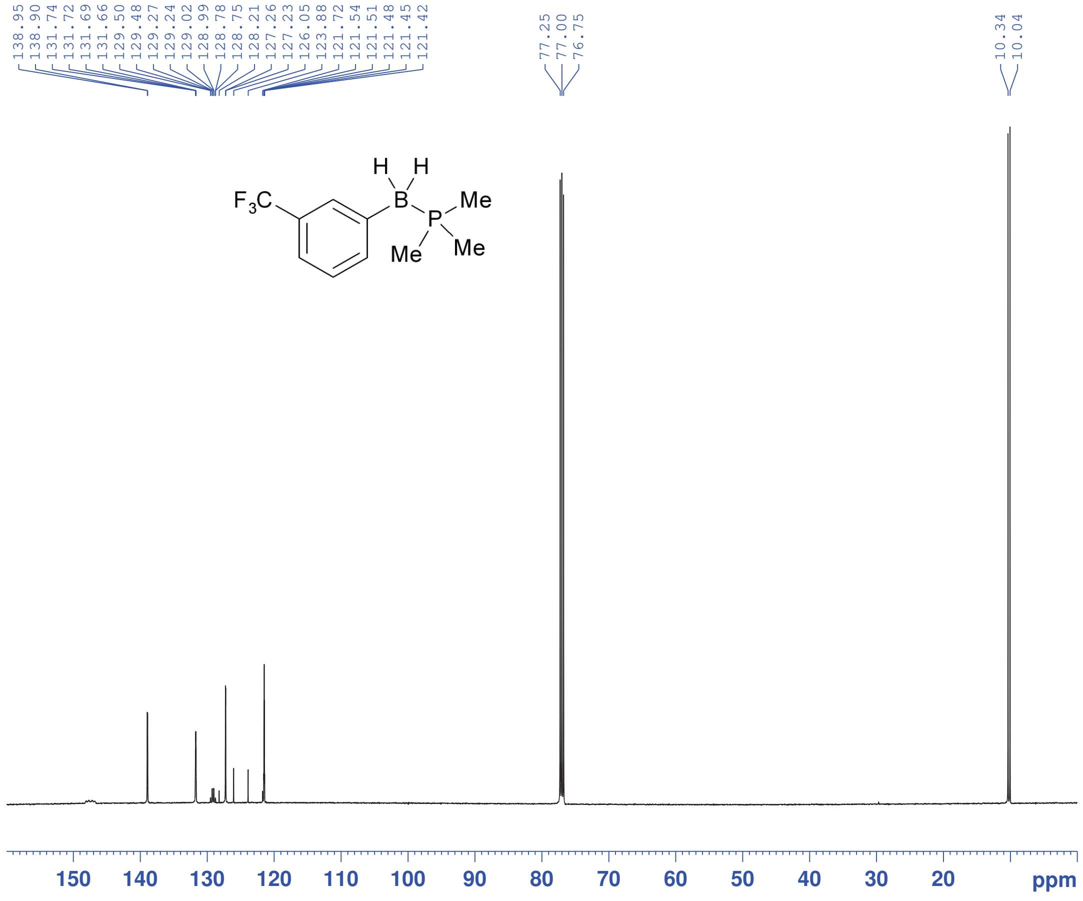

IBB-nmr Analysis

NAME	Mar07-2024
EXPNO	5
PROCNO	1
Date_	20240307
Time	23.44
INSTRUM	av500
PROBHD	5 mm CPDCH 13C
PULPROG	zgpg30
TD	65536
SOLVENT	CDCl3
NS	256
DS	4
SWH	30030.029 Hz
FIDRES	0.458222 Hz
AQ	1.0912410 sec
RG	35.9
DW	16.650 usec
DE	20.00 usec
TE	298.1 K
D1	2.00000000 sec
D11	0.03000000 sec
TD0	1
======== CHANNEL f1 ========	
NUC1	13C
P1	10.00 usec
PL1	-5.00 dB
PL1W	14.29790783 W
SFO1	125.7703643 MHz
======== CHANNEL f2 ========	
CPDPRG2	waltz16
NUC2	1H
PCPD2	80.00 usec
PL2	-4.00 dB
PL12	14.06 dB
PL13	17.00 dB
PL2W	6.30957365 W
PL12W	0.09862794 W
PL13W	0.05011872 W
SFO2	500.1320005 MHz
SI	32768
SF	125.7577946 MHz
WDW	EM
SSB	0
LB	1.00 Hz
GB	0
PC	0.30

3CF3PhBH2PMe3

IBB-nmr Analysis

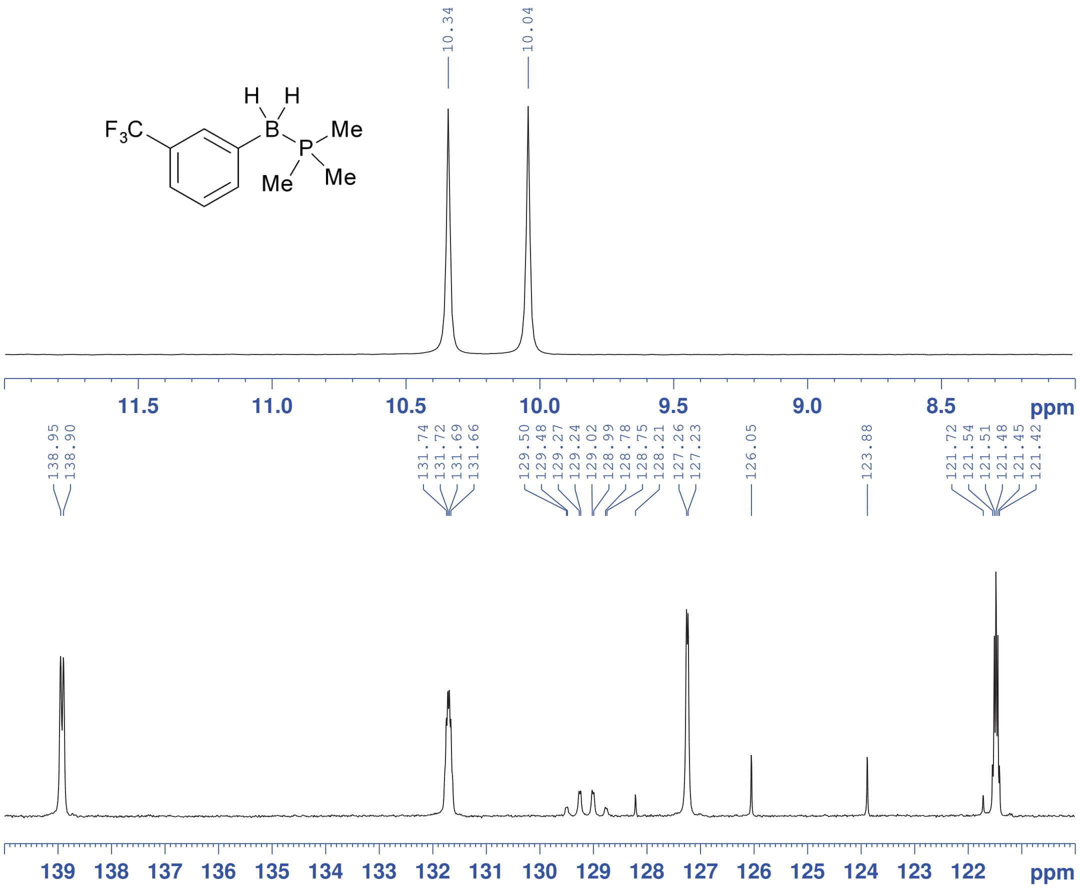

NAME	Mar07-2024	
EXPNO	5	
PROCNO	1	
Date_	20240307	
Time	23.44	
INSTRUM	av500	
PROBHD	5 mm CPDCH 13C	
PULPROG	zgpg30	
TD	65536	
SOLVENT	CDCl3	
NS	256	
DS	4	
SWH	30030.029	Hz
FIDRES	0.458222	Hz
AQ	1.0912410	sec
RG	35.9	
DW	16.650	usec
DE	20.00	usec
TE	298.1	K
D1	2.00000000	sec
D11	0.03000000	sec
TD0	1	

======== CHANNEL f1 ========

NUC1	13C	
P1	10.00	usec
PL1	-5.00	dB
PL1W	14.29790783	W
SFO1	125.7703643	MHz

======== CHANNEL f2 ========

CPDPRG2	waltz16	
NUC2	1H	
PCPD2	80.00	usec
PL2	-4.00	dB
PL12	14.06	dB
PL13	17.00	dB
PL2W	6.30957365	W
PL12W	0.09862794	W
PL13W	0.05011872	W
SFO2	500.1320005	MHz
SI	32768	
SF	125.7577946	MHz
WDW	EM	
SSB	0	
LB	1.00	Hz
GB	0	
PC	0.30	

3CF3PhBH2PMe3

IBB-nmr Analysis

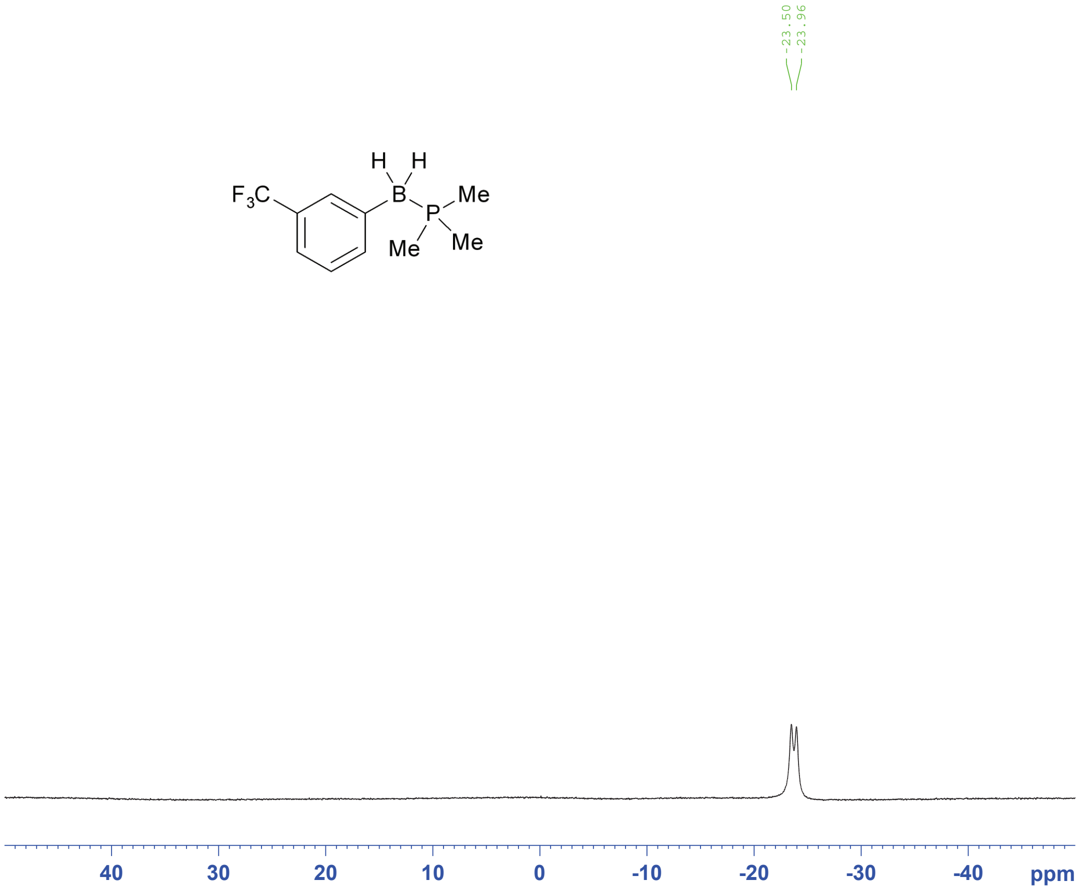

NAME	Mar12-2022	
EXPNO	5	
PROCNO	1	
Date_	20220312	
Time	22.06	
INSTRUM	av400	
PROBHD	5 mm PABBO BB-	
PULPROG	zgig	
TD	32768	
SOLVENT	DMSO	
NS	128	
DS	4	
SWH	20000.000	Hz
FIDRES	0.610352	Hz
AQ	0.8192500	sec
RG	203	
DW	25.000	usec
DE	20.00	usec
TE	297.2	K
D1	1.00000000	sec
D11	0.03000000	sec
TD0	1	

======== CHANNEL f1 ========

NUC1	11B	
P1	6.30	usec
PL1	-2.00	dB
SFO1	128.3776050	MHz

======== CHANNEL f2 ========

CPDPRG2	waltz16	
NUC2	1H	
PCPD2	80.00	usec
PL2	-1.70	dB
PL12	13.80	dB
PL2W	14.48987389	W
PL12W	0.40838012	W
SFO2	400.1316005	MHz
SI	32768	
SF	128.3776183	MHz
WDW	EM	
SSB	0	
LB	2.00	Hz
GB	0	
PC	1.40	

3CF3PhBH2PMe3

IBB-nmr Analysis

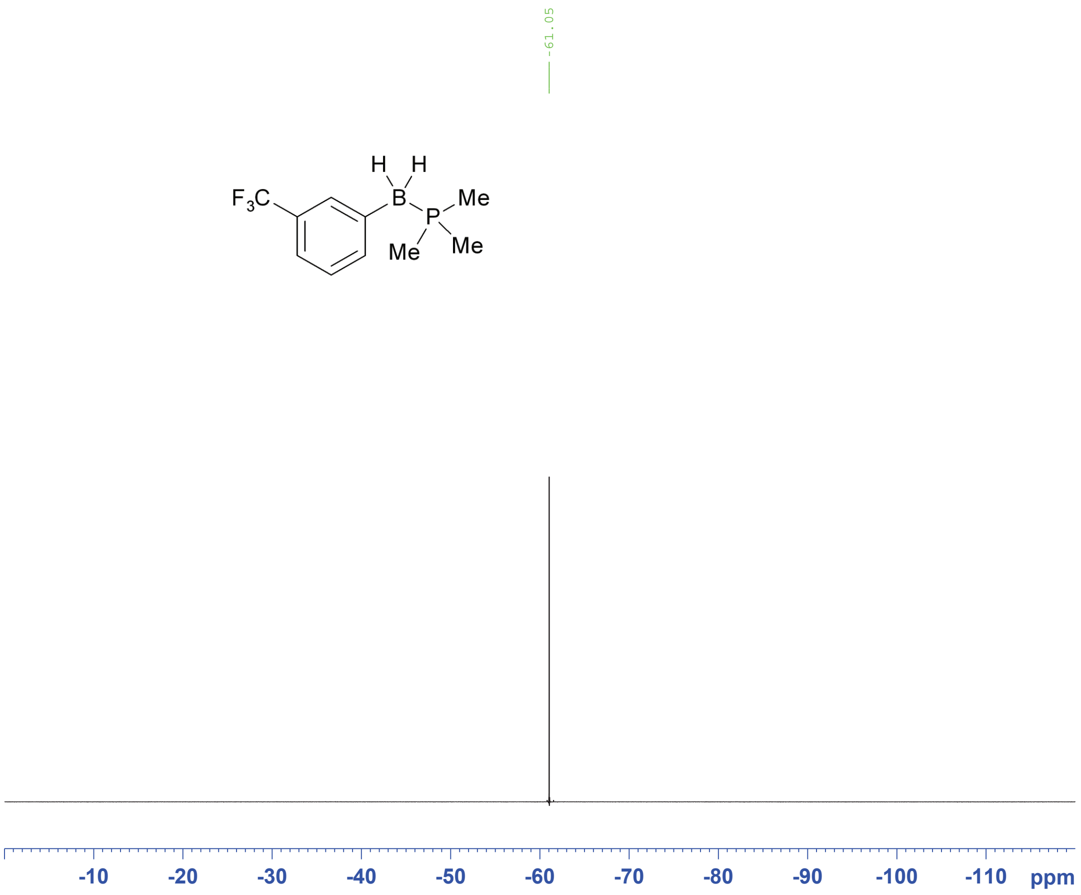

NAME	Mar12-2022
EXPNO	2
PROCNO	1
Date_	20220312
Time	21.55
INSTRUM	av400
PROBHD	5 mm PABBO BB-
PULPROG	zgfhigqn
TD	131072
SOLVENT	DMSO
NS	8
DS	4
SWH	89285.711 Hz
FIDRES	0.681196 Hz
AQ	0.7340532 sec
RG	203
DW	5.600 usec
DE	6.50 usec
TE	296.7 K
D1	1.00000000 sec
D11	0.03000000 sec
D12	0.00002000 sec
TD0	1

======== CHANNEL f1 ========

NUC1	19F
P1	19.50 usec
PL1	-4.00 dB
PL1W	16.40489006 W
SFO1	376.4607164 MHz

======== CHANNEL f2 ========

CPDPRG2	waltz16
NUC2	1H
PCPD2	112.00 usec
PL2	-4.00 dB
PL12	18.00 dB
PL2W	24.60733604 W
PL12W	0.15526180 W
SFO2	400.1316005 MHz
SI	65536
SF	376.4984617 MHz
WDW	EM
SSB	0
LB	0.30 Hz
GB	0
PC	1.00

3CF3PhBH2PMe3

IBB-nmr Analysis

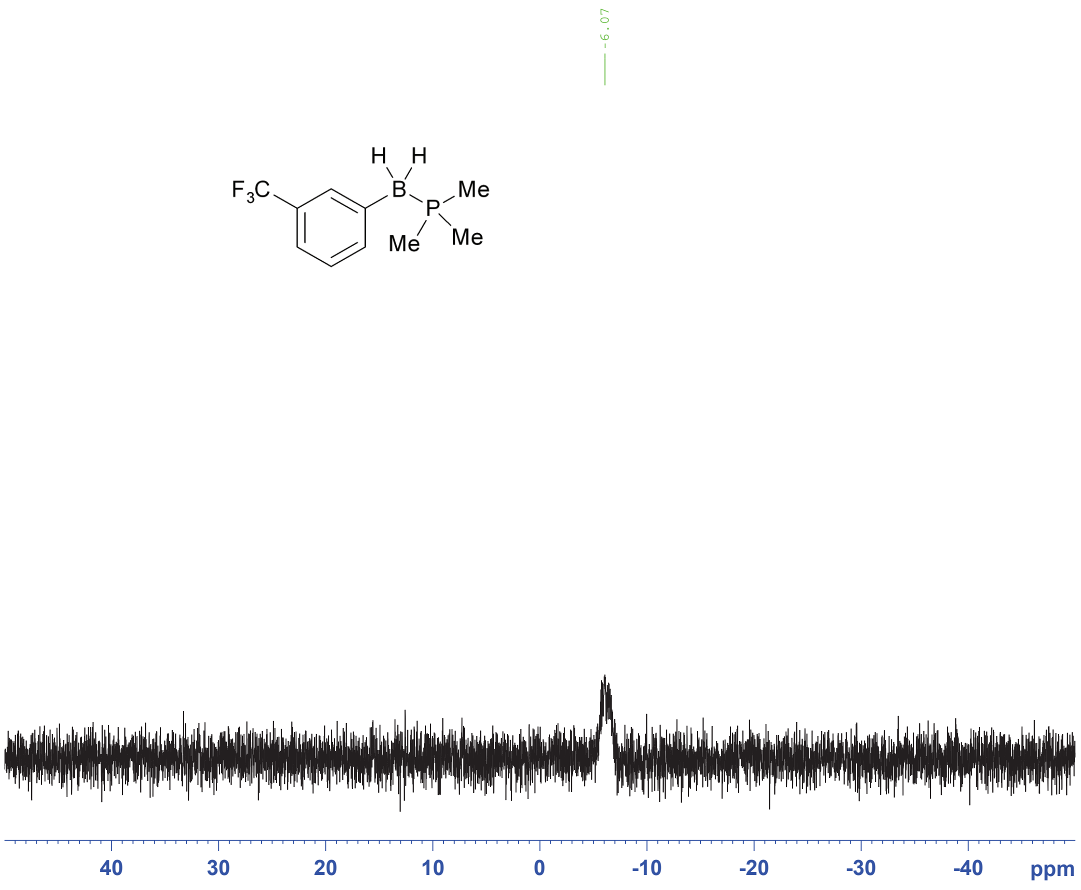

NAME	Mar12-2022
EXPNO	4
PROCNO	1
Date_	20220312
Time	21.58
INSTRUM	av400
PROBHD	5 mm PABBO BB-
PULPROG	zg30
TD	65536
SOLVENT	DMSO
NS	8
DS	4
SWH	64102.563 Hz
FIDRES	0.978127 Hz
AQ	0.5112308 sec
RG	203
DW	7.800 usec
DE	6.50 usec
TE	296.5 K
D1	2.00000000 sec
TD0	1

======== CHANNEL f1 ========

NUC1	31P
P1	10.00 usec
PL1	-0.70 dB
PL1W	30.10233498 W
SFO1	161.9674942 MHz
SI	32768
SF	161.9754962 MHz
WDW	EM
SSB	0
LB	1.00 Hz
GB	0
PC	1.40

3CF3PhBH2PPhMe2 21-60Fr

B-(3-Trifluoromethyl)phenyl dimethyl(phenyl)phosphine–borane (17)

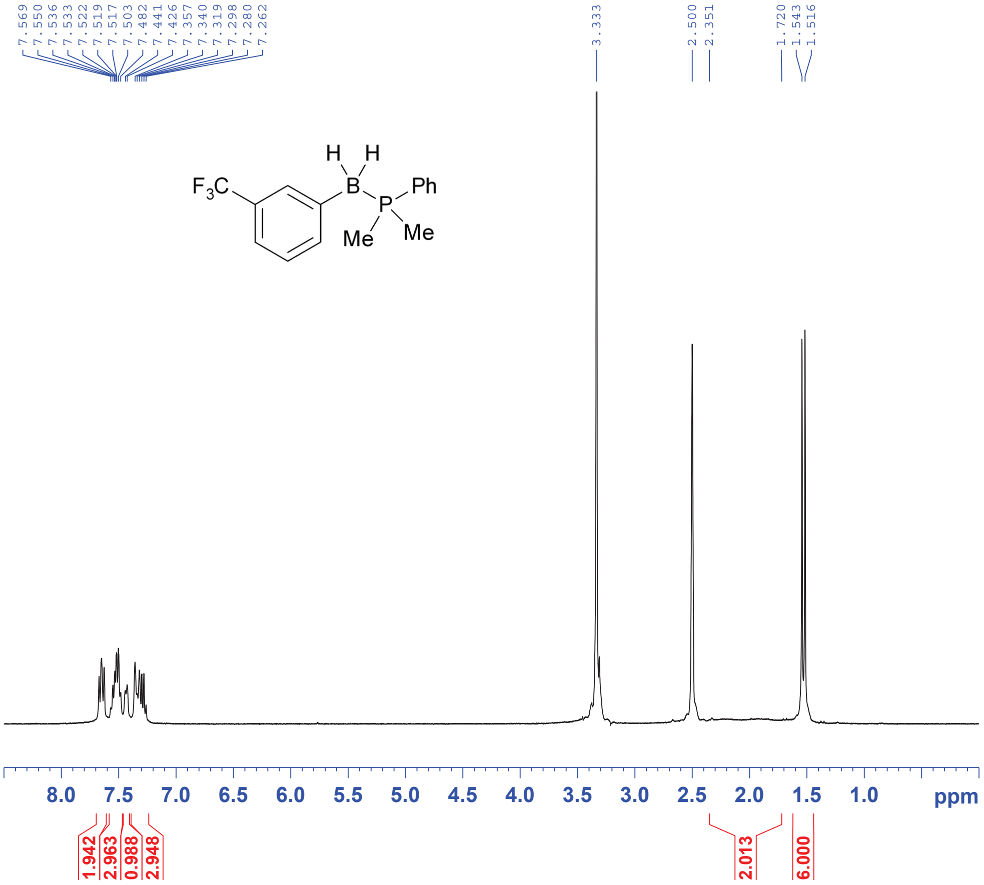

IBB-nmr Analysis

NAME	Apr14-2022
EXPNO	1
PROCNO	1
Date_	20220414
Time	21.36
INSTRUM	av400
PROBHD	5 mm PABBO BB-
PULPROG	zg30
TD	65536
SOLVENT	DMSO
NS	16
DS	2
SWH	8223.685 Hz
FIDRES	0.125483 Hz
AQ	3.9846387 sec
RG	203
DW	60.800 usec
DE	6.50 usec
TE	295.6 K
D1	1.00000000 sec
TD0	1
======== CHANNEL f1 ========	
NUC1	1H
P1	14.00 usec
PL1	-1.80 dB
PL1W	14.82738590 W
SFO1	400.1324710 MHz
SI	32768
SF	400.1300038 MHz
WDW	EM
SSB	0
LB	0.30 Hz
GB	0
PC	1.00

3CF3PhBH2PMe2Ph

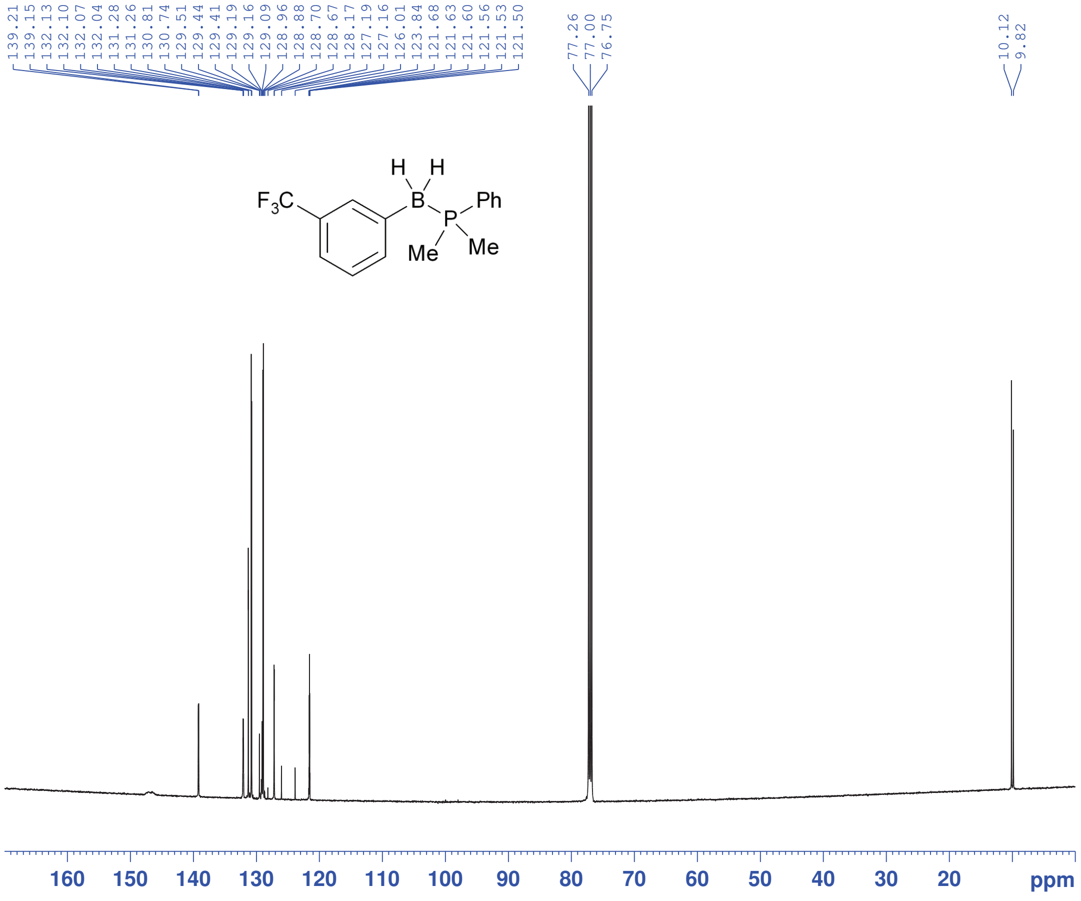

IBB-nmr Analysis

NAME	Mar08-2024
EXPNO	7
PROCNO	1
Date_	20240308
Time	3.33
INSTRUM	av500
PROBHD	5 mm CPDCH 13C
PULPROG	zgpg30
TD	65536
SOLVENT	CDCl3
NS	1024
DS	4
SWH	30030.029 Hz
FIDRES	0.458222 Hz
AQ	1.0912410 sec
RG	35.9
DW	16.650 usec
DE	20.00 usec
TE	298.1 K
D1	2.00000000 sec
D11	0.03000000 sec
TD0	1
======== CHANNEL f1 ========	
NUC1	13C
P1	10.00 usec
PL1	-5.00 dB
PL1W	14.29790783 W
SFO1	125.7703643 MHz
======== CHANNEL f2 ========	
CPDPRG2	waltz16
NUC2	1H
PCPD2	80.00 usec
PL2	-4.00 dB
PL12	14.06 dB
PL13	17.00 dB
PL2W	6.30957365 W
PL12W	0.09862794 W
PL13W	0.05011872 W
SFO2	500.1320005 MHz
SI	32768
SF	125.7577928 MHz
WDW	EM
SSB	0
LB	1.00 Hz
GB	0
PC	0.30

3CF3PhBH2PMe2Ph

IBB-nmr Analysis

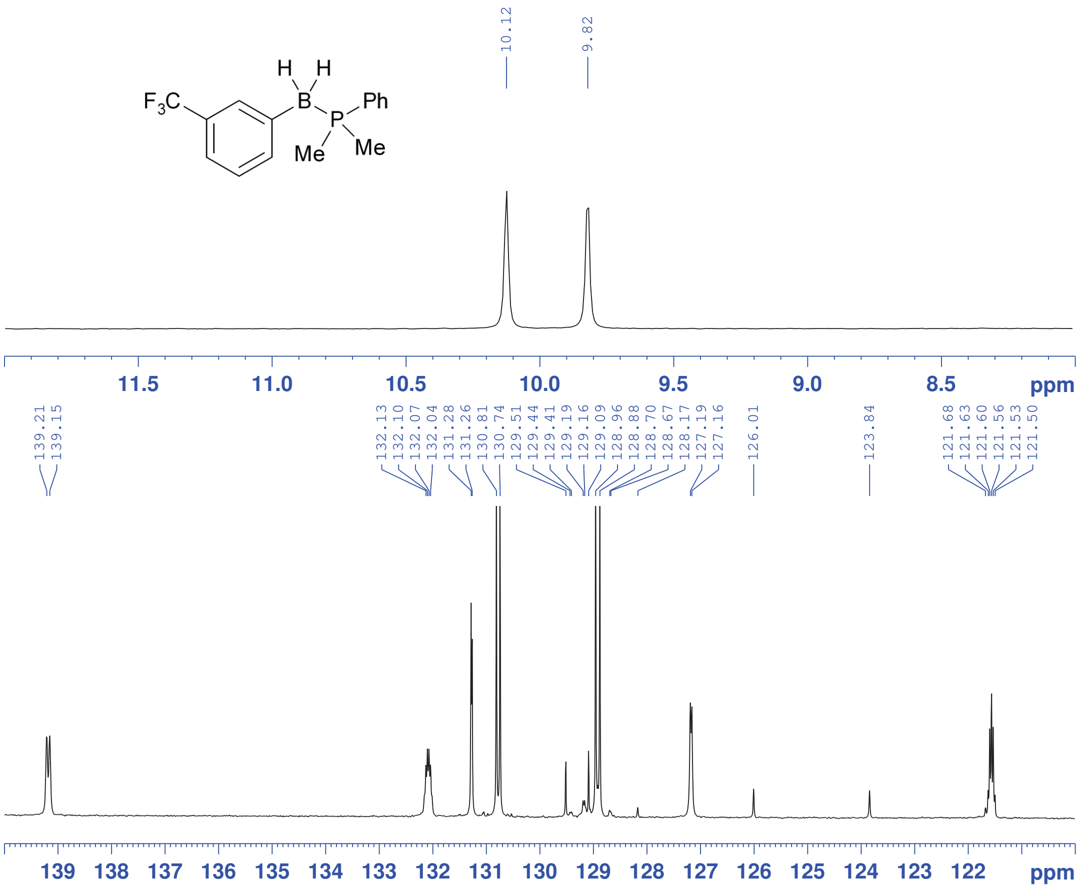

NAME	Mar08-2024
EXPNO	7
PROCNO	1
Date_	20240308
Time	3.33
INSTRUM	av500
PROBHD	5 mm CPDCH 13C
PULPROG	zgpg30
TD	65536
SOLVENT	CDCl3
NS	1024
DS	4
SWH	30030.029 Hz
FIDRES	0.458222 Hz
AQ	1.0912410 sec
RG	35.9
DW	16.650 usec
DE	20.00 usec
TE	298.1 K
D1	2.00000000 sec
D11	0.03000000 sec
TD0	1

======== CHANNEL f1 ========

NUC1	13C
P1	10.00 usec
PL1	-5.00 dB
PL1W	14.29790783 W
SFO1	125.7703643 MHz

======== CHANNEL f2 ========

CPDPRG2	waltz16
NUC2	1H
PCPD2	80.00 usec
PL2	-4.00 dB
PL12	14.06 dB
PL13	17.00 dB
PL2W	6.30957365 W
PL12W	0.09862794 W
PL13W	0.05011872 W
SFO2	500.1320005 MHz
SI	32768
SF	125.7577928 MHz
WDW	EM
SSB	0
LB	1.00 Hz
GB	0
PC	0.30

3HOPhBH2PMe2Ph

IBB-nmr Analysis

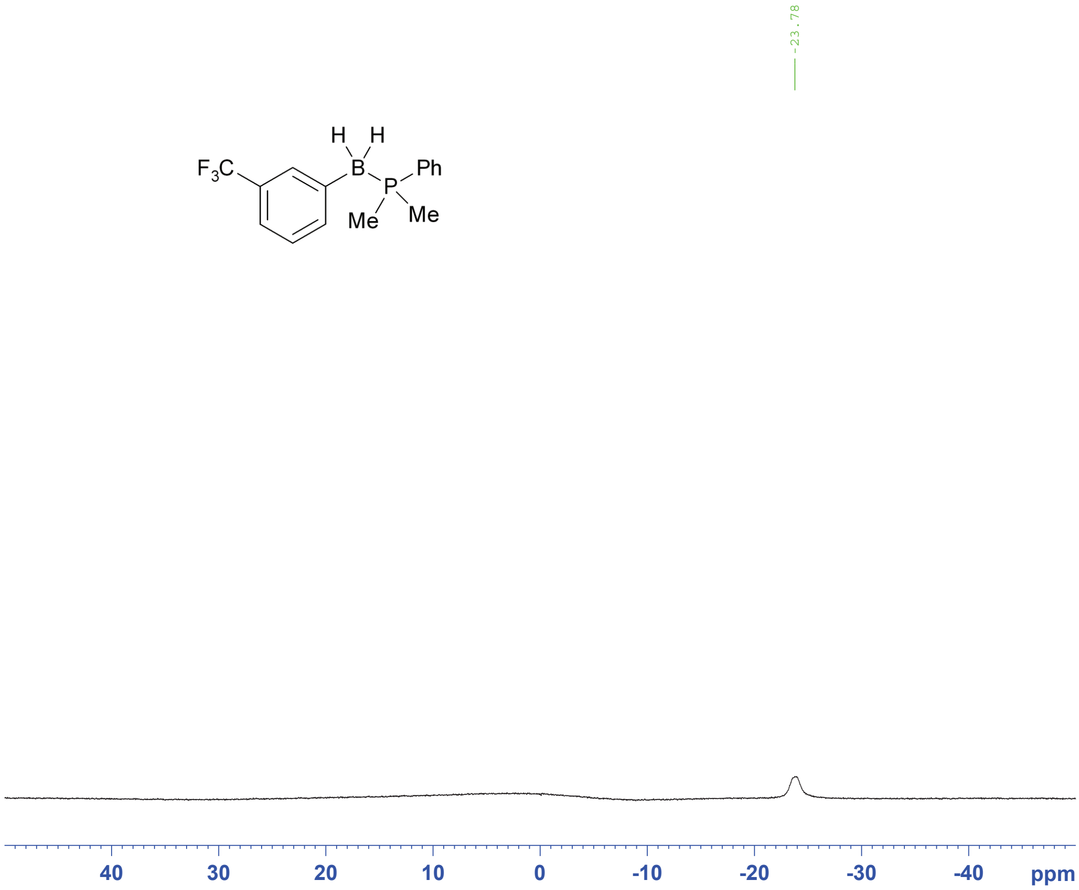

NAME	Jul30-2023
EXPNO	6
PROCNO	1
Date_	20230730
Time	13.35
INSTRUM	av400
PROBHD	5 mm PABBO BB-
PULPROG	zgig
TD	32768
SOLVENT	DMSO
NS	128
DS	4
SWH	20000.000 Hz
FIDRES	0.610352 Hz
AQ	0.8192500 sec
RG	203
DW	25.000 usec
DE	20.00 usec
TE	297.3 K
D1	1.00000000 sec
D11	0.03000000 sec
TD0	1

======== CHANNEL f1 ========

NUC1	11B
P1	6.30 usec
PL1	-2.00 dB
SFO1	128.3776050 MHz

======== CHANNEL f2 ========

CPDPRG2	waltz16
NUC2	1H
PCPD2	80.00 usec
PL2	-1.70 dB
PL12	13.80 dB
PL2W	14.48987389 W
PL12W	0.40838012 W
SFO2	400.1316005 MHz
SI	32768
SF	128.3776183 MHz
WDW	EM
SSB	0
LB	2.00 Hz
GB	0
PC	1.40

3CF3PhBH2PPhMe2

IBB-nmr Analysis

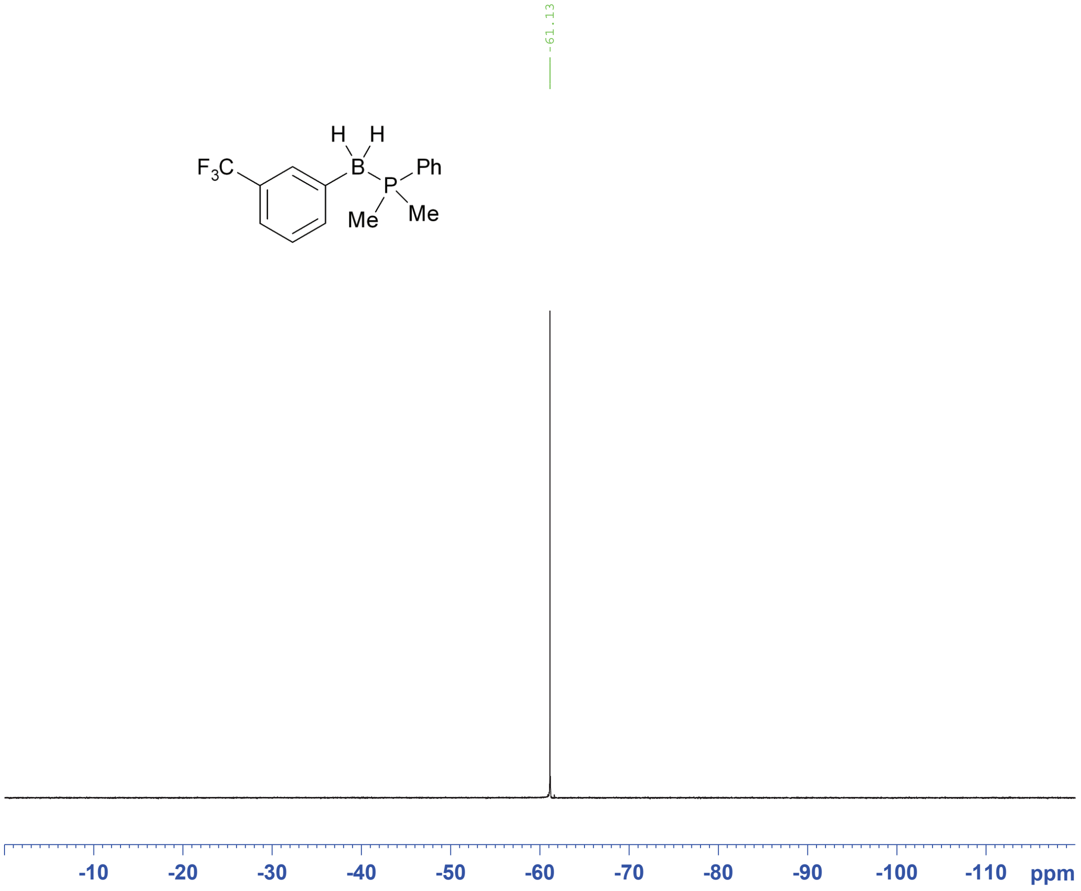


```
NAME            Apr14-2022
EXPNO                    3
PROCNO                   1
Date_             20220414
Time                 21.45
INSTRUM              av400
PROBHD   5 mm PABBO BB-
PULPROG           zgfhigqn
TD                  131072
SOLVENT               DMSO
NS                       8
DS                       4
SWH              89285.711 Hz
FIDRES            0.681196 Hz
AQ               0.7340532 sec
RG                     203
DW                   5.600 usec
DE                    6.50 usec
TE                   295.4 K
D1              1.00000000 sec
D11             0.03000000 sec
D12             0.00002000 sec
TD0                      1

======== CHANNEL f1 ========
NUC1                   19F
P1                   19.50 usec
PL1                  -4.00 dB
PL1W           16.40489006 W
SFO1           376.4607164 MHz

======== CHANNEL f2 ========
CPDPRG2            waltz16
NUC2                    1H
PCPD2               112.00 usec
PL2                  -4.00 dB
PL12                 18.00 dB
PL2W           24.60733604 W
PL12W           0.15526180 W
SFO2           400.1316005 MHz
SI                   65536
SF             376.4984617 MHz
WDW                     EM
SSB                      0
LB                    0.30 Hz
GB                       0
PC                    1.00
```

3CF3PhBH2PPhMe2 21-60Fr

IBB-nmr Analysis

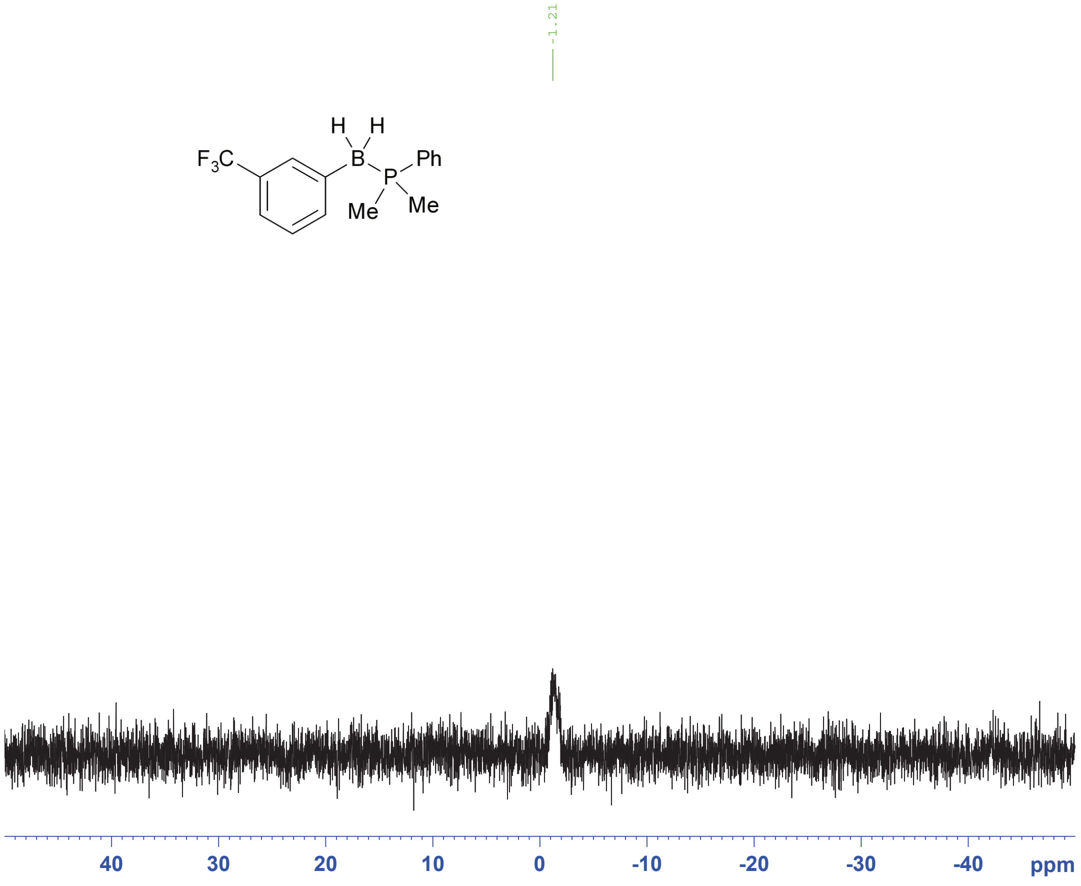


```
NAME            Apr14-2022
EXPNO                    2
PROCNO                   1
Date_             20220414
Time                 21.41
INSTRUM              av400
PROBHD   5 mm PABBO BB-
PULPROG               zg30
TD                   65536
SOLVENT               DMSO
NS                      16
DS                       4
SWH              64102.563 Hz
FIDRES            0.978127 Hz
AQ               0.5112308 sec
RG                     203
DW                   7.800 usec
DE                    6.50 usec
TE                   295.5 K
D1              2.00000000 sec
TD0                      1

======== CHANNEL f1 ========
NUC1                   31P
P1                   10.00 usec
PL1                  -0.70 dB
PL1W           30.10233498 W
SFO1           161.9674942 MHz
SI                   32768
SF             161.9754962 MHz
WDW                     EM
SSB                      0
LB                    1.00 Hz
GB                       0
PC                    1.40
```

3CF3PhBH2PEt3

B-3-(Trifluoromethyl)phenyl triethylphosphine–borane (18)

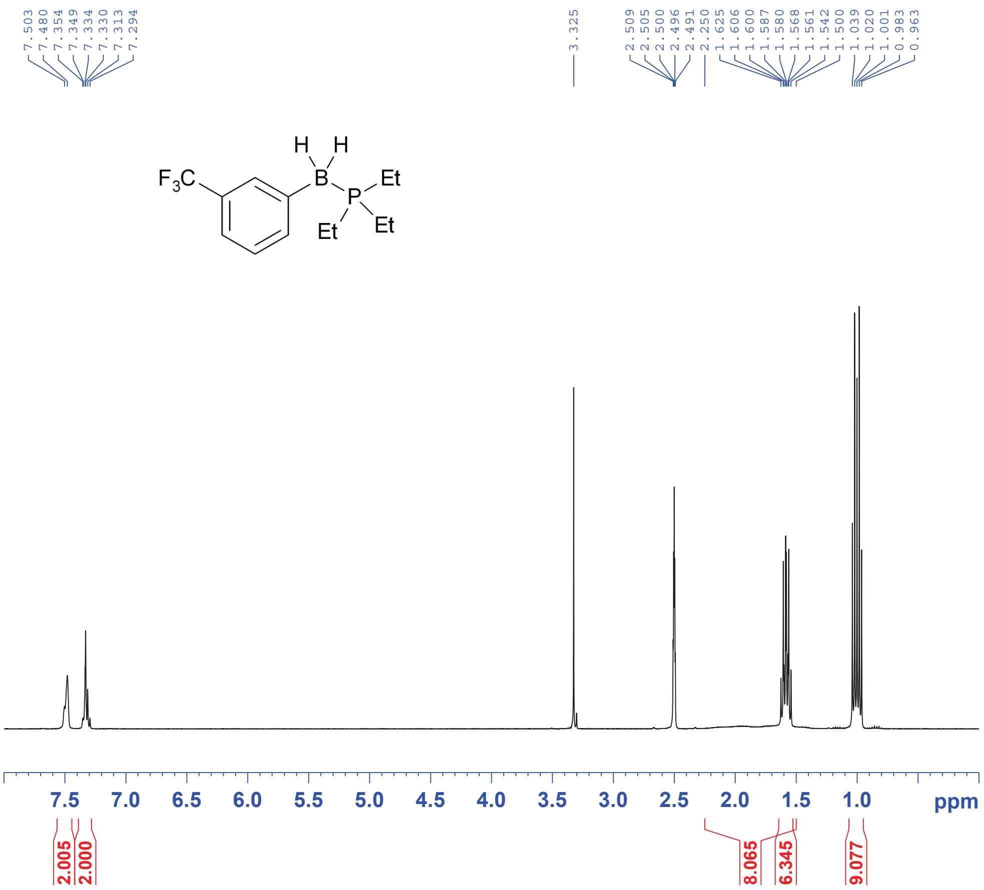

IBB-nmr Analysis

NAME Jan16-2022
EXPNO 4
PROCNO 1
Date_ 20220116
Time 23.53
INSTRUM av400
PROBHD 5 mm PABBO BB-
PULPROG zg30
TD 65536
SOLVENT DMSO
NS 16
DS 2
SWH 8223.685 Hz
FIDRES 0.125483 Hz
AQ 3.9846387 sec
RG 203
DW 60.800 usec
DE 6.50 usec
TE 296.2 K
D1 1.00000000 sec
TD0 1

======== CHANNEL f1 ========
NUC1 1H
P1 14.00 usec
PL1 -1.80 dB
PL1W 14.82738590 W
SFO1 400.1324710 MHz
SI 32768
SF 400.1300034 MHz
WDW EM
SSB 0
LB 0.30 Hz
GB 0
PC 1.00

3CF3PhBH2PEt3

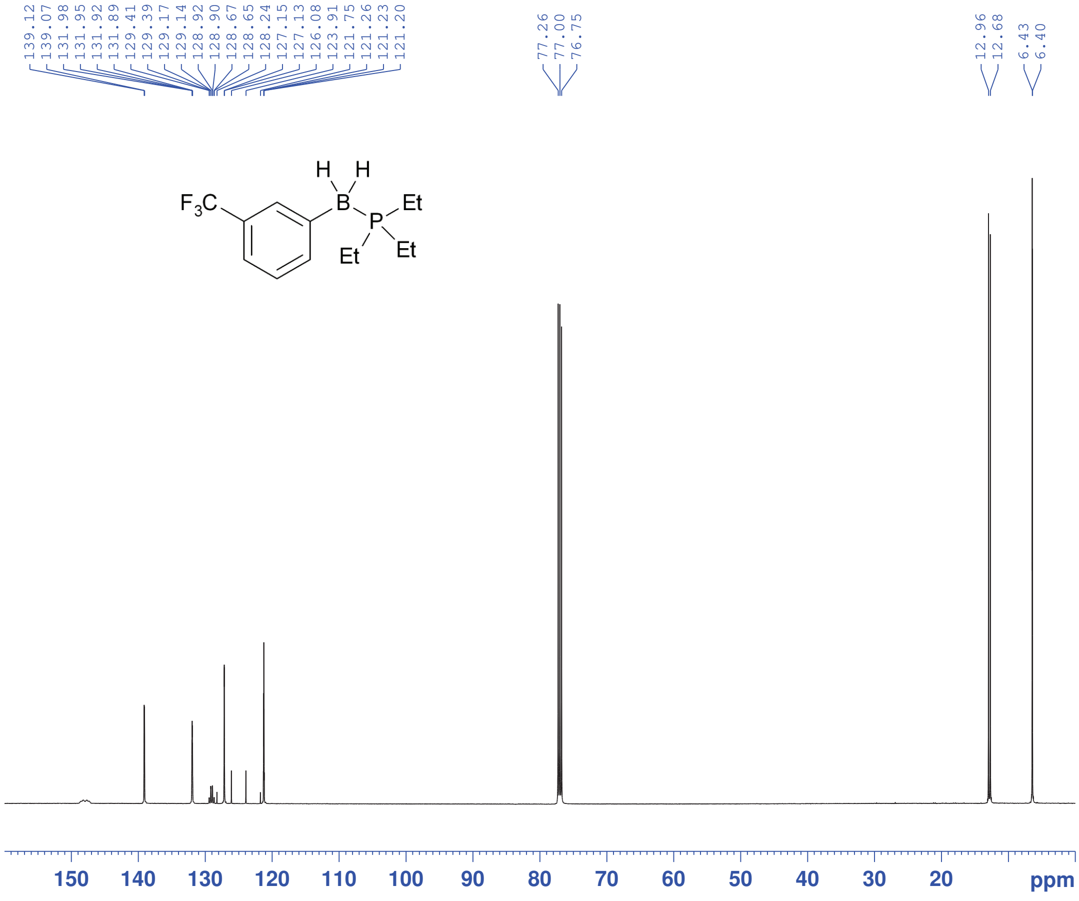

IBB-nmr Analysis

NAME Mar08-2024
EXPNO 15
PROCNO 1
Date_ 20240308
Time 8.30
INSTRUM av500
PROBHD 5 mm CPDCH 13C
PULPROG zgpg30
TD 65536
SOLVENT CDCl3
NS 1024
DS 4
SWH 30030.029 Hz
FIDRES 0.458222 Hz
AQ 1.0912410 sec
RG 90.5
DW 16.650 usec
DE 20.00 usec
TE 298.0 K
D1 2.00000000 sec
D11 0.03000000 sec
TD0 1

======== CHANNEL f1 ========
NUC1 13C
P1 10.00 usec
PL1 -5.00 dB
PL1W 14.29790783 W
SFO1 125.7703643 MHz

======== CHANNEL f2 ========
CPDPRG2 waltz16
NUC2 1H
PCPD2 80.00 usec
PL2 -4.00 dB
PL12 14.06 dB
PL13 17.00 dB
PL2W 6.30957365 W
PL12W 0.09862794 W
PL13W 0.05011872 W
SFO2 500.1320005 MHz
SI 32768
SF 125.7577946 MHz
WDW EM
SSB 0
LB 1.00 Hz
GB 0
PC 0.30

3CF3PhBH2PEt3

IBB-nmr Analysis

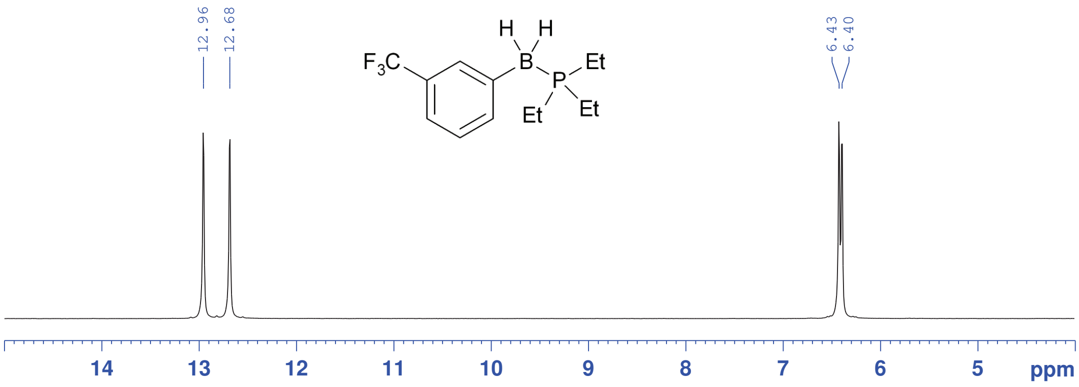

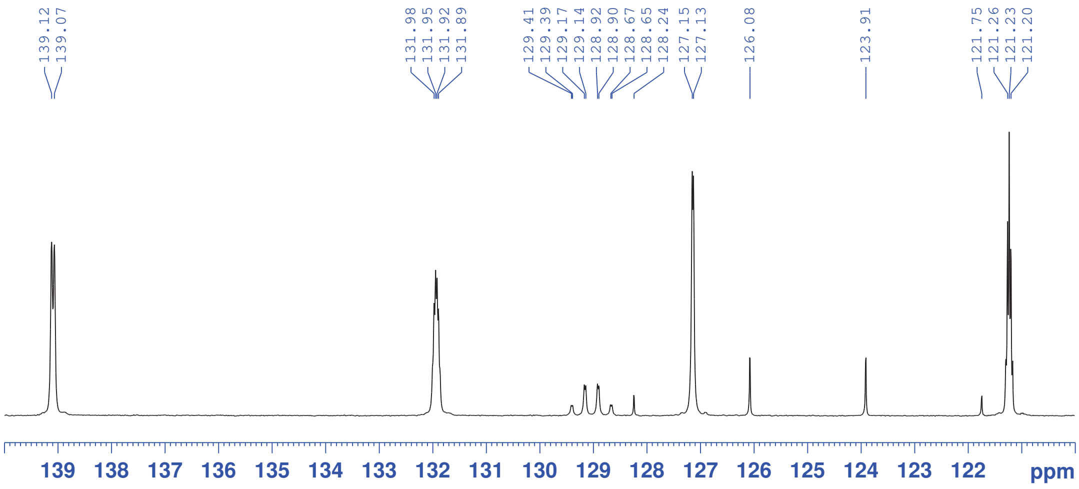


```
NAME           Mar08-2024
EXPNO                  15
PROCNO                  1
Date_            20240308
Time                 8.30
INSTRUM             av500
PROBHD   5 mm CPDCH 13C
PULPROG            zgpg30
TD                  65536
SOLVENT             CDCl3
NS                   1024
DS                      4
SWH             30030.029 Hz
FIDRES           0.458222 Hz
AQ              1.0912410 sec
RG                   90.5
DW                 16.650 usec
DE                  20.00 usec
TE                  298.0 K
D1             2.00000000 sec
D11            0.03000000 sec
TD0                     1

======== CHANNEL f1 ========
NUC1                  13C
P1                  10.00 usec
PL1                 -5.00 dB
PL1W          14.29790783 W
SFO1          125.7703643 MHz

======== CHANNEL f2 ========
CPDPRG2           waltz16
NUC2                   1H
PCPD2               80.00 usec
PL2                 -4.00 dB
PL12                14.06 dB
PL13                17.00 dB
PL2W           6.30957365 W
PL12W          0.09862794 W
PL13W          0.05011872 W
SFO2          500.1320005 MHz
SI                  32768
SF            125.7577946 MHz
WDW                    EM
SSB                     0
LB                   1.00 Hz
GB                      0
PC                   0.30
```

3CF3PhBH2PEt3

IBB-nmr Analysis

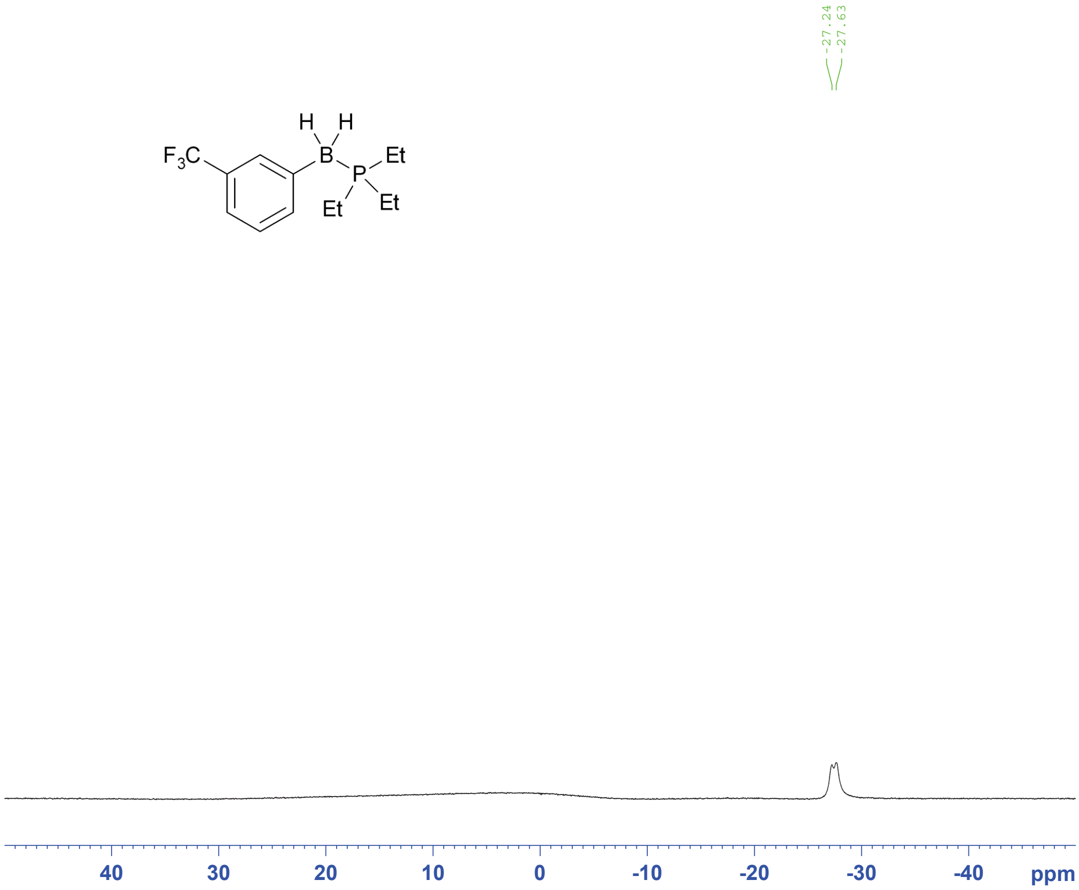


```
NAME           Jan16-2022
EXPNO                   5
PROCNO                  1
Date_            20220117
Time                 0.00
INSTRUM             av400
PROBHD   5 mm PABBO BB-
PULPROG              zgig
TD                  32768
SOLVENT              DMSO
NS                    128
DS                      4
SWH             20000.000 Hz
FIDRES           0.610352 Hz
AQ              0.8192500 sec
RG                    203
DW                 25.000 usec
DE                  20.00 usec
TE                  296.9 K
D1             1.00000000 sec
D11            0.03000000 sec
TD0                     1

======== CHANNEL f1 ========
NUC1                  11B
P1                   6.30 usec
PL1                 -2.00 dB
SFO1          128.3776050 MHz

======== CHANNEL f2 ========
CPDPRG2           waltz16
NUC2                   1H
PCPD2               80.00 usec
PL2                 -1.70 dB
PL12                13.80 dB
PL2W          14.48987389 W
PL12W          0.40838012 W
SFO2          400.1316005 MHz
SI                  32768
SF            128.3776183 MHz
WDW                    EM
SSB                     0
LB                   2.00 Hz
GB                      0
PC                   1.40
```

3CF3PhBH2PEt3

IBB-nmr Analysis

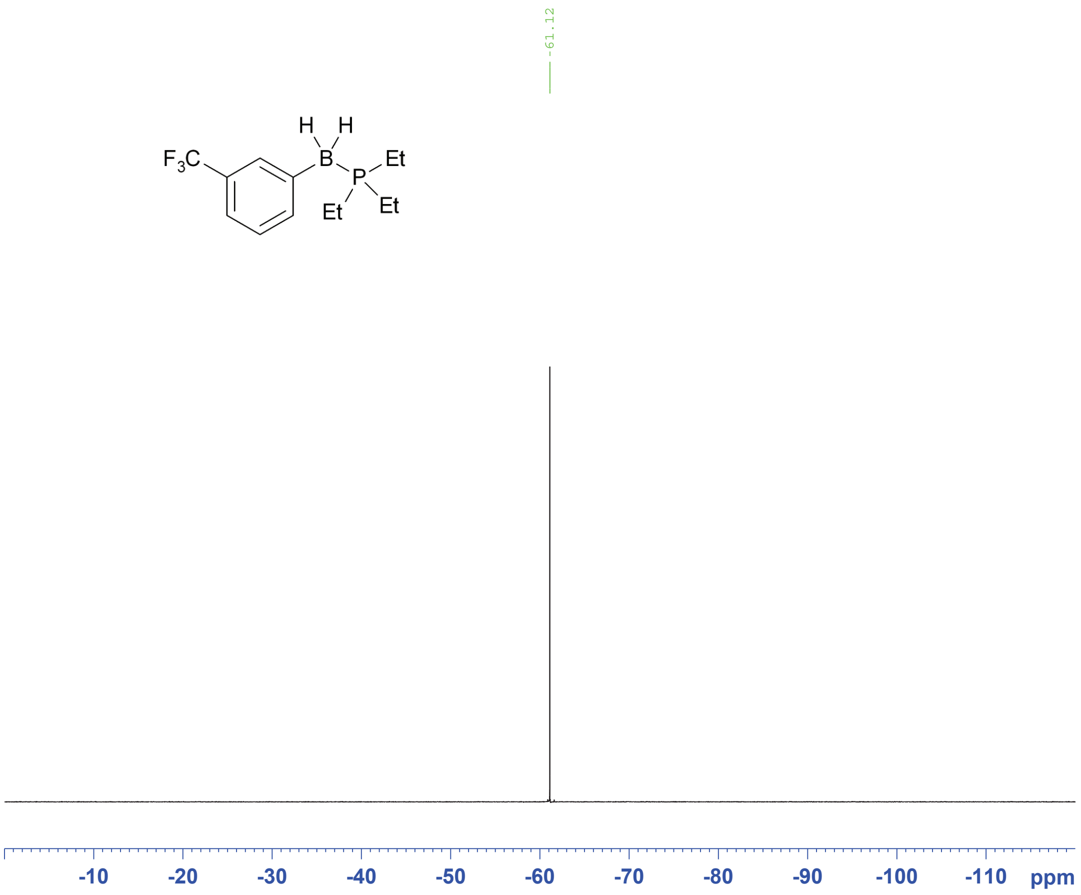

NAME	Jan16-2022
EXPNO	8
PROCNO	1
Date_	20220117
Time	0.18
INSTRUM	av400
PROBHD	5 mm PABBO BB-
PULPROG	zgfhigqn
TD	131072
SOLVENT	DMSO
NS	8
DS	4
SWH	89285.711 Hz
FIDRES	0.681196 Hz
AQ	0.7340532 sec
RG	203
DW	5.600 usec
DE	6.50 usec
TE	296.4 K
D1	1.00000000 sec
D11	0.03000000 sec
D12	0.00002000 sec
TD0	1
======== CHANNEL f1 ========	
NUC1	19F
P1	19.50 usec
PL1	-4.00 dB
PL1W	16.40489006 W
SFO1	376.4607164 MHz
======== CHANNEL f2 ========	
CPDPRG2	waltz16
NUC2	1H
PCPD2	112.00 usec
PL2	-4.00 dB
PL12	18.00 dB
PL2W	24.60733604 W
PL12W	0.15526180 W
SFO2	400.1316005 MHz
SI	65536
SF	376.4984677 MHz
WDW	EM
SSB	0
LB	0.30 Hz
GB	0
PC	1.00

3CF3PhBH2PEt3

IBB-nmr Analysis

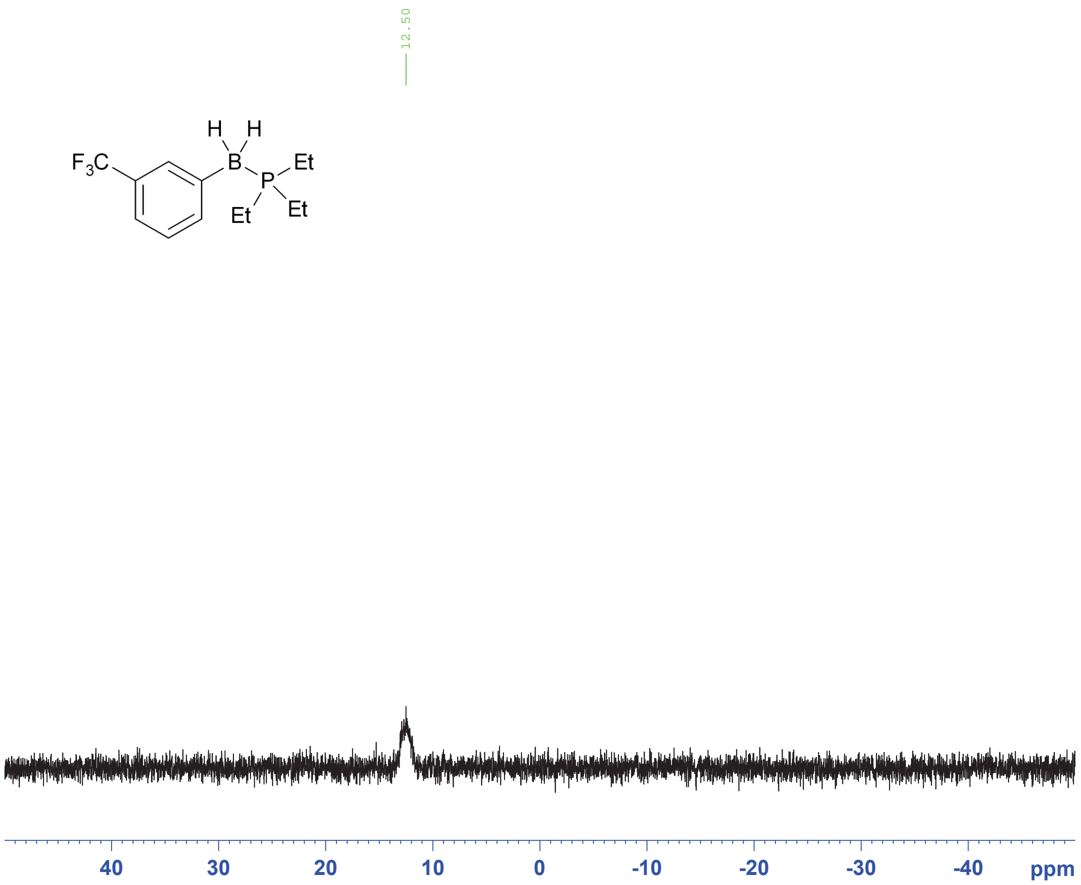

NAME	Jan16-2022
EXPNO	6
PROCNO	1
Date_	20220117
Time	0.05
INSTRUM	av400
PROBHD	5 mm PABBO BB-
PULPROG	zg30
TD	65536
SOLVENT	DMSO
NS	16
DS	4
SWH	64102.563 Hz
FIDRES	0.978127 Hz
AQ	0.5112308 sec
RG	203
DW	7.800 usec
DE	6.50 usec
TE	296.3 K
D1	2.00000000 sec
TD0	1
======== CHANNEL f1 ========	
NUC1	31P
P1	10.00 usec
PL1	-0.70 dB
PL1W	30.10233498 W
SFO1	161.9674942 MHz
SI	32768
SF	161.9754962 MHz
WDW	EM
SSB	0
LB	1.00 Hz
GB	0
PC	1.40

3CF3PhBH2PEt2Ph

B-3-(Trifluoromethyl)phenyl diethyl(phenyl)phosphine–borane (19)

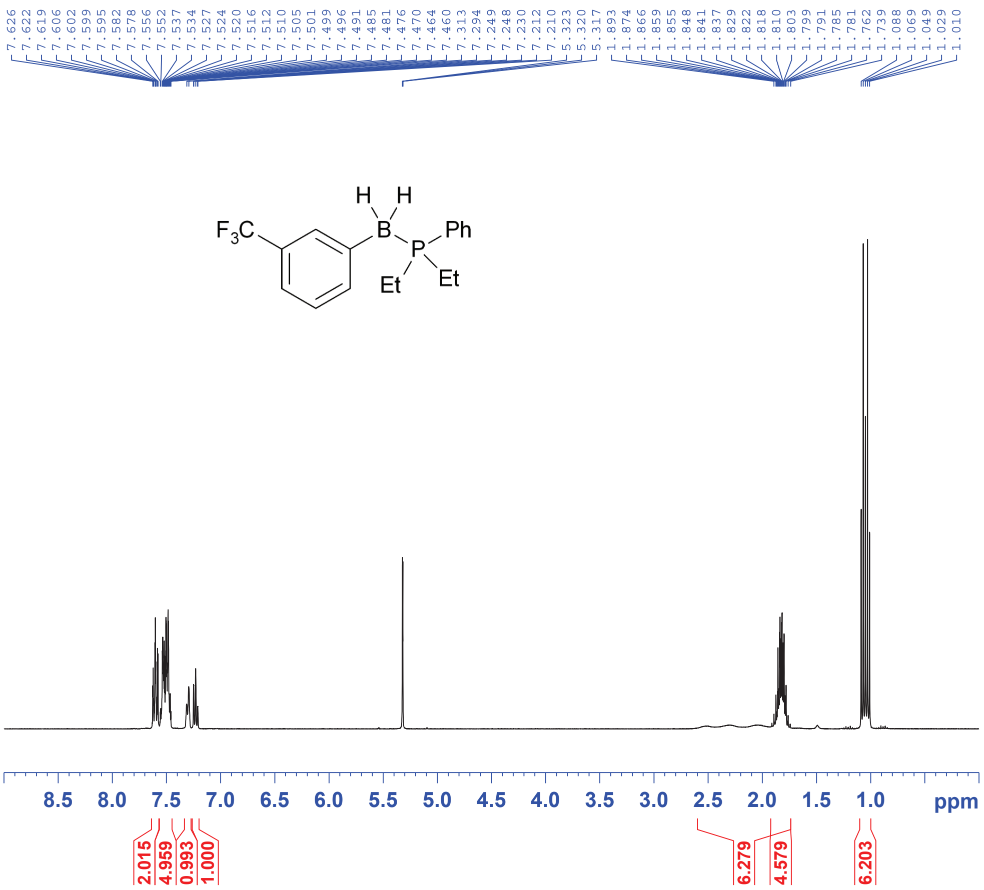

IBB-nmr Analysis

NAME	Mar5-2022
EXPNO	1
PROCNO	1
Date_	20220305
Time	22.04
INSTRUM	av400
PROBHD	5 mm PABBO BB-
PULPROG	zg30
TD	65536
SOLVENT	CD2Cl2
NS	16
DS	2
SWH	8223.685 Hz
FIDRES	0.125483 Hz
AQ	3.9846387 sec
RG	203
DW	60.800 usec
DE	6.50 usec
TE	296.5 K
D1	1.00000000 sec
TD0	1

======== CHANNEL f1 ========

NUC1	1H
P1	14.00 usec
PL1	-1.80 dB
PL1W	14.82738590 W
SFO1	400.1324710 MHz
SI	32768
SF	400.1300154 MHz
WDW	EM
SSB	0
LB	0.30 Hz
GB	0
PC	1.00

3CF3PhBH2PPhEt2

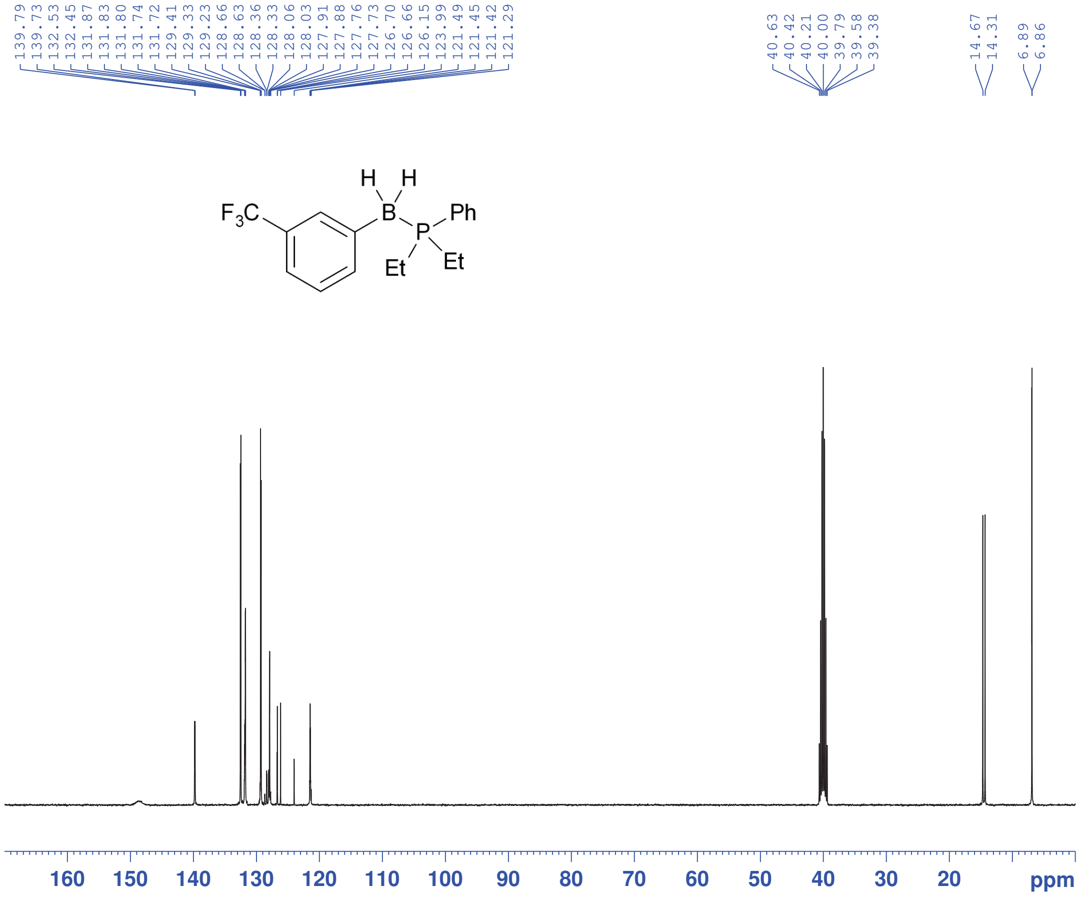

IBB-nmr Analysis

NAME	Aug19-2023
EXPNO	2
PROCNO	1
Date_	20230819
Time	20.58
INSTRUM	av400
PROBHD	5 mm PABBO BB-
PULPROG	zgpg30
TD	65536
SOLVENT	DMSO
NS	1024
DS	2
SWH	24038.461 Hz
FIDRES	0.366798 Hz
AQ	1.3631988 sec
RG	128
DW	20.800 usec
DE	6.50 usec
TE	298.4 K
D1	2.00000000 sec
D11	0.03000000 sec
TD0	1

======== CHANNEL f1 ========

NUC1	13C
P1	9.00 usec
PL1	-3.00 dB
PL1W	73.17333984 W
SFO1	100.6228298 MHz

======== CHANNEL f2 ========

CPDPRG2	waltz16
NUC2	1H
PCPD2	80.00 usec
PL2	-1.70 dB
PL12	13.80 dB
PL13	13.80 dB
PL2W	14.48987389 W
PL12W	0.40838012 W
PL13W	0.40838012 W
SFO2	400.1316005 MHz
SI	32768
SF	100.6127689 MHz
WDW	EM
SSB	0
LB	1.00 Hz
GB	0
PC	0.30

3CF3PhBH2PPhEt2

IBB-nmr Analysis

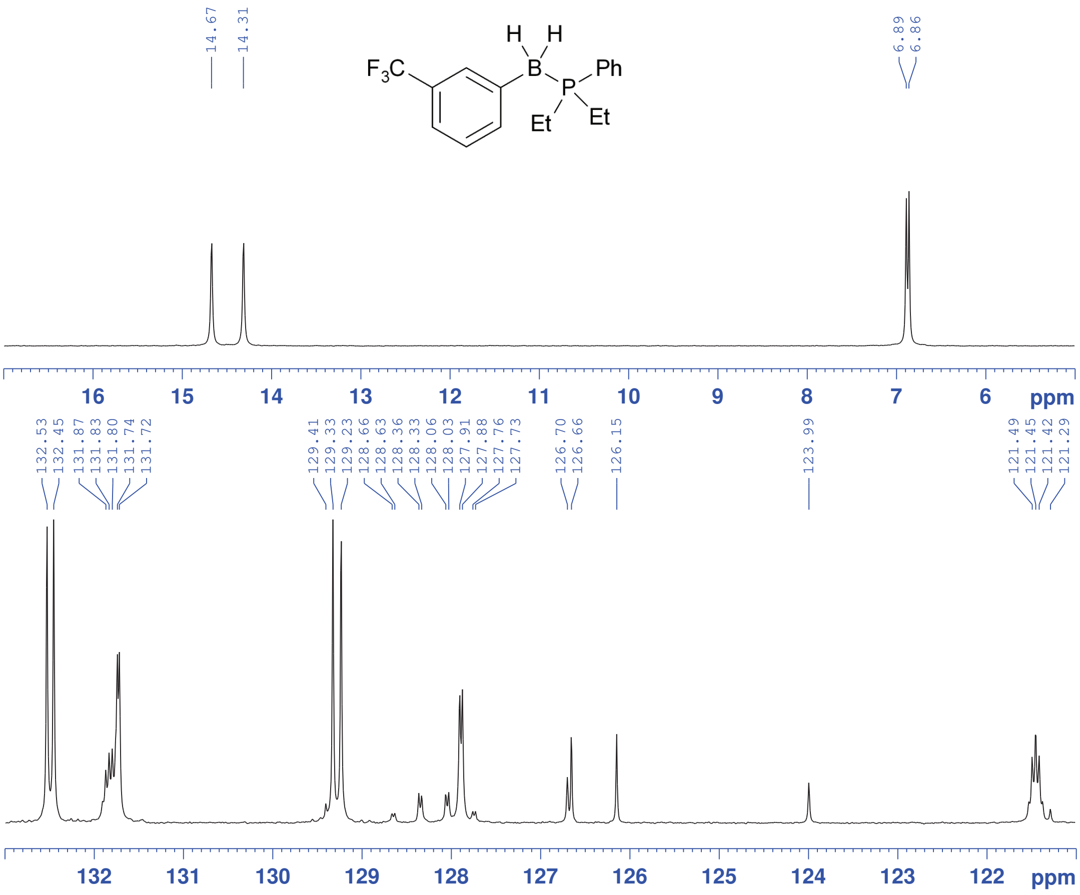


```
NAME          Aug19-2023
EXPNO                  2
PROCNO                 1
Date_           20230819
Time               20.58
INSTRUM            av400
PROBHD   5 mm PABBO BB-
PULPROG          zgpg30
TD                 65536
SOLVENT             DMSO
NS                  1024
DS                     2
SWH            24038.461 Hz
FIDRES          0.366798 Hz
AQ             1.3631988 sec
RG                   128
DW                20.800 usec
DE                  6.50 usec
TE                 298.4 K
D1            2.00000000 sec
D11           0.03000000 sec
TD0                    1

======== CHANNEL f1 ========
NUC1                 13C
P1                  9.00 usec
PL1                -3.00 dB
PL1W         73.17333984 W
SFO1         100.6228298 MHz

======== CHANNEL f2 ========
CPDPRG2          waltz16
NUC2                  1H
PCPD2              80.00 usec
PL2                -1.70 dB
PL12               13.80 dB
PL13               13.80 dB
PL2W         14.48987389 W
PL12W         0.40838012 W
PL13W         0.40838012 W
SFO2         400.1316005 MHz
SI                 32768
SF           100.6127689 MHz
WDW                   EM
SSB                    0
LB                  1.00 Hz
GB                     0
PC                  0.30
```

3CF3PhBH2Et2Ph

IBB-nmr Analysis

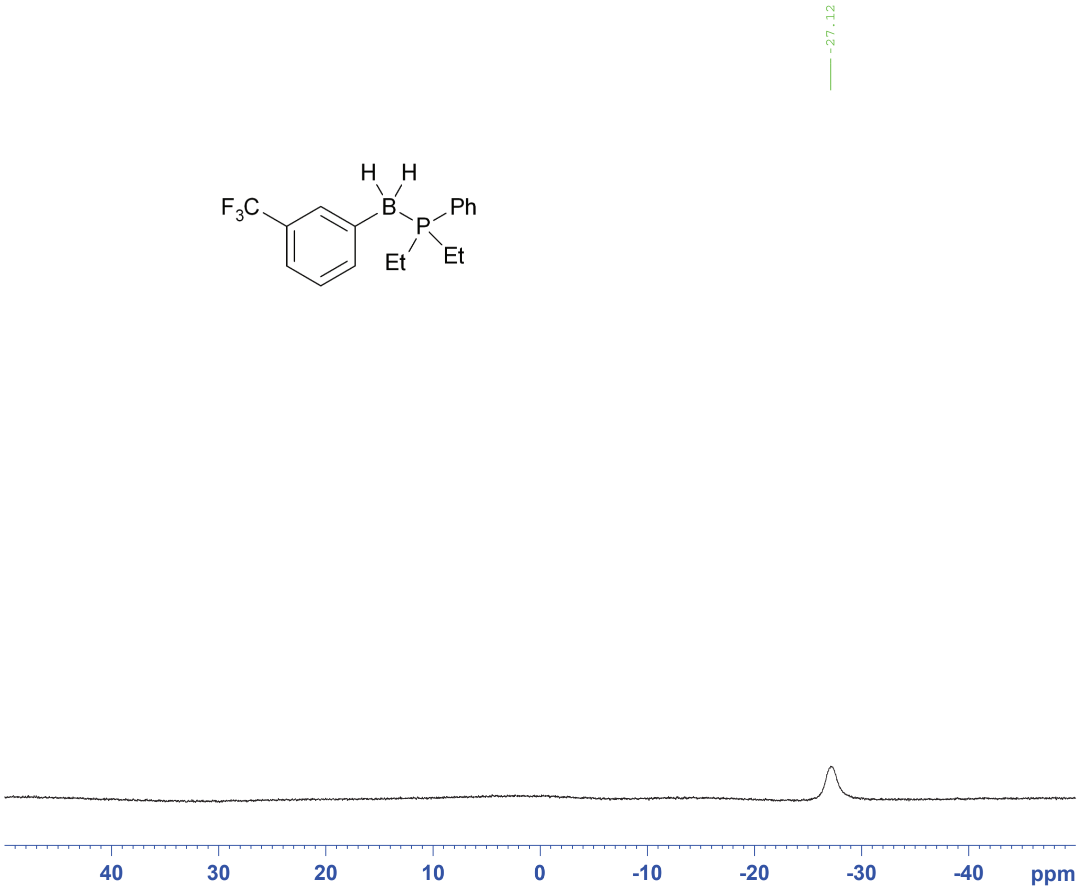


```
NAME          Feb14-2022
EXPNO                  4
PROCNO                 1
Date_           20220214
Time               23.57
INSTRUM            av400
PROBHD   5 mm PABBO BB-
PULPROG             zgig
TD                 32768
SOLVENT             DMSO
NS                   256
DS                     4
SWH            20000.000 Hz
FIDRES          0.610352 Hz
AQ             0.8192500 sec
RG                   203
DW                25.000 usec
DE                 20.00 usec
TE                 296.8 K
D1            1.00000000 sec
D11           0.03000000 sec
TD0                    1

======== CHANNEL f1 ========
NUC1                 11B
P1                  6.30 usec
PL1                -2.00 dB
SFO1         128.3776050 MHz

======== CHANNEL f2 ========
CPDPRG2          waltz16
NUC2                  1H
PCPD2              80.00 usec
PL2                -1.70 dB
PL12               13.80 dB
PL2W         14.48987389 W
PL12W         0.40838012 W
SFO2         400.1316005 MHz
SI                 32768
SF           128.3776183 MHz
WDW                   EM
SSB                    0
LB                  2.00 Hz
GB                     0
PC                  1.40
```

3CF3PhBH2Et2Ph

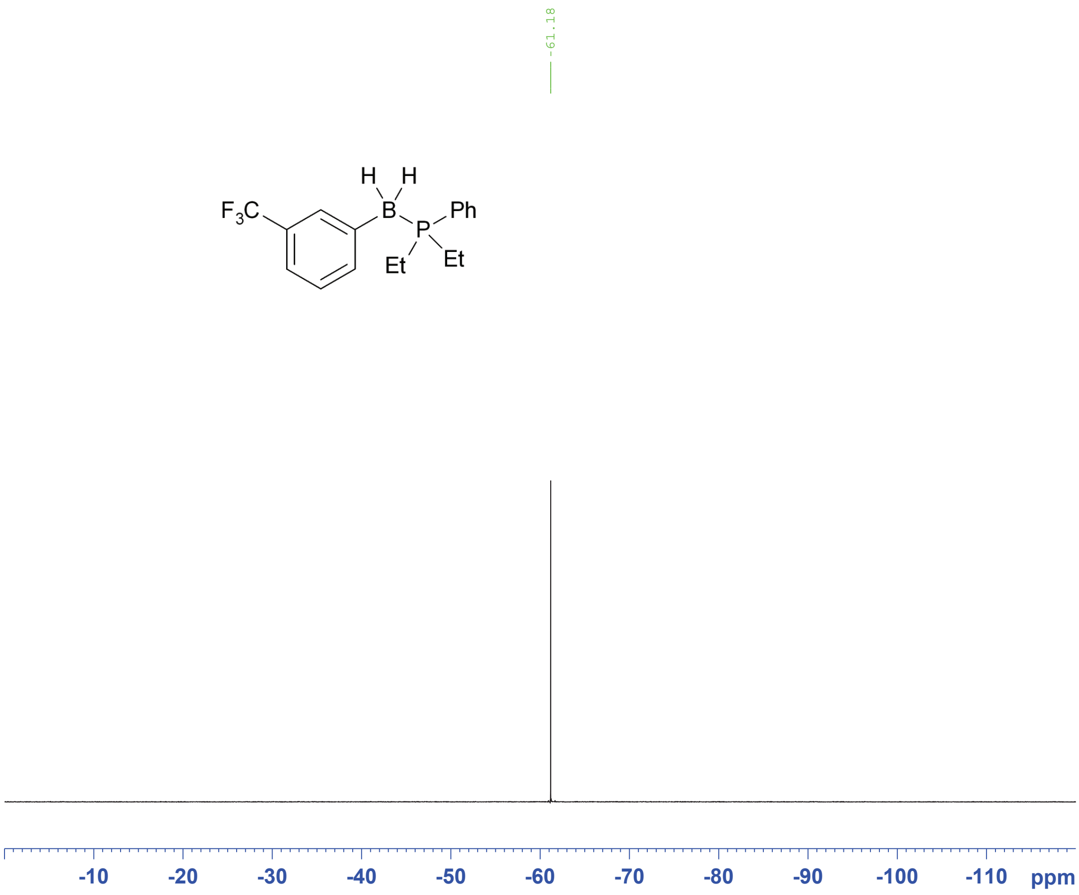

IBB-nmr Analysis

NAME	Feb14-2022
EXPNO	5
PROCNO	1
Date_	20220215
Time	0.01
INSTRUM	av400
PROBHD	5 mm PABBO BB-
PULPROG	zgfhigqn
TD	131072
SOLVENT	DMSO
NS	8
DS	4
SWH	89285.711 Hz
FIDRES	0.681196 Hz
AQ	0.7340532 sec
RG	203
DW	5.600 usec
DE	6.50 usec
TE	296.3 K
D1	1.00000000 sec
D11	0.03000000 sec
D12	0.00002000 sec
TD0	1

======== CHANNEL f1 ========

NUC1	19F
P1	19.50 usec
PL1	-4.00 dB
PL1W	16.40489006 W
SFO1	376.4607164 MHz

======== CHANNEL f2 ========

CPDPRG2	waltz16
NUC2	1H
PCPD2	112.00 usec
PL2	-4.00 dB
PL12	18.00 dB
PL2W	24.60733604 W
PL12W	0.15526180 W
SFO2	400.1316005 MHz
SI	65536
SF	376.4984617 MHz
WDW	EM
SSB	0
LB	0.30 Hz
GB	0
PC	1.00

3CF3PhBH2Et2Ph

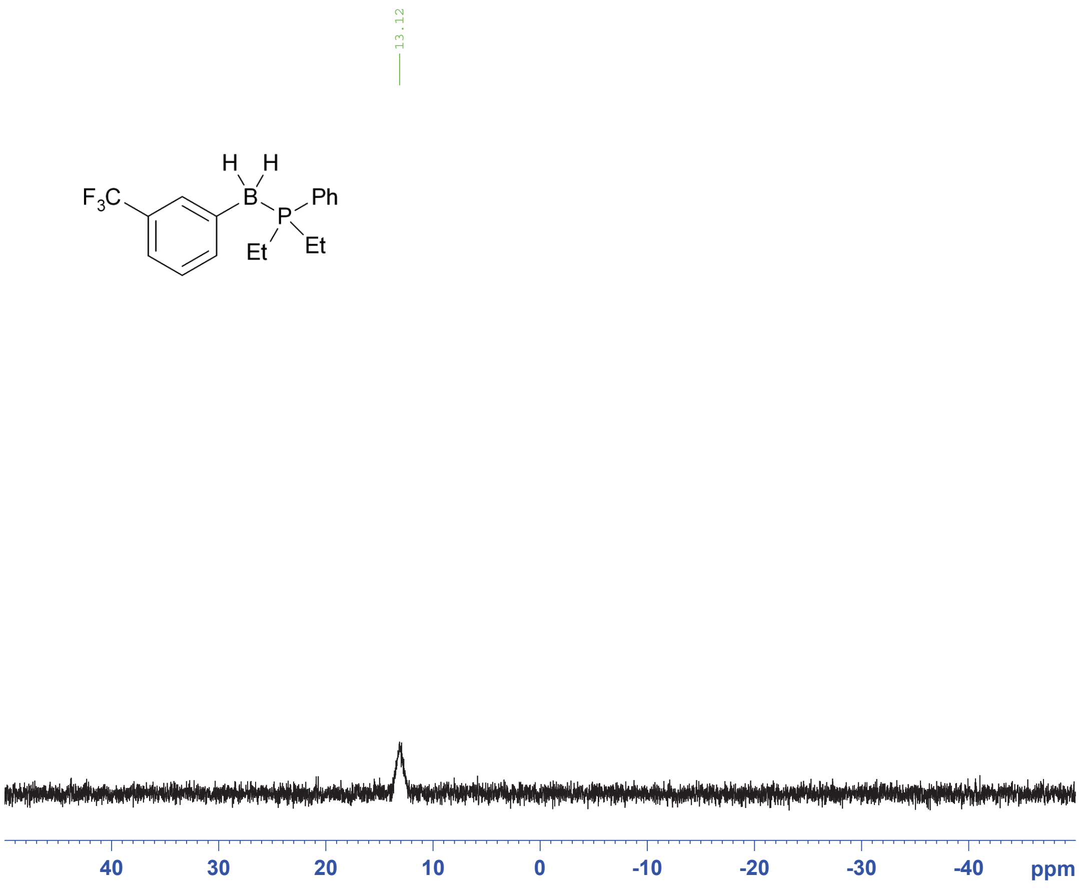

IBB-nmr Analysis

NAME	Feb14-2022
EXPNO	3
PROCNO	1
Date_	20220214
Time	23.46
INSTRUM	av400
PROBHD	5 mm PABBO BB-
PULPROG	zg30
TD	65536
SOLVENT	DMSO
NS	64
DS	4
SWH	64102.563 Hz
FIDRES	0.978127 Hz
AQ	0.5112308 sec
RG	203
DW	7.800 usec
DE	6.50 usec
TE	296.1 K
D1	2.00000000 sec
TD0	1

======== CHANNEL f1 ========

NUC1	31P
P1	10.00 usec
PL1	-0.70 dB
PL1W	30.10233498 W
SFO1	161.9674942 MHz
SI	32768
SF	161.9754962 MHz
WDW	EM
SSB	0
LB	1.00 Hz
GB	0
PC	1.40

3CF3PhBH2P(i-Pr)3

B-3-(Trifluoromethyl)phenyl triisopropylphosphine–borane (20)

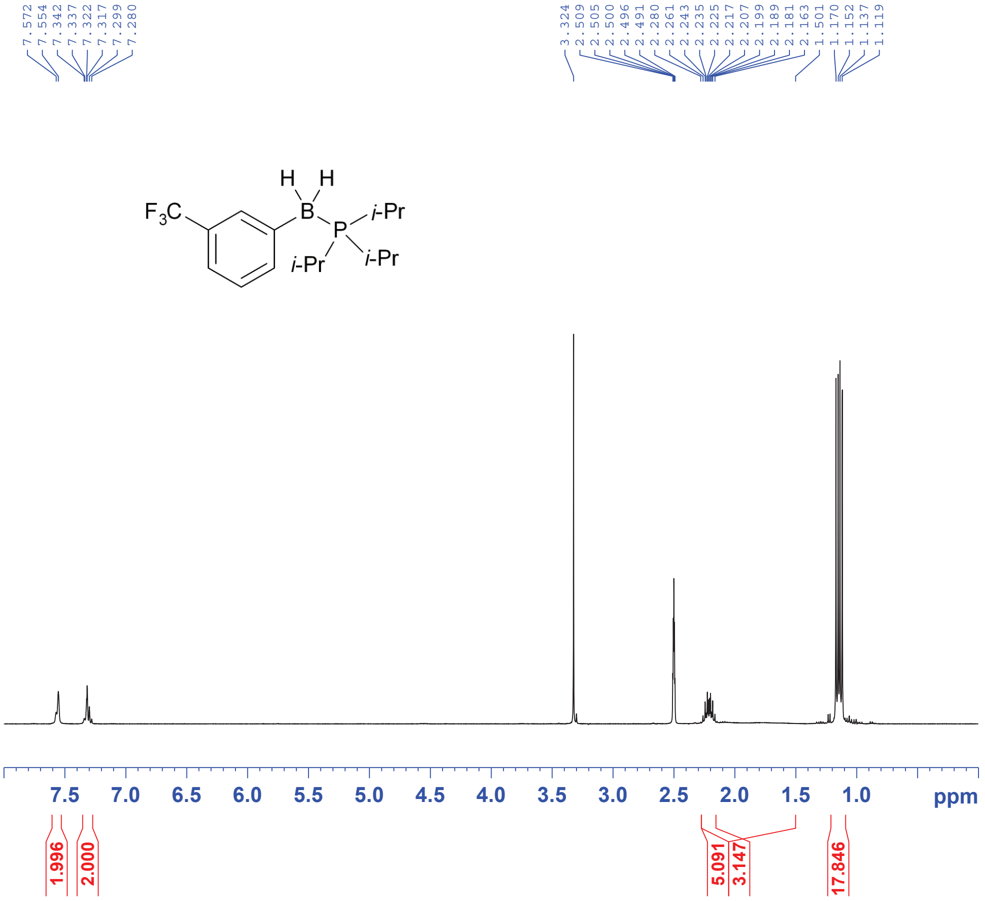

IBB-nmr Analysis

NAME	Apr29-2022
EXPNO	1
PROCNO	1
Date_	20220429
Time	18.21
INSTRUM	av400
PROBHD	5 mm PABBO BB-
PULPROG	zg30
TD	65536
SOLVENT	DMSO
NS	16
DS	2
SWH	8223.685 Hz
FIDRES	0.125483 Hz
AQ	3.9846387 sec
RG	203
DW	60.800 usec
DE	6.50 usec
TE	296.8 K
D1	1.00000000 sec
TD0	1

======== CHANNEL f1 ========

NUC1	1H
P1	14.00 usec
PL1	-1.80 dB
PL1W	14.82738590 W
SFO1	400.1324710 MHz
SI	32768
SF	400.1300028 MHz
WDW	EM
SSB	0
LB	0.30 Hz
GB	0
PC	1.00

3CF3PhBH2P(i-Pr)3

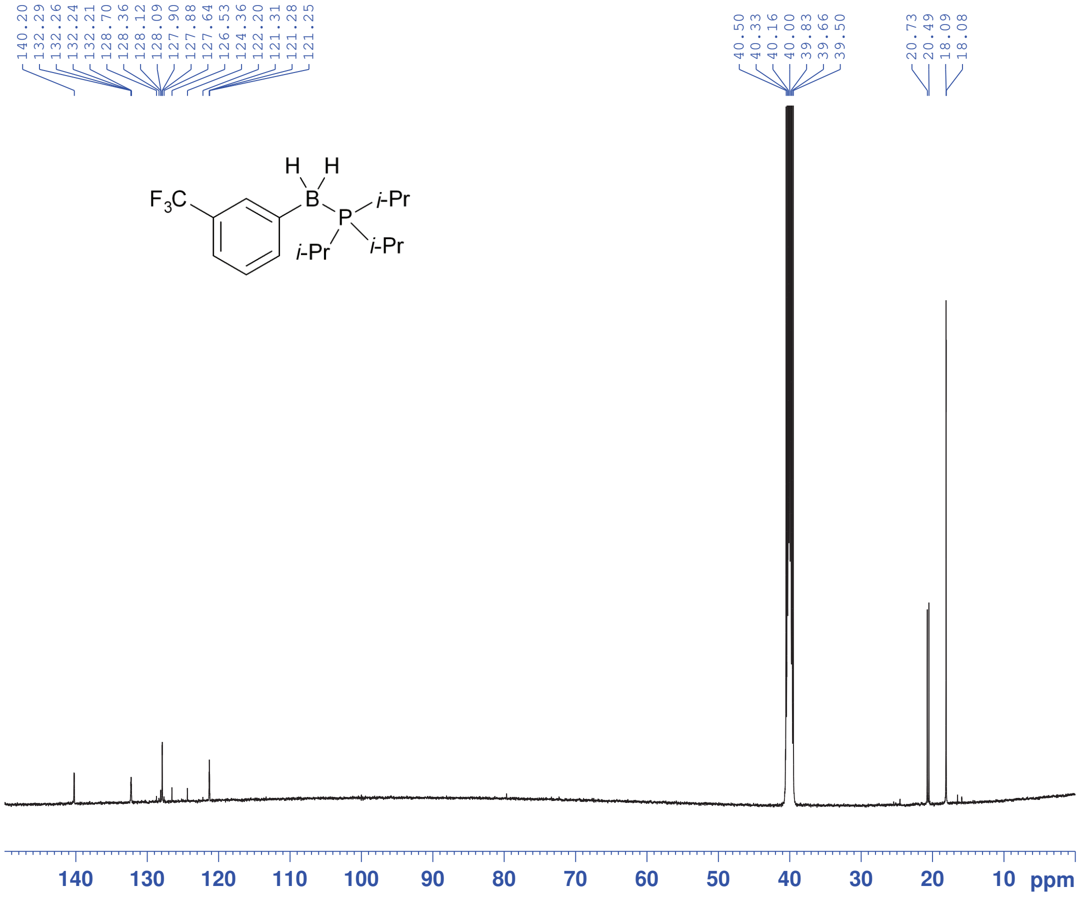

IBB-nmr Analysis

NAME	Apr29-2022
EXPNO	8
PROCNO	1
Date_	20220429
Time	19.54
INSTRUM	av500
PROBHD	5 mm CPDCH 13C
PULPROG	zgpg30
TD	65536
SOLVENT	DMSO
NS	1024
DS	4
SWH	30030.029 Hz
FIDRES	0.458222 Hz
AQ	1.0912410 sec
RG	35.9
DW	16.650 usec
DE	20.00 usec
TE	298.0 K
D1	2.00000000 sec
D11	0.03000000 sec
TD0	1

======== CHANNEL f1 ========

NUC1	13C
P1	10.00 usec
PL1	-5.00 dB
PL1W	14.29790783 W
SFO1	125.7703643 MHz

======== CHANNEL f2 ========

CPDPRG2	waltz16
NUC2	1H
PCPD2	80.00 usec
PL2	-4.00 dB
PL12	14.06 dB
PL13	17.00 dB
PL2W	6.30957365 W
PL12W	0.09862794 W
PL13W	0.05011872 W
SFO2	500.1320005 MHz
SI	32768
SF	125.7577875 MHz
WDW	EM
SSB	0
LB	1.00 Hz
GB	0
PC	0.30

3CF3PhBH2P(i-Pr)3

IBB-nmr Analysis

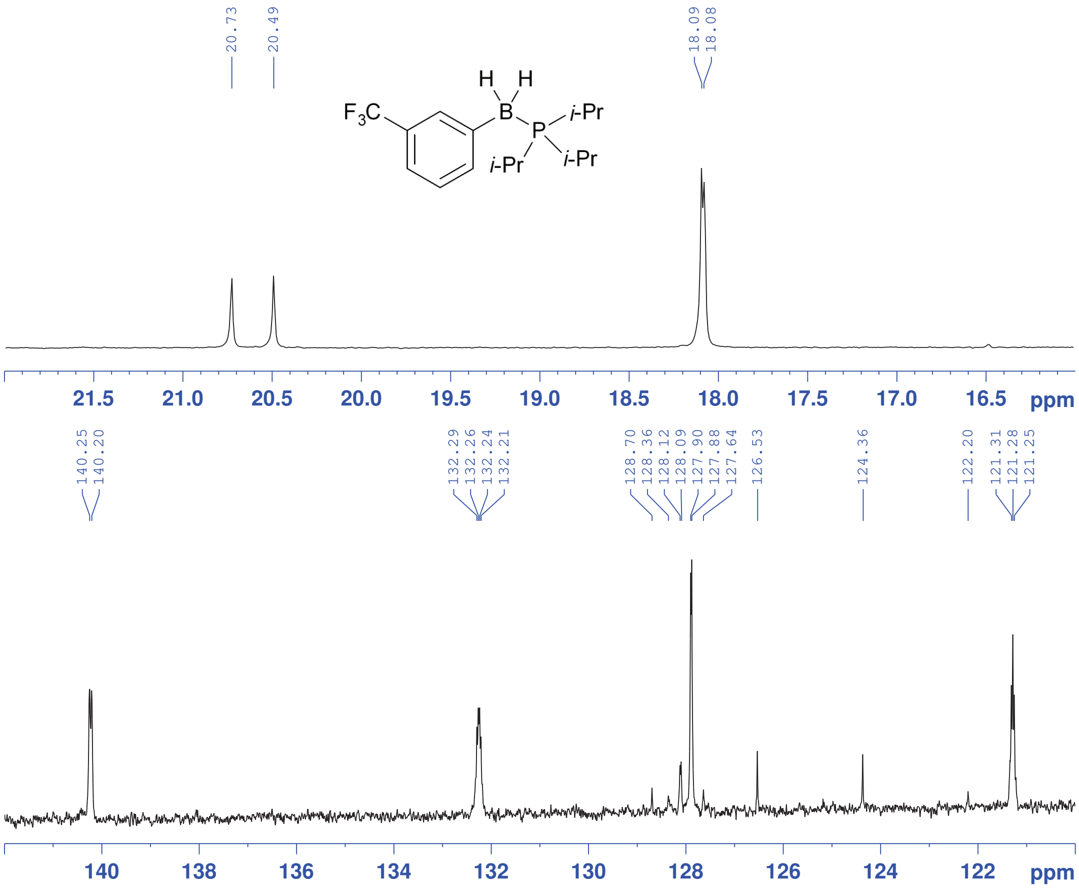


```
NAME          Apr29-2022
EXPNO                  8
PROCNO                 1
Date_           20220429
Time               19.54
INSTRUM            av500
PROBHD   5 mm CPDCH 13C
PULPROG           zgpg30
TD                 65536
SOLVENT             DMSO
NS                  1024
DS                     4
SWH            30030.029 Hz
FIDRES          0.458222 Hz
AQ             1.0912410 sec
RG                  35.9
DW                16.650 usec
DE                 20.00 usec
TE                 298.0 K
D1            2.00000000 sec
D11           0.03000000 sec
TD0                    1

======== CHANNEL f1 ========
NUC1                 13C
P1                 10.00 usec
PL1                -5.00 dB
PL1W         14.29790783 W
SFO1         125.7703643 MHz

======== CHANNEL f2 ========
CPDPRG2          waltz16
NUC2                  1H
PCPD2              80.00 usec
PL2                -4.00 dB
PL12               14.06 dB
PL13               17.00 dB
PL2W          6.30957365 W
PL12W         0.09862794 W
PL13W         0.05011872 W
SFO2         500.1320005 MHz
SI                 32768
SF           125.7577875 MHz
WDW                   EM
SSB                    0
LB                  1.00 Hz
GB                     0
PC                  0.30
```

3CF3PhBH2P(i-Pr)3

IBB-nmr Analysis

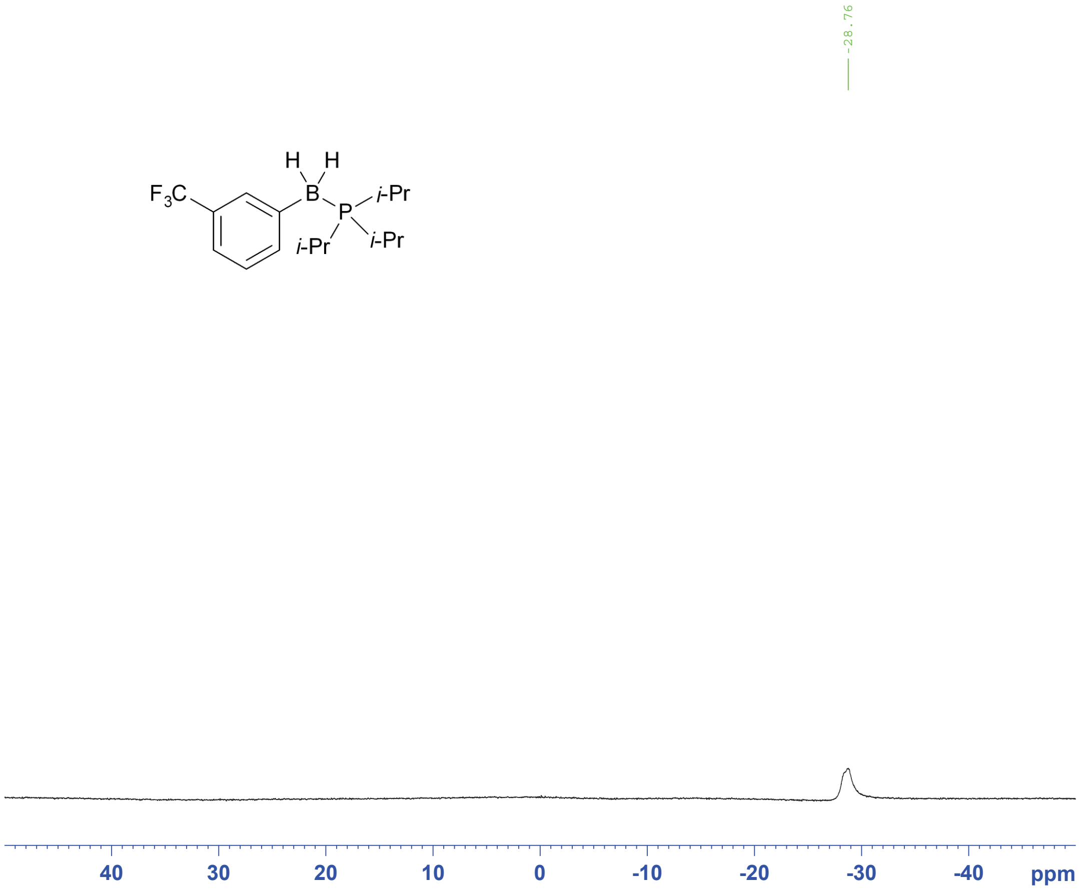


```
NAME          Apr29-2022
EXPNO                  5
PROCNO                 1
Date_           20220429
Time               18.39
INSTRUM            av400
PROBHD   5 mm PABBO BB-
PULPROG             zgig
TD                 32768
SOLVENT             DMSO
NS                   128
DS                     4
SWH            20000.000 Hz
FIDRES          0.610352 Hz
AQ             0.8192500 sec
RG                   203
DW                25.000 usec
DE                 20.00 usec
TE                 297.3 K
D1            1.00000000 sec
D11           0.03000000 sec
TD0                    1

======== CHANNEL f1 ========
NUC1                 11B
P1                  6.30 usec
PL1                -2.00 dB
SFO1         128.3776050 MHz

======== CHANNEL f2 ========
CPDPRG2          waltz16
NUC2                  1H
PCPD2              80.00 usec
PL2                -1.70 dB
PL12               13.80 dB
PL2W         14.48987389 W
PL12W         0.40838012 W
SFO2         400.1316005 MHz
SI                 32768
SF           128.3776183 MHz
WDW                   EM
SSB                    0
LB                  2.00 Hz
GB                     0
PC                  1.40
```

3CF3PhBH2P(i-Pr)3

IBB-nmr Analysis

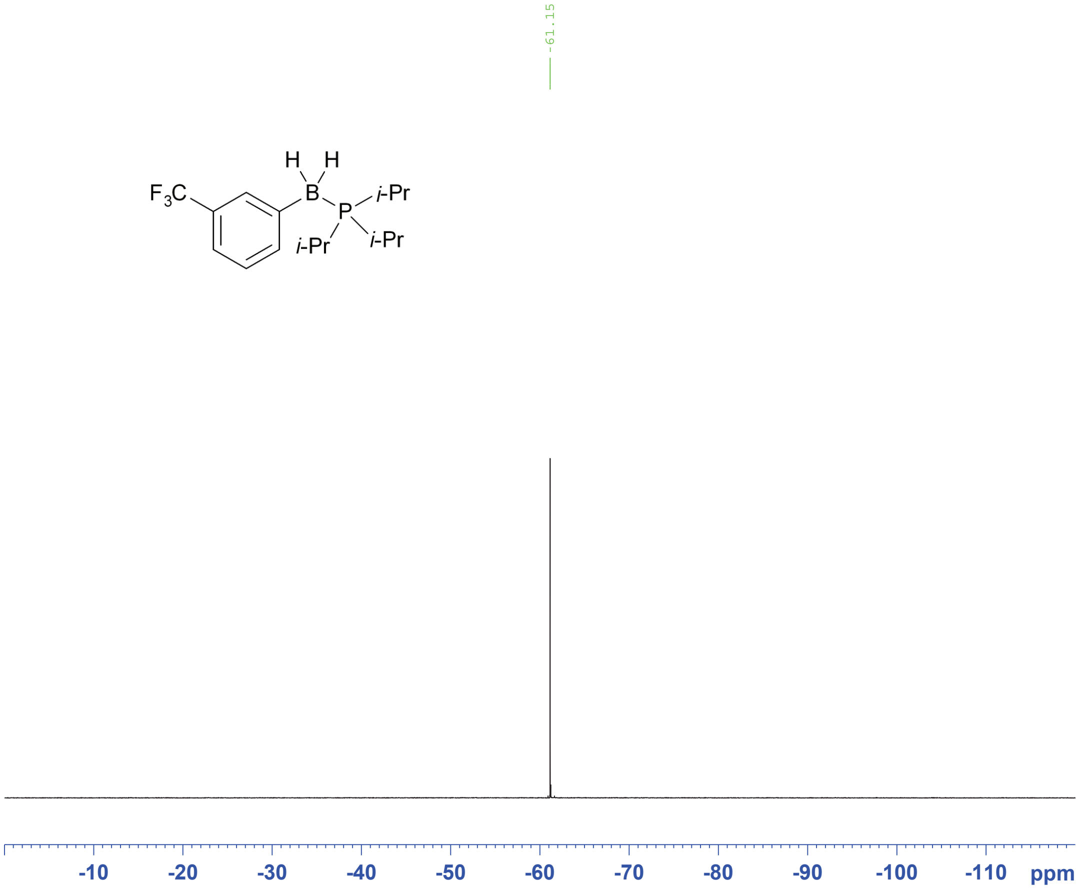

NAME	Apr29-2022
EXPNO	4
PROCNO	1
Date_	20220429
Time	18.32
INSTRUM	av400
PROBHD	5 mm PABBO BB-
PULPROG	zgfhigqn
TD	131072
SOLVENT	DMSO
NS	8
DS	4
SWH	89285.711 Hz
FIDRES	0.681196 Hz
AQ	0.7340532 sec
RG	203
DW	5.600 usec
DE	6.50 usec
TE	296.7 K
D1	1.00000000 sec
D11	0.03000000 sec
D12	0.00002000 sec
TD0	1
======== CHANNEL f1 ========	
NUC1	19F
P1	19.50 usec
PL1	-4.00 dB
PL1W	16.40489006 W
SFO1	376.4607164 MHz
======== CHANNEL f2 ========	
CPDPRG2	waltz16
NUC2	1H
PCPD2	112.00 usec
PL2	-4.00 dB
PL12	18.00 dB
PL2W	24.60733604 W
PL12W	0.15526180 W
SFO2	400.1316005 MHz
SI	65536
SF	376.4984617 MHz
WDW	EM
SSB	0
LB	0.30 Hz
GB	0
PC	1.00

3CF3PhBH2P(i-Pr)3

IBB-nmr Analysis

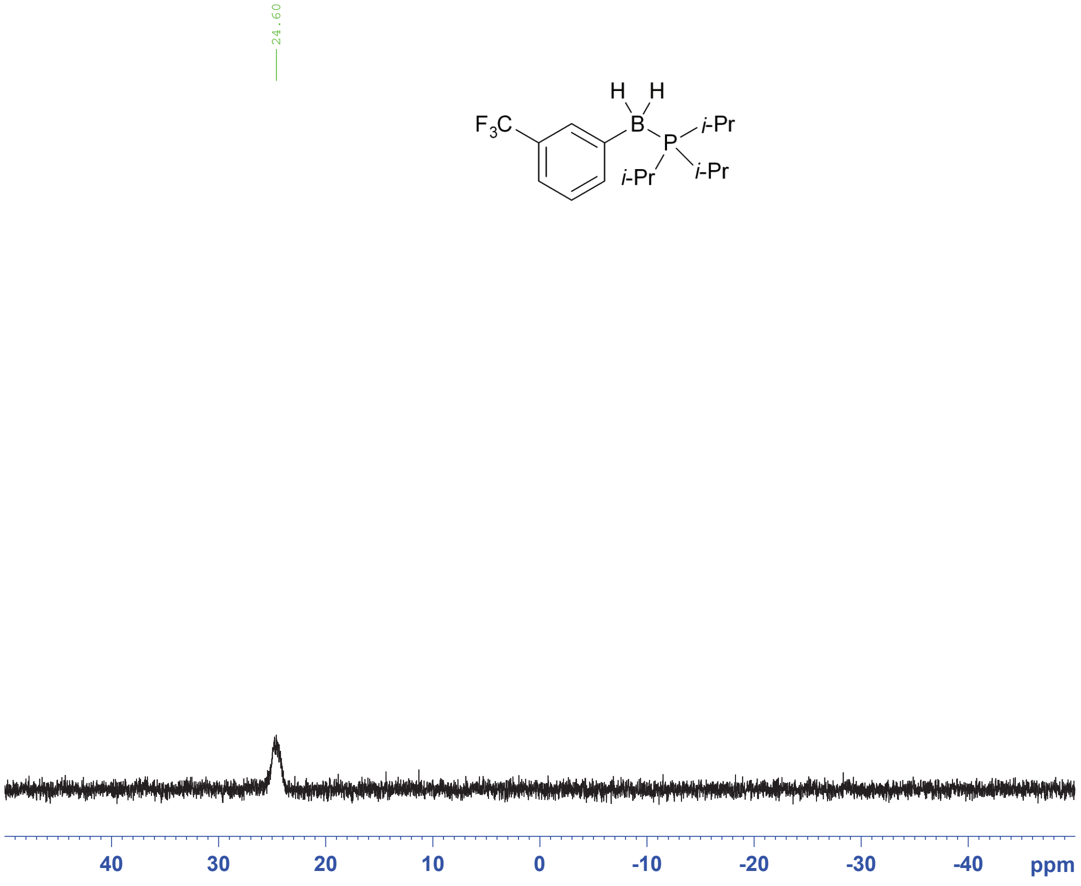

NAME	Apr29-2022
EXPNO	3
PROCNO	1
Date_	20220429
Time	18.28
INSTRUM	av400
PROBHD	5 mm PABBO BB-
PULPROG	zg30
TD	65536
SOLVENT	DMSO
NS	64
DS	4
SWH	64102.563 Hz
FIDRES	0.978127 Hz
AQ	0.5112308 sec
RG	203
DW	7.800 usec
DE	6.50 usec
TE	296.8 K
D1	2.00000000 sec
TD0	1
======== CHANNEL f1 ========	
NUC1	31P
P1	10.00 usec
PL1	-0.70 dB
PL1W	30.10233498 W
SFO1	161.9674942 MHz
SI	32768
SF	161.9754962 MHz
WDW	EM
SSB	0
LB	1.00 Hz
GB	0
PC	1.40

B-3-(Trifluoromethyl)phenyl diisopropyl(phenyl)phosphine–borane (21)

3CF3PhBH2PPh(i-Pr)2

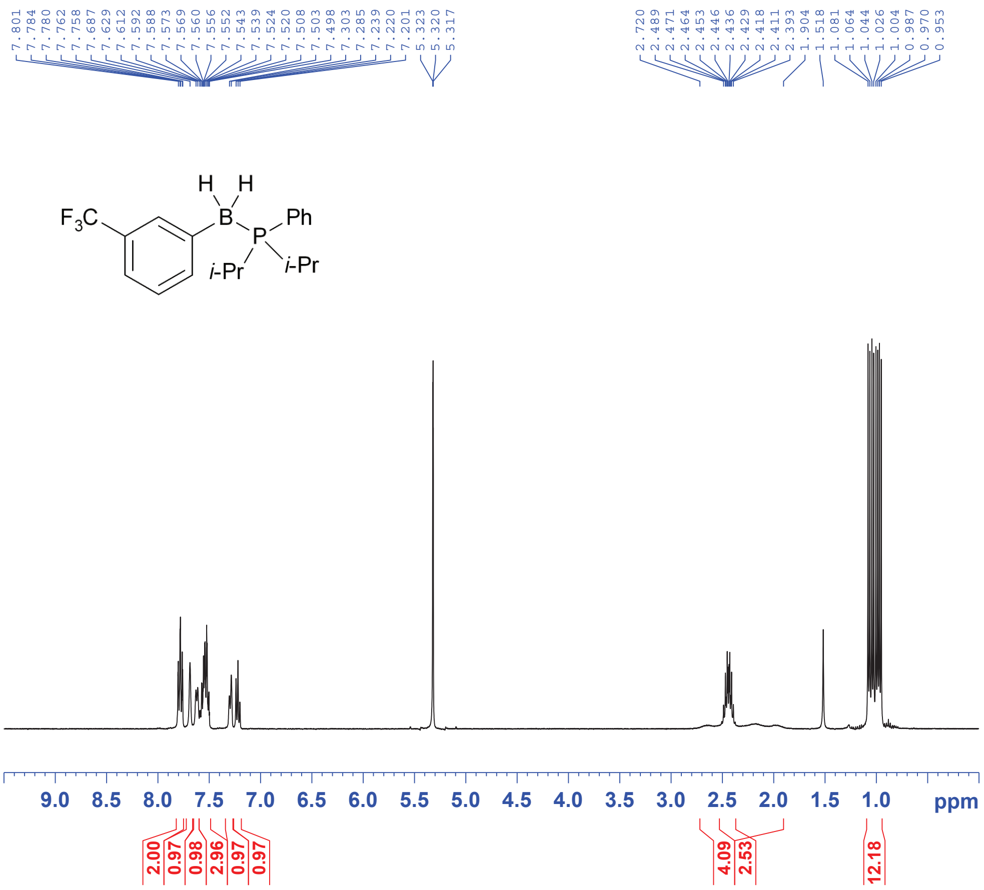

IBB-nmr Analysis

```
NAME          Jun12-2022
EXPNO                  8
PROCNO                 1
Date_           20220613
Time                0.40
INSTRUM            av400
PROBHD    5 mm PABBO BB-
PULPROG             zg30
TD                 65536
SOLVENT            CD2Cl2
NS                    16
DS                     2
SWH             8223.685 Hz
FIDRES          0.125483 Hz
AQ             3.9846387 sec
RG                   203
DW                60.800 usec
DE                  6.50 usec
TE                 296.9 K
D1            1.00000000 sec
TD0                    1

======== CHANNEL f1 ========
NUC1                  1H
P1                 14.00 usec
PL1                -1.80 dB
PL1W         14.82738590 W
SFO1         400.1324710 MHz
SI                 32768
SF           400.1300154 MHz
WDW                   EM
SSB                    0
LB                  0.30 Hz
GB                     0
PC                  1.00
```

3CF3PhBH2PPh(i-Pr)2

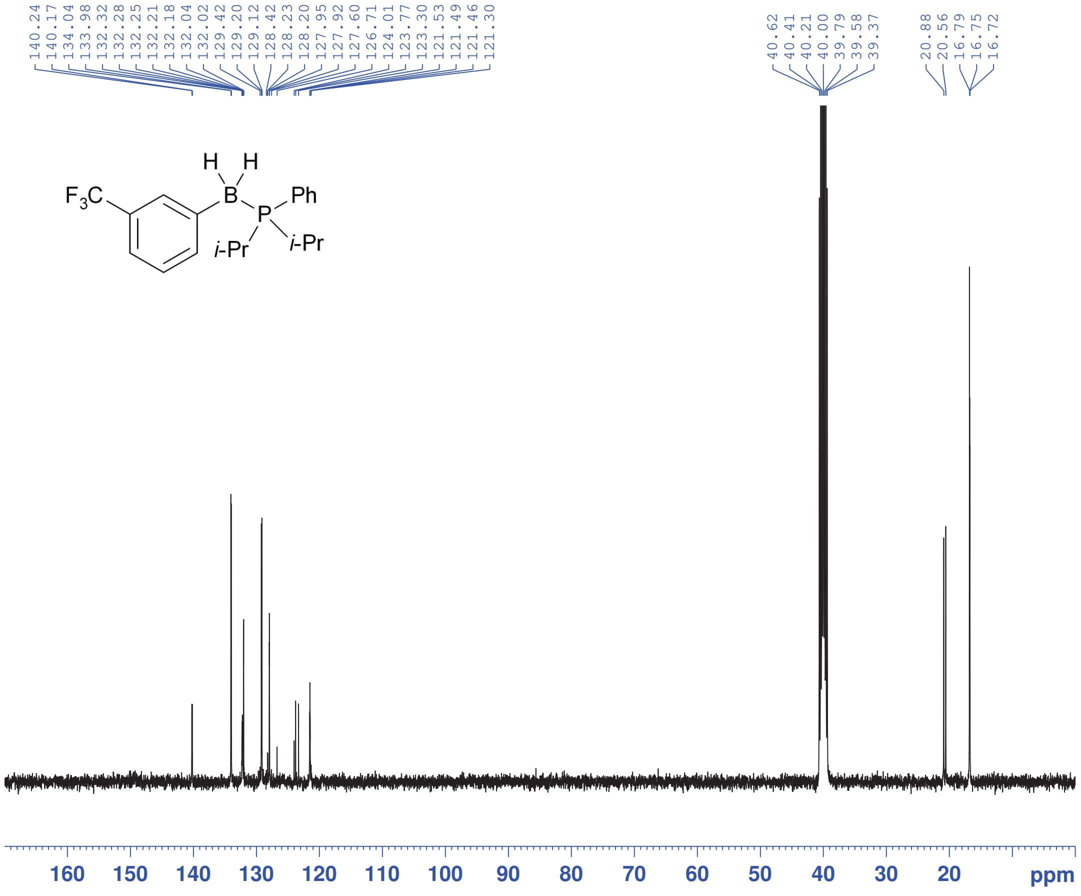

IBB-nmr Analysis

```
NAME          Aug21-2023
EXPNO                  2
PROCNO                 1
Date_           20230821
Time                7.55
INSTRUM            av400
PROBHD    5 mm PABBO BB-
PULPROG           zgpg30
TD                 65536
SOLVENT             DMSO
NS                  2048
DS                     2
SWH            24038.461 Hz
FIDRES          0.366798 Hz
AQ             1.3631988 sec
RG                   128
DW                20.800 usec
DE                  6.50 usec
TE                 298.7 K
D1            2.00000000 sec
D11           0.03000000 sec
TD0                    1

======== CHANNEL f1 ========
NUC1                 13C
P1                  9.00 usec
PL1                -3.00 dB
PL1W         73.17333984 W
SFO1         100.6228298 MHz

======== CHANNEL f2 ========
CPDPRG2          waltz16
NUC2                  1H
PCPD2              80.00 usec
PL2                -1.70 dB
PL12               13.80 dB
PL13               13.80 dB
PL2W         14.48987389 W
PL12W         0.40838012 W
PL13W         0.40838012 W
SFO2         400.1316005 MHz
SI                 32768
SF           100.6127696 MHz
WDW                   EM
SSB                    0
LB                  1.00 Hz
GB                     0
PC                  0.30
```

3CF3PhBH2PPh(i-Pr)2

IBB-nmr Analysis

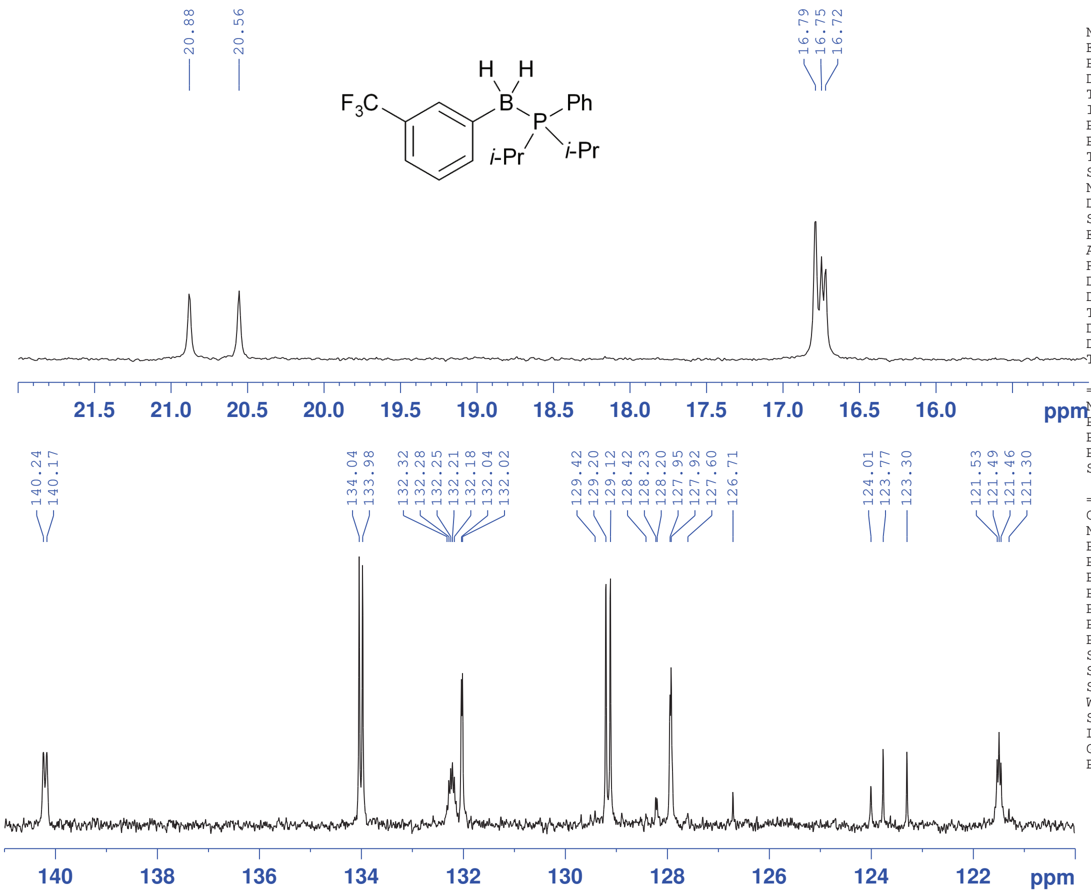


```
NAME          Aug21-2023
EXPNO                  2
PROCNO                 1
Date_           20230821
Time                7.55
INSTRUM            av400
PROBHD    5 mm PABBO BB-
PULPROG          zgpg30
TD                 65536
SOLVENT             DMSO
NS                  2048
DS                     2
SWH            24038.461 Hz
FIDRES          0.366798 Hz
AQ             1.3631988 sec
RG                   128
DW                20.800 usec
DE                  6.50 usec
TE                 298.7 K
D1            2.00000000 sec
D11           0.03000000 sec
TD0                    1

======== CHANNEL f1 ========
NUC1                 13C
P1                  9.00 usec
PL1                -3.00 dB
PL1W         73.17333984 W
SFO1         100.6228298 MHz

======== CHANNEL f2 ========
CPDPRG2          waltz16
NUC2                  1H
PCPD2              80.00 usec
PL2                -1.70 dB
PL12               13.80 dB
PL13               13.80 dB
PL2W         14.48987389 W
PL12W         0.40838012 W
PL13W         0.40838012 W
SFO2         400.1316005 MHz
SI                 32768
SF           100.6127696 MHz
WDW                   EM
SSB                    0
LB                  1.00 Hz
GB                     0
PC                  0.30
```

3CF3PhBH2PPh(i-Pr)2

IBB-nmr Analysis

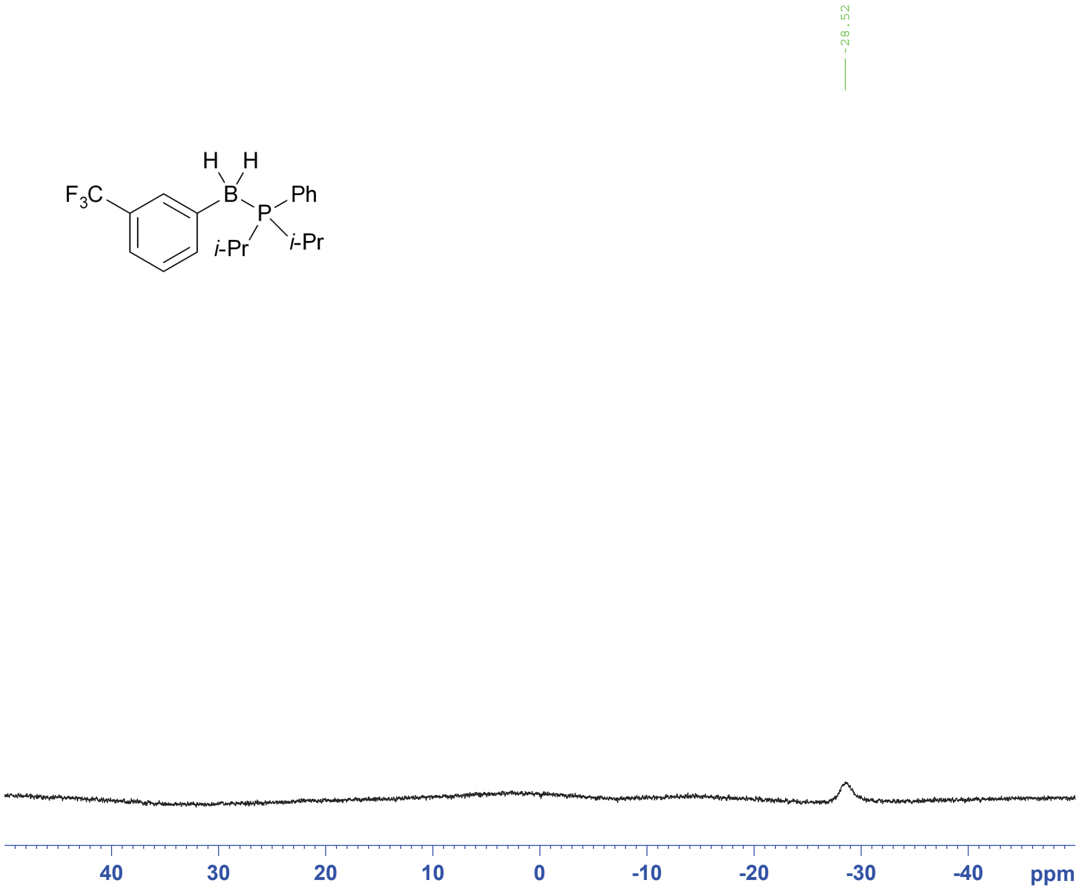


```
NAME          Jun12-2022
EXPNO                  2
PROCNO                 1
Date_           20220612
Time               23.00
INSTRUM            av400
PROBHD    5 mm PABBO BB-
PULPROG             zgig
TD                 32768
SOLVENT             DMSO
NS                   256
DS                     4
SWH            20000.000 Hz
FIDRES          0.610352 Hz
AQ             0.8192500 sec
RG                   203
DW                25.000 usec
DE                 20.00 usec
TE                 297.6 K
D1            1.00000000 sec
D11           0.03000000 sec
TD0                    1

======== CHANNEL f1 ========
NUC1                 11B
P1                  6.30 usec
PL1                -2.00 dB
SFO1         128.3776050 MHz

======== CHANNEL f2 ========
CPDPRG2          waltz16
NUC2                  1H
PCPD2              80.00 usec
PL2                -1.70 dB
PL12               13.80 dB
PL2W         14.48987389 W
PL12W         0.40838012 W
SFO2         400.1316005 MHz
SI                 32768
SF           128.3776183 MHz
WDW                   EM
SSB                    0
LB                  2.00 Hz
GB                     0
PC                  1.40
```

3CF3PhBH2PPh(i-Pr)2

IBB-nmr Analysis

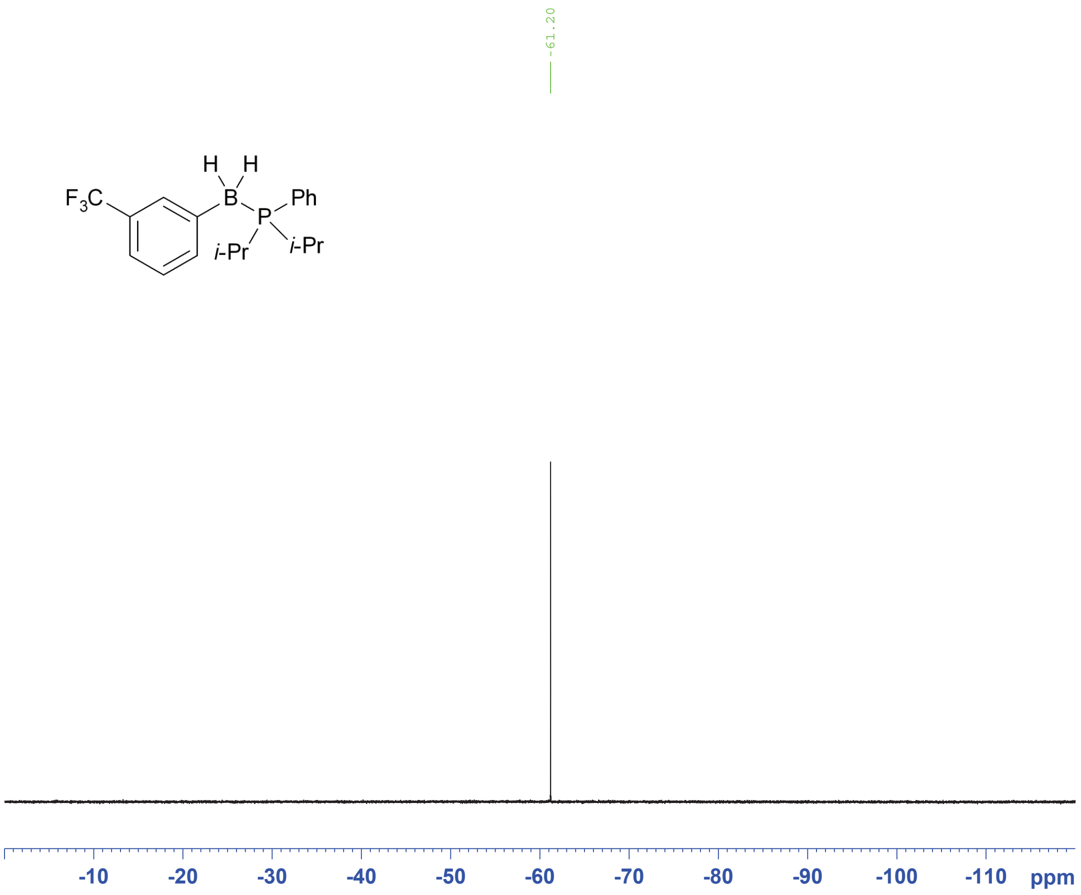


```
NAME            Jun12-2022
EXPNO                    3
PROCNO                   1
Date_             20220612
Time                 23.04
INSTRUM              av400
PROBHD     5 mm PABBO BB-
PULPROG           zgfhigqn
TD                  131072
SOLVENT               DMSO
NS                       8
DS                       4
SWH              89285.711 Hz
FIDRES            0.681196 Hz
AQ               0.7340532 sec
RG                     203
DW                   5.600 usec
DE                    6.50 usec
TE                   297.1 K
D1              1.00000000 sec
D11             0.03000000 sec
D12             0.00002000 sec
TD0                      1

======== CHANNEL f1 ========
NUC1                   19F
P1                   19.50 usec
PL1                  -4.00 dB
PL1W           16.40489006 W
SFO1           376.4607164 MHz

======== CHANNEL f2 ========
CPDPRG2            waltz16
NUC2                    1H
PCPD2               112.00 usec
PL2                  -4.00 dB
PL12                 18.00 dB
PL2W           24.60733604 W
PL12W           0.15526180 W
SFO2           400.1316005 MHz
SI                   65536
SF             376.4984617 MHz
WDW                     EM
SSB                      0
LB                    0.30 Hz
GB                       0
PC                    1.00
```

3CF3PhBH2PPh(i-Pr)2

IBB-nmr Analysis

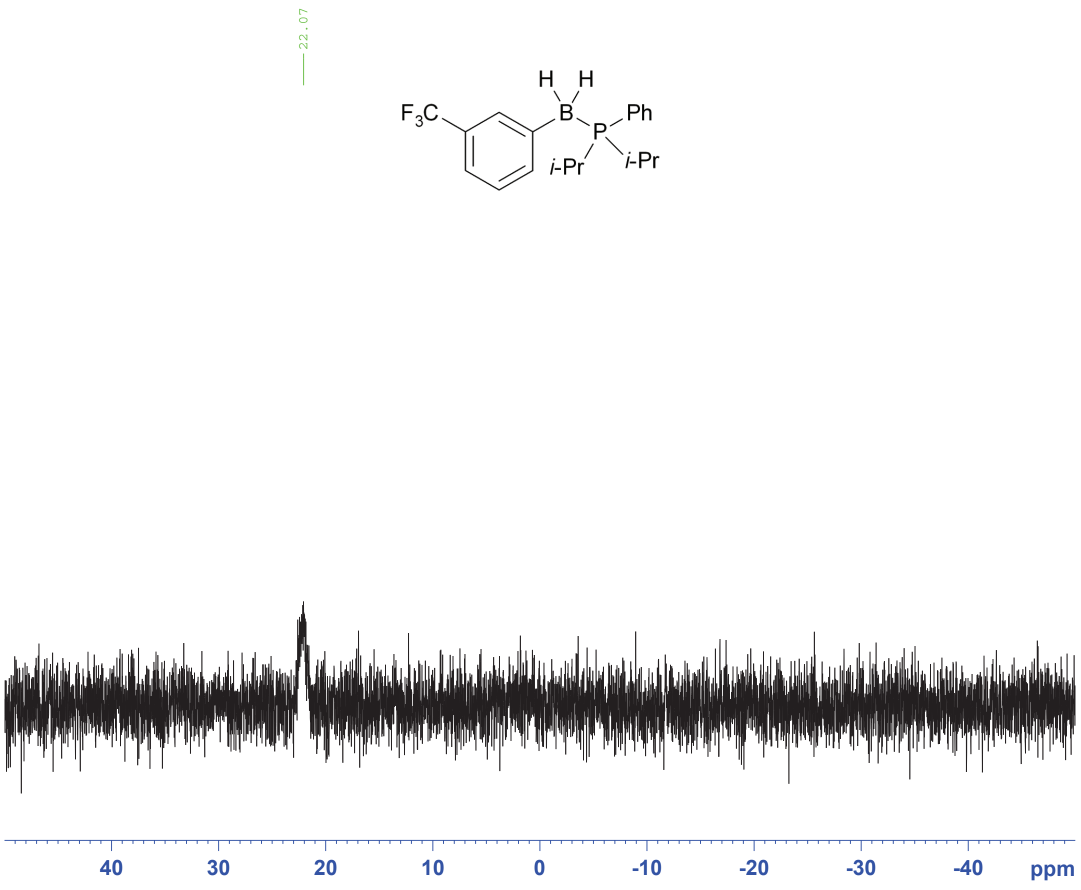


```
NAME            Jun12-2022
EXPNO                    7
PROCNO                   1
Date_             20220613
Time                  0.33
INSTRUM              av400
PROBHD     5 mm PABBO BB-
PULPROG               zg30
TD                   65536
SOLVENT               DMSO
NS                     128
DS                       4
SWH              64102.563 Hz
FIDRES            0.978127 Hz
AQ               0.5112308 sec
RG                     203
DW                   7.800 usec
DE                    6.50 usec
TE                   297.0 K
D1              2.00000000 sec
TD0                      1

======== CHANNEL f1 ========
NUC1                   31P
P1                   10.00 usec
PL1                  -0.70 dB
PL1W           30.10233498 W
SFO1           161.9674942 MHz
SI                   32768
SF             161.9754962 MHz
WDW                     EM
SSB                      0
LB                    1.00 Hz
GB                       0
PC                    1.40
```

B-3-(Trifluoromethyl)phenyl tricyclopropylphosphine–borane (22)

3CF3PhBH2P(cyclopropyl)3

IBB-nmr Analysis

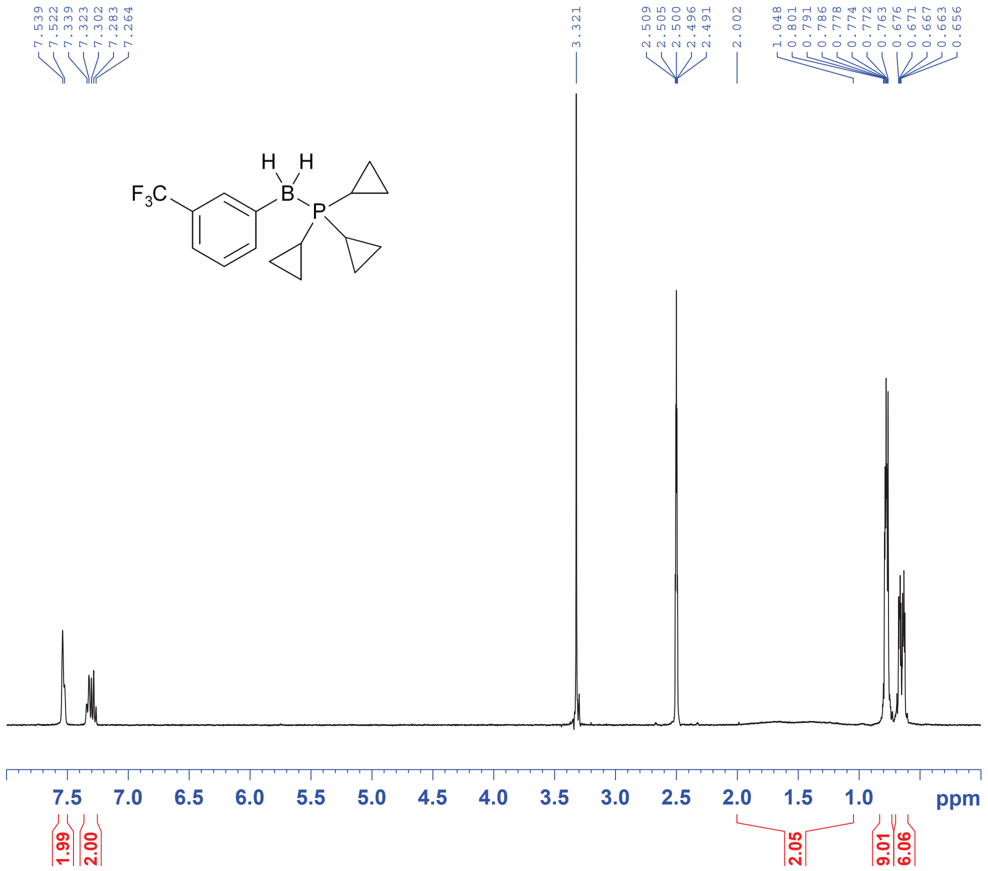

NAME	July11-2022
EXPNO	1
PROCNO	1
Date_	20220711
Time	21.22
INSTRUM	av400
PROBHD	5 mm PABBO BB-
PULPROG	zg30
TD	65536
SOLVENT	DMSO
NS	4
DS	2
SWH	8223.685 Hz
FIDRES	0.125483 Hz
AQ	3.9846387 sec
RG	203
DW	60.800 usec
DE	6.50 usec
TE	297.4 K
D1	1.00000000 sec
TD0	1
======== CHANNEL f1 ========	
NUC1	1H
P1	14.00 usec
PL1	-1.80 dB
PL1W	14.82738590 W
SFO1	400.1324710 MHz
SI	32768
SF	400.1300029 MHz
WDW	EM
SSB	0
LB	0.30 Hz
GB	0
PC	1.00

3CF3PhBH2P(cyclopropyl)3

IBB-nmr Analysis

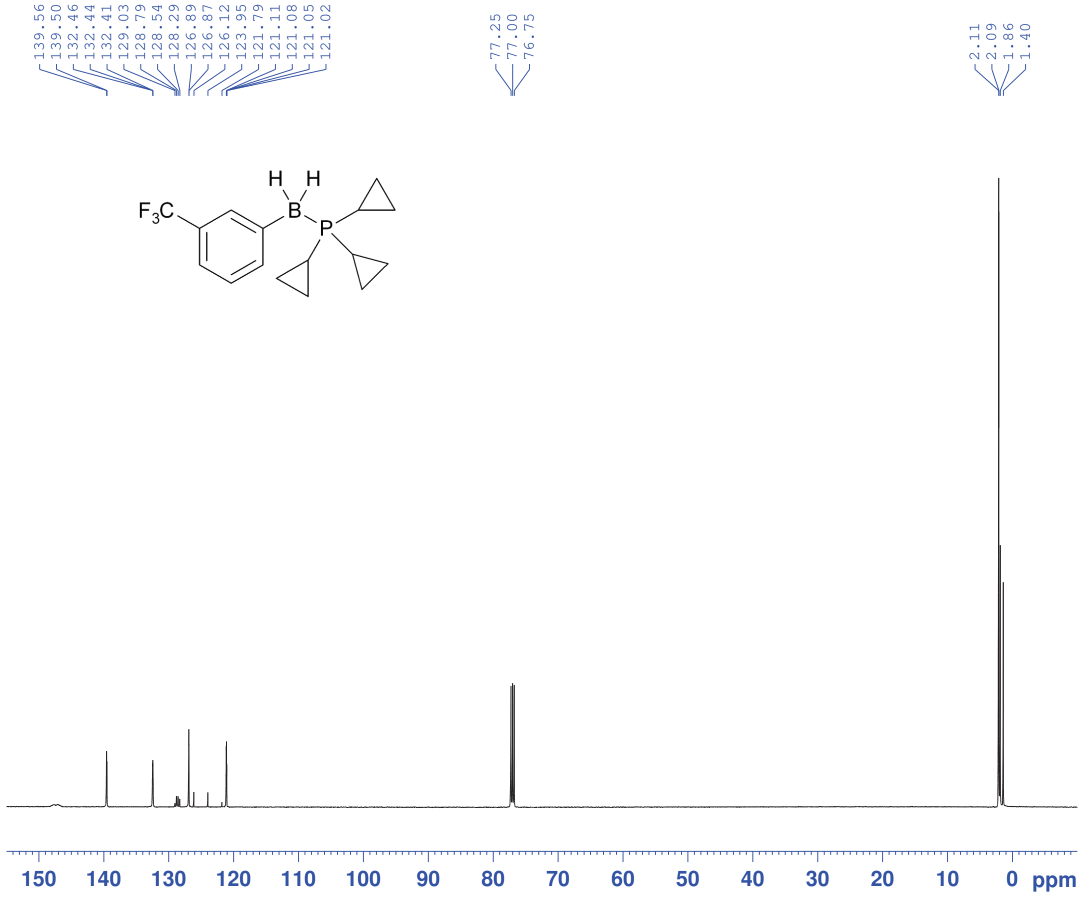

NAME	Mar11-2024
EXPNO	3
PROCNO	1
Date_	20240311
Time	15.20
INSTRUM	av500
PROBHD	5 mm CPDCH 13C
PULPROG	zgpg30
TD	65536
SOLVENT	CDCl3
NS	64
DS	4
SWH	30030.029 Hz
FIDRES	0.458222 Hz
AQ	1.0912410 sec
RG	40.3
DW	16.650 usec
DE	20.00 usec
TE	298.1 K
D1	2.00000000 sec
D11	0.03000000 sec
TD0	1
======== CHANNEL f1 ========	
NUC1	13C
P1	10.00 usec
PL1	-5.00 dB
PL1W	14.29790783 W
SFO1	125.7703643 MHz
======== CHANNEL f2 ========	
CPDPRG2	waltz16
NUC2	1H
PCPD2	80.00 usec
PL2	-4.00 dB
PL12	14.06 dB
PL13	17.00 dB
PL2W	6.30957365 W
PL12W	0.09862794 W
PL13W	0.05011872 W
SFO2	500.1320005 MHz
SI	32768
SF	125.7577974 MHz
WDW	EM
SSB	0
LB	1.00 Hz
GB	0
PC	0.30

3CF3PhBH2P(cyclopropyl)3

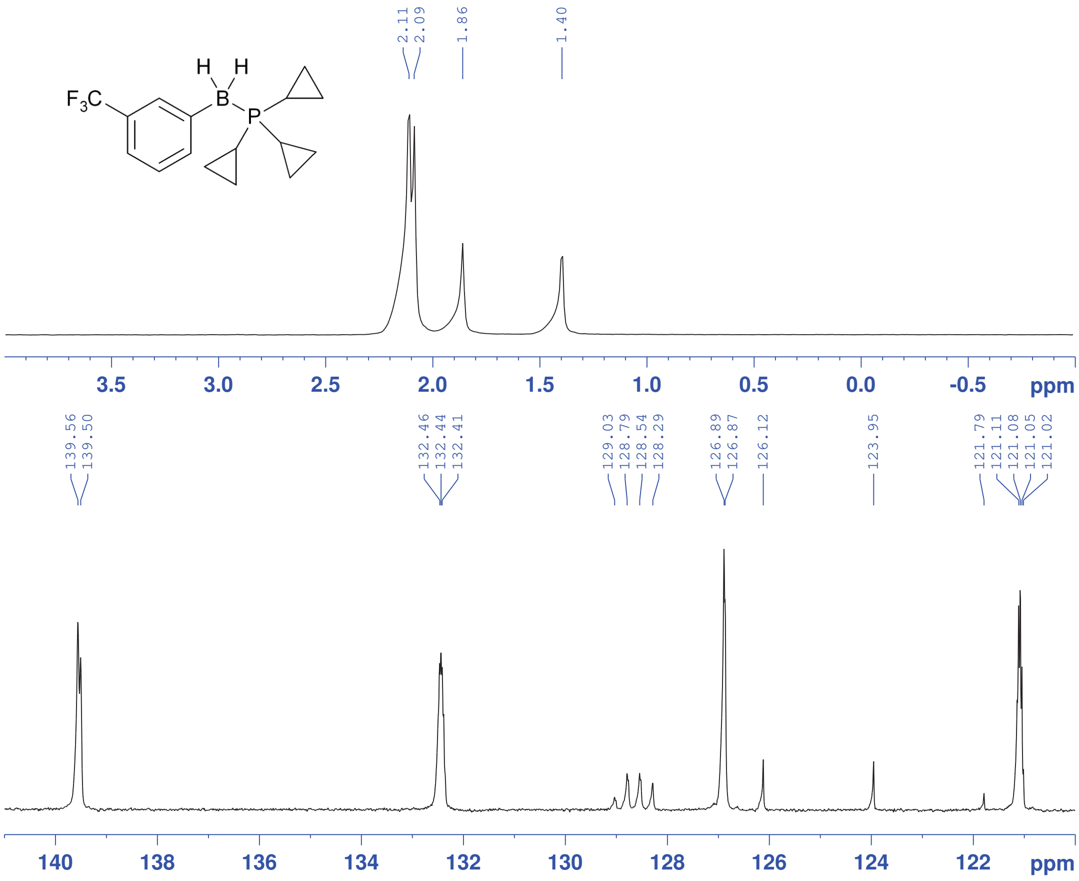

NAME Mar11-2024
EXPNO 3
PROCNO 1
Date_ 20240311
Time 15.20
INSTRUM av500
PROBHD 5 mm CPDCH 13C
PULPROG zgpg30
TD 65536
SOLVENT CDCl3
NS 64
DS 4
SWH 30030.029 Hz
FIDRES 0.458222 Hz
AQ 1.0912410 sec
RG 40.3
DW 16.650 usec
DE 20.00 usec
TE 298.1 K
D1 2.00000000 sec
D11 0.03000000 sec
TD0 1

======== CHANNEL f1 ========
NUC1 13C
P1 10.00 usec
PL1 -5.00 dB
PL1W 14.29790783 W
SFO1 125.7703643 MHz

======== CHANNEL f2 ========
CPDPRG2 waltz16
NUC2 1H
PCPD2 80.00 usec
PL2 -4.00 dB
PL12 14.06 dB
PL13 17.00 dB
PL2W 6.30957365 W
PL12W 0.09862794 W
PL13W 0.05011872 W
SFO2 500.1320005 MHz
SI 32768
SF 125.7577974 MHz
WDW EM
SSB 0
LB 1.00 Hz
GB 0
PC 0.30

3CF3PhBH2P(cyclopropyl)3

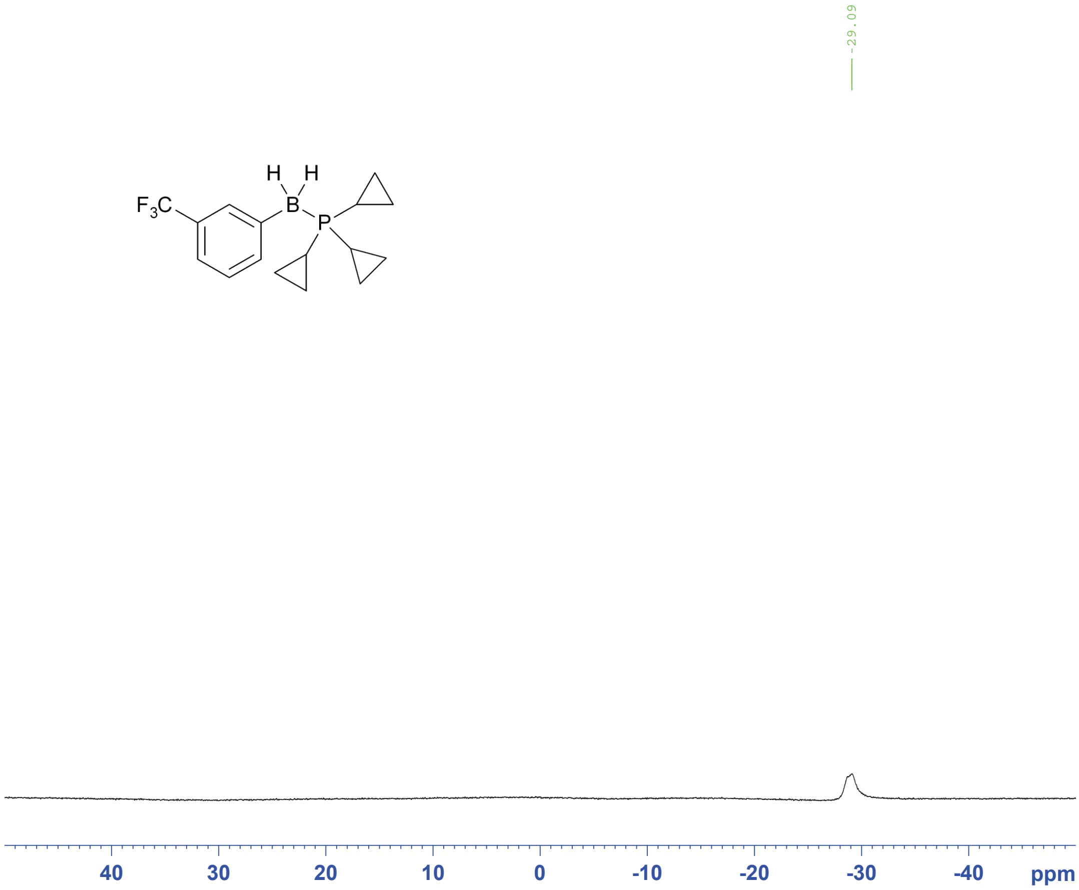

NAME July11-2022
EXPNO 5
PROCNO 1
Date_ 20220711
Time 23.00
INSTRUM av400
PROBHD 5 mm PABBO BB-
PULPROG zgig
TD 32768
SOLVENT DMSO
NS 128
DS 4
SWH 20000.000 Hz
FIDRES 0.610352 Hz
AQ 0.8192500 sec
RG 203
DW 25.000 usec
DE 20.00 usec
TE 297.8 K
D1 1.00000000 sec
D11 0.03000000 sec
TD0 1

======== CHANNEL f1 ========
NUC1 11B
P1 6.30 usec
PL1 -2.00 dB
SFO1 128.3776050 MHz

======== CHANNEL f2 ========
CPDPRG2 waltz16
NUC2 1H
PCPD2 80.00 usec
PL2 -1.70 dB
PL12 13.80 dB
PL2W 14.48987389 W
PL12W 0.40838012 W
SFO2 400.1316005 MHz
SI 32768
SF 128.3776183 MHz
WDW EM
SSB 0
LB 2.00 Hz
GB 0
PC 1.40

3CF3PhBH2P(cyclopropyl)3

IBB-nmr Analysis

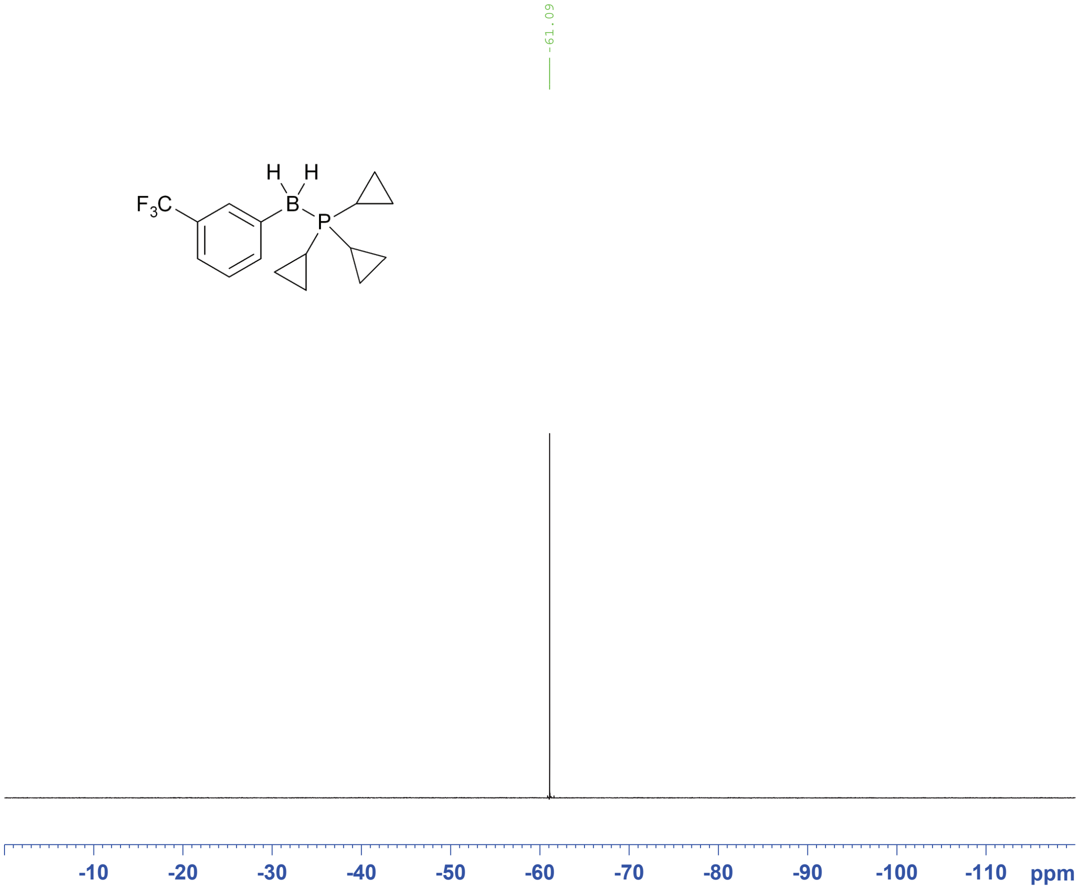

NAME	July11-2022
EXPNO	2
PROCNO	1
Date_	20220711
Time	21.26
INSTRUM	av400
PROBHD	5 mm PABBO BB-
PULPROG	zgfhigqn
TD	131072
SOLVENT	DMSO
NS	8
DS	4
SWH	89285.711 Hz
FIDRES	0.681196 Hz
AQ	0.7340532 sec
RG	203
DW	5.600 usec
DE	6.50 usec
TE	297.5 K
D1	1.00000000 sec
D11	0.03000000 sec
D12	0.00002000 sec
TD0	1
======== CHANNEL f1 ========	
NUC1	19F
P1	19.50 usec
PL1	-4.00 dB
PL1W	16.40489006 W
SFO1	376.4607164 MHz
======== CHANNEL f2 ========	
CPDPRG2	waltz16
NUC2	1H
PCPD2	112.00 usec
PL2	-4.00 dB
PL12	18.00 dB
PL2W	24.60733604 W
PL12W	0.15526180 W
SFO2	400.1316005 MHz
SI	65536
SF	376.4984617 MHz
WDW	EM
SSB	0
LB	0.30 Hz
GB	0
PC	1.00

3CF3PhBH2P(cyclopropyl)3

IBB-nmr Analysis

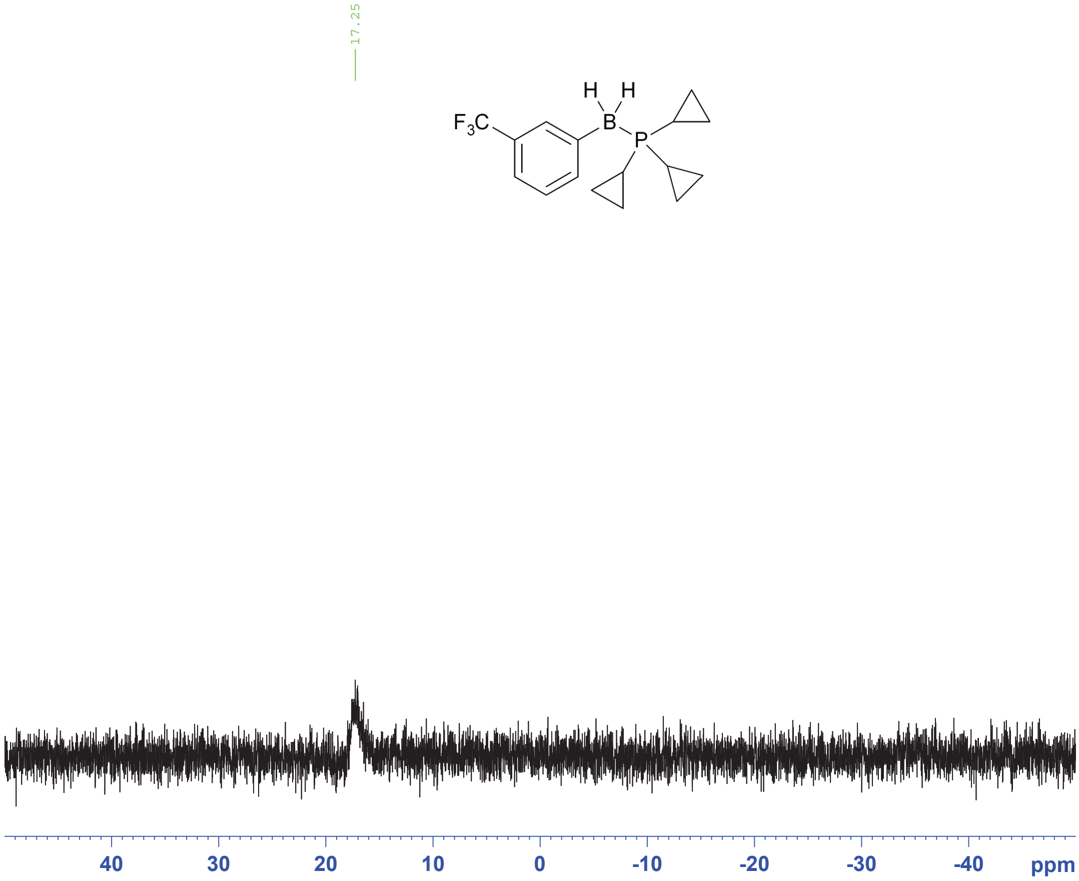

NAME	July11-2022
EXPNO	4
PROCNO	1
Date_	20220711
Time	22.52
INSTRUM	av400
PROBHD	5 mm PABBO BB-
PULPROG	zg30
TD	65536
SOLVENT	DMSO
NS	16
DS	4
SWH	64102.563 Hz
FIDRES	0.978127 Hz
AQ	0.5112308 sec
RG	203
DW	7.800 usec
DE	6.50 usec
TE	297.3 K
D1	2.00000000 sec
TD0	1
======== CHANNEL f1 ========	
NUC1	31P
P1	10.00 usec
PL1	-0.70 dB
PL1W	30.10233498 W
SFO1	161.9674942 MHz
SI	32768
SF	161.9754962 MHz
WDW	EM
SSB	0
LB	1.00 Hz
GB	0
PC	1.40

3CF3PhBH2P(n-Bu)3

B-3-(Trifluoromethyl)phenyl tri-n-butylphosphine–borane (23)

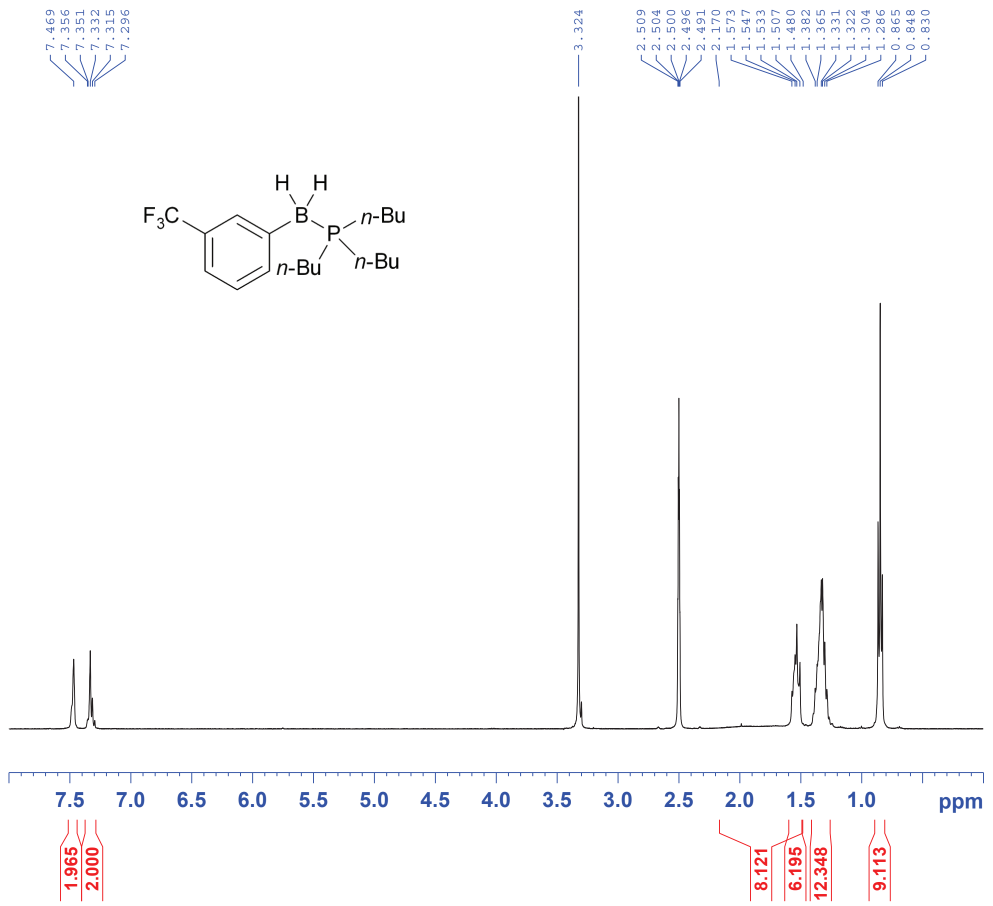

IBB-nmr Analysis

NAME	May23-2022
EXPNO	1
PROCNO	1
Date_	20220523
Time	21.48
INSTRUM	av400
PROBHD	5 mm PABBO BB-
PULPROG	zg30
TD	65536
SOLVENT	DMSO
NS	16
DS	2
SWH	8223.685 Hz
FIDRES	0.125483 Hz
AQ	3.9846387 sec
RG	203
DW	60.800 usec
DE	6.50 usec
TE	296.9 K
D1	1.00000000 sec
TD0	1
======== CHANNEL f1 ========	
NUC1	1H
P1	14.00 usec
PL1	-1.80 dB
PL1W	14.82738590 W
SFO1	400.1324710 MHz
SI	32768
SF	400.1300029 MHz
WDW	EM
SSB	0
LB	0.30 Hz
GB	0
PC	1.00

3CF3PhBH2P(n-Bu)3

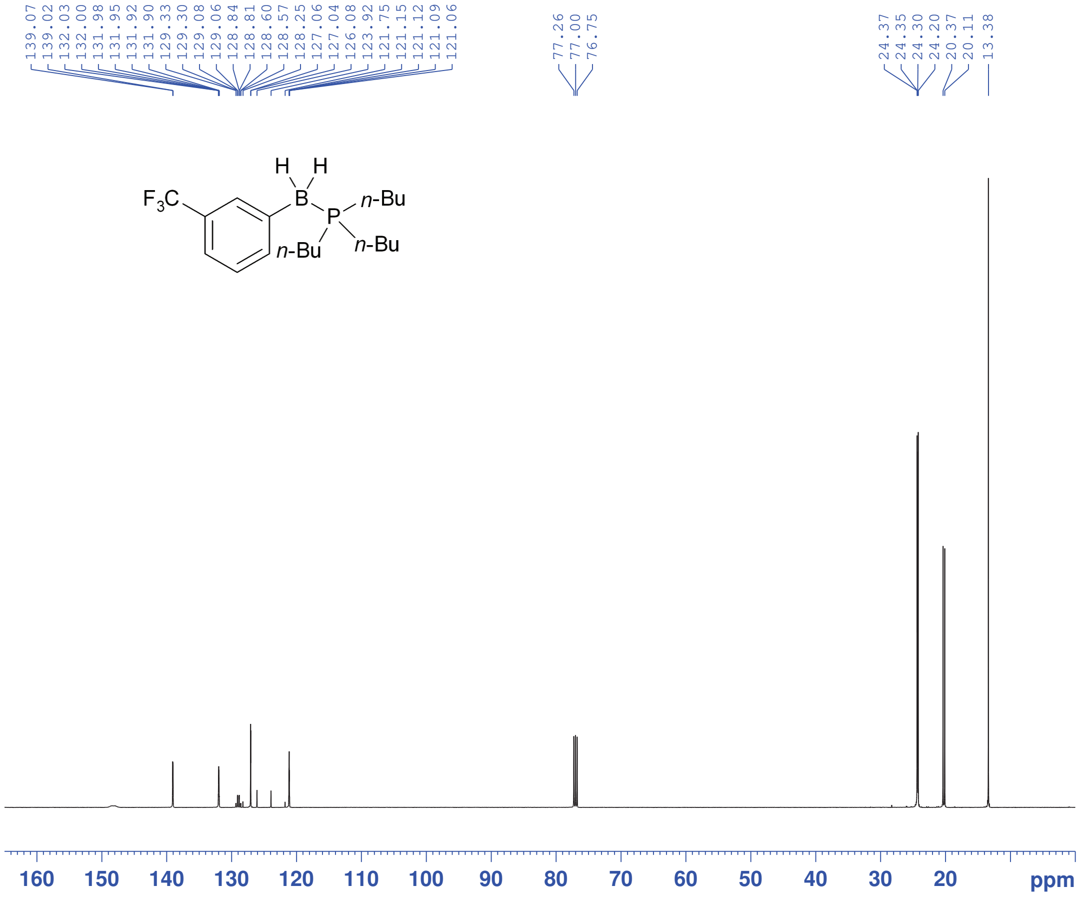

IBB-nmr Analysis

NAME	Mar08-2024
EXPNO	10
PROCNO	1
Date_	20240308
Time	5.46
INSTRUM	av500
PROBHD	5 mm CPDCH 13C
PULPROG	zgpg30
TD	65536
SOLVENT	CDCl3
NS	64
DS	4
SWH	30030.029 Hz
FIDRES	0.458222 Hz
AQ	1.0912410 sec
RG	90.5
DW	16.650 usec
DE	20.00 usec
TE	298.0 K
D1	2.00000000 sec
D11	0.03000000 sec
TD0	1
======== CHANNEL f1 ========	
NUC1	13C
P1	10.00 usec
PL1	-5.00 dB
PL1W	14.29790783 W
SFO1	125.7703643 MHz
======== CHANNEL f2 ========	
CPDPRG2	waltz16
NUC2	1H
PCPD2	80.00 usec
PL2	-4.00 dB
PL12	14.06 dB
PL13	17.00 dB
PL2W	6.30957365 W
PL12W	0.09862794 W
PL13W	0.05011872 W
SFO2	500.1320005 MHz
SI	32768
SF	125.7578001 MHz
WDW	EM
SSB	0
LB	1.00 Hz
GB	0
PC	0.30

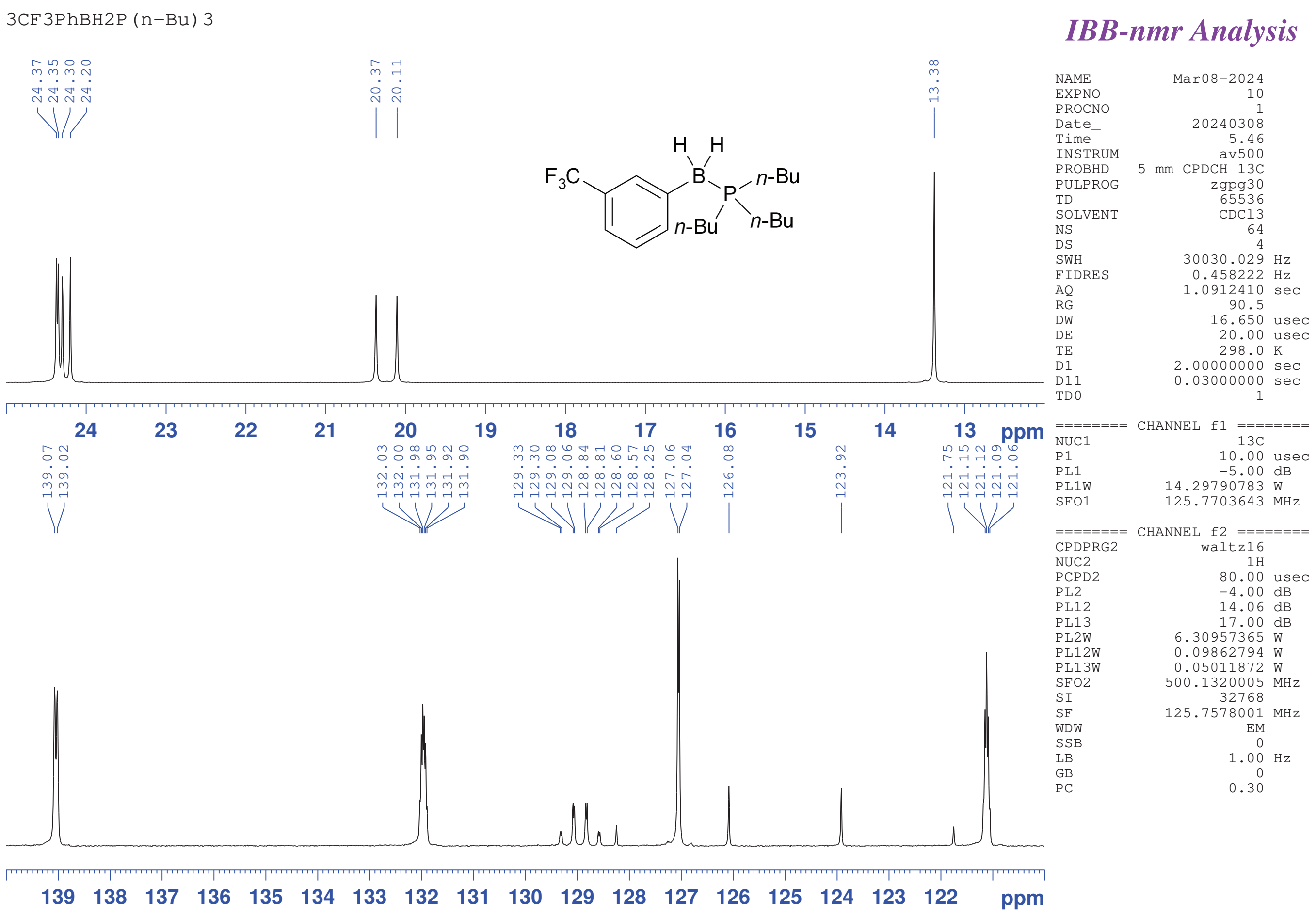

3CF3PhBH2P(n-Bu)3
IBB-nmr Analysis
24.37 24.35 24.30 24.20 20.37 20.11 13.38
139.07 139.02 132.03 132.00 131.98 131.95 131.92 131.90 129.33 129.30 129.08 129.06 128.84 128.81 128.60 128.57 128.25 127.06 127.04 126.08 123.92 121.75 121.15 121.12 121.09 121.06
F3C H H B P n-Bu n-Bu n-Bu
NAME Mar08-2024
EXPNO 10
PROCNO 1
Date_ 20240308
Time 5.46
INSTRUM av500
PROBHD 5 mm CPDCH 13C
PULPROG zgpg30
TD 65536
SOLVENT CDCl3
NS 64
DS 4
SWH 30030.029 Hz
FIDRES 0.458222 Hz
AQ 1.0912410 sec
RG 90.5
DW 16.650 usec
DE 20.00 usec
TE 298.0 K
D1 2.00000000 sec
D11 0.03000000 sec
TD0 1
======== CHANNEL f1 ========
NUC1 13C
P1 10.00 usec
PL1 -5.00 dB
PL1W 14.29790783 W
SFO1 125.7703643 MHz
======== CHANNEL f2 ========
CPDPRG2 waltz16
NUC2 1H
PCPD2 80.00 usec
PL2 -4.00 dB
PL12 14.06 dB
PL13 17.00 dB
PL2W 6.30957365 W
PL12W 0.09862794 W
PL13W 0.05011872 W
SFO2 500.1320005 MHz
SI 32768
SF 125.7578001 MHz
WDW EM
SSB 0
LB 1.00 Hz
GB 0
PC 0.30
24 23 22 21 20 19 18 17 16 15 14 13 ppm
139 138 137 136 135 134 133 132 131 130 129 128 127 126 125 124 123 122 ppm

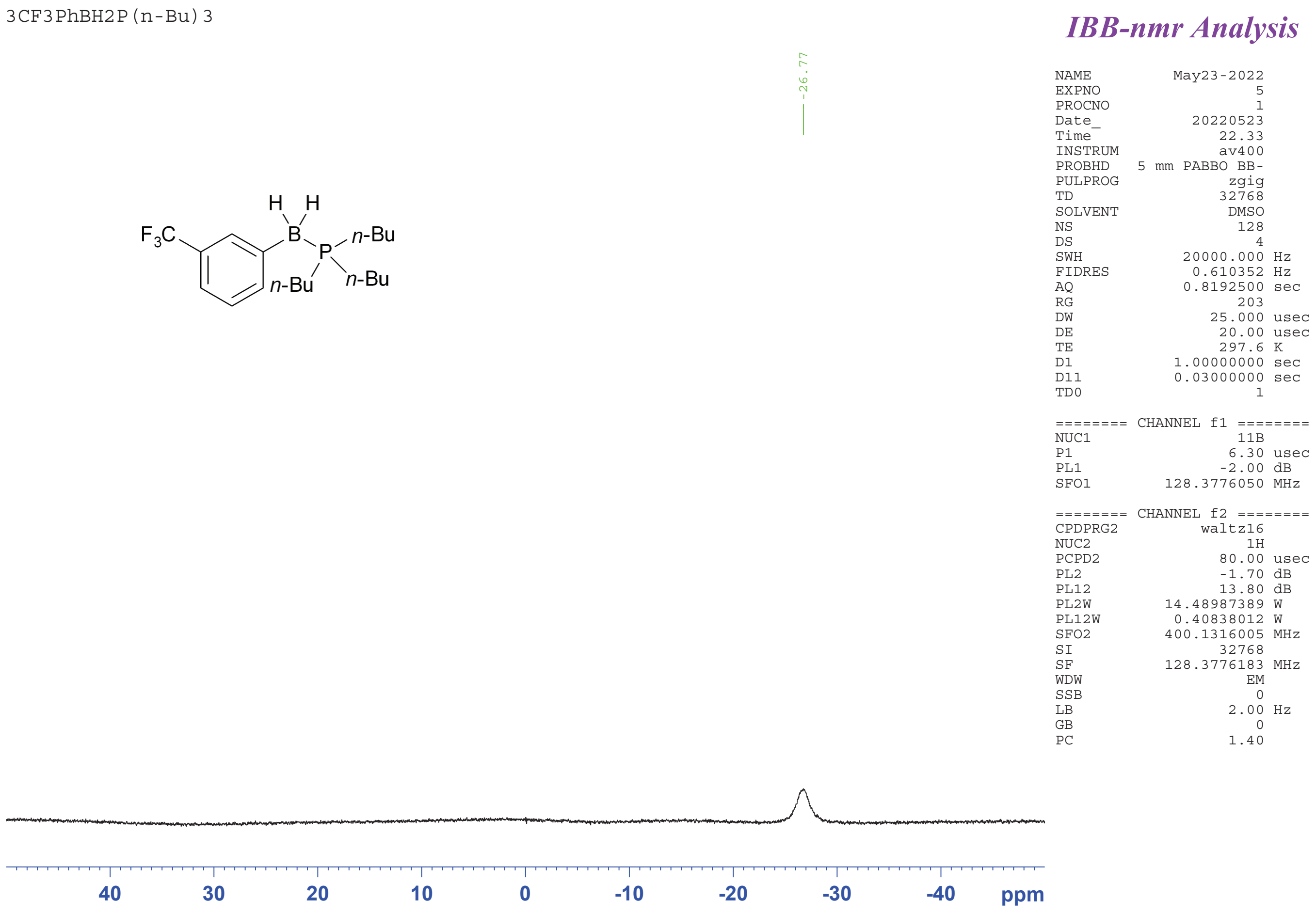

3CF3PhBH2P(n-Bu)3
IBB-nmr Analysis
-26.77
F3C H H B P n-Bu n-Bu n-Bu
NAME May23-2022
EXPNO 5
PROCNO 1
Date_ 20220523
Time 22.33
INSTRUM av400
PROBHD 5 mm PABBO BB-
PULPROG zgig
TD 32768
SOLVENT DMSO
NS 128
DS 4
SWH 20000.000 Hz
FIDRES 0.610352 Hz
AQ 0.8192500 sec
RG 203
DW 25.000 usec
DE 20.00 usec
TE 297.6 K
D1 1.00000000 sec
D11 0.03000000 sec
TD0 1
======== CHANNEL f1 ========
NUC1 11B
P1 6.30 usec
PL1 -2.00 dB
SFO1 128.3776050 MHz
======== CHANNEL f2 ========
CPDPRG2 waltz16
NUC2 1H
PCPD2 80.00 usec
PL2 -1.70 dB
PL12 13.80 dB
PL2W 14.48987389 W
PL12W 0.40838012 W
SFO2 400.1316005 MHz
SI 32768
SF 128.3776183 MHz
WDW EM
SSB 0
LB 2.00 Hz
GB 0
PC 1.40
40 30 20 10 0 -10 -20 -30 -40 ppm

3CF3PhBH2P(n-Bu)3

IBB-nmr Analysis

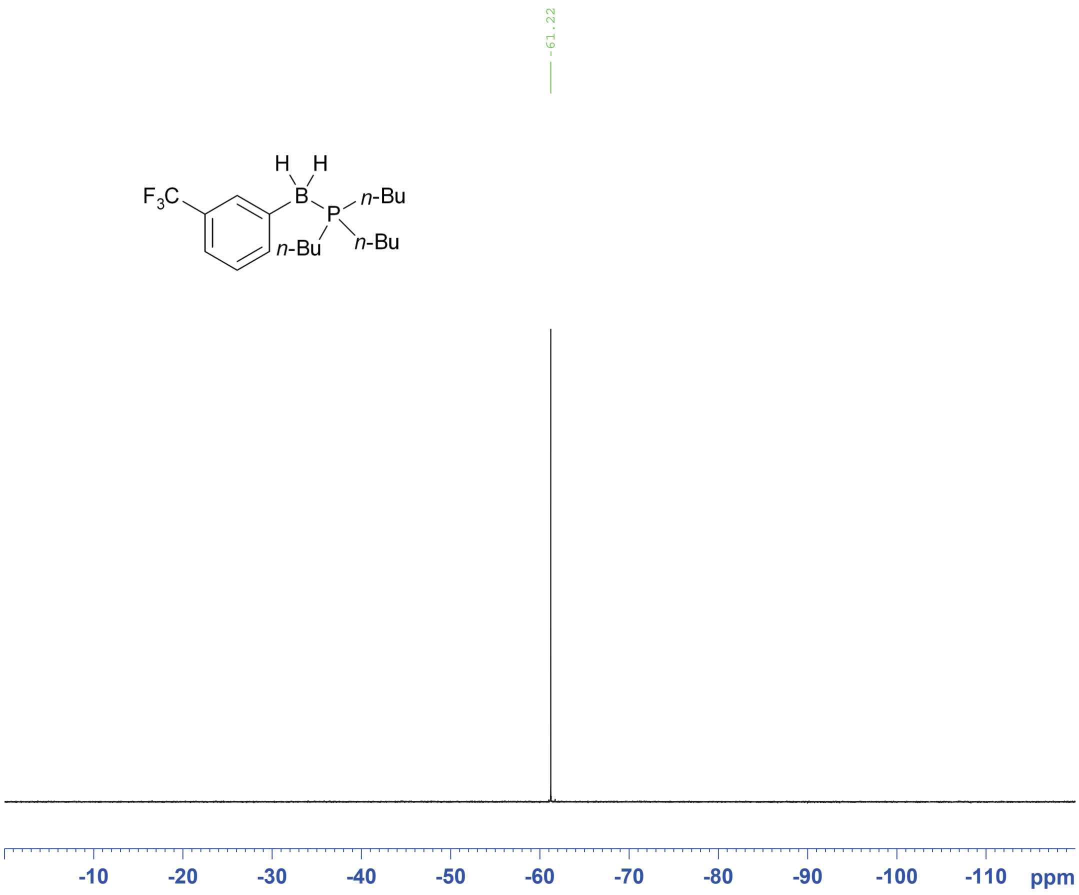

NAME	May23-2022
EXPNO	2
PROCNO	1
Date_	20220523
Time	21.52
INSTRUM	av400
PROBHD	5 mm PABBO BB-
PULPROG	zgfhigqn
TD	131072
SOLVENT	DMSO
NS	8
DS	4
SWH	89285.711 Hz
FIDRES	0.681196 Hz
AQ	0.7340532 sec
RG	203
DW	5.600 usec
DE	6.50 usec
TE	297.0 K
D1	1.00000000 sec
D11	0.03000000 sec
D12	0.00002000 sec
TD0	1

======== CHANNEL f1 ========

NUC1	19F
P1	19.50 usec
PL1	-4.00 dB
PL1W	16.40489006 W
SFO1	376.4607164 MHz

======== CHANNEL f2 ========

CPDPRG2	waltz16
NUC2	1H
PCPD2	112.00 usec
PL2	-4.00 dB
PL12	18.00 dB
PL2W	24.60733604 W
PL12W	0.15526180 W
SFO2	400.1316005 MHz
SI	65536
SF	376.4984617 MHz
WDW	EM
SSB	0
LB	0.30 Hz
GB	0
PC	1.00

3CF3PhBH2P(n-Bu)3

IBB-nmr Analysis

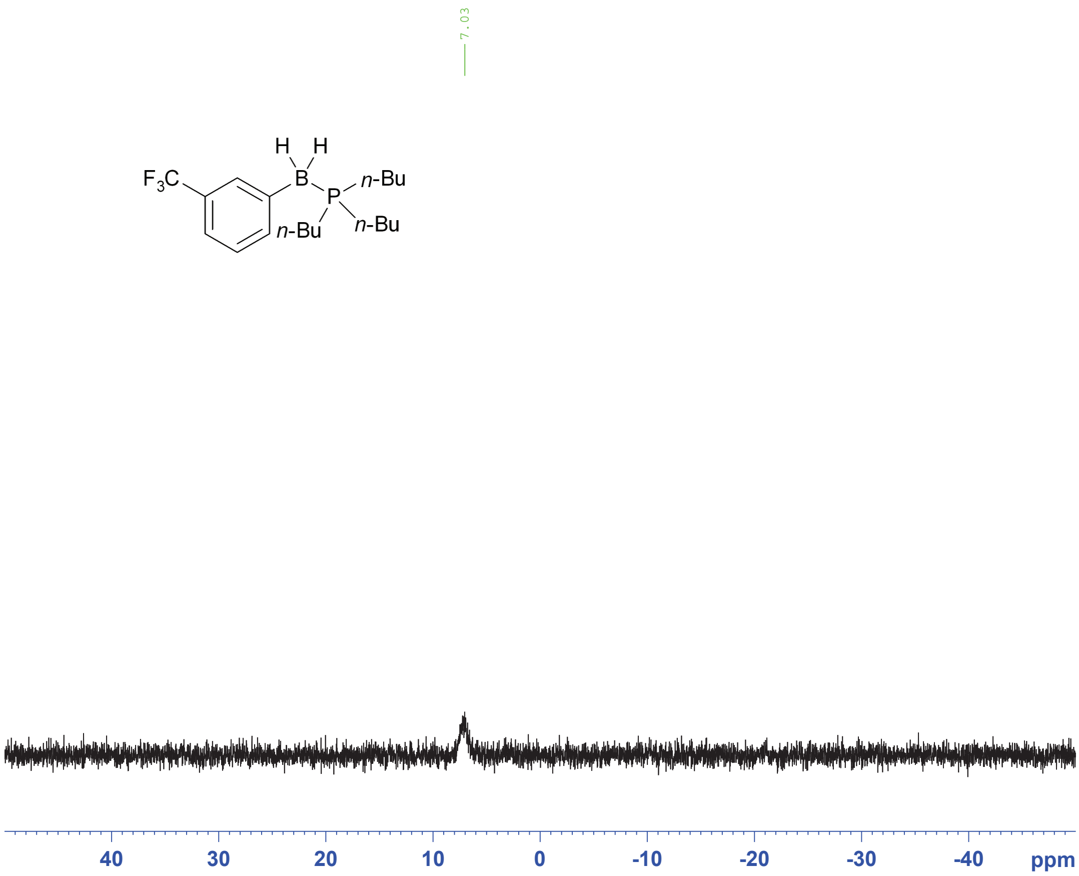

NAME	May23-2022
EXPNO	4
PROCNO	1
Date_	20220523
Time	22.26
INSTRUM	av400
PROBHD	5 mm PABBO BB-
PULPROG	zg30
TD	65536
SOLVENT	DMSO
NS	16
DS	4
SWH	64102.563 Hz
FIDRES	0.978127 Hz
AQ	0.5112308 sec
RG	203
DW	7.800 usec
DE	6.50 usec
TE	296.9 K
D1	2.00000000 sec
TD0	1

======== CHANNEL f1 ========

NUC1	31P
P1	10.00 usec
PL1	-0.70 dB
PL1W	30.10233498 W
SFO1	161.9674942 MHz
SI	32768
SF	161.9754962 MHz
WDW	EM
SSB	0
LB	1.00 Hz
GB	0
PC	1.40

B-3-(Trifluoromethyl)phenyl tricyclopentylphosphine–borane (24)

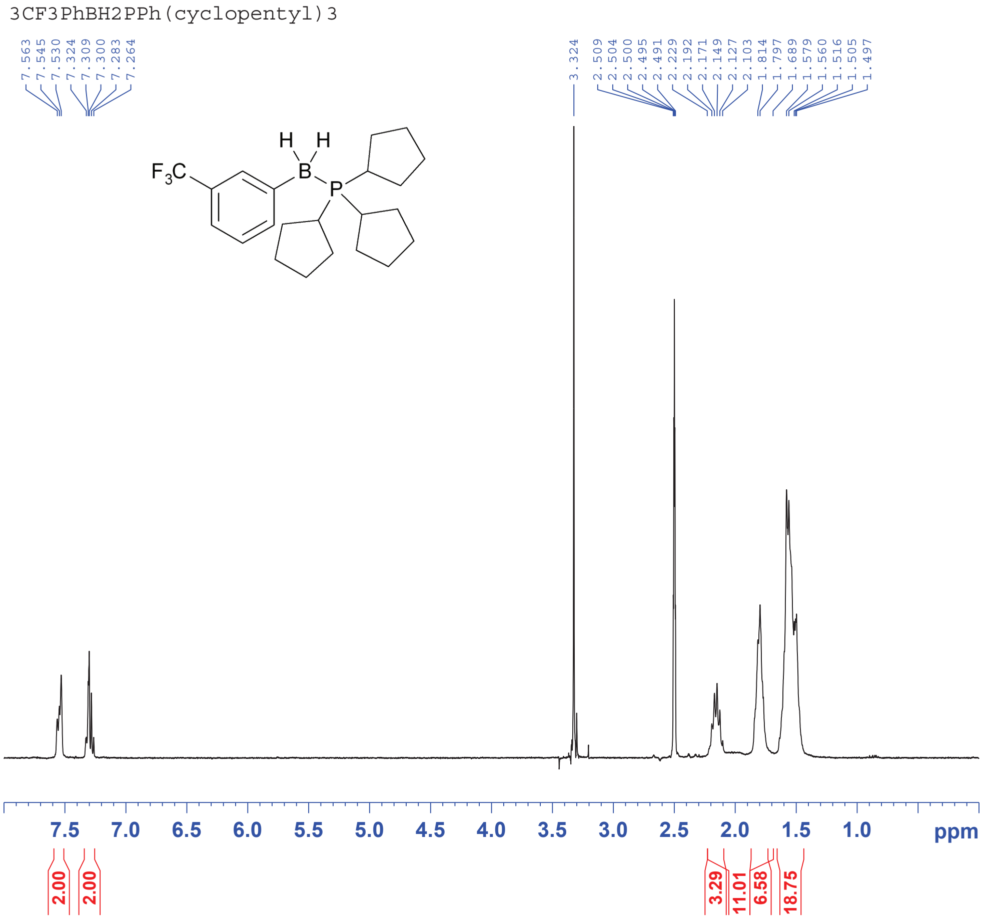

IBB-nmr Analysis

NAME	Apr23-2022
EXPNO	1
PROCNO	1
Date_	20220423
Time	12.06
INSTRUM	av400
PROBHD	5 mm PABBO BB-
PULPROG	zg30
TD	65536
SOLVENT	DMSO
NS	4
DS	2
SWH	8223.685 Hz
FIDRES	0.125483 Hz
AQ	3.9846387 sec
RG	203
DW	60.800 usec
DE	6.50 usec
TE	296.7 K
D1	1.00000000 sec
TD0	1

======== CHANNEL f1 ========

NUC1	1H
P1	14.00 usec
PL1	-1.80 dB
PL1W	14.82738590 W
SFO1	400.1324710 MHz
SI	32768
SF	400.1300031 MHz
WDW	EM
SSB	0
LB	0.30 Hz
GB	0
PC	1.00

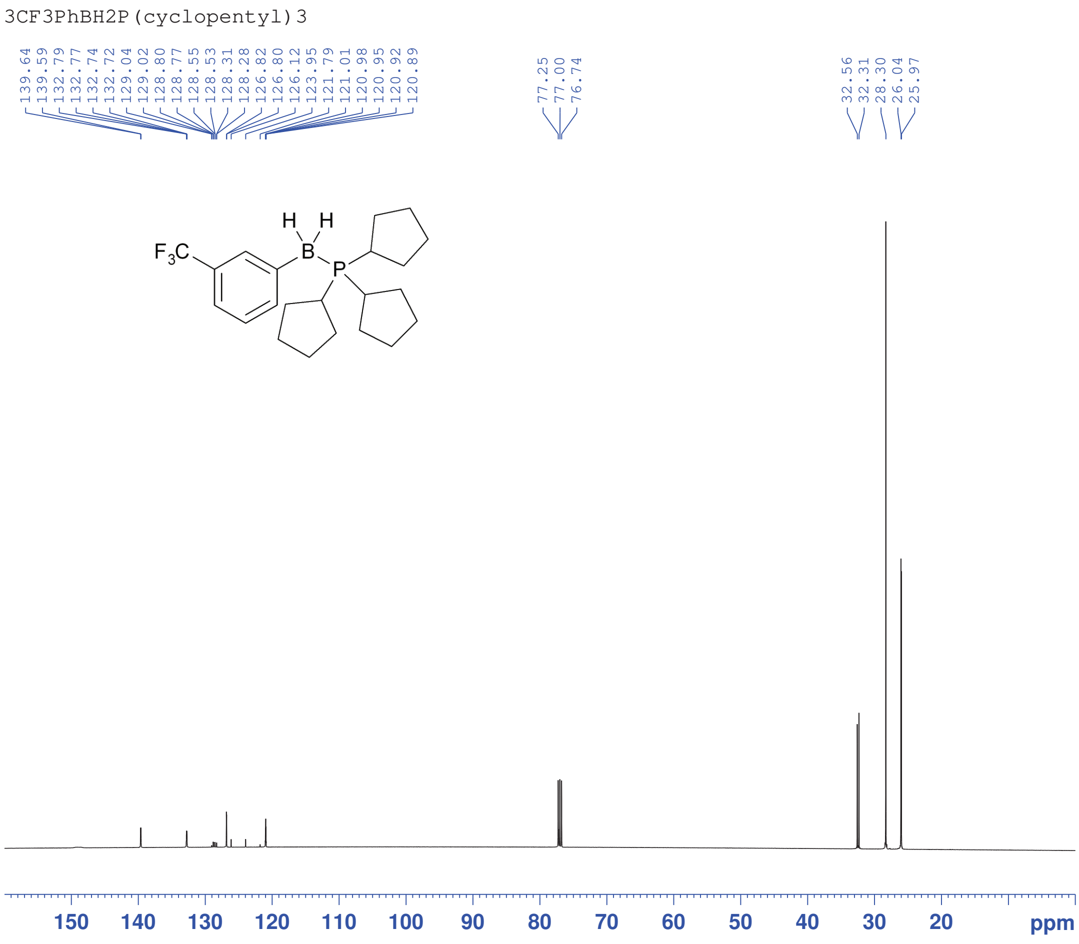

IBB-nmr Analysis

NAME	Mar08-2024
EXPNO	2
PROCNO	1
Date_	20240308
Time	0.47
INSTRUM	av500
PROBHD	5 mm CPDCH 13C
PULPROG	zgpg30
TD	65536
SOLVENT	CDCl3
NS	64
DS	4
SWH	30030.029 Hz
FIDRES	0.458222 Hz
AQ	1.0912410 sec
RG	90.5
DW	16.650 usec
DE	20.00 usec
TE	298.0 K
D1	2.00000000 sec
D11	0.03000000 sec
TD0	1

======== CHANNEL f1 ========

NUC1	13C
P1	10.00 usec
PL1	-5.00 dB
PL1W	14.29790783 W
SFO1	125.7703643 MHz

======== CHANNEL f2 ========

CPDPRG2	waltz16
NUC2	1H
PCPD2	80.00 usec
PL2	-4.00 dB
PL12	14.06 dB
PL13	17.00 dB
PL2W	6.30957365 W
PL12W	0.09862794 W
PL13W	0.05011872 W
SFO2	500.1320005 MHz
SI	32768
SF	125.7577985 MHz
WDW	EM
SSB	0
LB	1.00 Hz
GB	0
PC	0.30

3CF3PhBH2P(cyclopentyl)3

IBB-nmr Analysis

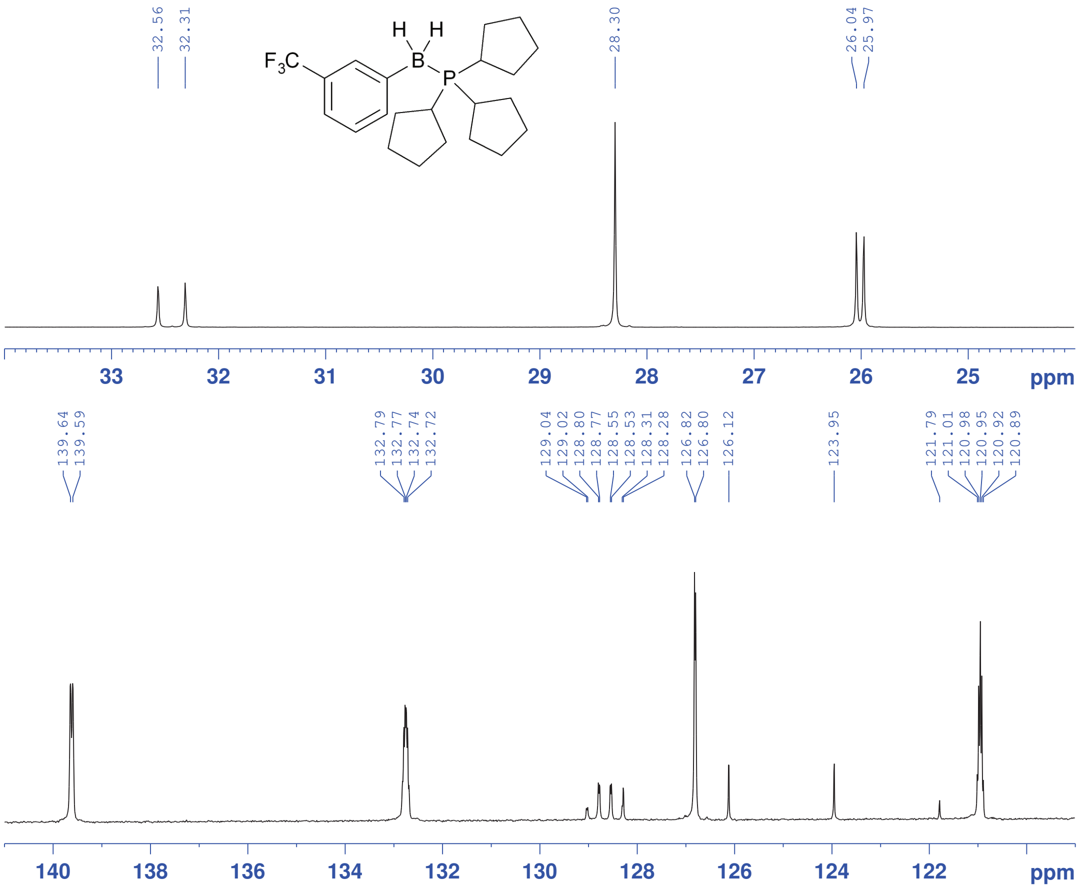


```
NAME           Mar08-2024
EXPNO                   2
PROCNO                  1
Date_            20240308
Time                 0.47
INSTRUM             av500
PROBHD   5 mm CPDCH 13C
PULPROG           zgpg30
TD                  65536
SOLVENT             CDCl3
NS                     64
DS                      4
SWH             30030.029 Hz
FIDRES           0.458222 Hz
AQ              1.0912410 sec
RG                   90.5
DW                 16.650 usec
DE                  20.00 usec
TE                  298.0 K
D1             2.00000000 sec
D11            0.03000000 sec
TD0                     1

======== CHANNEL f1 ========
NUC1                  13C
P1                  10.00 usec
PL1                 -5.00 dB
PL1W          14.29790783 W
SFO1         125.7703643 MHz

======== CHANNEL f2 ========
CPDPRG2           waltz16
NUC2                   1H
PCPD2               80.00 usec
PL2                 -4.00 dB
PL12                14.06 dB
PL13                17.00 dB
PL2W           6.30957365 W
PL12W          0.09862794 W
PL13W          0.05011872 W
SFO2         500.1320005 MHz
SI                  32768
SF           125.7577985 MHz
WDW                    EM
SSB                     0
LB                   1.00 Hz
GB                      0
PC                   0.30
```

3CF3PhBH2PPh(cyclopentyl)3

IBB-nmr Analysis

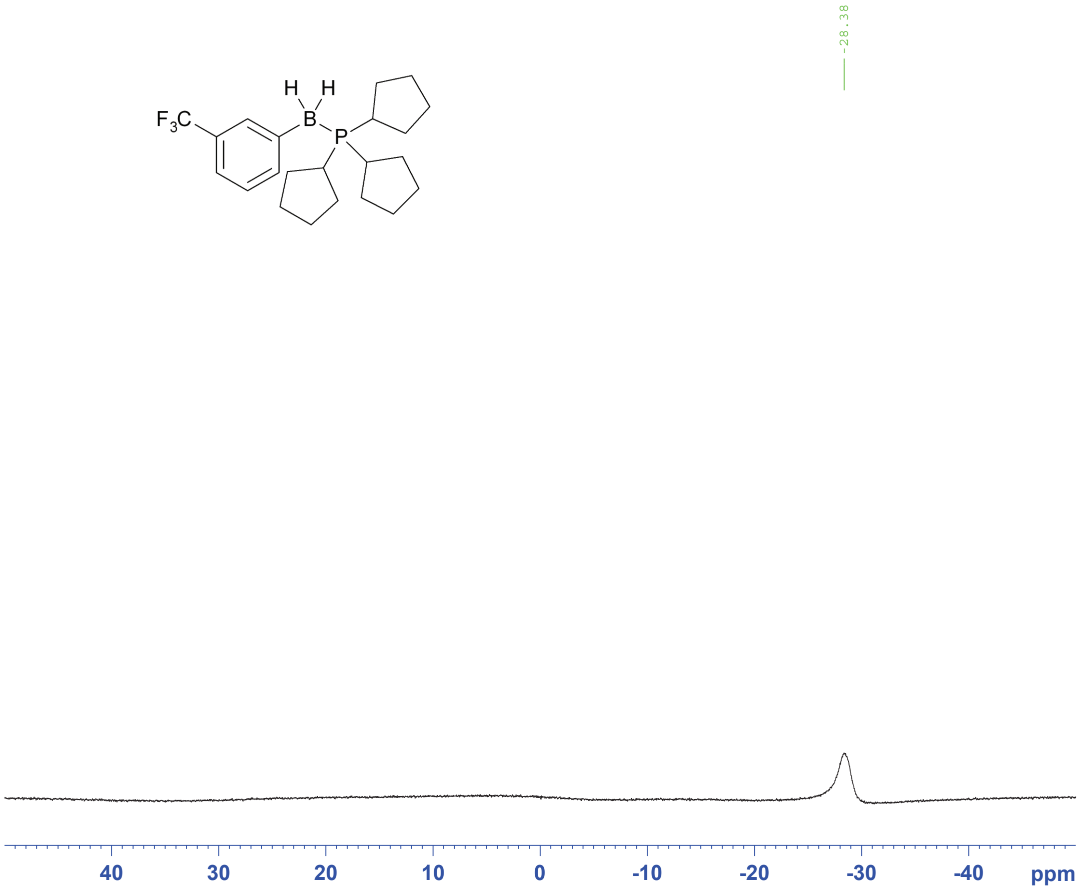


```
NAME           Apr23-2022
EXPNO                   4
PROCNO                  1
Date_            20220423
Time                12.30
INSTRUM             av400
PROBHD   5 mm PABBO BB-
PULPROG              zgig
TD                  32768
SOLVENT              DMSO
NS                    256
DS                      4
SWH             20000.000 Hz
FIDRES           0.610352 Hz
AQ              0.8192500 sec
RG                    203
DW                 25.000 usec
DE                  20.00 usec
TE                  297.5 K
D1             1.00000000 sec
D11            0.03000000 sec
TD0                     1

======== CHANNEL f1 ========
NUC1                  11B
P1                   6.30 usec
PL1                 -2.00 dB
SFO1         128.3776050 MHz

======== CHANNEL f2 ========
CPDPRG2           waltz16
NUC2                   1H
PCPD2               80.00 usec
PL2                 -1.70 dB
PL12                13.80 dB
PL2W          14.48987389 W
PL12W          0.40838012 W
SFO2         400.1316005 MHz
SI                  32768
SF           128.3776183 MHz
WDW                    EM
SSB                     0
LB                   2.00 Hz
GB                      0
PC                   1.40
```

3CF3PhBH2PPh(cyclopentyl)3

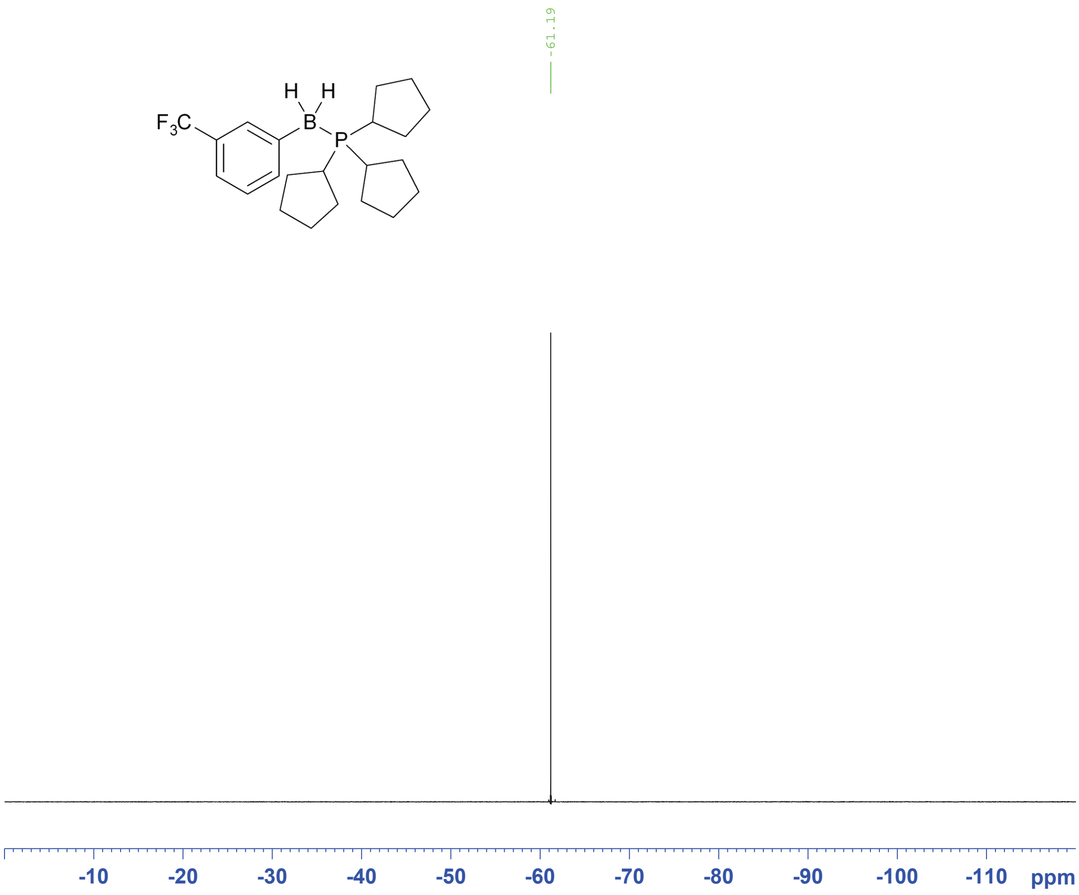

IBB-nmr Analysis

NAME	Apr23-2022
EXPNO	3
PROCNO	1
Date_	20220423
Time	12.18
INSTRUM	av400
PROBHD	5 mm PABBO BB-
PULPROG	zgfhigqn
TD	131072
SOLVENT	DMSO
NS	8
DS	4
SWH	89285.711 Hz
FIDRES	0.681196 Hz
AQ	0.7340532 sec
RG	203
DW	5.600 usec
DE	6.50 usec
TE	296.8 K
D1	1.00000000 sec
D11	0.03000000 sec
D12	0.00002000 sec
TD0	1
======== CHANNEL f1 ========	
NUC1	19F
P1	19.50 usec
PL1	-4.00 dB
PL1W	16.40489006 W
SFO1	376.4607164 MHz
======== CHANNEL f2 ========	
CPDPRG2	waltz16
NUC2	1H
PCPD2	112.00 usec
PL2	-4.00 dB
PL12	18.00 dB
PL2W	24.60733604 W
PL12W	0.15526180 W
SFO2	400.1316005 MHz
SI	65536
SF	376.4984617 MHz
WDW	EM
SSB	0
LB	0.30 Hz
GB	0
PC	1.00

3CF3PhBH2PPh(cyclopentyl)3

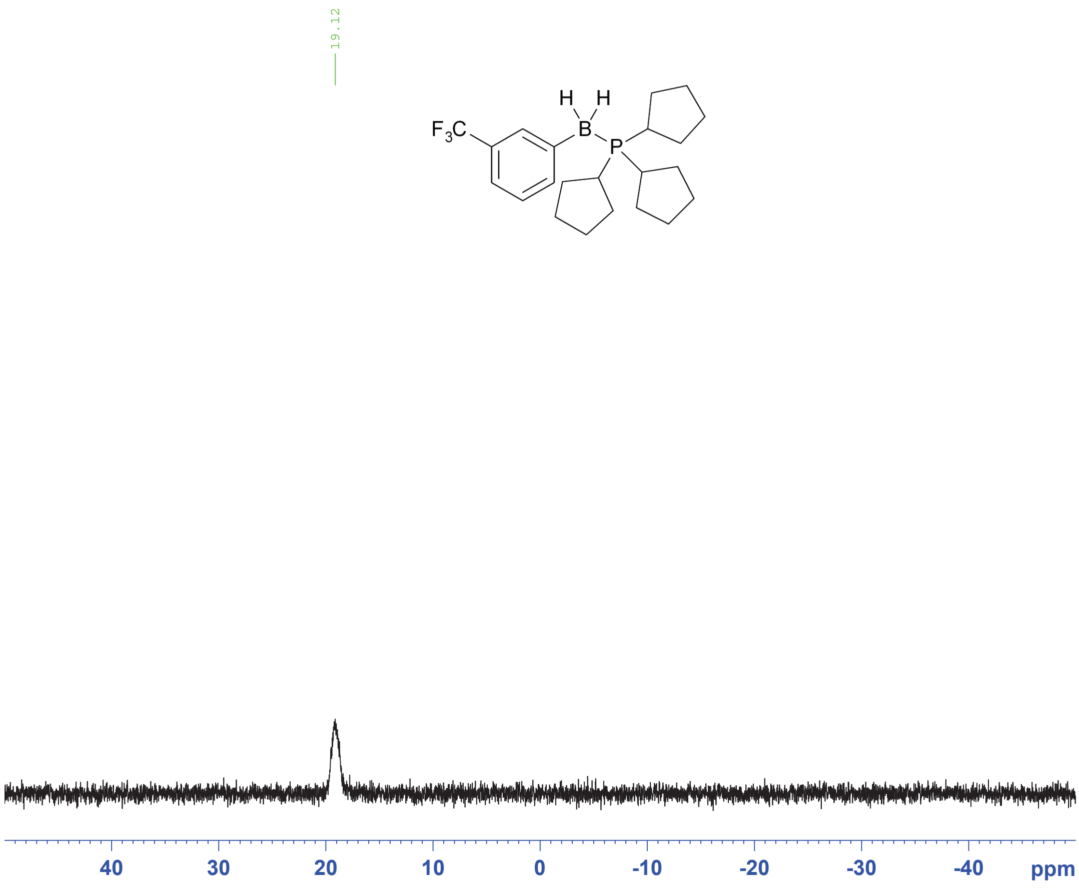

IBB-nmr Analysis

NAME	Apr23-2022
EXPNO	2
PROCNO	1
Date_	20220423
Time	12.13
INSTRUM	av400
PROBHD	5 mm PABBO BB-
PULPROG	zg30
TD	65536
SOLVENT	DMSO
NS	64
DS	4
SWH	64102.563 Hz
FIDRES	0.978127 Hz
AQ	0.5112308 sec
RG	203
DW	7.800 usec
DE	6.50 usec
TE	296.7 K
D1	2.00000000 sec
TD0	1
======== CHANNEL f1 ========	
NUC1	31P
P1	10.00 usec
PL1	-0.70 dB
PL1W	30.10233498 W
SFO1	161.9674942 MHz
SI	32768
SF	161.9754962 MHz
WDW	EM
SSB	0
LB	1.00 Hz
GB	0
PC	1.40

3CF3PhBH2P(cyclohexyl)3

B-3-(Trifluoromethyl)phenyl tricyclohexylphosphine–borane (25)

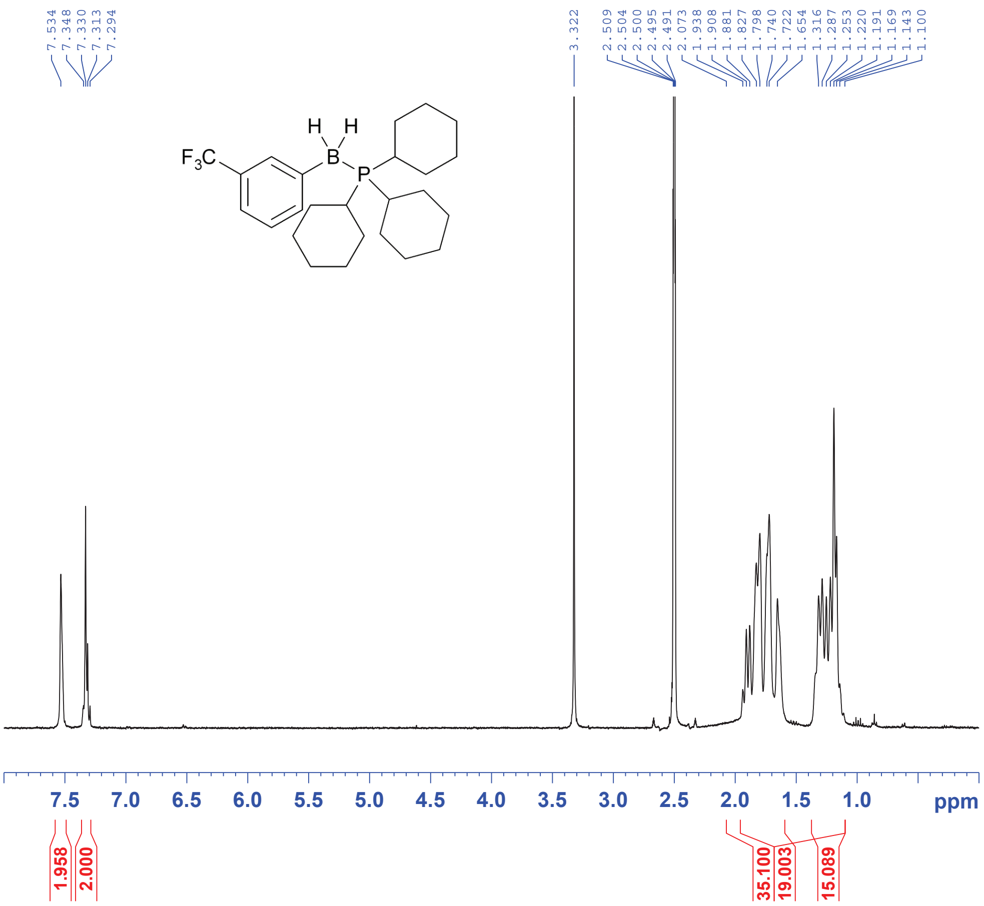

IBB-nmr Analysis

NAME	Mar23-2022
EXPNO	2
PROCNO	1
Date_	20220323
Time	20.41
INSTRUM	av400
PROBHD	5 mm PABBO BB-
PULPROG	zg30
TD	65536
SOLVENT	DMSO
NS	16
DS	2
SWH	8223.685 Hz
FIDRES	0.125483 Hz
AQ	3.9846387 sec
RG	203
DW	60.800 usec
DE	6.50 usec
TE	296.6 K
D1	1.00000000 sec
TD0	1
======== CHANNEL f1 ========	
NUC1	1H
P1	14.00 usec
PL1	-1.80 dB
PL1W	14.82738590 W
SFO1	400.1324710 MHz
SI	32768
SF	400.1300029 MHz
WDW	EM
SSB	0
LB	0.30 Hz
GB	0
PC	1.00

3CF3PhBH2P(cyclohexyl)3

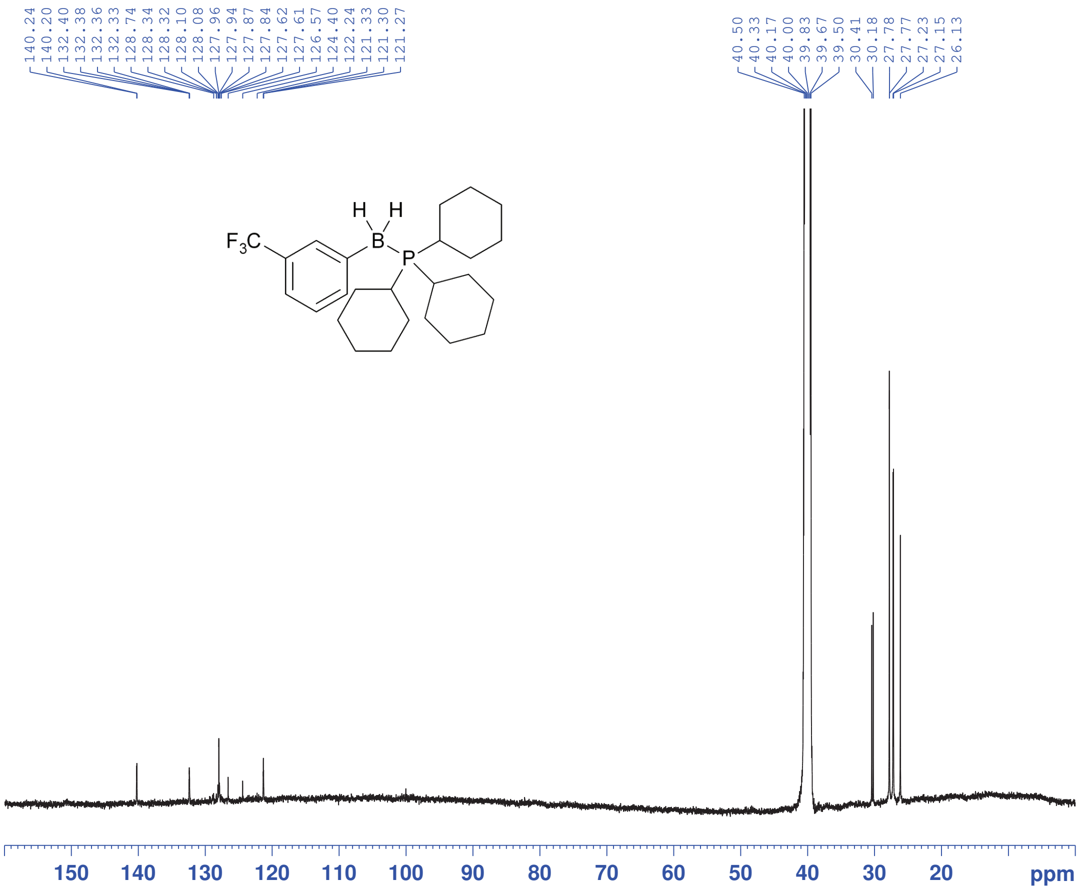

IBB-nmr Analysis

NAME	July30-2022
EXPNO	4
PROCNO	1
Date_	20220731
Time	4.53
INSTRUM	av500
PROBHD	5 mm CPDCH 13C
PULPROG	zgpg30
TD	65536
SOLVENT	DMSO
NS	8192
DS	4
SWH	30030.029 Hz
FIDRES	0.458222 Hz
AQ	1.0912410 sec
RG	35.9
DW	16.650 usec
DE	20.00 usec
TE	298.0 K
D1	2.00000000 sec
D11	0.03000000 sec
TD0	1
======== CHANNEL f1 ========	
NUC1	13C
P1	10.00 usec
PL1	-5.00 dB
PL1W	14.29790783 W
SFO1	125.7703643 MHz
======== CHANNEL f2 ========	
CPDPRG2	waltz16
NUC2	1H
PCPD2	80.00 usec
PL2	-4.00 dB
PL12	14.06 dB
PL13	17.00 dB
PL2W	6.30957365 W
PL12W	0.09862794 W
PL13W	0.05011872 W
SFO2	500.1320005 MHz
SI	32768
SF	125.7577857 MHz
WDW	EM
SSB	0
LB	1.00 Hz
GB	0
PC	0.30

3CF3PhBH2P(cyclohexyl)3

IBB-nmr Analysis

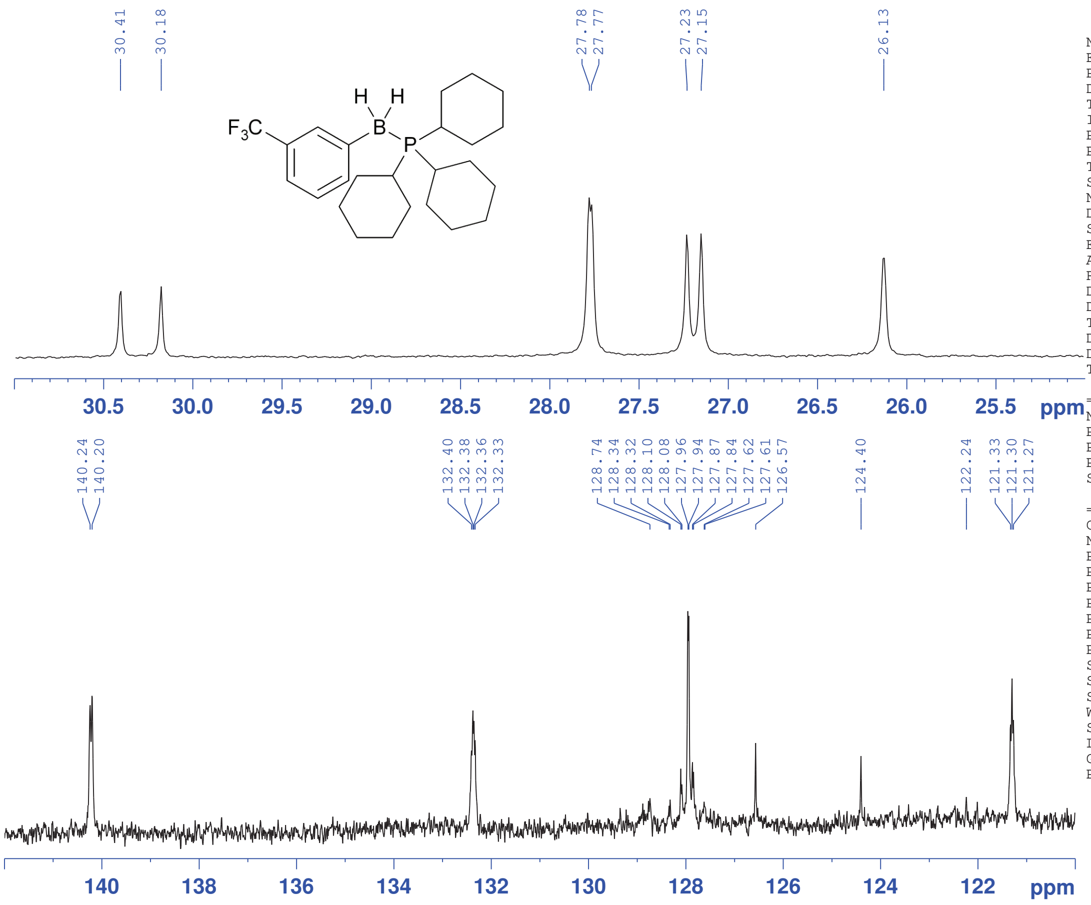

NAME	July30-2022
EXPNO	4
PROCNO	1
Date_	20220731
Time	4.53
INSTRUM	av500
PROBHD	5 mm CPDCH 13C
PULPROG	zgpg30
TD	65536
SOLVENT	DMSO
NS	8192
DS	4
SWH	30030.029 Hz
FIDRES	0.458222 Hz
AQ	1.0912410 sec
RG	35.9
DW	16.650 usec
DE	20.00 usec
TE	298.0 K
D1	2.00000000 sec
D11	0.03000000 sec
TD0	1
======== CHANNEL f1 ========	
NUC1	13C
P1	10.00 usec
PL1	-5.00 dB
PL1W	14.29790783 W
SFO1	125.7703643 MHz
======== CHANNEL f2 ========	
CPDPRG2	waltz16
NUC2	1H
PCPD2	80.00 usec
PL2	-4.00 dB
PL12	14.06 dB
PL13	17.00 dB
PL2W	6.30957365 W
PL12W	0.09862794 W
PL13W	0.05011872 W
SFO2	500.1320005 MHz
SI	32768
SF	125.7577857 MHz
WDW	EM
SSB	0
LB	1.00 Hz
GB	0
PC	0.30

3CF3PhBH2P(cyclohexyl)3

IBB-nmr Analysis

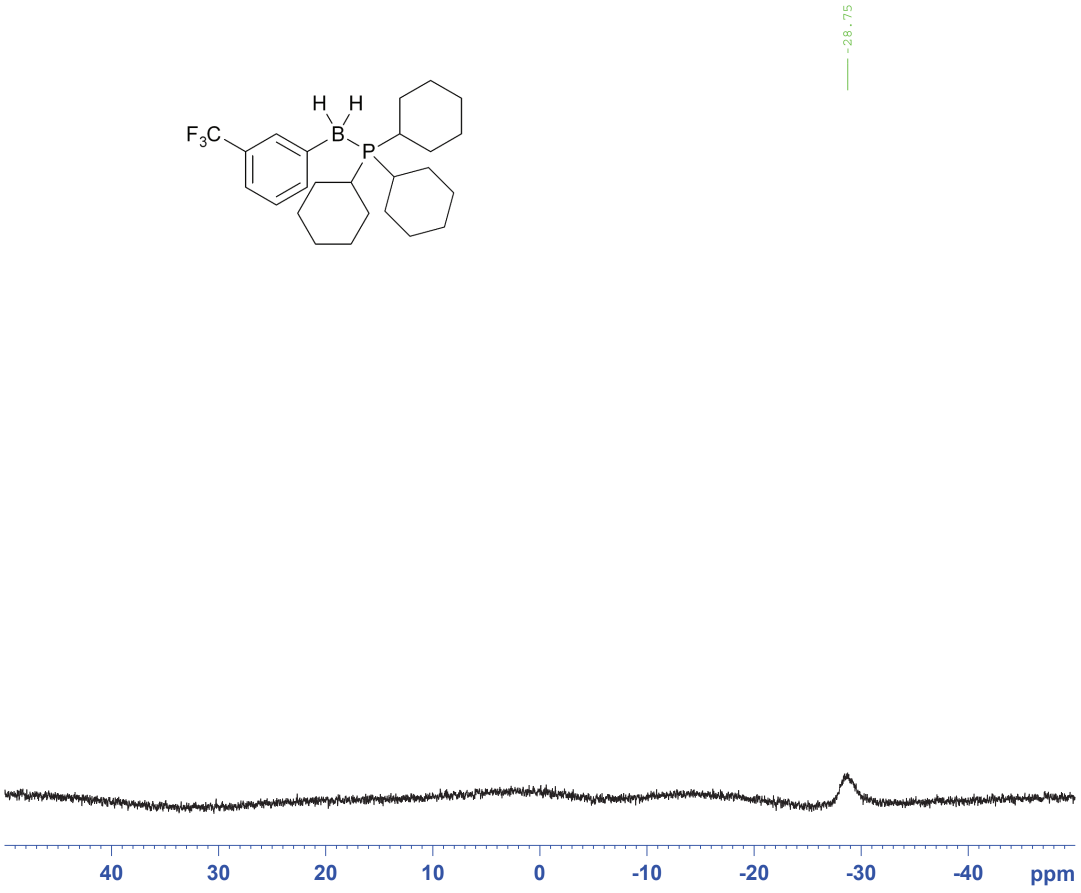

NAME	Mar13-2022
EXPNO	3
PROCNO	1
Date_	20220313
Time	19.40
INSTRUM	av400
PROBHD	5 mm PABBO BB-
PULPROG	zgig
TD	32768
SOLVENT	DMSO
NS	256
DS	4
SWH	20000.000 Hz
FIDRES	0.610352 Hz
AQ	0.8192500 sec
RG	203
DW	25.000 usec
DE	20.00 usec
TE	297.2 K
D1	1.00000000 sec
D11	0.03000000 sec
TD0	1
======== CHANNEL f1 ========	
NUC1	11B
P1	6.30 usec
PL1	-2.00 dB
SFO1	128.3776050 MHz
======== CHANNEL f2 ========	
CPDPRG2	waltz16
NUC2	1H
PCPD2	80.00 usec
PL2	-1.70 dB
PL12	13.80 dB
PL2W	14.48987389 W
PL12W	0.40838012 W
SFO2	400.1316005 MHz
SI	32768
SF	128.3776183 MHz
WDW	EM
SSB	0
LB	2.00 Hz
GB	0
PC	1.40

3CF3PhBH2P(cyclohexyl)3

IBB-nmr Analysis

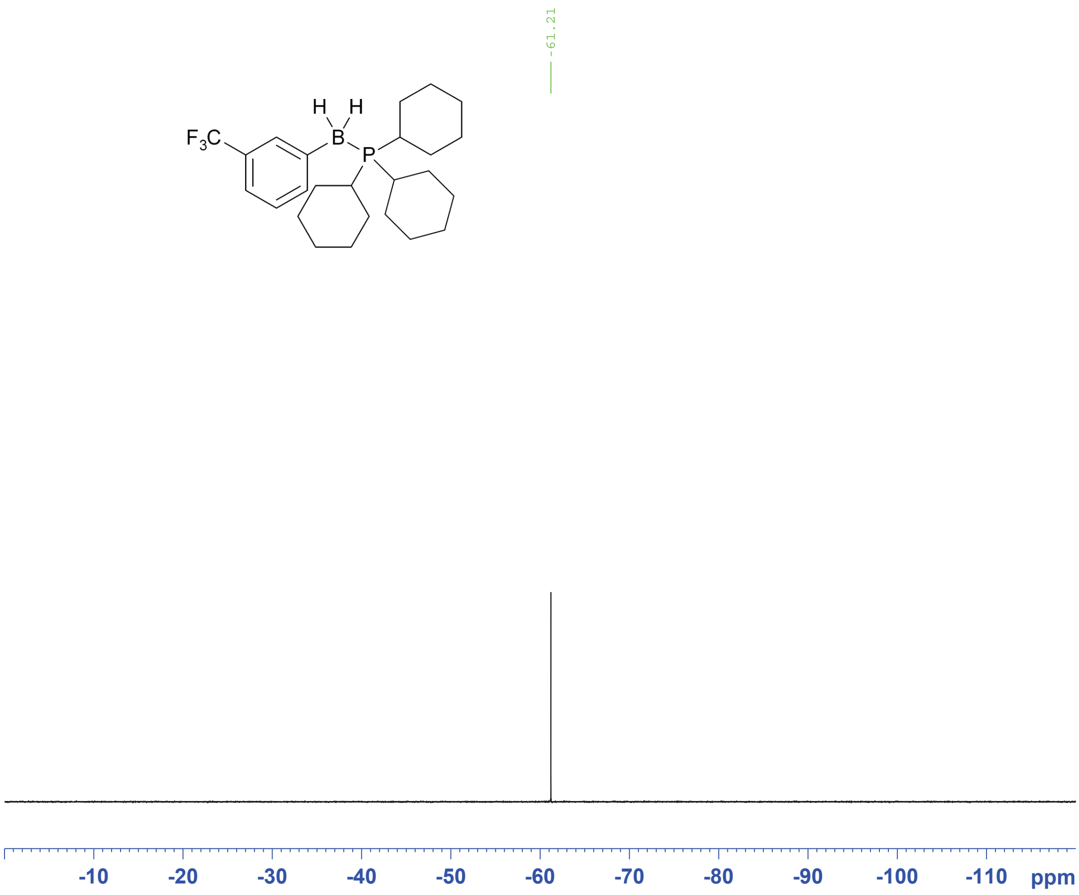


```
NAME            Mar13-2022
EXPNO                    2
PROCNO                   1
Date_             20220313
Time                 19.29
INSTRUM              av400
PROBHD    5 mm PABBO BB-
PULPROG           zgfhigqn
TD                  131072
SOLVENT               DMSO
NS                       8
DS                       4
SWH              89285.711 Hz
FIDRES            0.681196 Hz
AQ               0.7340532 sec
RG                     203
DW                   5.600 usec
DE                    6.50 usec
TE                   296.7 K
D1              1.00000000 sec
D11             0.03000000 sec
D12             0.00002000 sec
TD0                      1

======== CHANNEL f1 ========
NUC1                   19F
P1                   19.50 usec
PL1                  -4.00 dB
PL1W           16.40489006 W
SFO1           376.4607164 MHz

======== CHANNEL f2 ========
CPDPRG2            waltz16
NUC2                    1H
PCPD2               112.00 usec
PL2                  -4.00 dB
PL12                 18.00 dB
PL2W           24.60733604 W
PL12W           0.15526180 W
SFO2           400.1316005 MHz
SI                   65536
SF             376.4984617 MHz
WDW                     EM
SSB                      0
LB                    0.30 Hz
GB                       0
PC                    1.00
```

3CF3PhBH2P(cyclohexyl)3

IBB-nmr Analysis

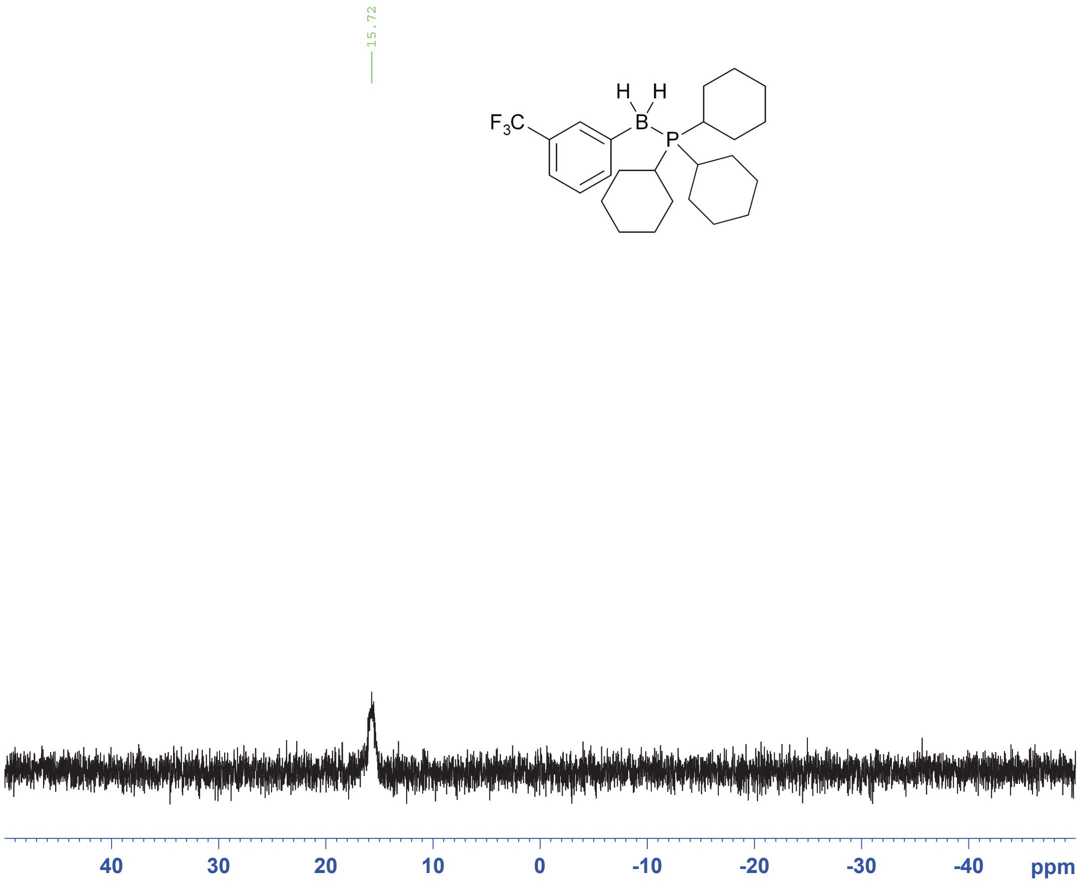


```
NAME            Mar13-2022
EXPNO                    5
PROCNO                   1
Date_             20220313
Time                 20.02
INSTRUM              av400
PROBHD    5 mm PABBO BB-
PULPROG               zg30
TD                   65536
SOLVENT               DMSO
NS                     256
DS                       4
SWH              64102.563 Hz
FIDRES            0.978127 Hz
AQ               0.5112308 sec
RG                     203
DW                   7.800 usec
DE                    6.50 usec
TE                   296.5 K
D1              2.00000000 sec
TD0                      1

======== CHANNEL f1 ========
NUC1                   31P
P1                   10.00 usec
PL1                  -0.70 dB
PL1W           30.10233498 W
SFO1           161.9674942 MHz
SI                   32768
SF             161.9754962 MHz
WDW                     EM
SSB                      0
LB                    1.00 Hz
GB                       0
PC                    1.40
```

B-3-(Trifluoromethyl)phenyl dicyclohexylphosphine–borane (26)

3CF3PhBH2PH(cyclohexyl)2

IBB-nmr Analysis

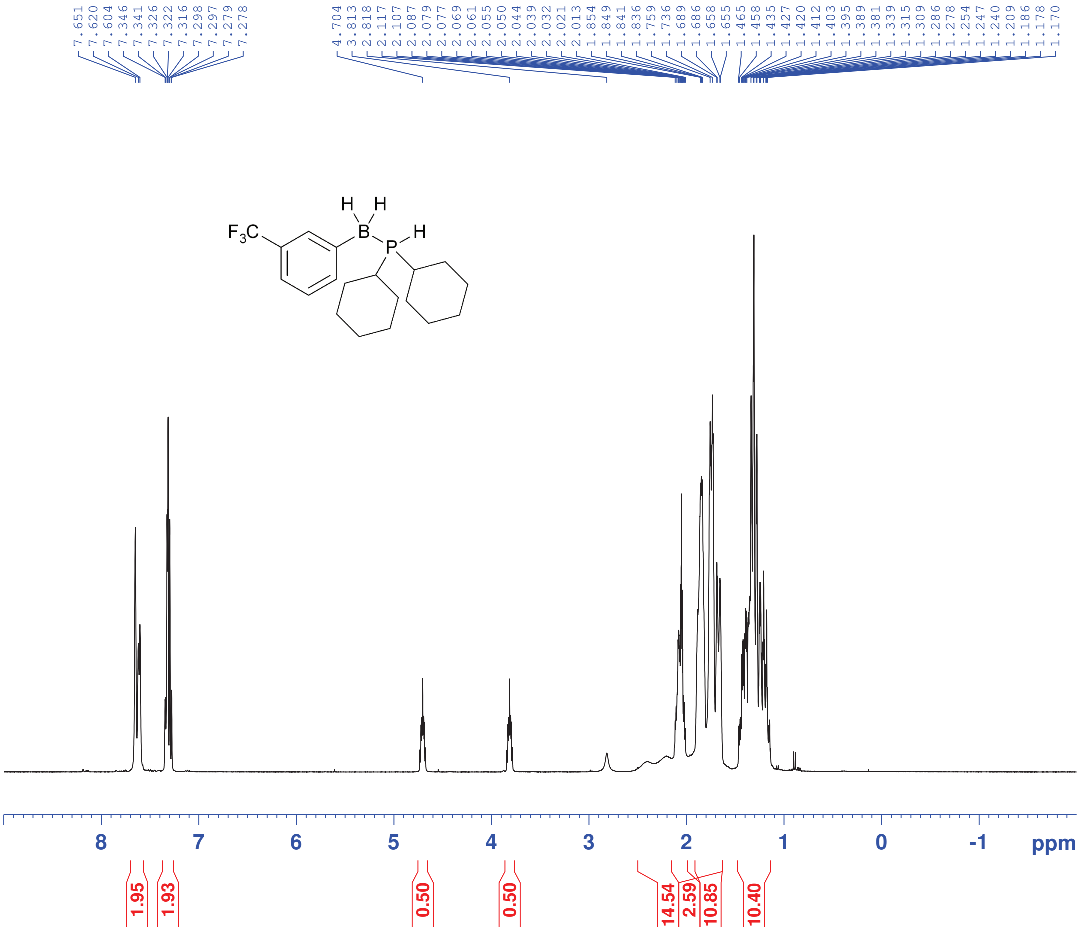


```
NAME         Aug12-2023
EXPNO                 2
PROCNO                1
Date_          20230812
Time              19.39
INSTRUM           av400
PROBHD   5 mm PABBO BB-
PULPROG            zg30
TD                65536
SOLVENT         Acetone
NS                   16
DS                    2
SWH            8223.685 Hz
FIDRES         0.125483 Hz
AQ            3.9846387 sec
RG                   32
DW               60.800 usec
DE                 6.50 usec
TE                297.1 K
D1           1.00000000 sec
TD0                   1

======== CHANNEL f1 ========
NUC1                 1H
P1                14.00 usec
PL1               -1.80 dB
PL1W        14.82738590 W
SFO1        400.1324710 MHz
SI                32768
SF          400.1300070 MHz
WDW                  EM
SSB                   0
LB                 0.30 Hz
GB                    0
PC                 1.00
```

3CF3PhBH2PH(cyclohexyl)2

IBB-nmr Analysis

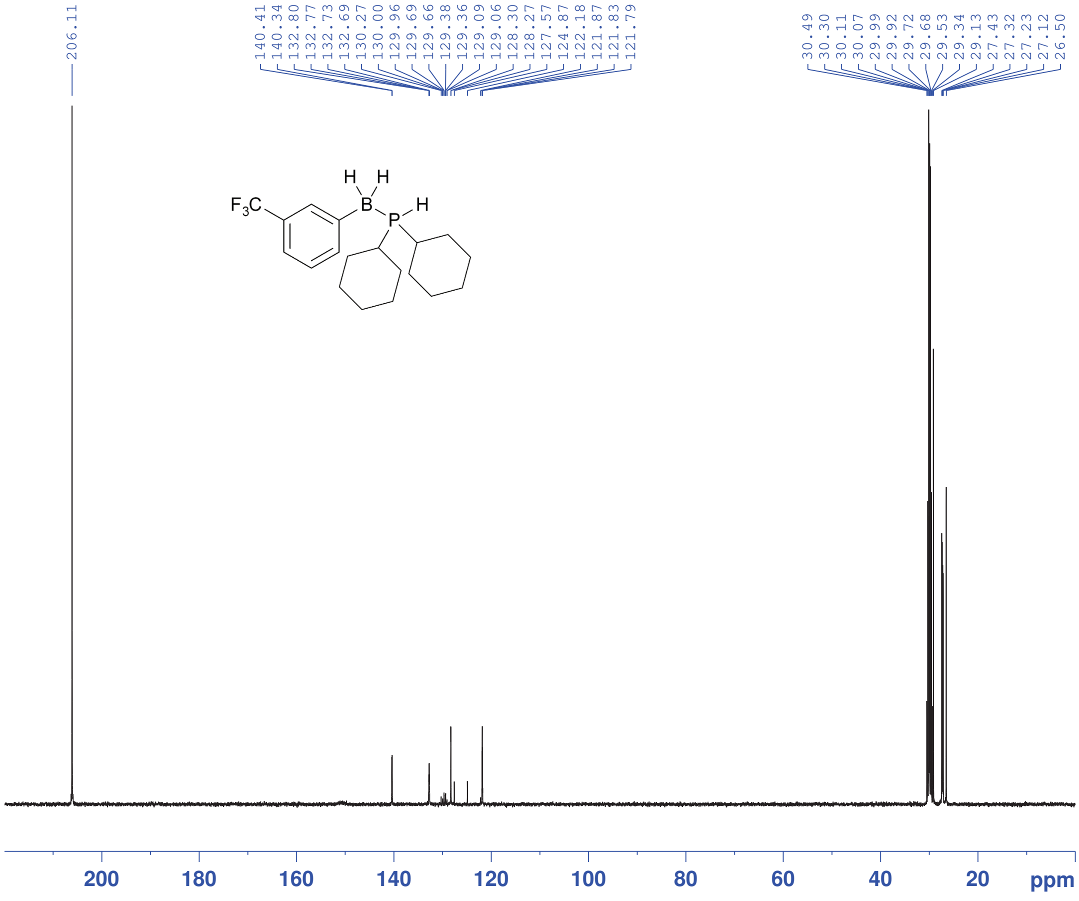


```
NAME         Aug12-2023
EXPNO                 3
PROCNO                1
Date_          20230812
Time              19.57
INSTRUM           av400
PROBHD   5 mm PABBO BB-
PULPROG          zgpg30
TD                65536
SOLVENT         Acetone
NS                  257
DS                    2
SWH           24038.461 Hz
FIDRES         0.366798 Hz
AQ            1.3631988 sec
RG                  128
DW               20.800 usec
DE                 6.50 usec
TE                298.4 K
D1           2.00000000 sec
D11          0.03000000 sec
TD0                   1

======== CHANNEL f1 ========
NUC1                13C
P1                 9.00 usec
PL1               -3.00 dB
PL1W        73.17333984 W
SFO1        100.6228298 MHz

======== CHANNEL f2 ========
CPDPRG2         waltz16
NUC2                 1H
PCPD2             80.00 usec
PL2               -1.70 dB
PL12              13.80 dB
PL13              13.80 dB
PL2W        14.48987389 W
PL12W        0.40838012 W
PL13W        0.40838012 W
SFO2        400.1316005 MHz
SI                32768
SF          100.6126724 MHz
WDW                  EM
SSB                   0
LB                 1.00 Hz
GB                    0
PC                 0.30
```

3CF3PhBH2PH(cyclohexyl)2

IBB-nmr Analysis

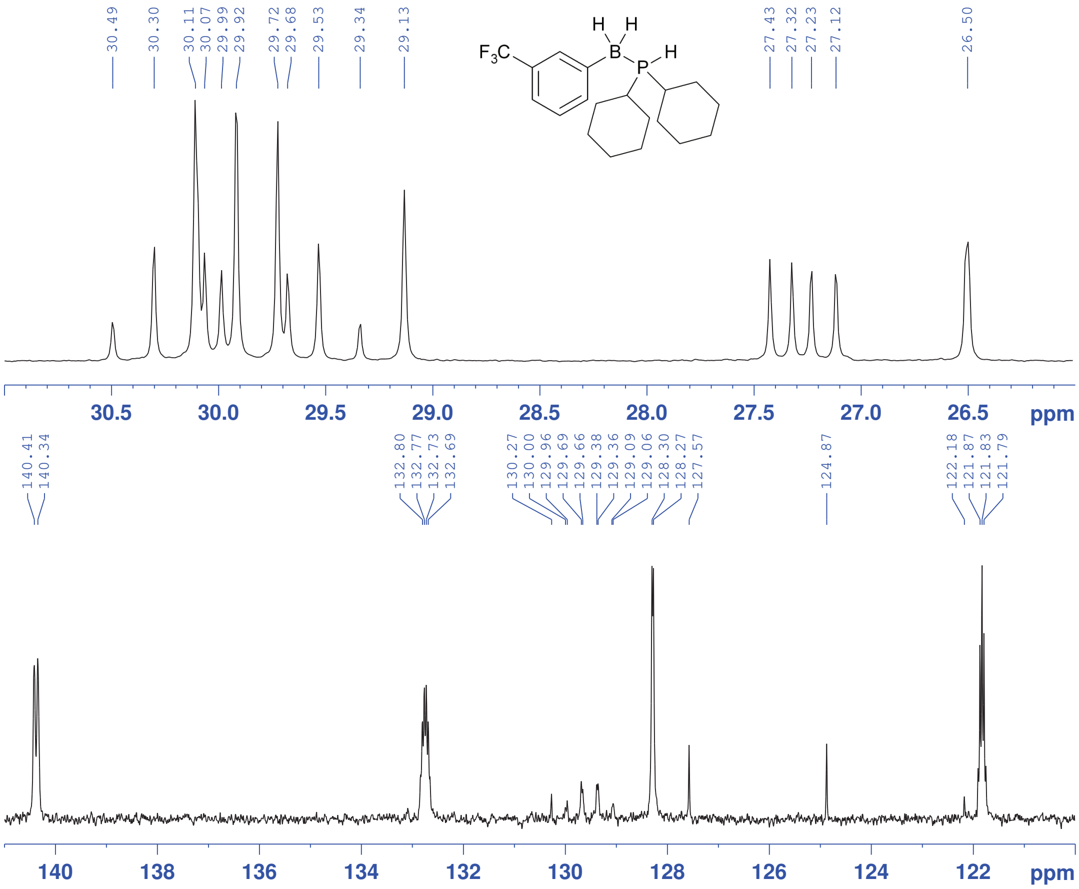


```
NAME          Aug12-2023
EXPNO                  3
PROCNO                 1
Date_           20230812
Time               19.57
INSTRUM            av400
PROBHD   5 mm PABBO BB-
PULPROG          zgpg30
TD                 65536
SOLVENT          Acetone
NS                   257
DS                     2
SWH            24038.461 Hz
FIDRES          0.366798 Hz
AQ             1.3631988 sec
RG                   128
DW                20.800 usec
DE                  6.50 usec
TE                 298.4 K
D1            2.00000000 sec
D11           0.03000000 sec
TD0                    1

======== CHANNEL f1 ========
NUC1                 13C
P1                  9.00 usec
PL1                -3.00 dB
PL1W         73.17333984 W
SFO1         100.6228298 MHz

======== CHANNEL f2 ========
CPDPRG2          waltz16
NUC2                  1H
PCPD2              80.00 usec
PL2                -1.70 dB
PL12               13.80 dB
PL13               13.80 dB
PL2W         14.48987389 W
PL12W         0.40838012 W
PL13W         0.40838012 W
SFO2         400.1316005 MHz
SI                 32768
SF           100.6126724 MHz
WDW                   EM
SSB                    0
LB                  1.00 Hz
GB                     0
PC                  0.30
```

3CF3PhBH2PH(cyclohexyl)2

IBB-nmr Analysis

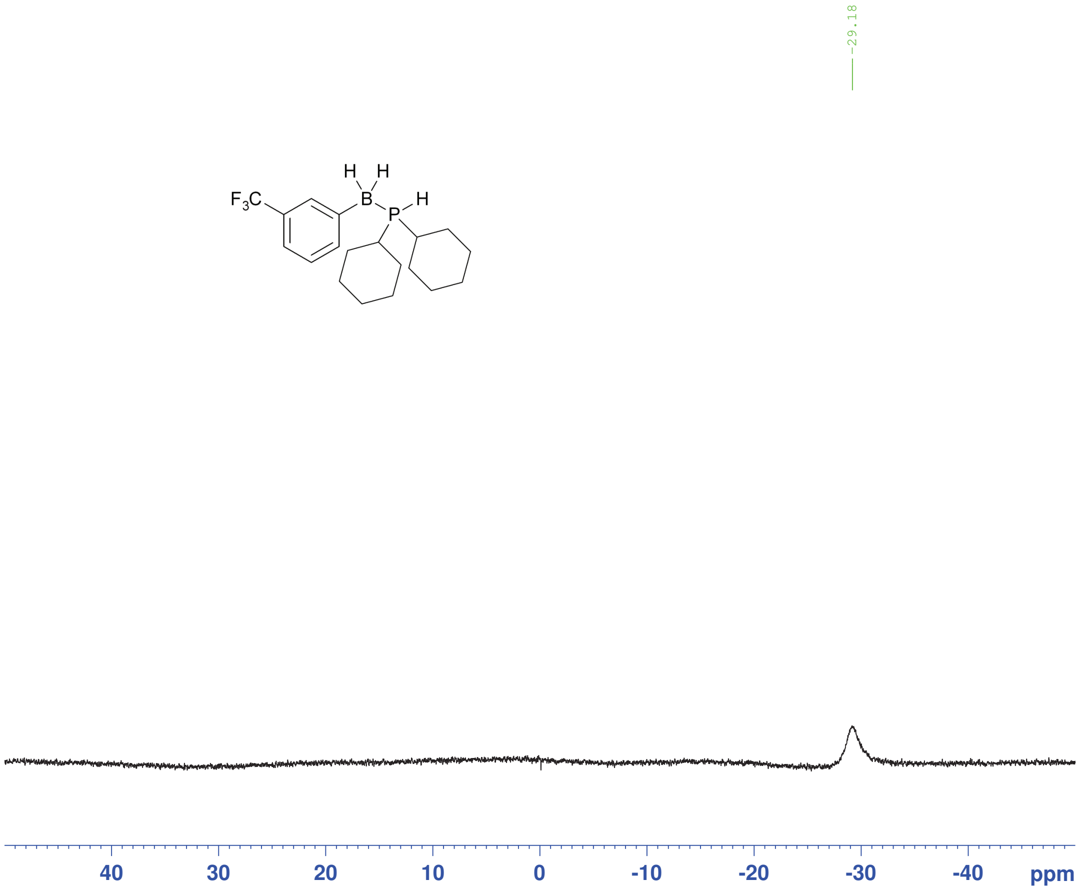


```
NAME          Jul27-2023
EXPNO                  4
PROCNO                 1
Date_           20230727
Time                9.26
INSTRUM            av400
PROBHD   5 mm PABBO BB-
PULPROG             zgig
TD                 32768
SOLVENT             DMSO
NS                    64
DS                     4
SWH            20000.000 Hz
FIDRES          0.610352 Hz
AQ             0.8192500 sec
RG                   203
DW                25.000 usec
DE                 20.00 usec
TE                 297.1 K
D1            1.00000000 sec
D11           0.03000000 sec
TD0                    1

======== CHANNEL f1 ========
NUC1                 11B
P1                  6.30 usec
PL1                -2.00 dB
SFO1         128.3776050 MHz

======== CHANNEL f2 ========
CPDPRG2          waltz16
NUC2                  1H
PCPD2              80.00 usec
PL2                -1.70 dB
PL12               13.80 dB
PL2W         14.48987389 W
PL12W         0.40838012 W
SFO2         400.1316005 MHz
SI                 32768
SF           128.3776183 MHz
WDW                   EM
SSB                    0
LB                  2.00 Hz
GB                     0
PC                  1.40
```

3CF3PhBH2PH(cyclohexyl)2

IBB-nmr Analysis

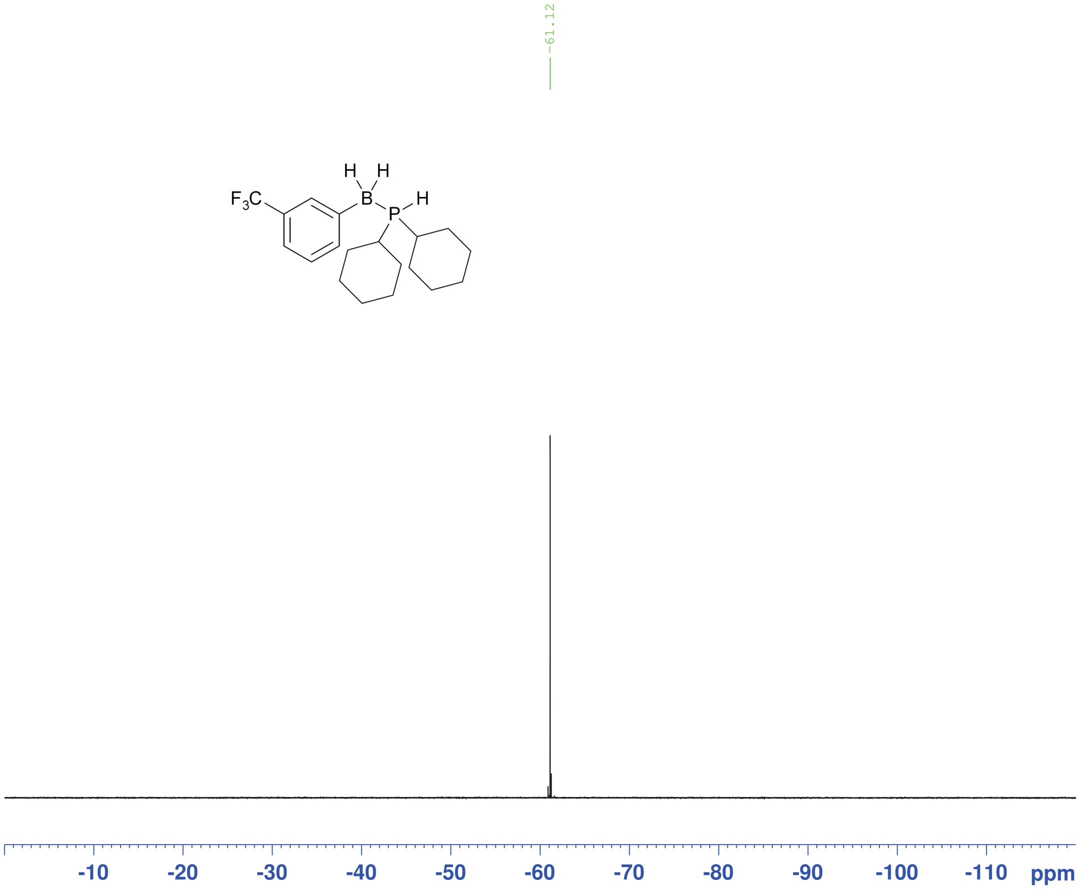


```
NAME          Jul27-2023
EXPNO                  5
PROCNO                 1
Date_           20230727
Time                9.30
INSTRUM            av400
PROBHD   5 mm PABBO BB-
PULPROG         zgfhigqn
TD                131072
SOLVENT             DMSO
NS                     8
DS                     4
SWH           89285.711 Hz
FIDRES         0.681196 Hz
AQ            0.7340532 sec
RG                   203
DW                 5.600 usec
DE                  6.50 usec
TE                 296.7 K
D1            1.00000000 sec
D11           0.03000000 sec
D12           0.00002000 sec
TD0                    1

======== CHANNEL f1 ========
NUC1                 19F
P1                 19.50 usec
PL1                -4.00 dB
PL1W         16.40489006 W
SFO1         376.4607164 MHz

======== CHANNEL f2 ========
CPDPRG2          waltz16
NUC2                  1H
PCPD2             112.00 usec
PL2                -4.00 dB
PL12               18.00 dB
PL2W         24.60733604 W
PL12W         0.15526180 W
SFO2         400.1316005 MHz
SI                 65536
SF           376.4984617 MHz
WDW                   EM
SSB                    0
LB                  0.30 Hz
GB                     0
PC                  1.00
```

3CF3PhBH2PH(cyclohexyl)2

IBB-nmr Analysis

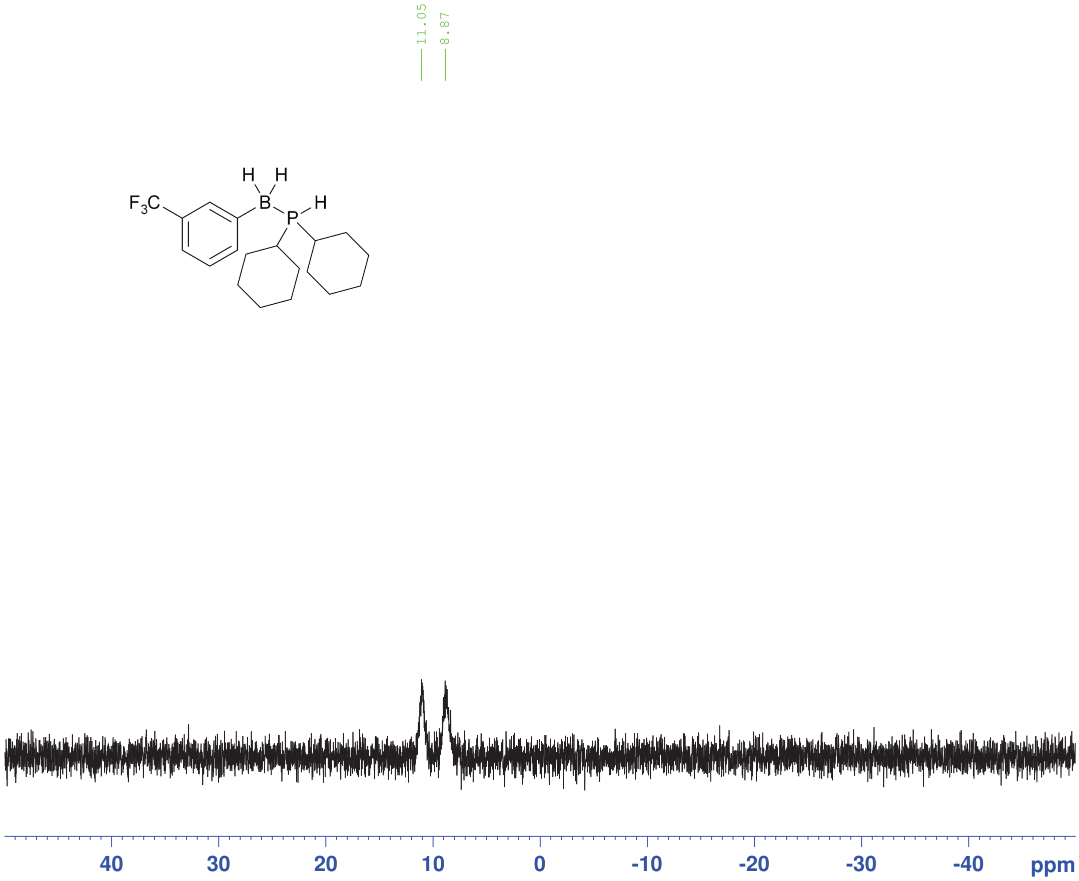


```
NAME          Jul27-2023
EXPNO                  3
PROCNO                 1
Date_           20230727
Time                9.21
INSTRUM            av400
PROBHD   5 mm PABBO BB-
PULPROG             zg30
TD                 65536
SOLVENT             DMSO
NS                    64
DS                     4
SWH           64102.563 Hz
FIDRES         0.978127 Hz
AQ            0.5112308 sec
RG                   203
DW                 7.800 usec
DE                  6.50 usec
TE                 296.6 K
D1            2.00000000 sec
TD0                    1

======== CHANNEL f1 ========
NUC1                 31P
P1                 10.00 usec
PL1                -0.70 dB
PL1W         30.10233498 W
SFO1         161.9674942 MHz
SI                 32768
SF           161.9754962 MHz
WDW                   EM
SSB                    0
LB                  1.00 Hz
GB                     0
PC                  1.40
```

3CF3PhBH2P(NMe2)3

B-3-(Trifluoromethyl)phenyl tris(dimethylamino)phosphine–borane (27)

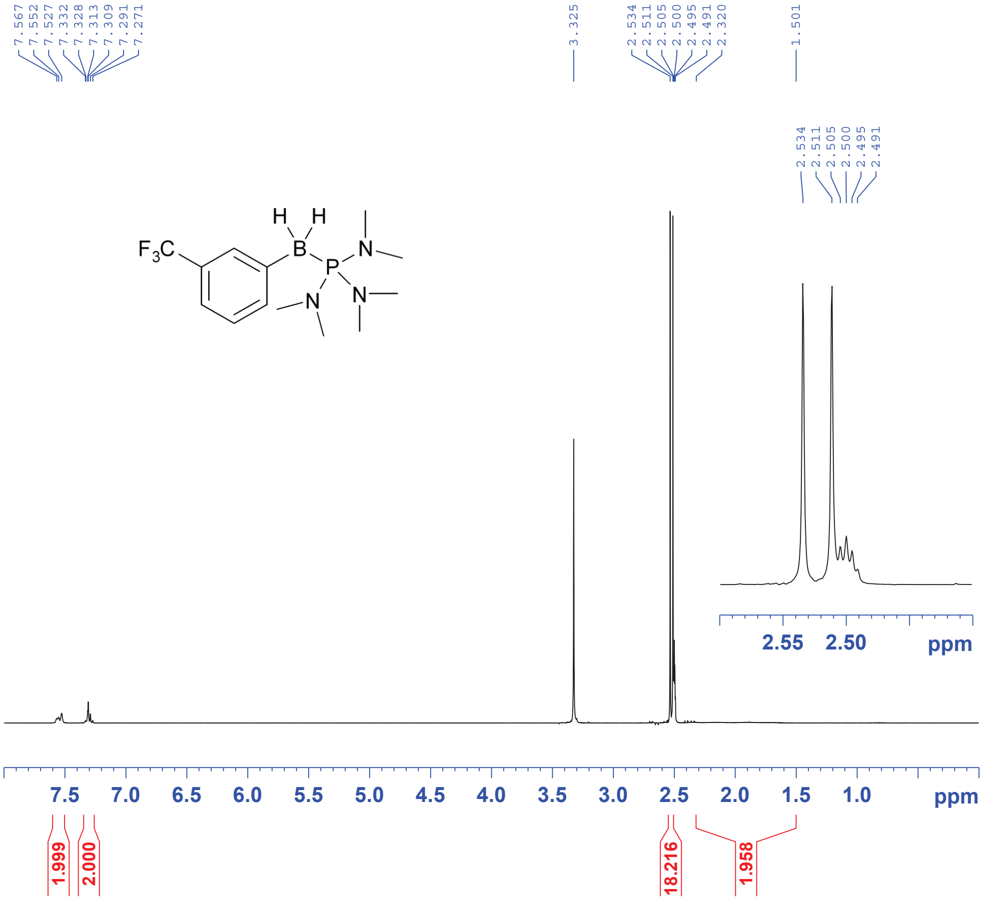

IBB-nmr Analysis

NAME	July18-2022
EXPNO	2
PROCNO	1
Date_	20220718
Time	17.37
INSTRUM	av400
PROBHD	5 mm PABBO BB-
PULPROG	zg30
TD	65536
SOLVENT	DMSO
NS	4
DS	2
SWH	8223.685 Hz
FIDRES	0.125483 Hz
AQ	3.9846387 sec
RG	203
DW	60.800 usec
DE	6.50 usec
TE	297.2 K
D1	1.00000000 sec
TD0	1

======== CHANNEL f1 ========

NUC1	1H
P1	14.00 usec
PL1	-1.80 dB
PL1W	14.82738590 W
SFO1	400.1324710 MHz
SI	32768
SF	400.1300034 MHz
WDW	EM
SSB	0
LB	0.30 Hz
GB	0
PC	1.00

3CF3PhBH2P(NMe2)3

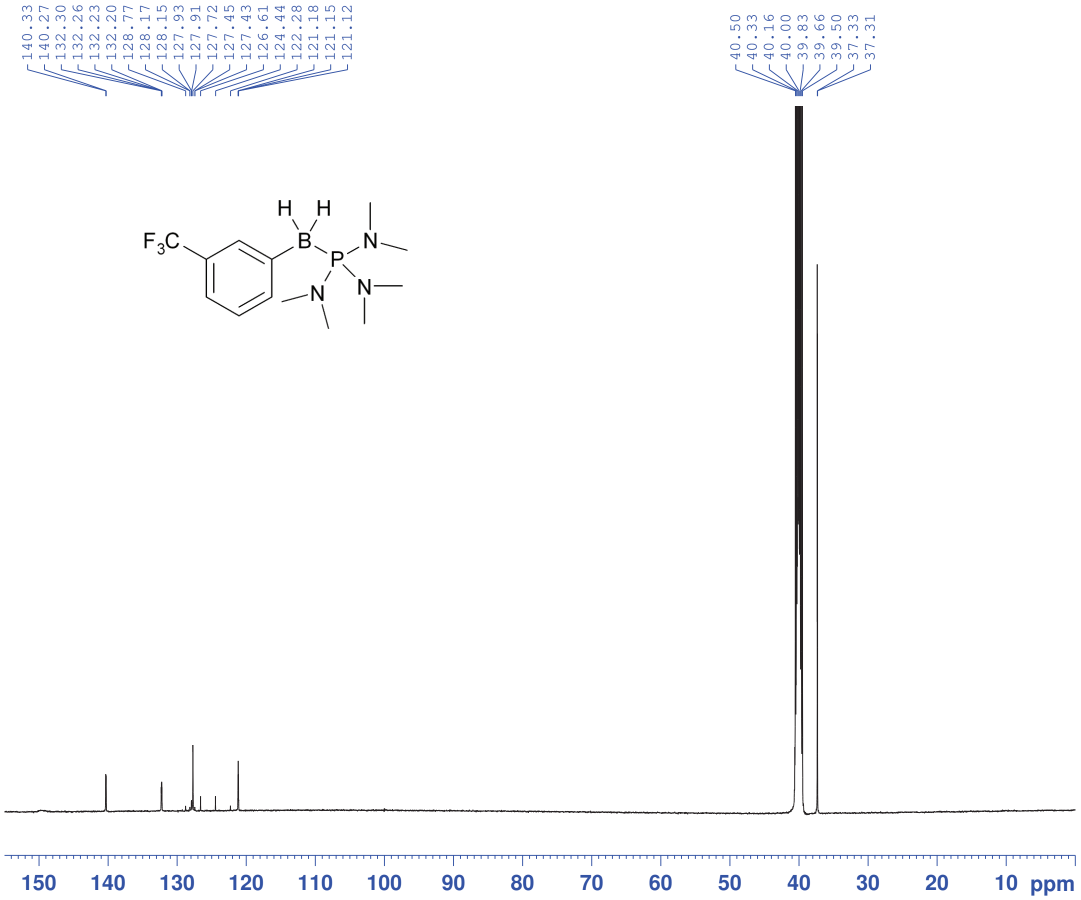

IBB-nmr Analysis

NAME	July18-2022
EXPNO	6
PROCNO	1
Date_	20220718
Time	21.37
INSTRUM	av500
PROBHD	5 mm CPDCH 13C
PULPROG	zgpg30
TD	65536
SOLVENT	DMSO
NS	4096
DS	4
SWH	30030.029 Hz
FIDRES	0.458222 Hz
AQ	1.0912410 sec
RG	35.9
DW	16.650 usec
DE	20.00 usec
TE	298.0 K
D1	2.00000000 sec
D11	0.03000000 sec
TD0	1

======== CHANNEL f1 ========

NUC1	13C
P1	10.00 usec
PL1	-5.00 dB
PL1W	14.29790783 W
SFO1	125.7703643 MHz

======== CHANNEL f2 ========

CPDPRG2	waltz16
NUC2	1H
PCPD2	80.00 usec
PL2	-4.00 dB
PL12	14.06 dB
PL13	17.00 dB
PL2W	6.30957365 W
PL12W	0.09862794 W
PL13W	0.05011872 W
SFO2	500.1320005 MHz
SI	32768
SF	125.7577866 MHz
WDW	EM
SSB	0
LB	1.00 Hz
GB	0
PC	0.30

3CF3PhBH2P(NMe2)3

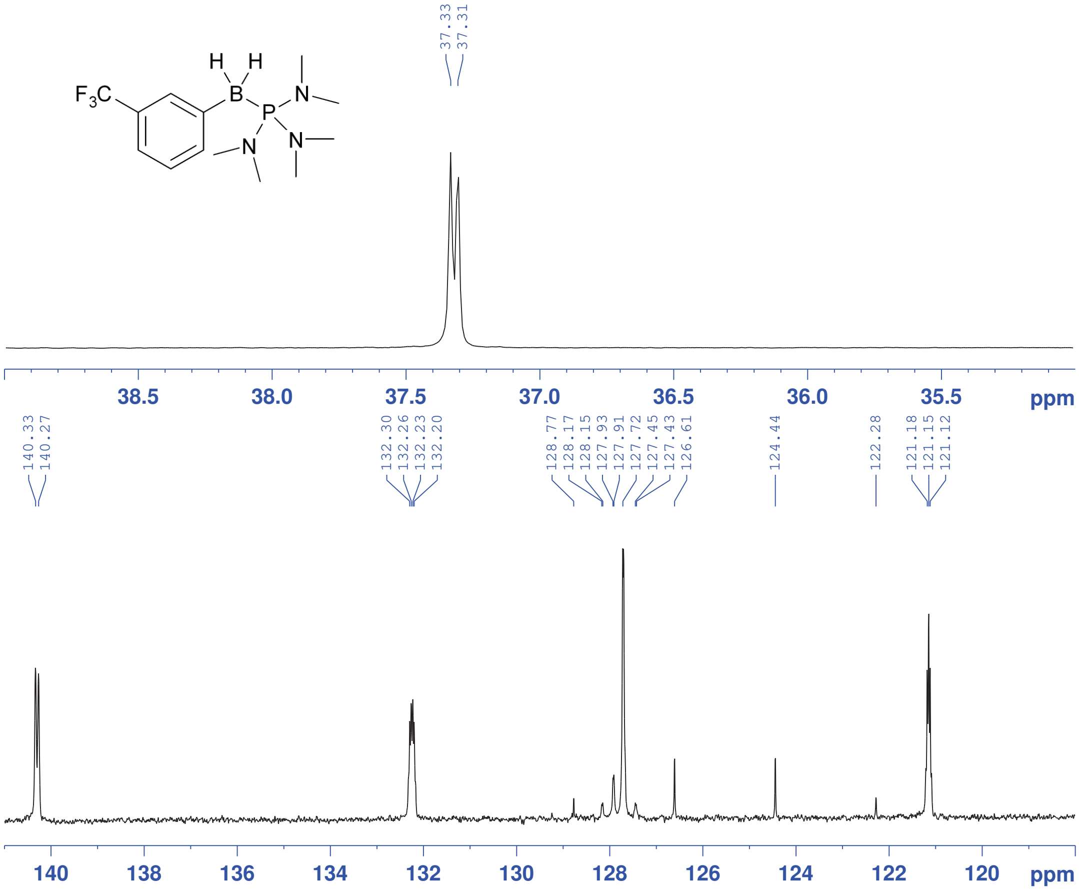

IBB-nmr Analysis

NAME	July18-2022
EXPNO	6
PROCNO	1
Date_	20220718
Time	21.37
INSTRUM	av500
PROBHD	5 mm CPDCH 13C
PULPROG	zgpg30
TD	65536
SOLVENT	DMSO
NS	4096
DS	4
SWH	30030.029 Hz
FIDRES	0.458222 Hz
AQ	1.0912410 sec
RG	35.9
DW	16.650 usec
DE	20.00 usec
TE	298.0 K
D1	2.00000000 sec
D11	0.03000000 sec
TD0	1
======== CHANNEL f1 ========	
NUC1	13C
P1	10.00 usec
PL1	-5.00 dB
PL1W	14.29790783 W
SFO1	125.7703643 MHz
======== CHANNEL f2 ========	
CPDPRG2	waltz16
NUC2	1H
PCPD2	80.00 usec
PL2	-4.00 dB
PL12	14.06 dB
PL13	17.00 dB
PL2W	6.30957365 W
PL12W	0.09862794 W
PL13W	0.05011872 W
SFO2	500.1320005 MHz
SI	32768
SF	125.7577866 MHz
WDW	EM
SSB	0
LB	1.00 Hz
GB	0
PC	0.30

3CF3PhBH2P(NMe2)3

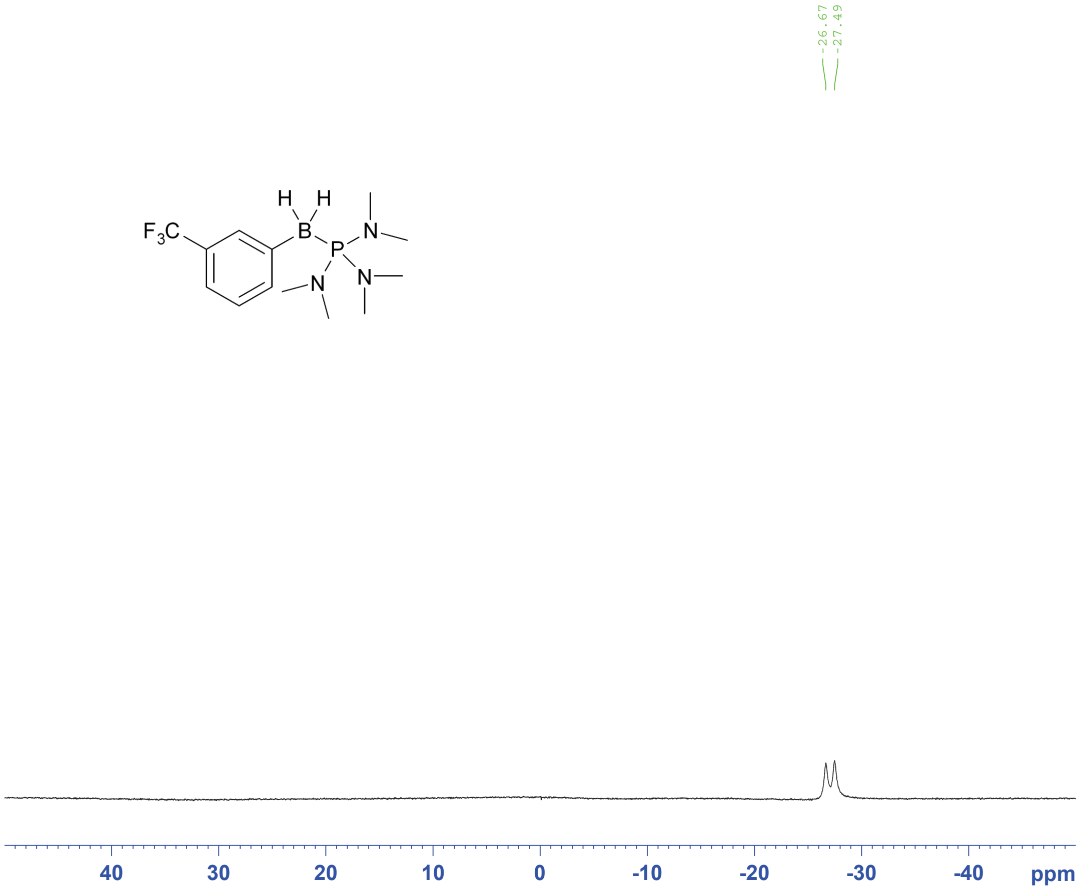

IBB-nmr Analysis

NAME	July18-2022
EXPNO	5
PROCNO	1
Date_	20220718
Time	17.52
INSTRUM	av400
PROBHD	5 mm PABBO BB-
PULPROG	zgig
TD	32768
SOLVENT	DMSO
NS	128
DS	4
SWH	20000.000 Hz
FIDRES	0.610352 Hz
AQ	0.8192500 sec
RG	203
DW	25.000 usec
DE	20.00 usec
TE	297.7 K
D1	1.00000000 sec
D11	0.03000000 sec
TD0	1
======== CHANNEL f1 ========	
NUC1	11B
P1	6.30 usec
PL1	-2.00 dB
SFO1	128.3776050 MHz
======== CHANNEL f2 ========	
CPDPRG2	waltz16
NUC2	1H
PCPD2	80.00 usec
PL2	-1.70 dB
PL12	13.80 dB
PL2W	14.48987389 W
PL12W	0.40838012 W
SFO2	400.1316005 MHz
SI	32768
SF	128.3776183 MHz
WDW	EM
SSB	0
LB	2.00 Hz
GB	0
PC	1.40

3CF3PhBH2P(NMe2)3

IBB-nmr Analysis

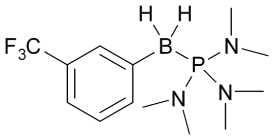

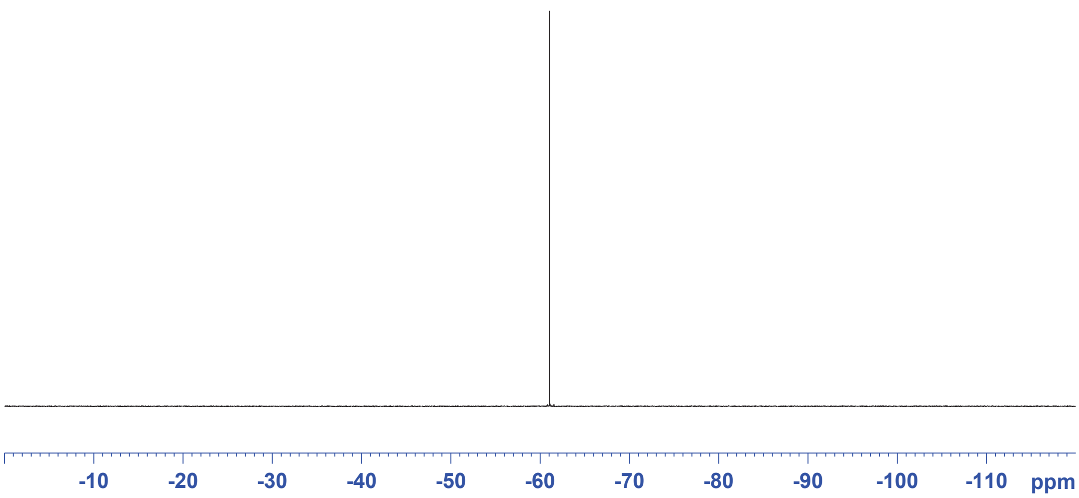


```
NAME          July18-2022
EXPNO                   4
PROCNO                  1
Date_            20220718
Time                17.45
INSTRUM             av400
PROBHD   5 mm PABBO BB-
PULPROG          zgfhigqn
TD                 131072
SOLVENT              DMSO
NS                      8
DS                      4
SWH             89285.711 Hz
FIDRES           0.681196 Hz
AQ              0.7340532 sec
RG                    203
DW                  5.600 usec
DE                   6.50 usec
TE                  297.2 K
D1             1.00000000 sec
D11            0.03000000 sec
D12            0.00002000 sec
TD0                     1

======== CHANNEL f1 ========
NUC1                  19F
P1                  19.50 usec
PL1                 -4.00 dB
PL1W          16.40489006 W
SFO1          376.4607164 MHz

======== CHANNEL f2 ========
CPDPRG2           waltz16
NUC2                   1H
PCPD2              112.00 usec
PL2                 -4.00 dB
PL12                18.00 dB
PL2W          24.60733604 W
PL12W          0.15526180 W
SFO2          400.1316005 MHz
SI                  65536
SF            376.4984617 MHz
WDW                    EM
SSB                     0
LB                   0.30 Hz
GB                      0
PC                   1.00
```

3CF3PhBH2P(NMe2)3

IBB-nmr Analysis

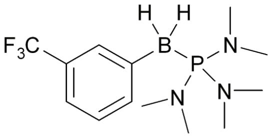

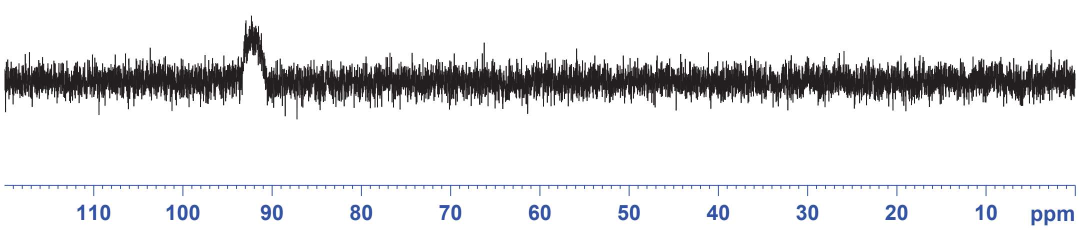


```
NAME          July18-2022
EXPNO                   3
PROCNO                  1
Date_            20220718
Time                17.42
INSTRUM             av400
PROBHD   5 mm PABBO BB-
PULPROG              zg30
TD                  65536
SOLVENT              DMSO
NS                     64
DS                      4
SWH             64102.563 Hz
FIDRES           0.978127 Hz
AQ              0.5112308 sec
RG                    203
DW                  7.800 usec
DE                   6.50 usec
TE                  297.1 K
D1             2.00000000 sec
TD0                     1

======== CHANNEL f1 ========
NUC1                  31P
P1                  10.00 usec
PL1                 -0.70 dB
PL1W          30.10233498 W
SFO1          161.9674942 MHz
SI                  32768
SF            161.9754962 MHz
WDW                    EM
SSB                     0
LB                   1.00 Hz
GB                      0
PC                   1.40
```

4CF3PhBH2PMe3 5-14Fr

B-4-(Trifluoromethyl)phenyl trimethylphosphine–borane (28)

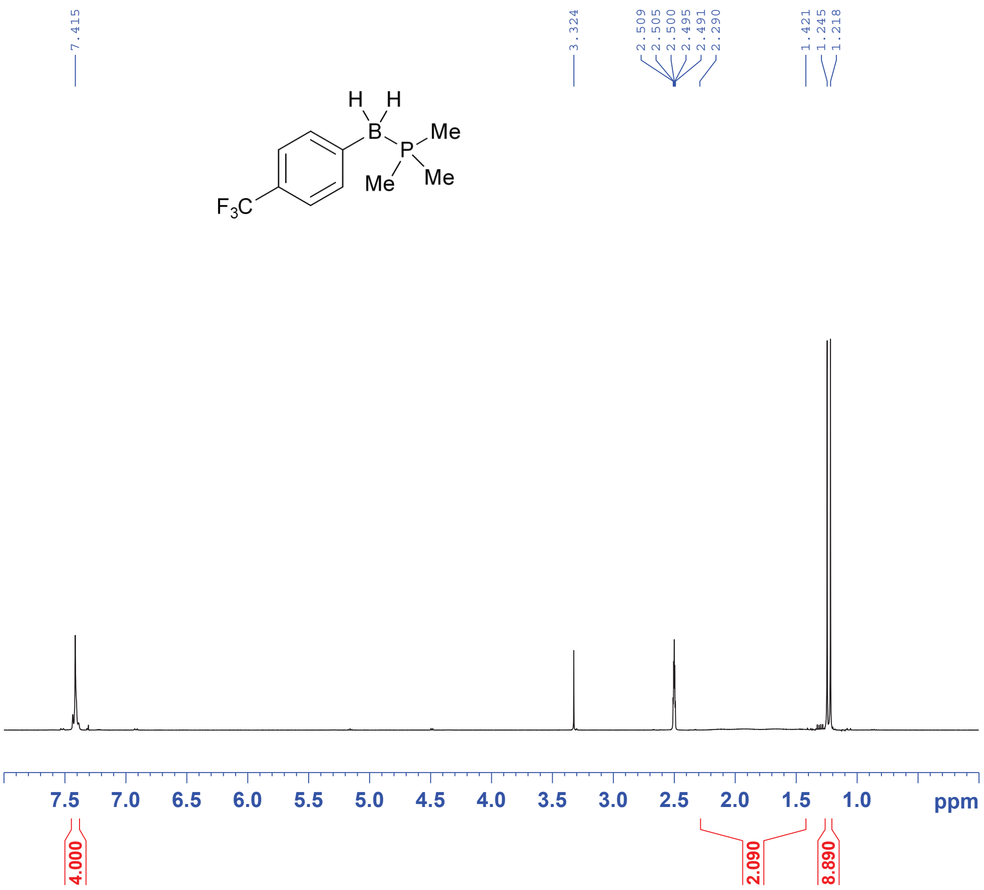

IBB-nmr Analysis

NAME	Dec6-2021
EXPNO	1
PROCNO	1
Date_	20211206
Time	20.49
INSTRUM	av400
PROBHD	5 mm PABBO BB-
PULPROG	zg30
TD	65536
SOLVENT	DMSO
NS	16
DS	2
SWH	8223.685 Hz
FIDRES	0.125483 Hz
AQ	3.9846387 sec
RG	203
DW	60.800 usec
DE	6.50 usec
TE	296.6 K
D1	1.00000000 sec
TD0	1
======== CHANNEL f1 ========	
NUC1	1H
P1	14.00 usec
PL1	-1.80 dB
PL1W	14.82738590 W
SFO1	400.1324710 MHz
SI	32768
SF	400.1300035 MHz
WDW	EM
SSB	0
LB	0.30 Hz
GB	0
PC	1.00

4CF3PhBH2PMe3

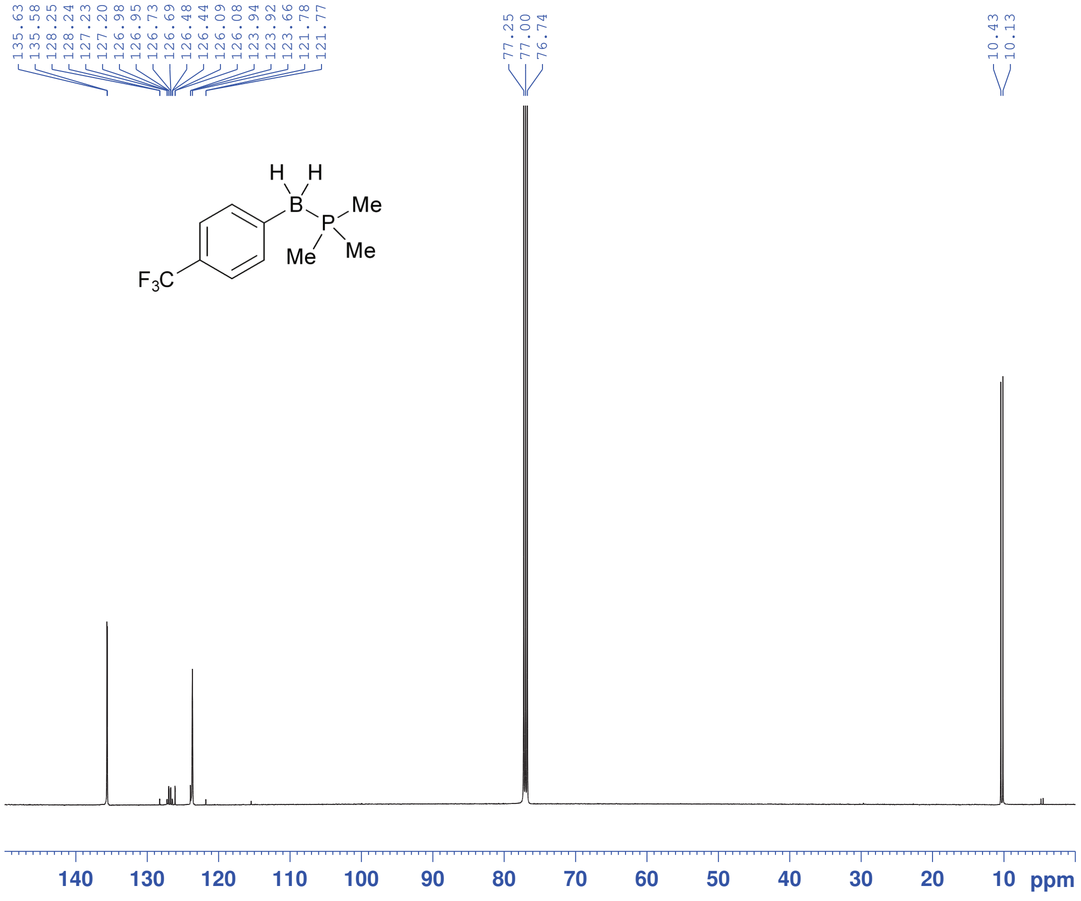

IBB-nmr Analysis

NAME	Mar07-2024
EXPNO	2
PROCNO	1
Date_	20240307
Time	12.21
INSTRUM	av500
PROBHD	5 mm CPDCH 13C
PULPROG	zgpg30
TD	65536
SOLVENT	CDCl3
NS	1024
DS	4
SWH	30030.029 Hz
FIDRES	0.458222 Hz
AQ	1.0912410 sec
RG	90.5
DW	16.650 usec
DE	20.00 usec
TE	297.9 K
D1	2.00000000 sec
D11	0.03000000 sec
TD0	1
======== CHANNEL f1 ========	
NUC1	13C
P1	10.00 usec
PL1	-5.00 dB
PL1W	14.29790783 W
SFO1	125.7703643 MHz
======== CHANNEL f2 ========	
CPDPRG2	waltz16
NUC2	1H
PCPD2	80.00 usec
PL2	-4.00 dB
PL12	14.06 dB
PL13	17.00 dB
PL2W	6.30957365 W
PL12W	0.09862794 W
PL13W	0.05011872 W
SFO2	500.1320005 MHz
SI	32768
SF	125.7577937 MHz
WDW	EM
SSB	0
LB	1.00 Hz
GB	0
PC	0.30

4CF3PhBH2PMe3

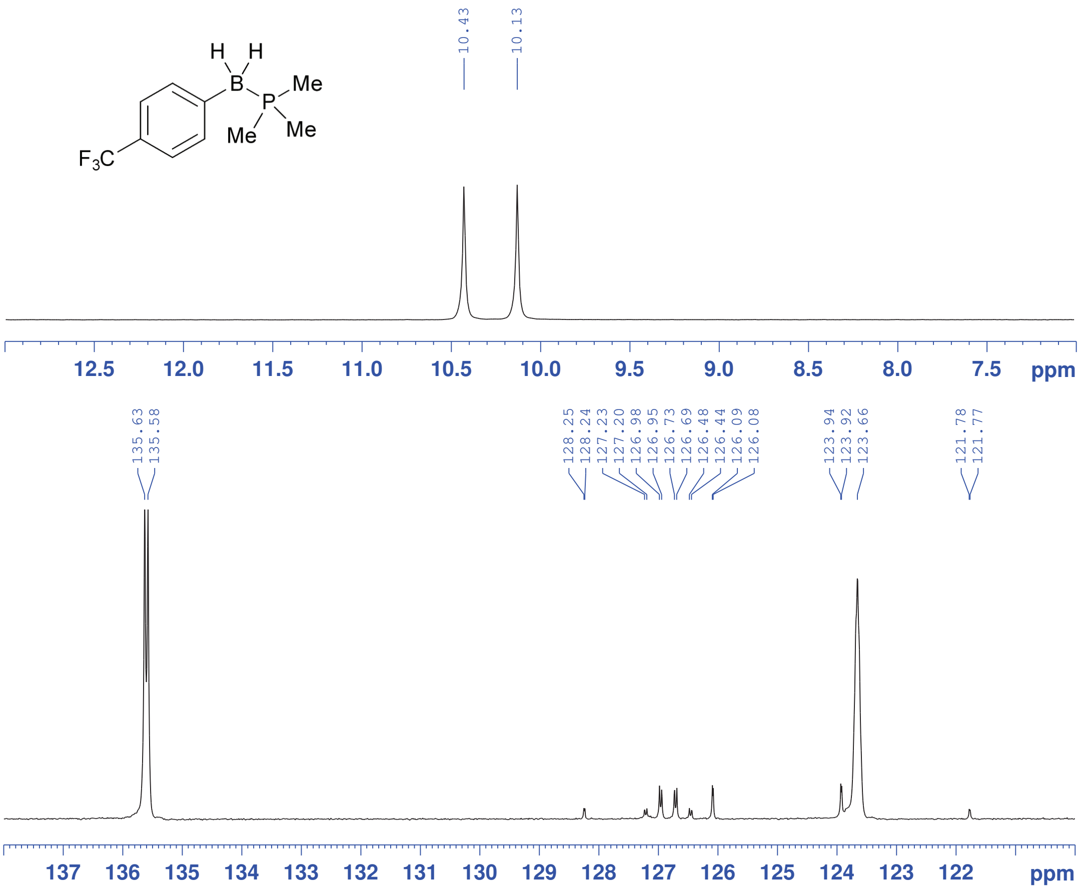

IBB-nmr Analysis

```
NAME           Mar07-2024
EXPNO                   2
PROCNO                  1
Date_            20240307
Time                12.21
INSTRUM             av500
PROBHD   5 mm CPDCH 13C
PULPROG           zgpg30
TD                  65536
SOLVENT             CDCl3
NS                   1024
DS                      4
SWH             30030.029 Hz
FIDRES           0.458222 Hz
AQ              1.0912410 sec
RG                   90.5
DW                 16.650 usec
DE                  20.00 usec
TE                  297.9 K
D1             2.00000000 sec
D11            0.03000000 sec
TD0                     1

======== CHANNEL f1 ========
NUC1                  13C
P1                  10.00 usec
PL1                 -5.00 dB
PL1W          14.29790783 W
SFO1          125.7703643 MHz

======== CHANNEL f2 ========
CPDPRG2           waltz16
NUC2                   1H
PCPD2               80.00 usec
PL2                 -4.00 dB
PL12                14.06 dB
PL13                17.00 dB
PL2W           6.30957365 W
PL12W          0.09862794 W
PL13W          0.05011872 W
SFO2          500.1320005 MHz
SI                  32768
SF            125.7577937 MHz
WDW                    EM
SSB                     0
LB                   1.00 Hz
GB                      0
PC                   0.30
```

4CF3PhBH2PMe3

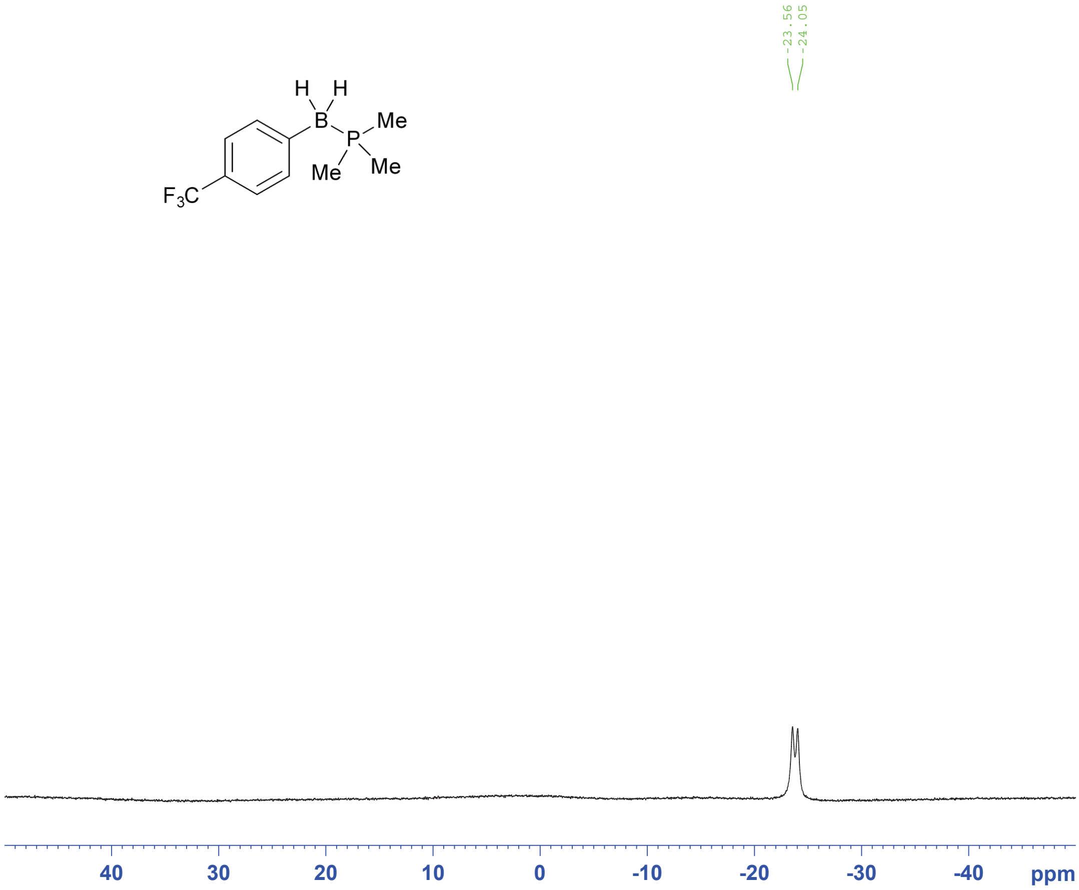

IBB-nmr Analysis

```
NAME           Dec4-2021
EXPNO                   4
PROCNO                  1
Date_            20211204
Time                12.23
INSTRUM             av400
PROBHD   5 mm PABBO BB-
PULPROG              zgig
TD                  32768
SOLVENT              DMSO
NS                    256
DS                      4
SWH             20000.000 Hz
FIDRES           0.610352 Hz
AQ              0.8192500 sec
RG                    203
DW                 25.000 usec
DE                  20.00 usec
TE                  297.3 K
D1             1.00000000 sec
D11            0.03000000 sec
TD0                     1

======== CHANNEL f1 ========
NUC1                  11B
P1                   6.30 usec
PL1                 -2.00 dB
SFO1          128.3776050 MHz

======== CHANNEL f2 ========
CPDPRG2           waltz16
NUC2                   1H
PCPD2               80.00 usec
PL2                 -1.70 dB
PL12                13.80 dB
PL2W          14.48987389 W
PL12W          0.40838012 W
SFO2          400.1316005 MHz
SI                  32768
SF            128.3776183 MHz
WDW                    EM
SSB                     0
LB                   2.00 Hz
GB                      0
PC                   1.40
```

4CF3PhBH2PMe3

IBB-nmr Analysis

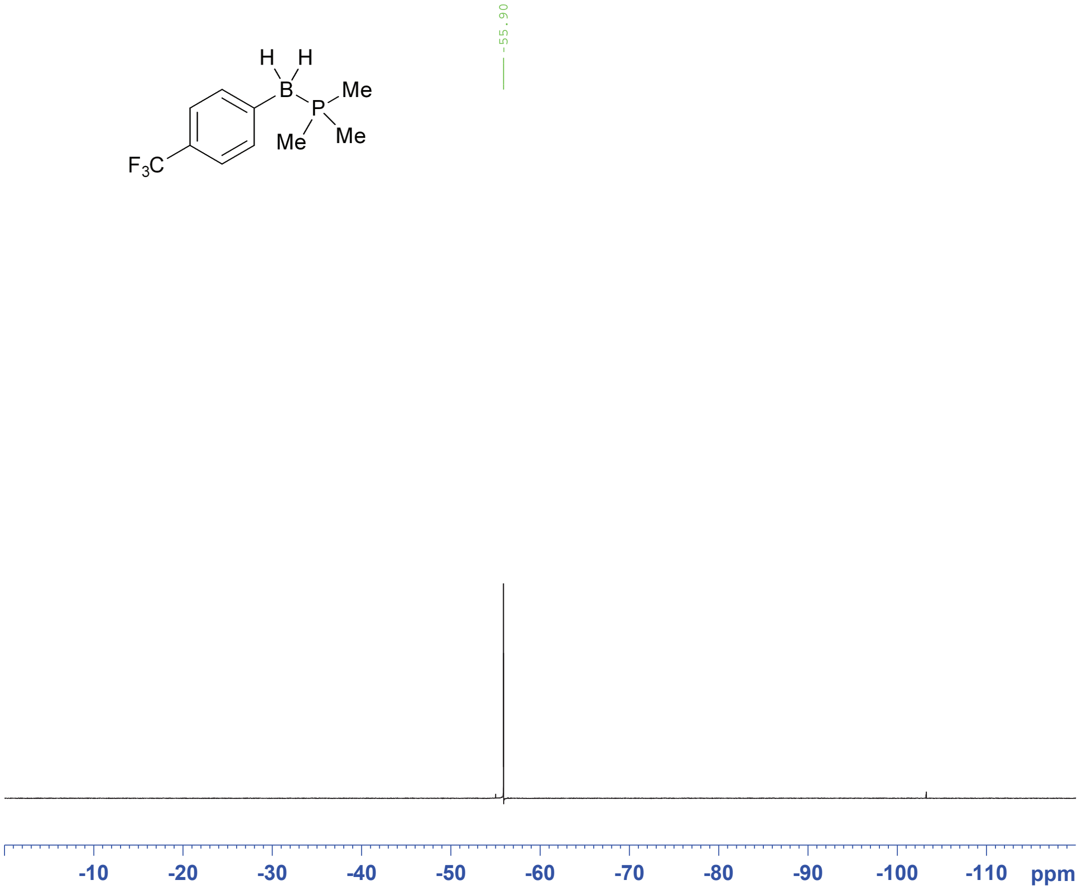


```
NAME            Dec4-2021
EXPNO                   6
PROCNO                  1
Date_            20211204
Time                12.36
INSTRUM             av400
PROBHD   5 mm PABBO BB-
PULPROG          zgfhigqn
TD                 131072
SOLVENT              DMSO
NS                      8
DS                      4
SWH            89285.711 Hz
FIDRES          0.681196 Hz
AQ             0.7340532 sec
RG                    203
DW                  5.600 usec
DE                   6.50 usec
TE                  296.7 K
D1             1.00000000 sec
D11            0.03000000 sec
D12            0.00002000 sec
TD0                     1

======== CHANNEL f1 ========
NUC1                  19F
P1                  19.50 usec
PL1                 -4.00 dB
PL1W          16.40489006 W
SFO1          376.4607164 MHz

======== CHANNEL f2 ========
CPDPRG2           waltz16
NUC2                   1H
PCPD2              112.00 usec
PL2                 -4.00 dB
PL12                18.00 dB
PL2W          24.60733604 W
PL12W          0.15526180 W
SFO2          400.1316005 MHz
SI                  65536
SF            376.4966788 MHz
WDW                    EM
SSB                     0
LB                   0.30 Hz
GB                      0
PC                   1.00
```

4CF3PhBH2PMe3

IBB-nmr Analysis

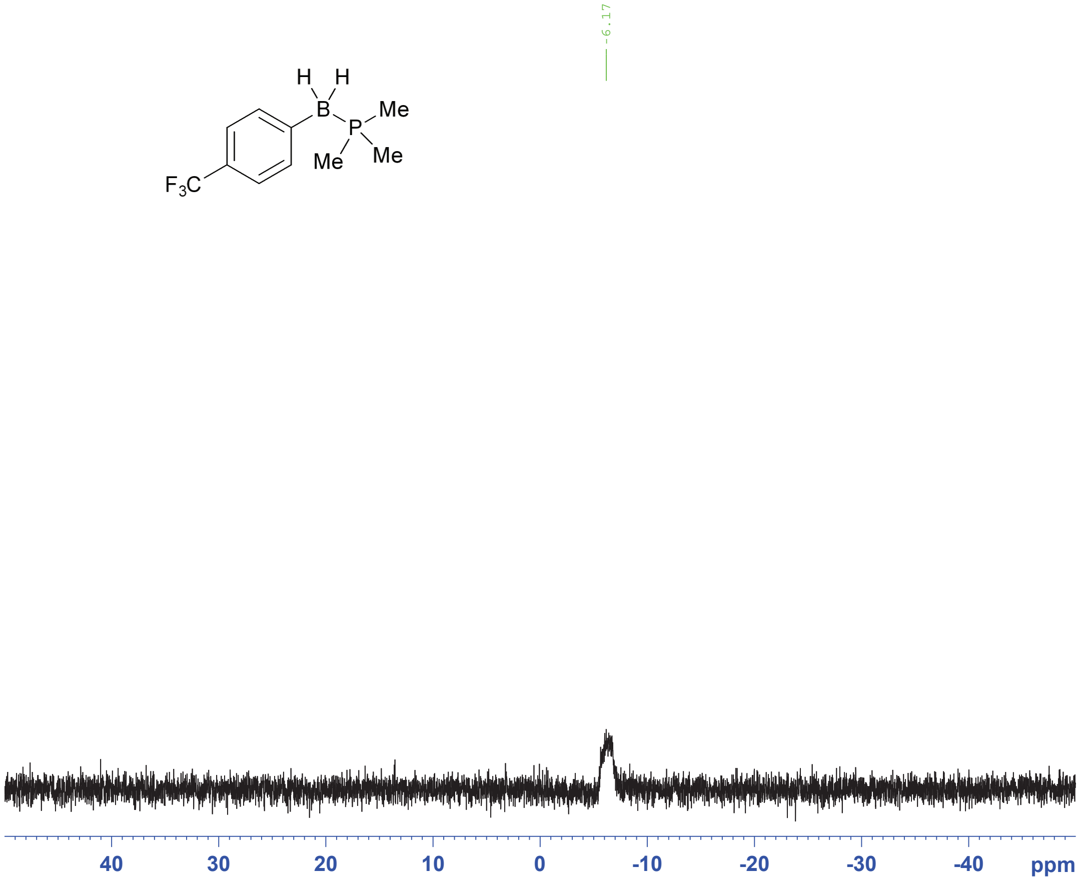


```
NAME            Dec4-2021
EXPNO                   3
PROCNO                  1
Date_            20211204
Time                12.12
INSTRUM             av400
PROBHD   5 mm PABBO BB-
PULPROG              zg30
TD                  65536
SOLVENT              DMSO
NS                     64
DS                      4
SWH            64102.563 Hz
FIDRES          0.978127 Hz
AQ             0.5112308 sec
RG                    203
DW                  7.800 usec
DE                   6.50 usec
TE                  296.6 K
D1             2.00000000 sec
TD0                     1

======== CHANNEL f1 ========
NUC1                  31P
P1                  10.00 usec
PL1                 -0.70 dB
PL1W          30.10233498 W
SFO1          161.9674942 MHz
SI                  32768
SF            161.9754962 MHz
WDW                    EM
SSB                     0
LB                   1.00 Hz
GB                      0
PC                   1.40
```

4CF3PhBH2Me2Ph 7-13Fr

B-4-(Trifluoromethyl)phenyl dimethyl(phenyl)phosphine–borane (29)

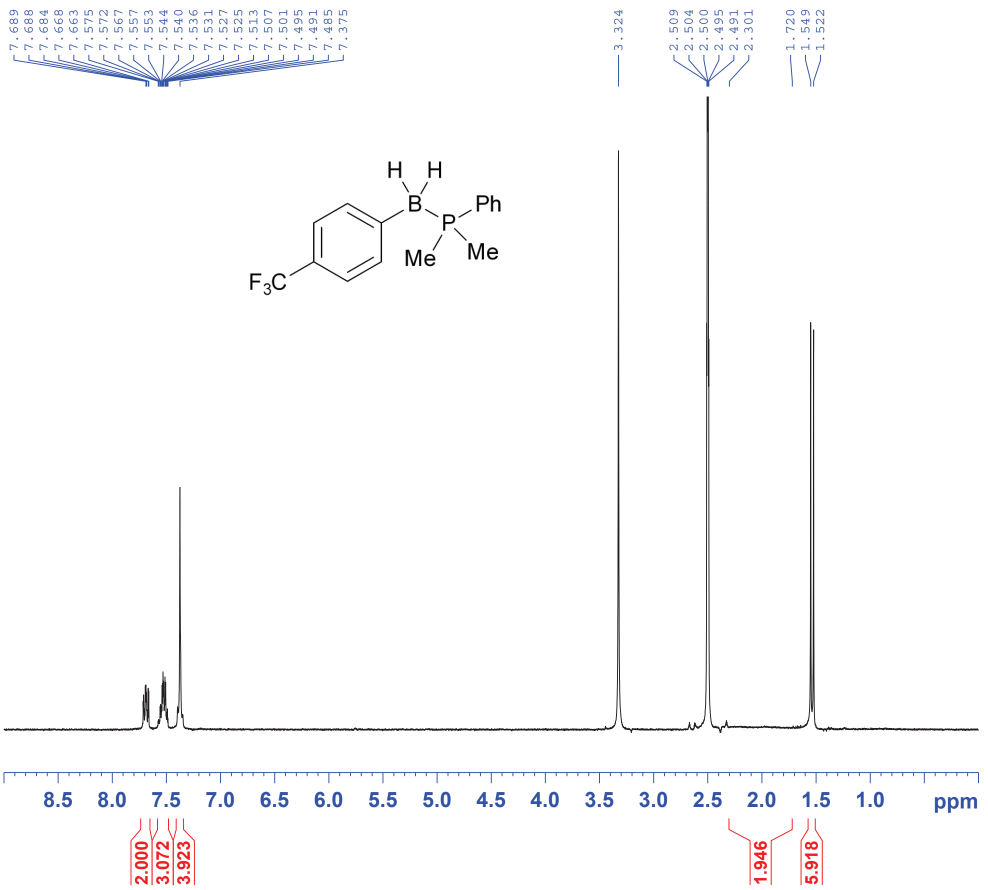

IBB-nmr Analysis

```
NAME            Feb6-2022
EXPNO                   5
PROCNO                  1
Date_            20220206
Time                14.34
INSTRUM             av400
PROBHD   5 mm PABBO BB-
PULPROG              zg30
TD                  65536
SOLVENT              DMSO
NS                     16
DS                      2
SWH              8223.685 Hz
FIDRES           0.125483 Hz
AQ              3.9846387 sec
RG                    203
DW                 60.800 usec
DE                   6.50 usec
TE                  296.2 K
D1             1.00000000 sec
TD0                     1

======== CHANNEL f1 ========
NUC1                   1H
P1                  14.00 usec
PL1                 -1.80 dB
PL1W          14.82738590 W
SFO1          400.1324710 MHz
SI                  32768
SF            400.1300033 MHz
WDW                    EM
SSB                     0
LB                   0.30 Hz
GB                      0
PC                   1.00
```

4CF3PhBH2PPhMe2

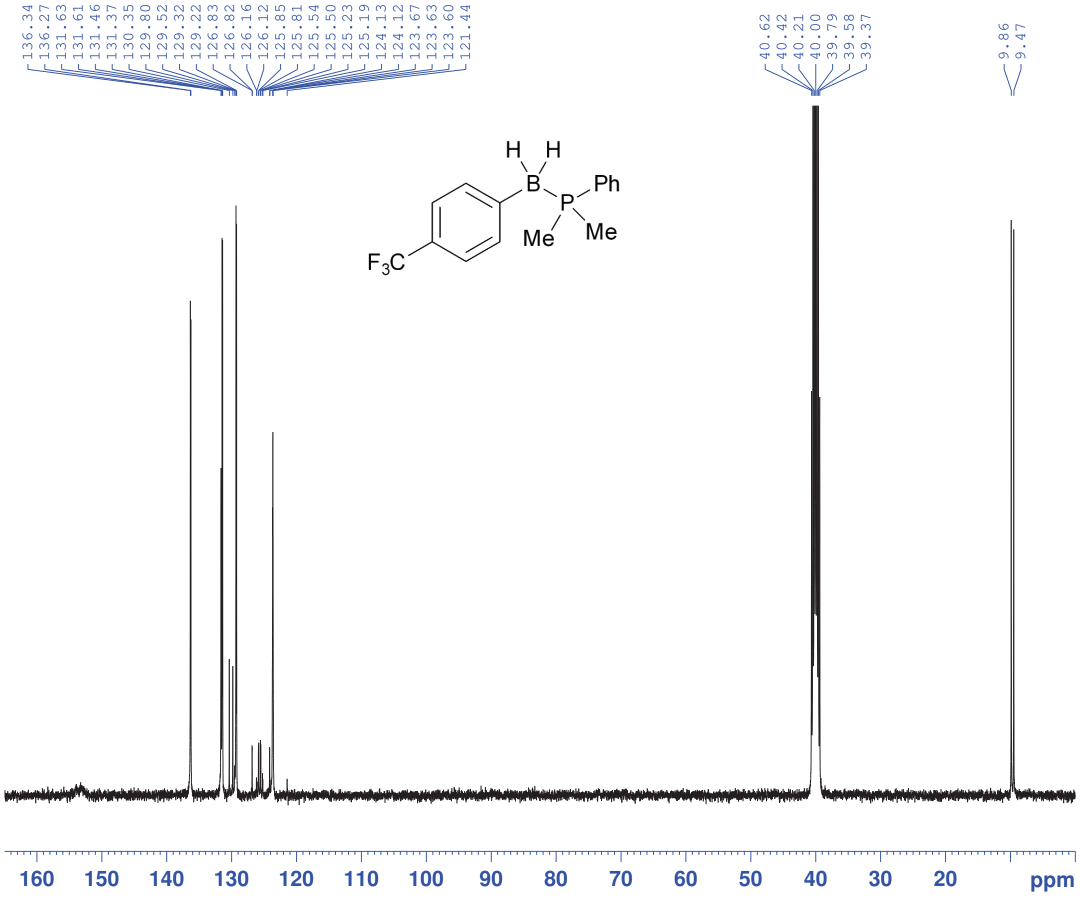

IBB-nmr Analysis

```
NAME            Aug16-2023
EXPNO                    1
PROCNO                   1
Date_             20230816
Time                 20.49
INSTRUM              av400
PROBHD    5 mm PABBO BB-
PULPROG            zgpg30
TD                   65536
SOLVENT               DMSO
NS                    2048
DS                       2
SWH              24038.461 Hz
FIDRES            0.366798 Hz
AQ               1.3631988 sec
RG                     128
DW                  20.800 usec
DE                    6.50 usec
TE                   298.6 K
D1              2.00000000 sec
D11             0.03000000 sec
TD0                      1

======== CHANNEL f1 ========
NUC1                   13C
P1                    9.00 usec
PL1                  -3.00 dB
PL1W           73.17333984 W
SFO1           100.6228298 MHz

======== CHANNEL f2 ========
CPDPRG2            waltz16
NUC2                    1H
PCPD2                80.00 usec
PL2                  -1.70 dB
PL12                 13.80 dB
PL13                 13.80 dB
PL2W           14.48987389 W
PL12W           0.40838012 W
PL13W           0.40838012 W
SFO2           400.1316005 MHz
SI                   32768
SF             100.6127689 MHz
WDW                     EM
SSB                      0
LB                    1.00 Hz
GB                       0
PC                    0.30
```

4CF3PhBH2PPhMe2

IBB-nmr Analysis

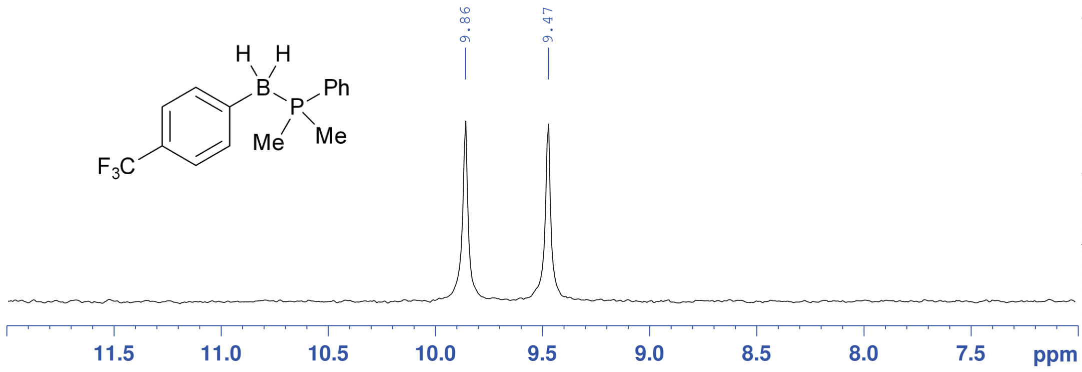

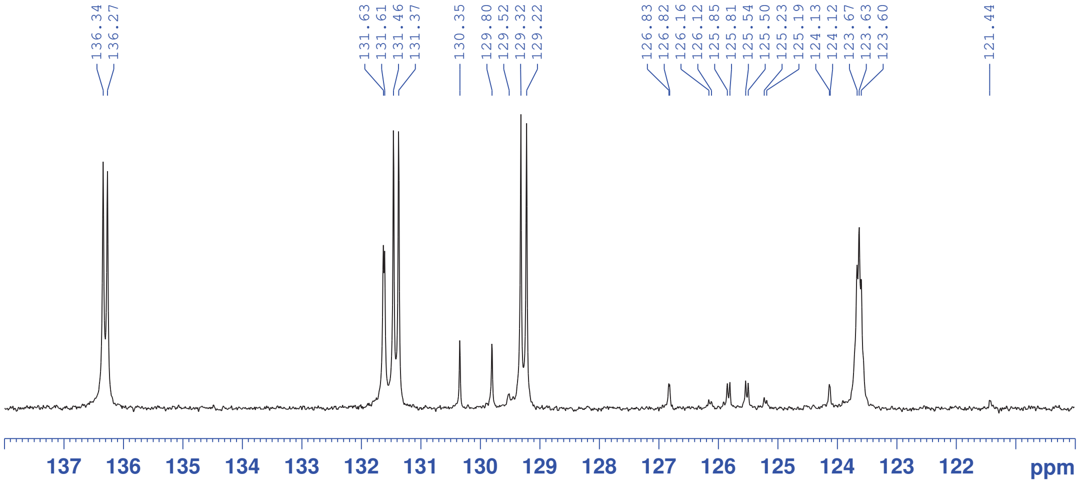


```
NAME          Aug16-2023
EXPNO                  1
PROCNO                 1
Date_           20230816
Time               20.49
INSTRUM            av400
PROBHD   5 mm PABBO BB-
PULPROG          zgpg30
TD                 65536
SOLVENT             DMSO
NS                  2048
DS                     2
SWH            24038.461 Hz
FIDRES          0.366798 Hz
AQ             1.3631988 sec
RG                   128
DW                20.800 usec
DE                  6.50 usec
TE                 298.6 K
D1            2.00000000 sec
D11           0.03000000 sec
TD0                    1

======== CHANNEL f1 ========
NUC1                 13C
P1                  9.00 usec
PL1                -3.00 dB
PL1W         73.17333984 W
SFO1         100.6228298 MHz

======== CHANNEL f2 ========
CPDPRG2          waltz16
NUC2                  1H
PCPD2              80.00 usec
PL2                -1.70 dB
PL12               13.80 dB
PL13               13.80 dB
PL2W         14.48987389 W
PL12W         0.40838012 W
PL13W         0.40838012 W
SFO2         400.1316005 MHz
SI                 32768
SF           100.6127689 MHz
WDW                   EM
SSB                    0
LB                  1.00 Hz
GB                     0
PC                  0.30
```

4CF3PhBH2Me2Ph 7-13Fr

IBB-nmr Analysis

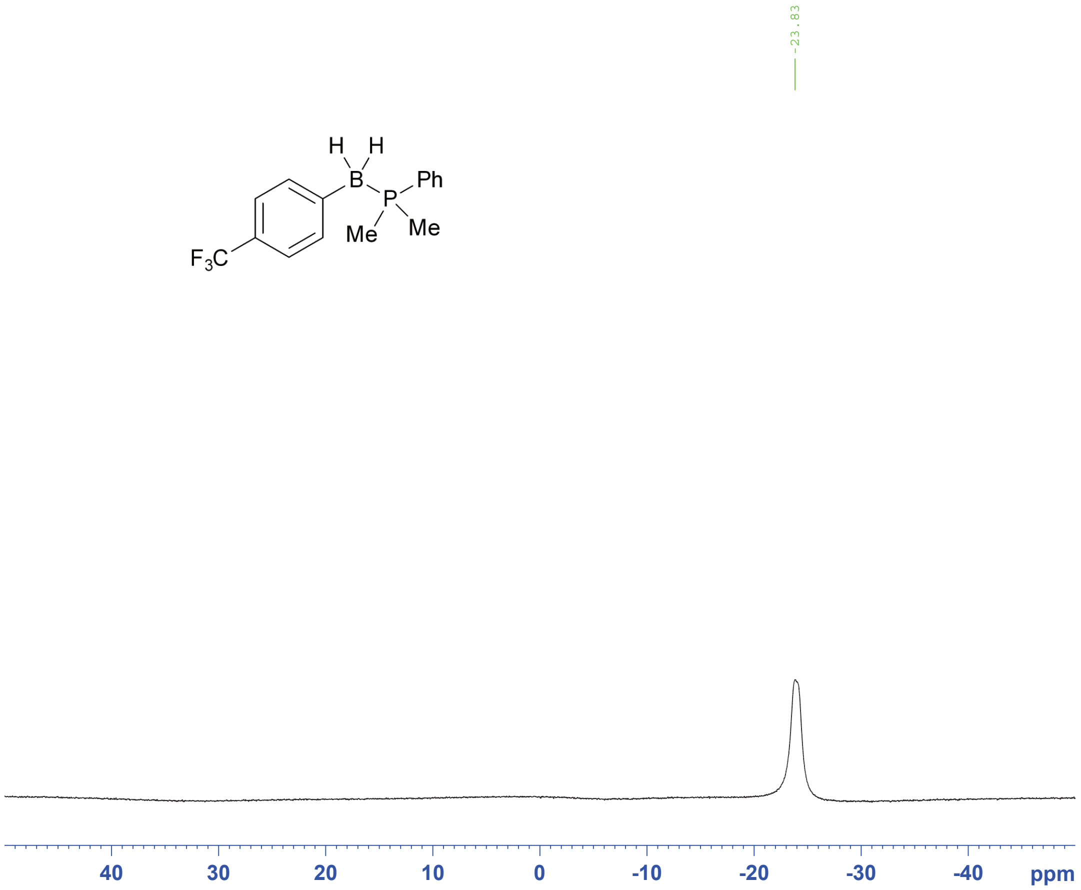


```
NAME           Feb6-2022
EXPNO                 14
PROCNO                 1
Date_           20220206
Time               23.03
INSTRUM            av400
PROBHD   5 mm PABBO BB-
PULPROG             zgig
TD                 32768
SOLVENT             DMSO
NS                   512
DS                     4
SWH            20000.000 Hz
FIDRES          0.610352 Hz
AQ             0.8192500 sec
RG                   203
DW                25.000 usec
DE                 20.00 usec
TE                 297.1 K
D1            1.00000000 sec
D11           0.03000000 sec
TD0                    1

======== CHANNEL f1 ========
NUC1                 11B
P1                  6.30 usec
PL1                -2.00 dB
SFO1         128.3776050 MHz

======== CHANNEL f2 ========
CPDPRG2          waltz16
NUC2                  1H
PCPD2              80.00 usec
PL2                -1.70 dB
PL12               13.80 dB
PL2W         14.48987389 W
PL12W         0.40838012 W
SFO2         400.1316005 MHz
SI                 32768
SF           128.3776183 MHz
WDW                   EM
SSB                    0
LB                  2.00 Hz
GB                     0
PC                  1.40
```

4CF3PhBH2Me2Ph 7-13Fr

IBB-nmr Analysis

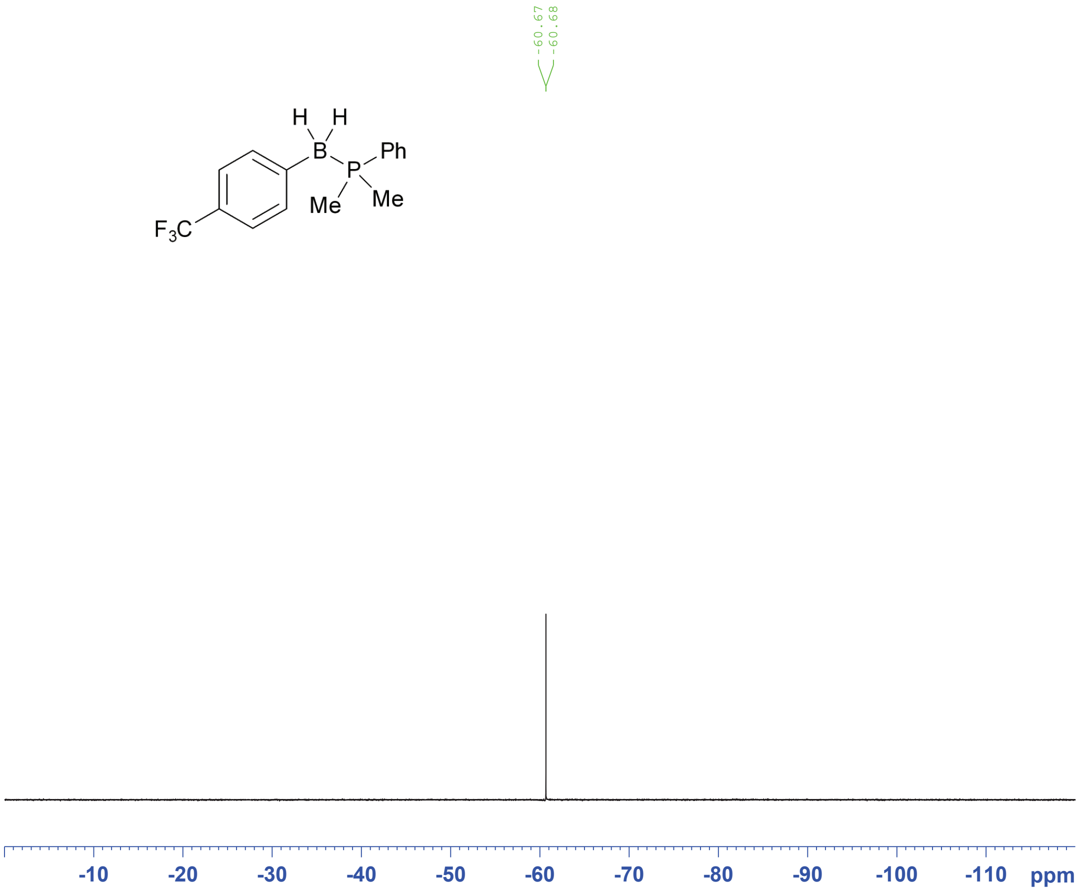

NAME	Feb6-2022
EXPNO	6
PROCNO	1
Date_	20220206
Time	14.40
INSTRUM	av400
PROBHD	5 mm PABBO BB-
PULPROG	zgfhigqn
TD	131072
SOLVENT	DMSO
NS	8
DS	4
SWH	89285.711 Hz
FIDRES	0.681196 Hz
AQ	0.7340532 sec
RG	203
DW	5.600 usec
DE	6.50 usec
TE	296.3 K
D1	1.00000000 sec
D11	0.03000000 sec
D12	0.00002000 sec
TD0	1
======== CHANNEL f1 ========	
NUC1	19F
P1	19.50 usec
PL1	-4.00 dB
PL1W	16.40489006 W
SFO1	376.4607164 MHz
======== CHANNEL f2 ========	
CPDPRG2	waltz16
NUC2	1H
PCPD2	112.00 usec
PL2	-4.00 dB
PL12	18.00 dB
PL2W	24.60733604 W
PL12W	0.15526180 W
SFO2	400.1316005 MHz
SI	65536
SF	376.4984617 MHz
WDW	EM
SSB	0
LB	0.30 Hz
GB	0
PC	1.00

4CF3PhBH2Me2Ph 7-13Fr

IBB-nmr Analysis

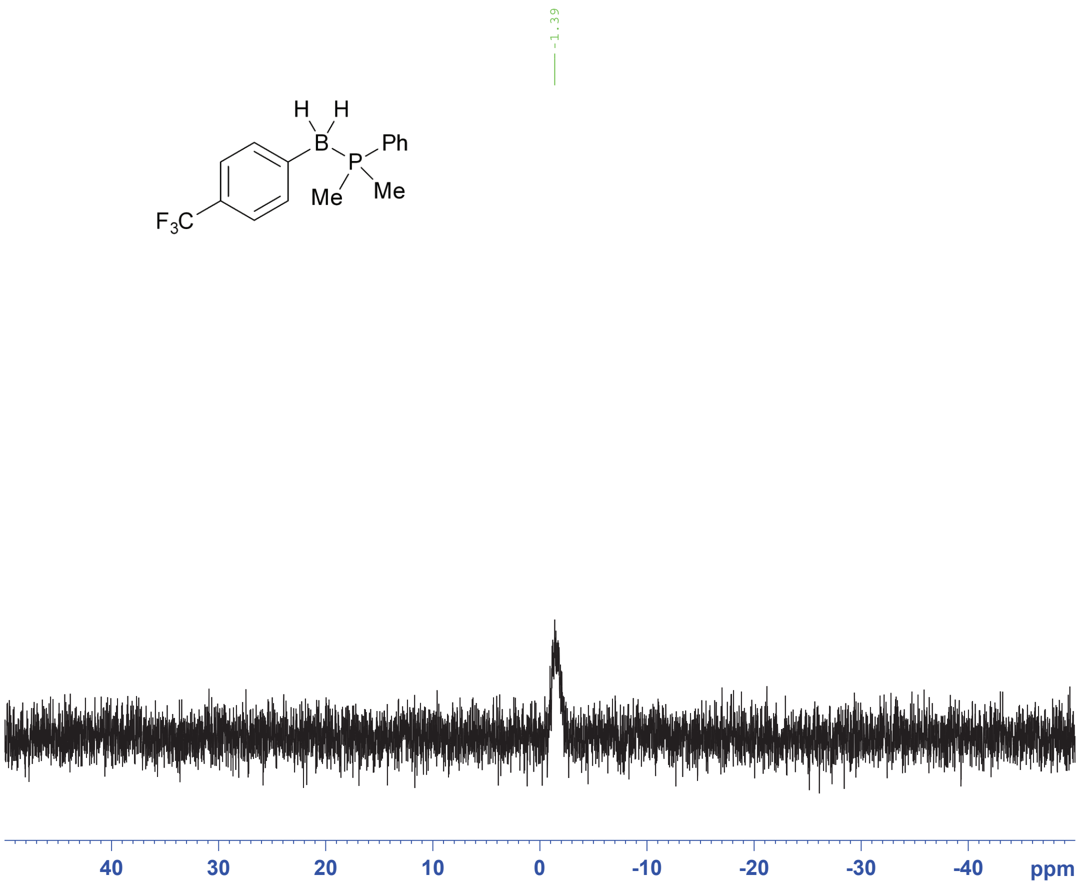

NAME	Feb6-2022
EXPNO	9
PROCNO	1
Date_	20220206
Time	15.10
INSTRUM	av400
PROBHD	5 mm PABBO BB-
PULPROG	zg30
TD	65536
SOLVENT	DMSO
NS	256
DS	4
SWH	64102.563 Hz
FIDRES	0.978127 Hz
AQ	0.5112308 sec
RG	203
DW	7.800 usec
DE	6.50 usec
TE	296.1 K
D1	2.00000000 sec
TD0	1
======== CHANNEL f1 ========	
NUC1	31P
P1	10.00 usec
PL1	-0.70 dB
PL1W	30.10233498 W
SFO1	161.9674942 MHz
SI	32768
SF	161.9754962 MHz
WDW	EM
SSB	0
LB	1.00 Hz
GB	0
PC	1.40

4CF3PhBH2PEt3 4-9Fr

B-4-(Trifluoromethyl)phenyl triethylphosphine–borane (30)

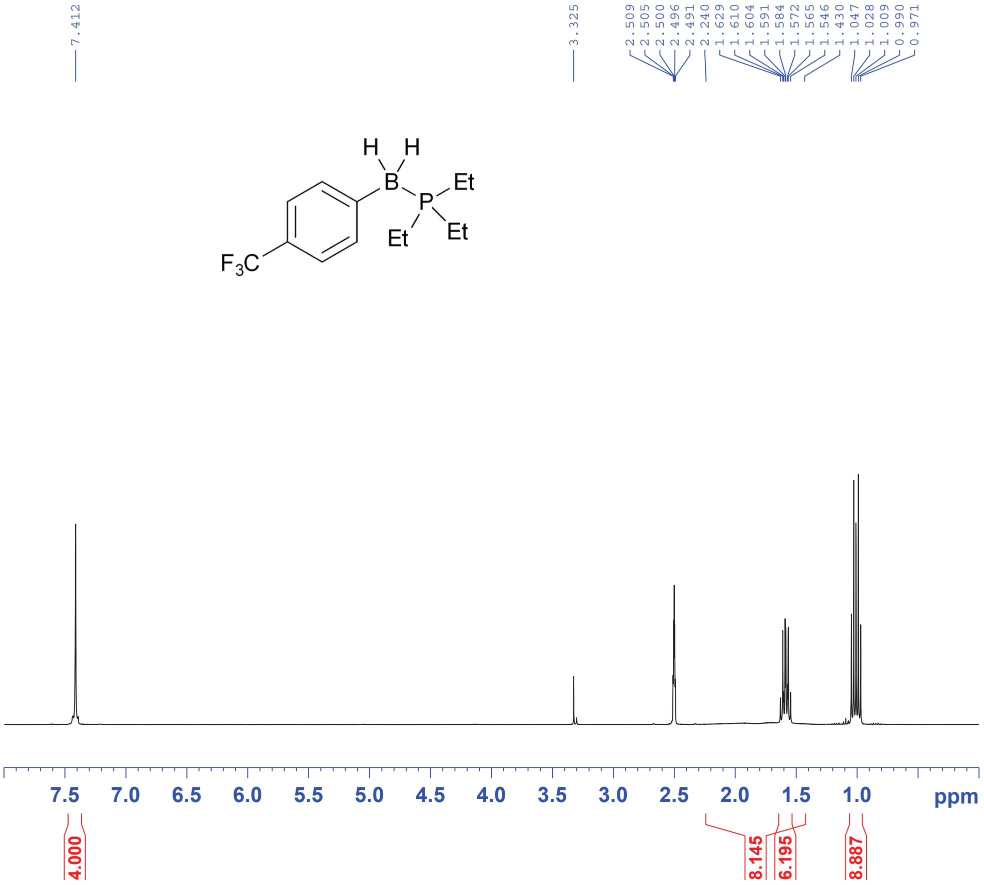

IBB-nmr Analysis

NAME	Feb2-2022
EXPNO	1
PROCNO	1
Date_	20220202
Time	22.04
INSTRUM	av400
PROBHD	5 mm PABBO BB-
PULPROG	zg30
TD	65536
SOLVENT	DMSO
NS	16
DS	2
SWH	8223.685 Hz
FIDRES	0.125483 Hz
AQ	3.9846387 sec
RG	203
DW	60.800 usec
DE	6.50 usec
TE	296.3 K
D1	1.00000000 sec
TD0	1
======== CHANNEL f1 ========	
NUC1	1H
P1	14.00 usec
PL1	-1.80 dB
PL1W	14.82738590 W
SFO1	400.1324710 MHz
SI	32768
SF	400.1300026 MHz
WDW	EM
SSB	0
LB	0.30 Hz
GB	0
PC	1.00

4CF3PhBH2PEt3

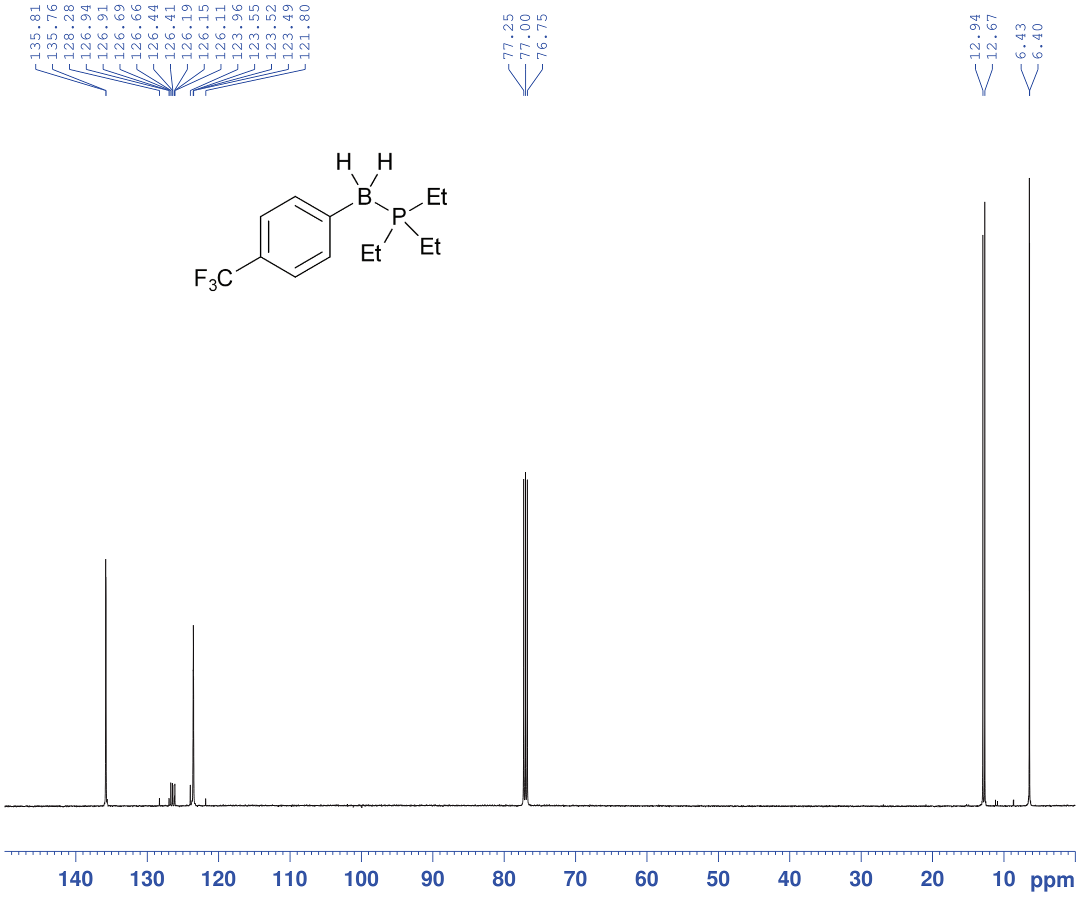

IBB-nmr Analysis

NAME	Mar07-2024
EXPNO	7
PROCNO	1
Date_	20240308
Time	0.11
INSTRUM	av500
PROBHD	5 mm CPDCH 13C
PULPROG	zgpg30
TD	65536
SOLVENT	CDCl3
NS	64
DS	4
SWH	30030.029 Hz
FIDRES	0.458222 Hz
AQ	1.0912410 sec
RG	40.3
DW	16.650 usec
DE	20.00 usec
TE	298.1 K
D1	2.00000000 sec
D11	0.03000000 sec
TD0	1
======== CHANNEL f1 ========	
NUC1	13C
P1	10.00 usec
PL1	-5.00 dB
PL1W	14.29790783 W
SFO1	125.7703643 MHz
======== CHANNEL f2 ========	
CPDPRG2	waltz16
NUC2	1H
PCPD2	80.00 usec
PL2	-4.00 dB
PL12	14.06 dB
PL13	17.00 dB
PL2W	6.30957365 W
PL12W	0.09862794 W
PL13W	0.05011872 W
SFO2	500.1320005 MHz
SI	32768
SF	125.7577965 MHz
WDW	EM
SSB	0
LB	1.00 Hz
GB	0
PC	0.30

4CF3PhBH2PEt3

IBB-nmr Analysis

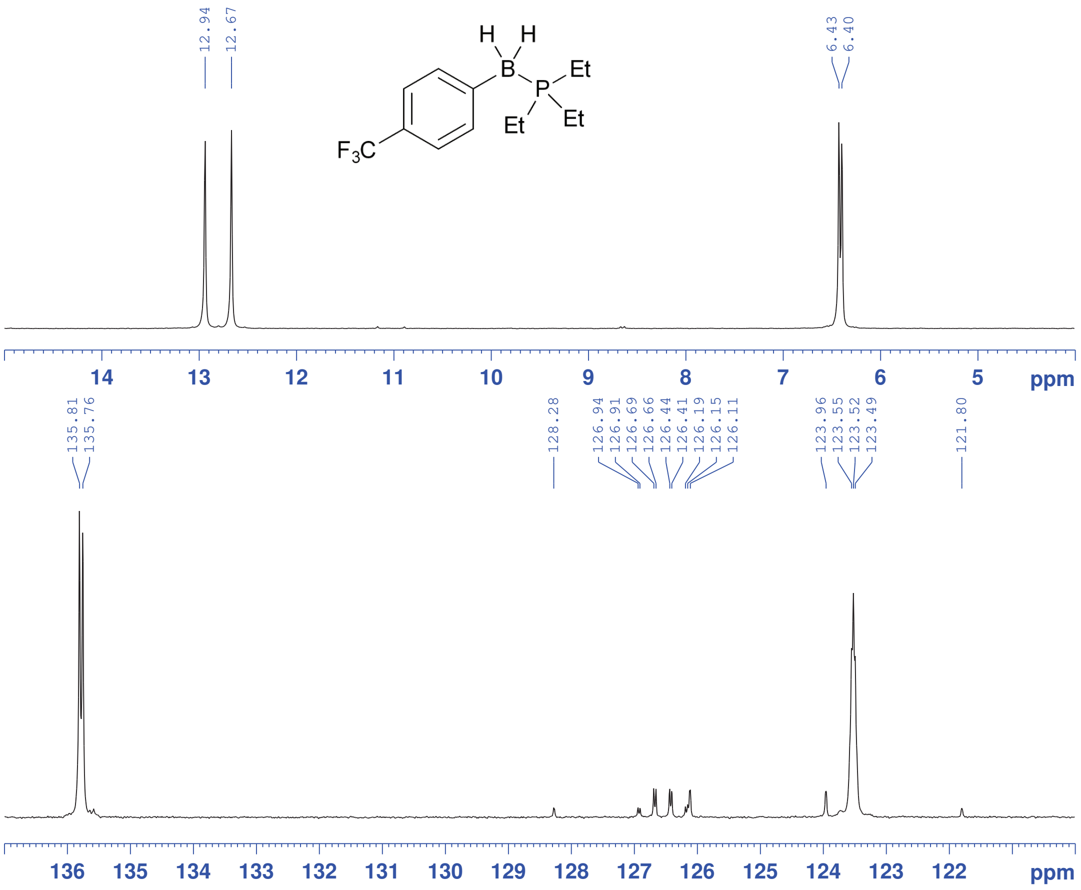


```
NAME        Mar07-2024
EXPNO                7
PROCNO               1
Date_         20240308
Time              0.11
INSTRUM          av500
PROBHD   5 mm CPDCH 13C
PULPROG         zgpg30
TD               65536
SOLVENT          CDCl3
NS                  64
DS                   4
SWH          30030.029 Hz
FIDRES        0.458222 Hz
AQ           1.0912410 sec
RG                40.3
DW              16.650 usec
DE               20.00 usec
TE               298.1 K
D1          2.00000000 sec
D11         0.03000000 sec
TD0                  1

======== CHANNEL f1 ========
NUC1               13C
P1               10.00 usec
PL1              -5.00 dB
PL1W       14.29790783 W
SFO1       125.7703643 MHz

======== CHANNEL f2 ========
CPDPRG2        waltz16
NUC2                1H
PCPD2            80.00 usec
PL2              -4.00 dB
PL12             14.06 dB
PL13             17.00 dB
PL2W        6.30957365 W
PL12W       0.09862794 W
PL13W       0.05011872 W
SFO2       500.1320005 MHz
SI               32768
SF         125.7577965 MHz
WDW                 EM
SSB                  0
LB                1.00 Hz
GB                   0
PC                0.30
```

4CF3PhBH2PEt3   4-9Fr

IBB-nmr Analysis

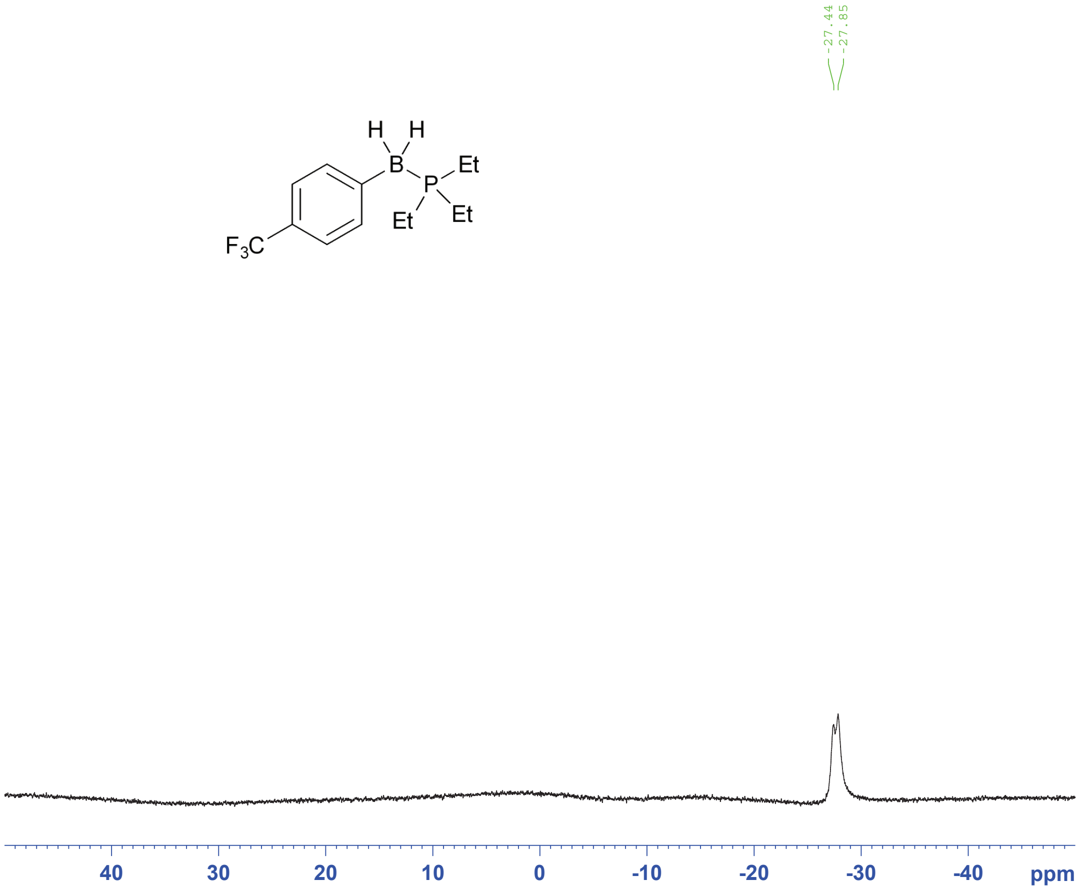


```
NAME        Feb1-2022
EXPNO                4
PROCNO               1
Date_         20220202
Time              0.13
INSTRUM          av400
PROBHD   5 mm PABBO BB-
PULPROG           zgig
TD               32768
SOLVENT           DMSO
NS                 256
DS                   4
SWH          20000.000 Hz
FIDRES        0.610352 Hz
AQ           0.8192500 sec
RG                 203
DW              25.000 usec
DE               20.00 usec
TE               297.0 K
D1          1.00000000 sec
D11         0.03000000 sec
TD0                  1

======== CHANNEL f1 ========
NUC1               11B
P1                6.30 usec
PL1              -2.00 dB
SFO1       128.3776050 MHz

======== CHANNEL f2 ========
CPDPRG2        waltz16
NUC2                1H
PCPD2            80.00 usec
PL2              -1.70 dB
PL12             13.80 dB
PL2W       14.48987389 W
PL12W       0.40838012 W
SFO2       400.1316005 MHz
SI               32768
SF         128.3776183 MHz
WDW                 EM
SSB                  0
LB                2.00 Hz
GB                   0
PC                1.40
```

4CF3PhBH2PEt3 4-9Fr

IBB-nmr Analysis

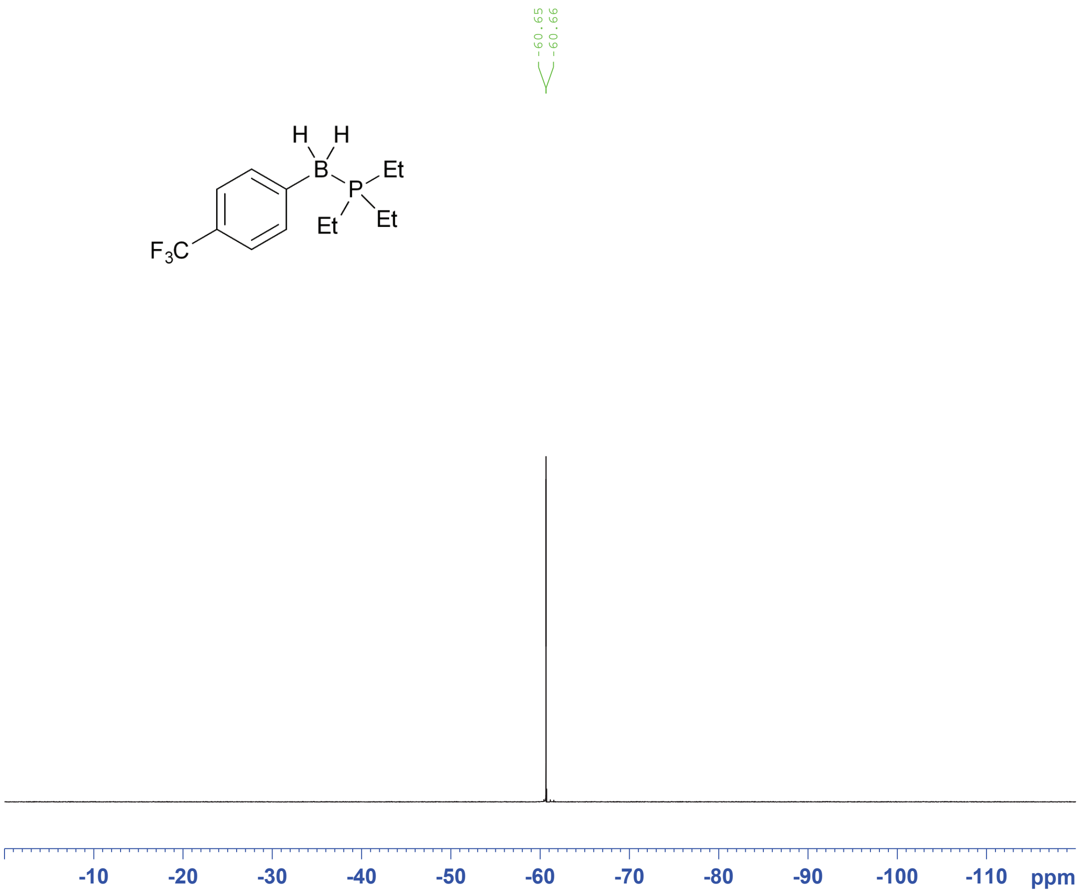

NAME	Feb2-2022
EXPNO	4
PROCNO	1
Date_	20220202
Time	22.19
INSTRUM	av400
PROBHD	5 mm PABBO BB-
PULPROG	zgfhigqn
TD	131072
SOLVENT	DMSO
NS	8
DS	4
SWH	89285.711 Hz
FIDRES	0.681196 Hz
AQ	0.7340532 sec
RG	203
DW	5.600 usec
DE	6.50 usec
TE	296.4 K
D1	1.00000000 sec
D11	0.03000000 sec
D12	0.00002000 sec
TD0	1
======== CHANNEL f1 ========	
NUC1	19F
P1	19.50 usec
PL1	-4.00 dB
PL1W	16.40489006 W
SFO1	376.4607164 MHz
======== CHANNEL f2 ========	
CPDPRG2	waltz16
NUC2	1H
PCPD2	112.00 usec
PL2	-4.00 dB
PL12	18.00 dB
PL2W	24.60733604 W
PL12W	0.15526180 W
SFO2	400.1316005 MHz
SI	65536
SF	376.4984677 MHz
WDW	EM
SSB	0
LB	0.30 Hz
GB	0
PC	1.00

4CF3PhBH2PEt3 4-9Fr

IBB-nmr Analysis

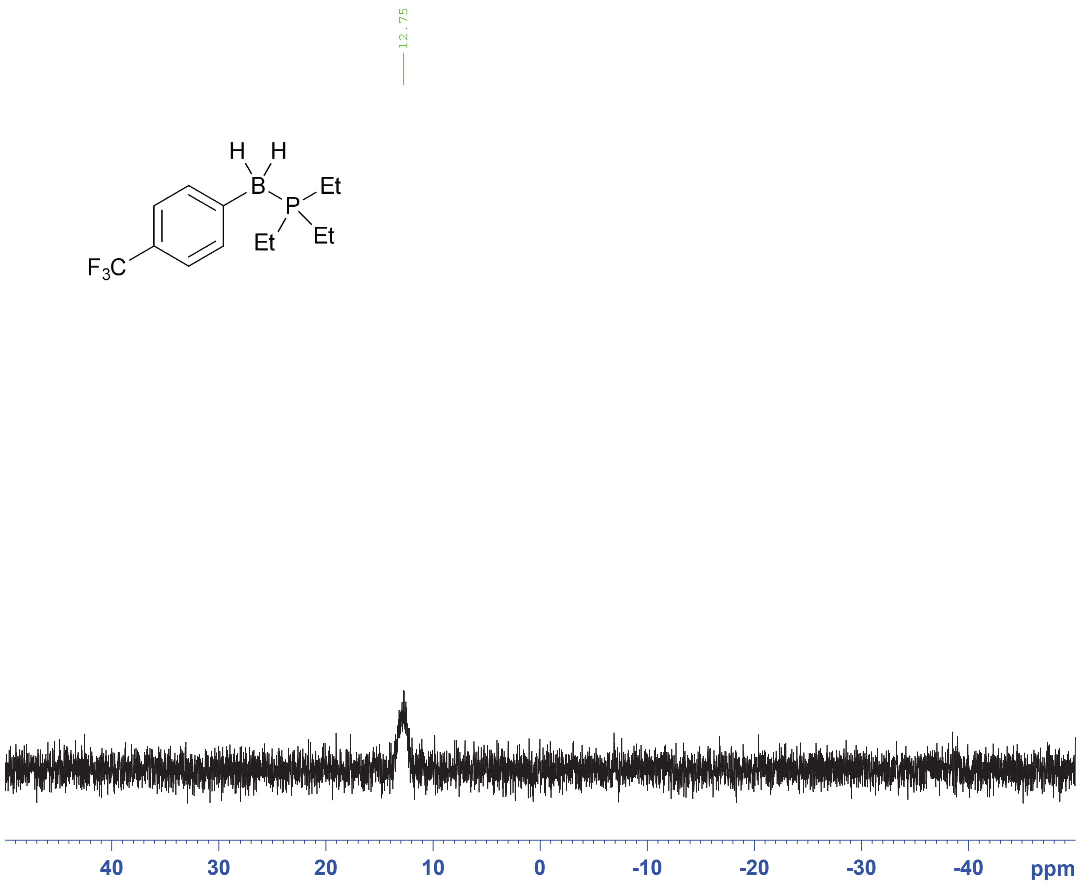

NAME	Feb1-2022
EXPNO	3
PROCNO	1
Date_	20220202
Time	0.02
INSTRUM	av400
PROBHD	5 mm PABBO BB-
PULPROG	zg30
TD	65536
SOLVENT	DMSO
NS	64
DS	4
SWH	64102.563 Hz
FIDRES	0.978127 Hz
AQ	0.5112308 sec
RG	203
DW	7.800 usec
DE	6.50 usec
TE	296.3 K
D1	2.00000000 sec
TD0	1
======== CHANNEL f1 ========	
NUC1	31P
P1	10.00 usec
PL1	-0.70 dB
PL1W	30.10233498 W
SFO1	161.9674942 MHz
SI	32768
SF	161.9754962 MHz
WDW	EM
SSB	0
LB	1.00 Hz
GB	0
PC	1.40

4CF3PhBH2Et2Ph 11-21Fr

B-4-(Trifluoromethyl)phenyl diethyl(phenyl)phosphine–borane (31)

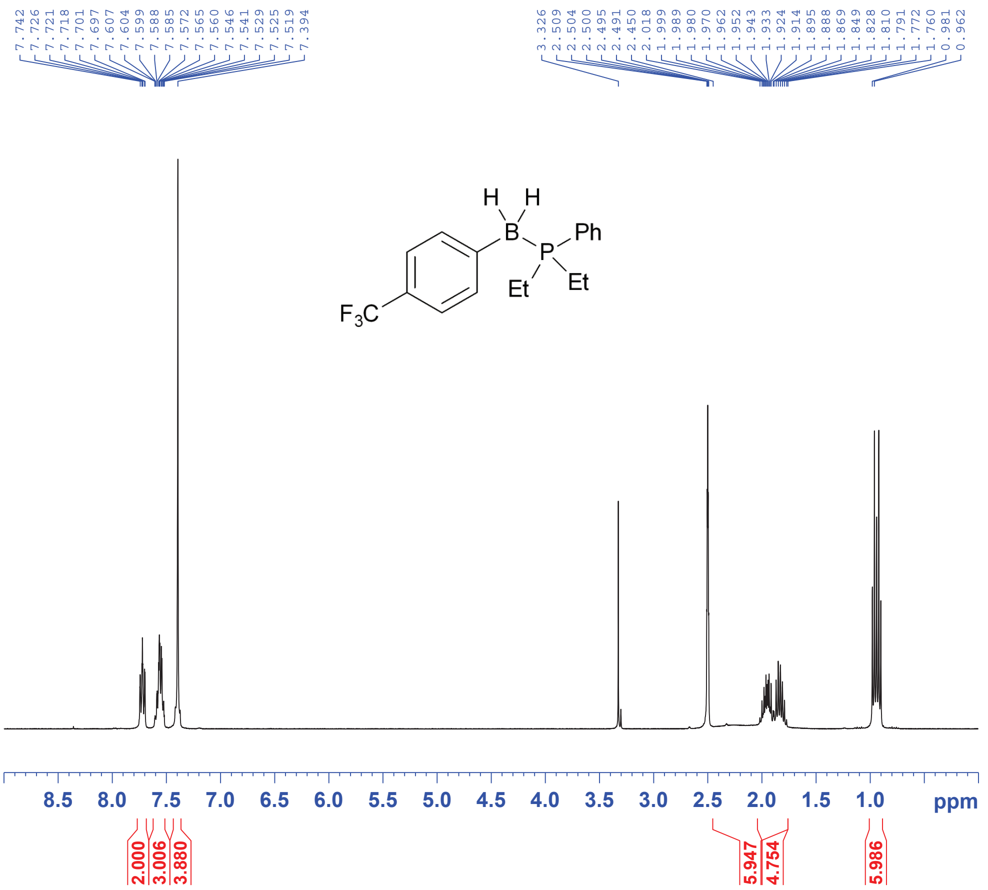

IBB-nmr Analysis

NAME	Feb11-2022
EXPNO	1
PROCNO	1
Date_	20220211
Time	18.22
INSTRUM	av400
PROBHD	5 mm PABBO BB-
PULPROG	zg30
TD	65536
SOLVENT	DMSO
NS	16
DS	2
SWH	8223.685 Hz
FIDRES	0.125483 Hz
AQ	3.9846387 sec
RG	203
DW	60.800 usec
DE	6.50 usec
TE	296.2 K
D1	1.00000000 sec
TD0	1

======== CHANNEL f1 ========

NUC1	1H
P1	14.00 usec
PL1	-1.80 dB
PL1W	14.82738590 W
SFO1	400.1324710 MHz
SI	32768
SF	400.1300036 MHz
WDW	EM
SSB	0
LB	0.30 Hz
GB	0
PC	1.00

4CF3PhBH2PEt2Ph

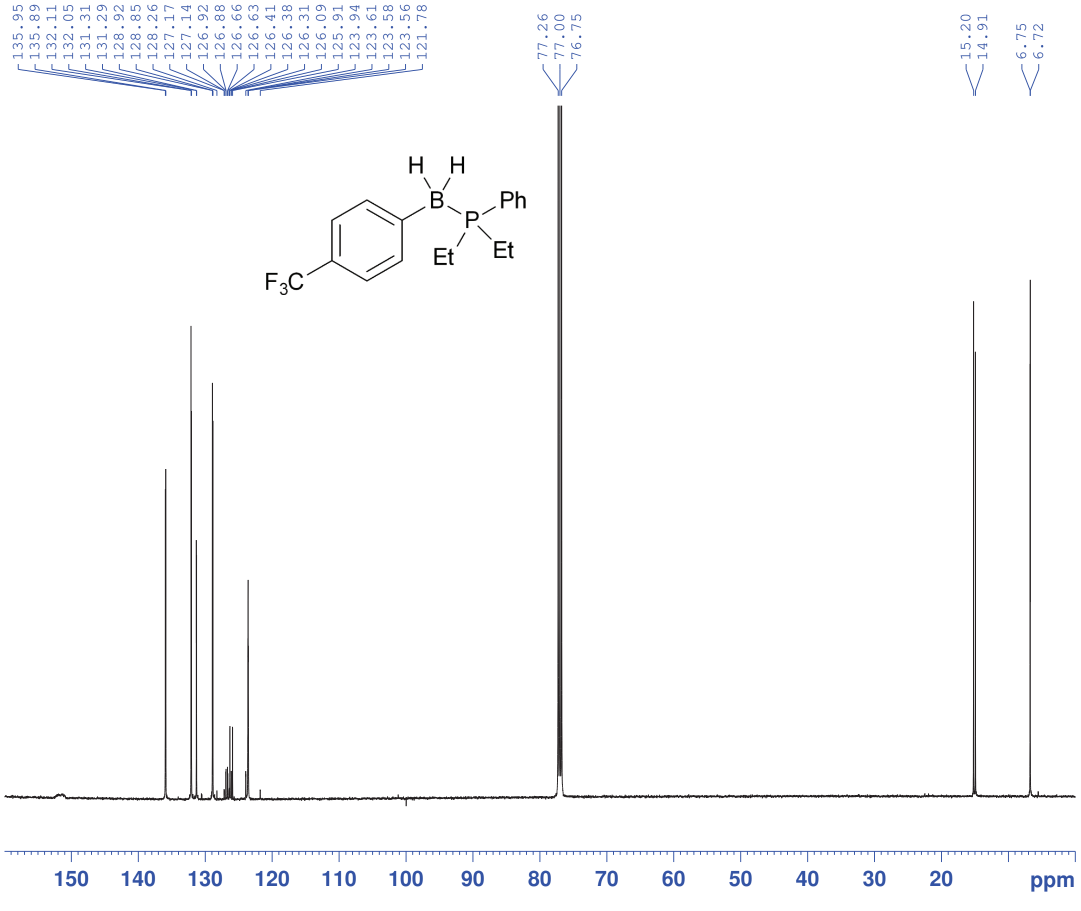

IBB-nmr Analysis

NAME	Mar07-2024
EXPNO	3
PROCNO	1
Date_	20240307
Time	13.36
INSTRUM	av500
PROBHD	5 mm CPDCH 13C
PULPROG	zgpg30
TD	65536
SOLVENT	CDCl3
NS	512
DS	4
SWH	30030.029 Hz
FIDRES	0.458222 Hz
AQ	1.0912410 sec
RG	40.3
DW	16.650 usec
DE	20.00 usec
TE	298.1 K
D1	2.00000000 sec
D11	0.03000000 sec
TD0	1

======== CHANNEL f1 ========

NUC1	13C
P1	10.00 usec
PL1	-5.00 dB
PL1W	14.29790783 W
SFO1	125.7703643 MHz

======== CHANNEL f2 ========

CPDPRG2	waltz16
NUC2	1H
PCPD2	80.00 usec
PL2	-4.00 dB
PL12	14.06 dB
PL13	17.00 dB
PL2W	6.30957365 W
PL12W	0.09862794 W
PL13W	0.05011872 W
SFO2	500.1320005 MHz
SI	32768
SF	125.7577928 MHz
WDW	EM
SSB	0
LB	1.00 Hz
GB	0
PC	0.30

4CF3PhBH2PEt2Ph

IBB-nmr Analysis

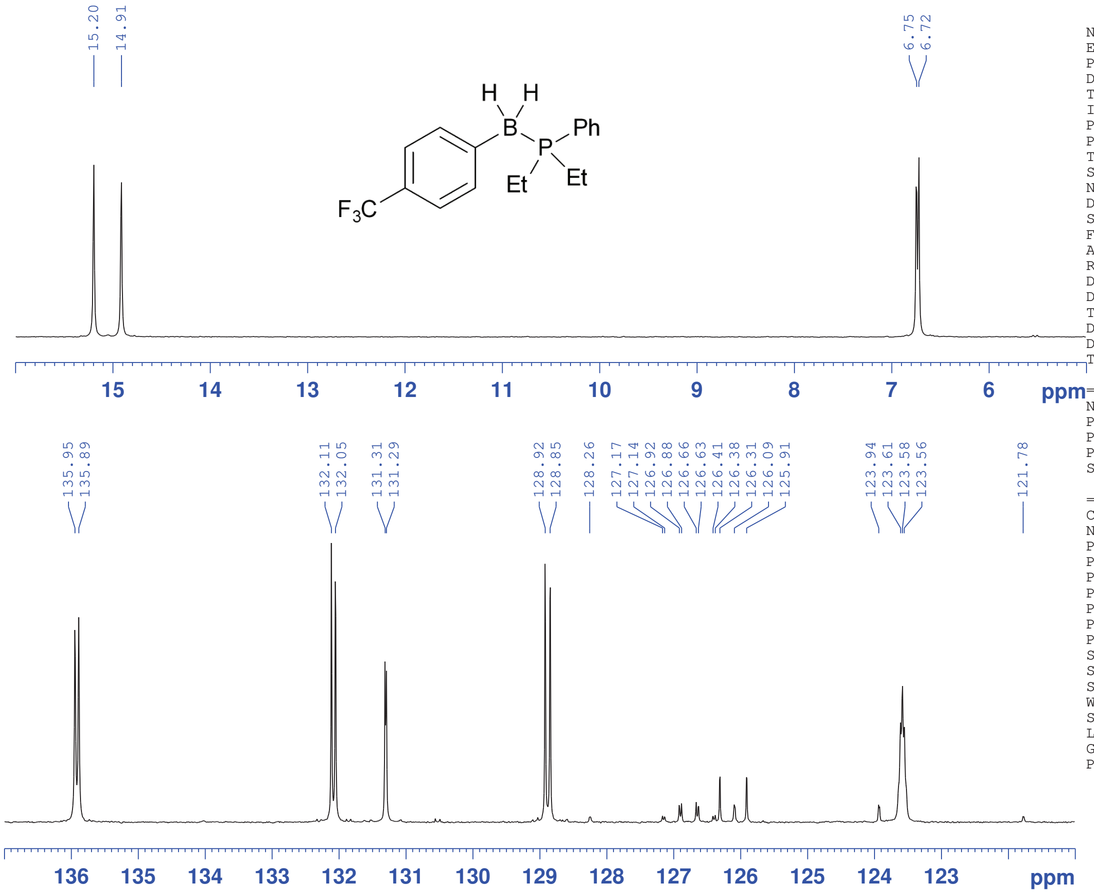

NAME Mar07-2024
EXPNO 3
PROCNO 1
Date_ 20240307
Time 13.36
INSTRUM av500
PROBHD 5 mm CPDCH 13C
PULPROG zgpg30
TD 65536
SOLVENT CDCl3
NS 512
DS 4
SWH 30030.029 Hz
FIDRES 0.458222 Hz
AQ 1.0912410 sec
RG 40.3
DW 16.650 usec
DE 20.00 usec
TE 298.1 K
D1 2.00000000 sec
D11 0.03000000 sec
TD0 1

======== CHANNEL f1 ========
NUC1 13C
P1 10.00 usec
PL1 -5.00 dB
PL1W 14.29790783 W
SFO1 125.7703643 MHz

======== CHANNEL f2 ========
CPDPRG2 waltz16
NUC2 1H
PCPD2 80.00 usec
PL2 -4.00 dB
PL12 14.06 dB
PL13 17.00 dB
PL2W 6.30957365 W
PL12W 0.09862794 W
PL13W 0.05011872 W
SFO2 500.1320005 MHz
SI 32768
SF 125.7577928 MHz
WDW EM
SSB 0
LB 1.00 Hz
GB 0
PC 0.30

4CF3PhBH2Et2Ph 11-21Fr

IBB-nmr Analysis

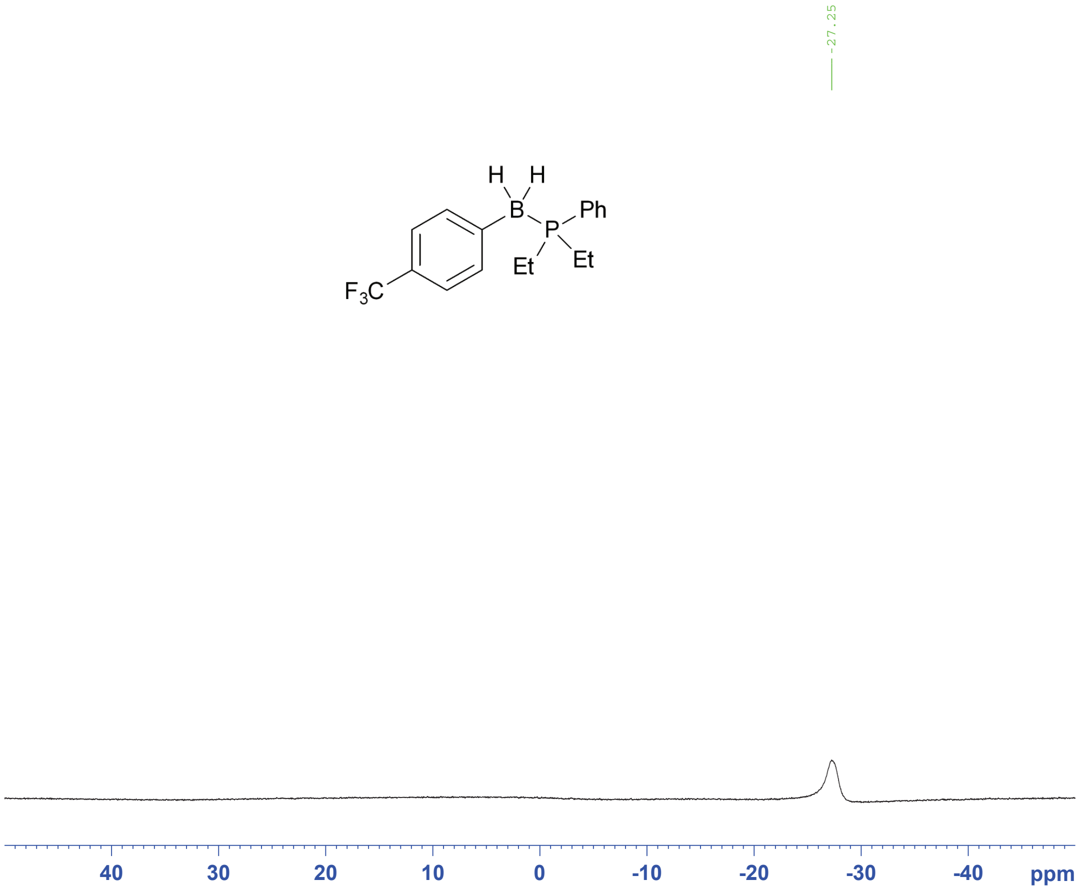

NAME Feb11-2022
EXPNO 4
PROCNO 1
Date_ 20220211
Time 18.40
INSTRUM av400
PROBHD 5 mm PABBO BB-
PULPROG zgig
TD 32768
SOLVENT DMSO
NS 128
DS 4
SWH 20000.000 Hz
FIDRES 0.610352 Hz
AQ 0.8192500 sec
RG 203
DW 25.000 usec
DE 20.00 usec
TE 296.9 K
D1 1.00000000 sec
D11 0.03000000 sec
TD0 1

======== CHANNEL f1 ========
NUC1 11B
P1 6.30 usec
PL1 -2.00 dB
SFO1 128.3776050 MHz

======== CHANNEL f2 ========
CPDPRG2 waltz16
NUC2 1H
PCPD2 80.00 usec
PL2 -1.70 dB
PL12 13.80 dB
PL2W 14.48987389 W
PL12W 0.40838012 W
SFO2 400.1316005 MHz
SI 32768
SF 128.3776183 MHz
WDW EM
SSB 0
LB 2.00 Hz
GB 0
PC 1.40

4CF3PhBH2Et2Ph 11-21Fr

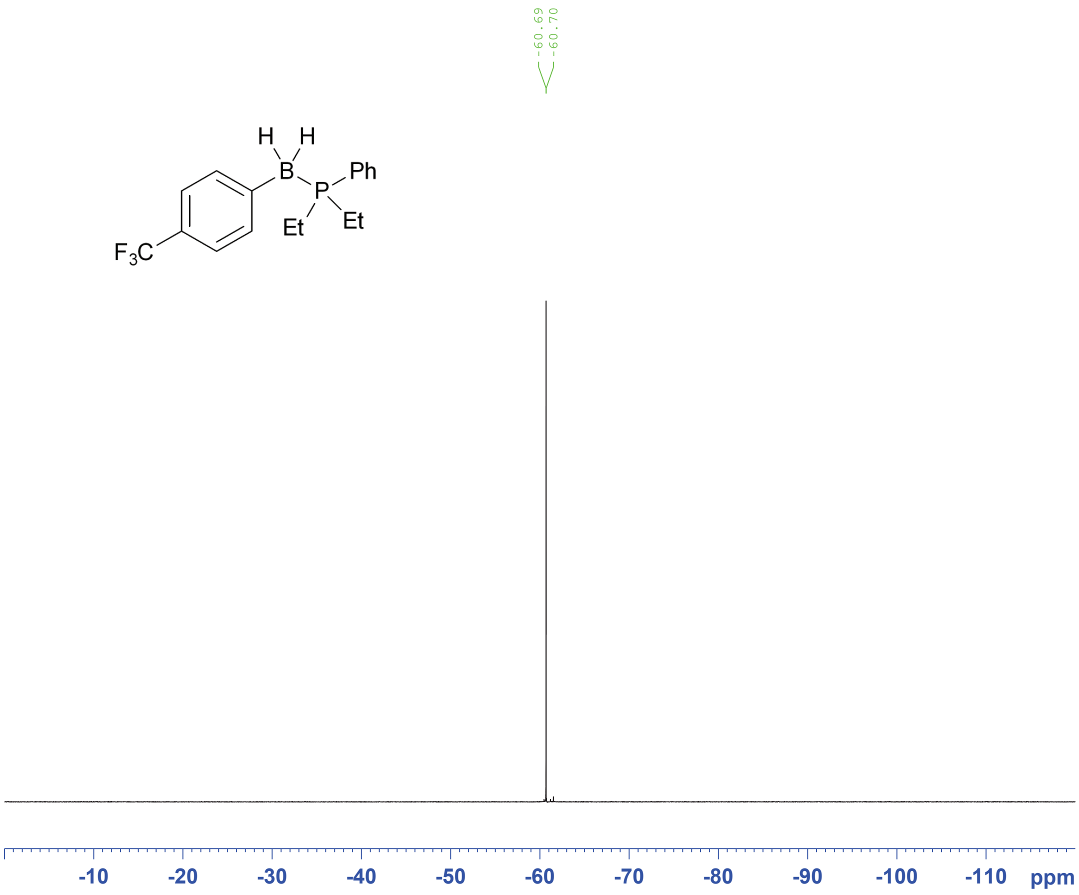

IBB-nmr Analysis

NAME	Feb11-2022
EXPNO	3
PROCNO	1
Date_	20220211
Time	18.32
INSTRUM	av400
PROBHD	5 mm PABBO BB-
PULPROG	zgfhigqn
TD	131072
SOLVENT	DMSO
NS	8
DS	4
SWH	89285.711 Hz
FIDRES	0.681196 Hz
AQ	0.7340532 sec
RG	203
DW	5.600 usec
DE	6.50 usec
TE	296.2 K
D1	1.00000000 sec
D11	0.03000000 sec
D12	0.00002000 sec
TD0	1
======== CHANNEL f1 ========	
NUC1	19F
P1	19.50 usec
PL1	-4.00 dB
PL1W	16.40489006 W
SFO1	376.4607164 MHz
======== CHANNEL f2 ========	
CPDPRG2	waltz16
NUC2	1H
PCPD2	112.00 usec
PL2	-4.00 dB
PL12	18.00 dB
PL2W	24.60733604 W
PL12W	0.15526180 W
SFO2	400.1316005 MHz
SI	65536
SF	376.4984617 MHz
WDW	EM
SSB	0
LB	0.30 Hz
GB	0
PC	1.00

4CF3PhBH2Et2Ph 11-21Fr

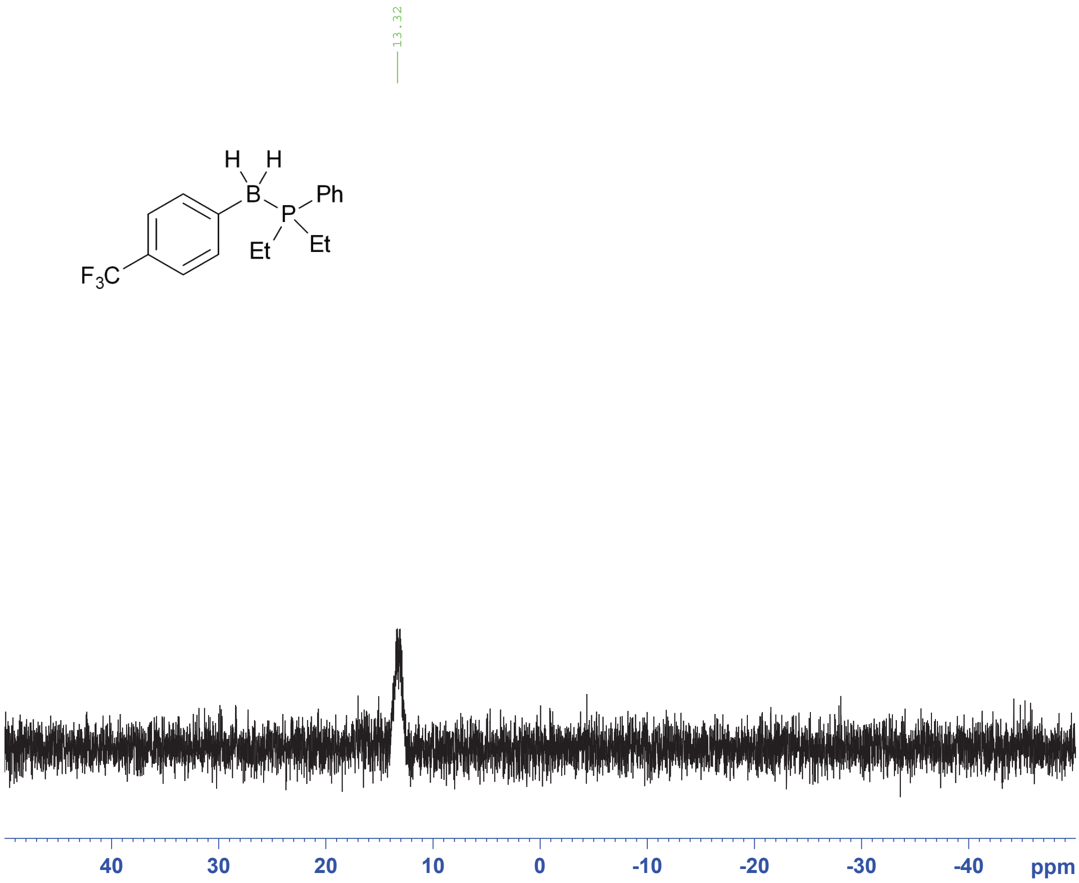

IBB-nmr Analysis

NAME	Feb11-2022
EXPNO	2
PROCNO	1
Date_	20220211
Time	18.28
INSTRUM	av400
PROBHD	5 mm PABBO BB-
PULPROG	zg30
TD	65536
SOLVENT	DMSO
NS	8
DS	4
SWH	64102.563 Hz
FIDRES	0.978127 Hz
AQ	0.5112308 sec
RG	203
DW	7.800 usec
DE	6.50 usec
TE	296.4 K
D1	2.00000000 sec
TD0	1
======== CHANNEL f1 ========	
NUC1	31P
P1	10.00 usec
PL1	-0.70 dB
PL1W	30.10233498 W
SFO1	161.9674942 MHz
SI	32768
SF	161.9754962 MHz
WDW	EM
SSB	0
LB	1.00 Hz
GB	0
PC	1.40

4CF3PhBH2P(i-Pr)3

B-4-(Trifluoromethyl)phenyl triisopropylphosphine–borane (32)

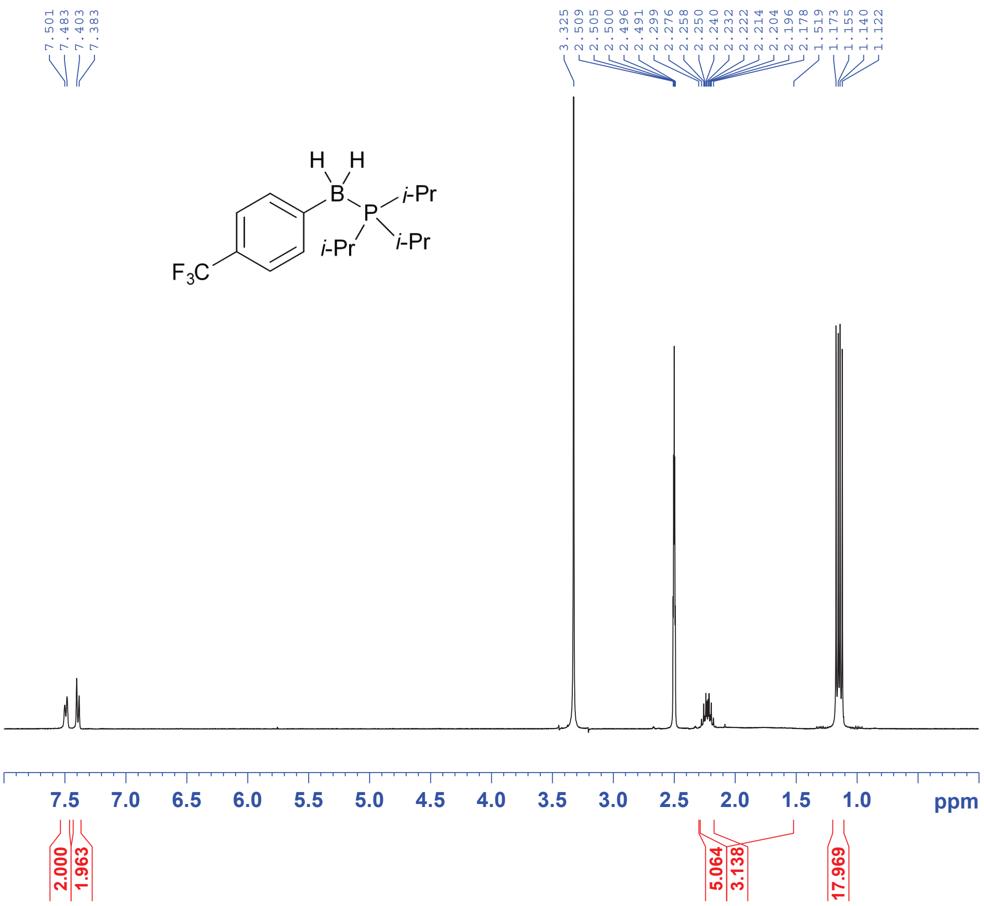

IBB-nmr Analysis

NAME	May28-2022
EXPNO	10
PROCNO	1
Date_	20220528
Time	22.52
INSTRUM	av400
PROBHD	5 mm PABBO BB-
PULPROG	zg30
TD	65536
SOLVENT	DMSO
NS	16
DS	2
SWH	8223.685 Hz
FIDRES	0.125483 Hz
AQ	3.9846387 sec
RG	203
DW	60.800 usec
DE	6.50 usec
TE	297.2 K
D1	1.00000000 sec
TD0	1

======== CHANNEL f1 ========

NUC1	1H
P1	14.00 usec
PL1	-1.80 dB
PL1W	14.82738590 W
SFO1	400.1324710 MHz
SI	32768
SF	400.1300034 MHz
WDW	EM
SSB	0
LB	0.30 Hz
GB	0
PC	1.00

4CF3PhBH2P(i-Pr)3

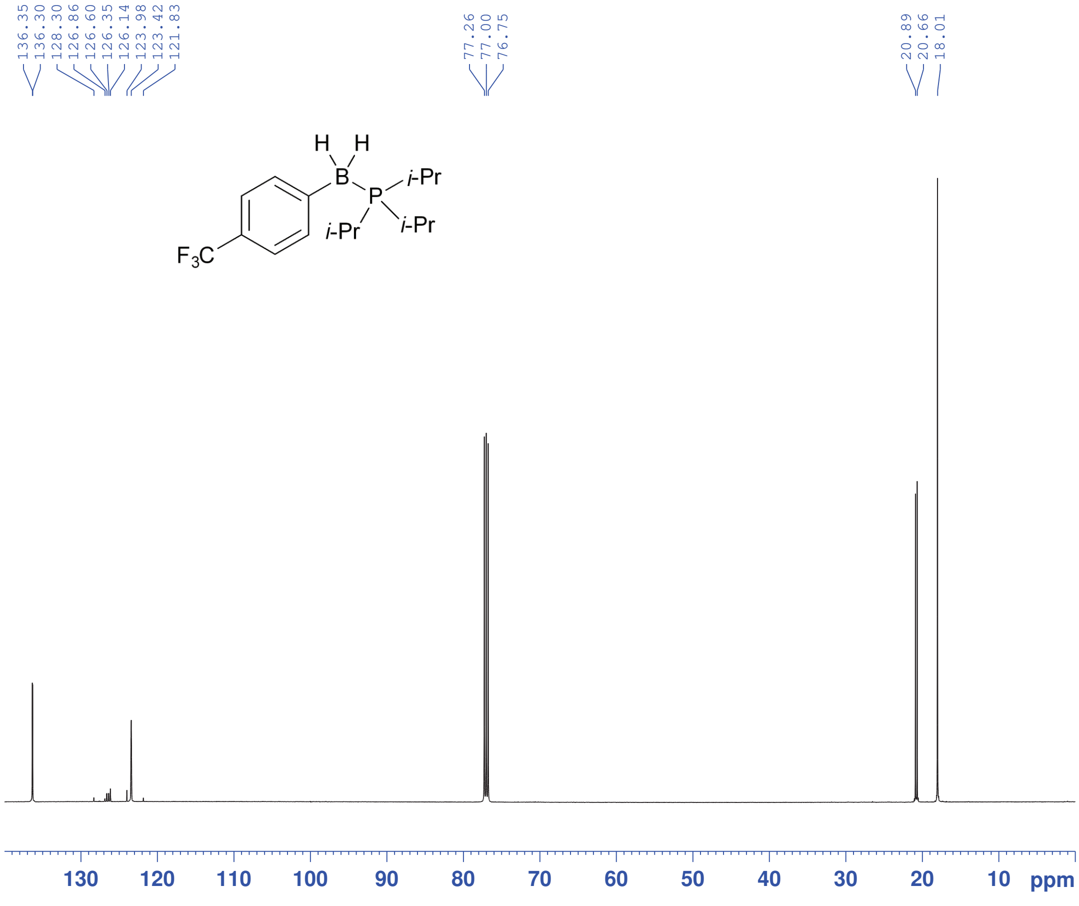

IBB-nmr Analysis

NAME	Mar08-2024
EXPNO	9
PROCNO	1
Date_	20240308
Time	5.14
INSTRUM	av500
PROBHD	5 mm CPDCH 13C
PULPROG	zgpg30
TD	65536
SOLVENT	CDCl3
NS	1024
DS	4
SWH	30030.029 Hz
FIDRES	0.458222 Hz
AQ	1.0912410 sec
RG	35.9
DW	16.650 usec
DE	20.00 usec
TE	298.0 K
D1	2.00000000 sec
D11	0.03000000 sec
TD0	1

======== CHANNEL f1 ========

NUC1	13C
P1	10.00 usec
PL1	-5.00 dB
PL1W	14.29790783 W
SFO1	125.7703643 MHz

======== CHANNEL f2 ========

CPDPRG2	waltz16
NUC2	1H
PCPD2	80.00 usec
PL2	-4.00 dB
PL12	14.06 dB
PL13	17.00 dB
PL2W	6.30957365 W
PL12W	0.09862794 W
PL13W	0.05011872 W
SFO2	500.1320005 MHz
SI	32768
SF	125.7577928 MHz
WDW	EM
SSB	0
LB	1.00 Hz
GB	0
PC	0.30

4CF3PhBH2P(i-Pr)3

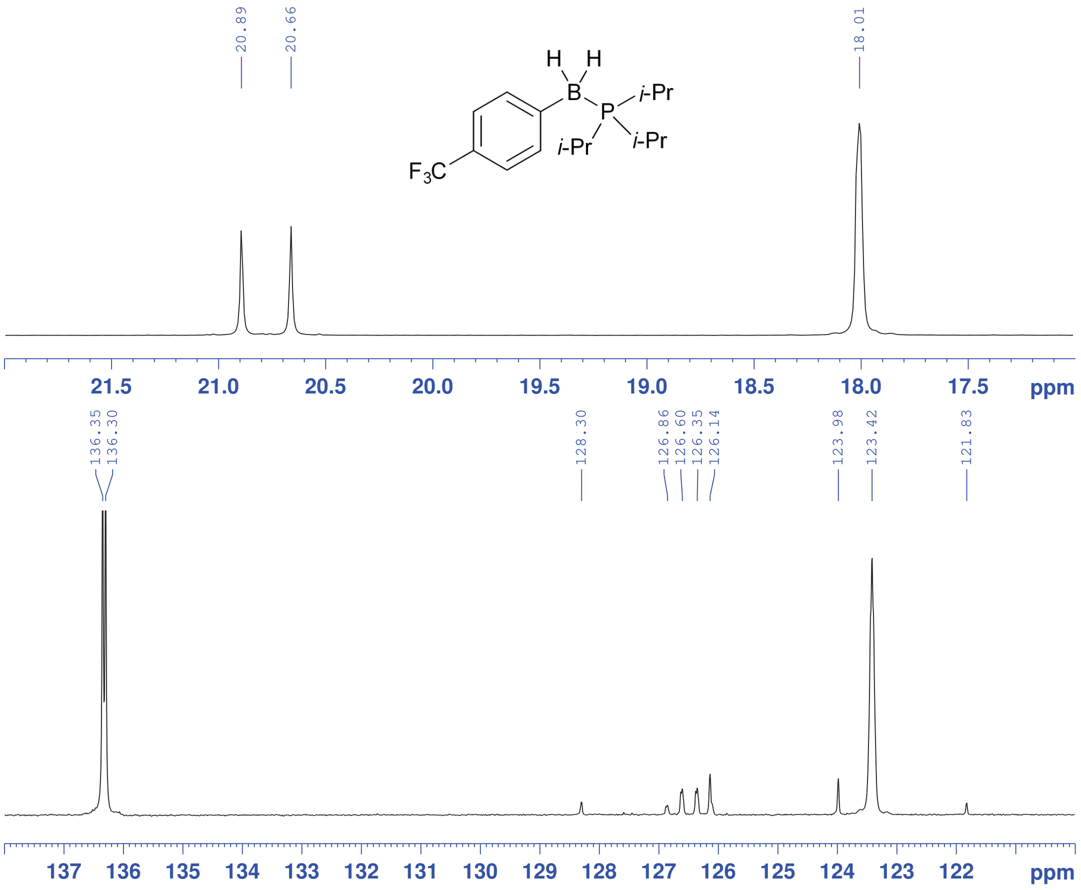

IBB-nmr Analysis

NAME	Mar08-2024
EXPNO	9
PROCNO	1
Date_	20240308
Time	5.14
INSTRUM	av500
PROBHD	5 mm CPDCH 13C
PULPROG	zgpg30
TD	65536
SOLVENT	CDCl3
NS	1024
DS	4
SWH	30030.029 Hz
FIDRES	0.458222 Hz
AQ	1.0912410 sec
RG	35.9
DW	16.650 usec
DE	20.00 usec
TE	298.0 K
D1	2.00000000 sec
D11	0.03000000 sec
TD0	1

======== CHANNEL f1 ========

NUC1	13C
P1	10.00 usec
PL1	-5.00 dB
PL1W	14.29790783 W
SFO1	125.7703643 MHz

======== CHANNEL f2 ========

CPDPRG2	waltz16
NUC2	1H
PCPD2	80.00 usec
PL2	-4.00 dB
PL12	14.06 dB
PL13	17.00 dB
PL2W	6.30957365 W
PL12W	0.09862794 W
PL13W	0.05011872 W
SFO2	500.1320005 MHz
SI	32768
SF	125.7577928 MHz
WDW	EM
SSB	0
LB	1.00 Hz
GB	0
PC	0.30

4CF3PhBH2P(i-Pr)3

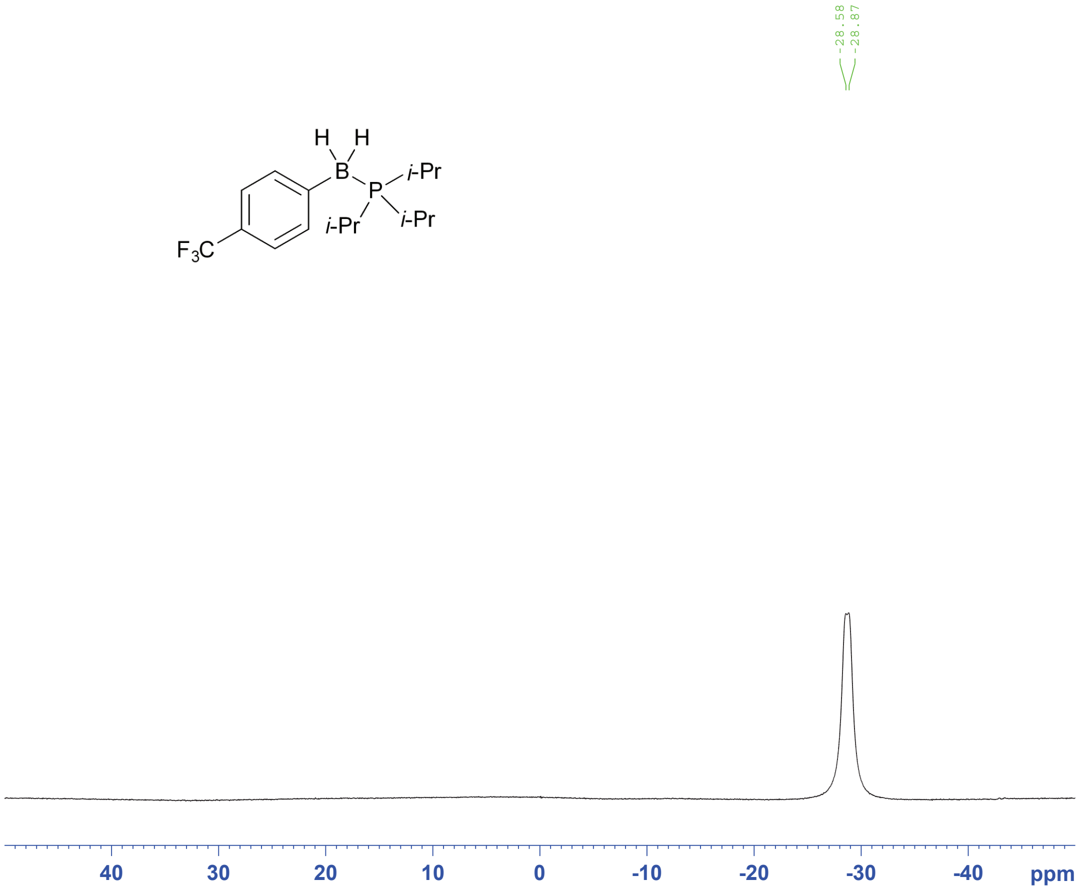

IBB-nmr Analysis

NAME	Apr10-2022
EXPNO	5
PROCNO	1
Date_	20220410
Time	19.49
INSTRUM	av400
PROBHD	5 mm PABBO BB-
PULPROG	zgig
TD	32768
SOLVENT	DMSO
NS	256
DS	4
SWH	20000.000 Hz
FIDRES	0.610352 Hz
AQ	0.8192500 sec
RG	203
DW	25.000 usec
DE	20.00 usec
TE	296.5 K
D1	1.00000000 sec
D11	0.03000000 sec
TD0	1

======== CHANNEL f1 ========

NUC1	11B
P1	6.30 usec
PL1	-2.00 dB
SFO1	128.3776050 MHz

======== CHANNEL f2 ========

CPDPRG2	waltz16
NUC2	1H
PCPD2	80.00 usec
PL2	-1.70 dB
PL12	13.80 dB
PL2W	14.48987389 W
PL12W	0.40838012 W
SFO2	400.1316005 MHz
SI	32768
SF	128.3776183 MHz
WDW	EM
SSB	0
LB	2.00 Hz
GB	0
PC	1.40

4CF3PhBH2P(i-Pr)3

IBB-nmr Analysis

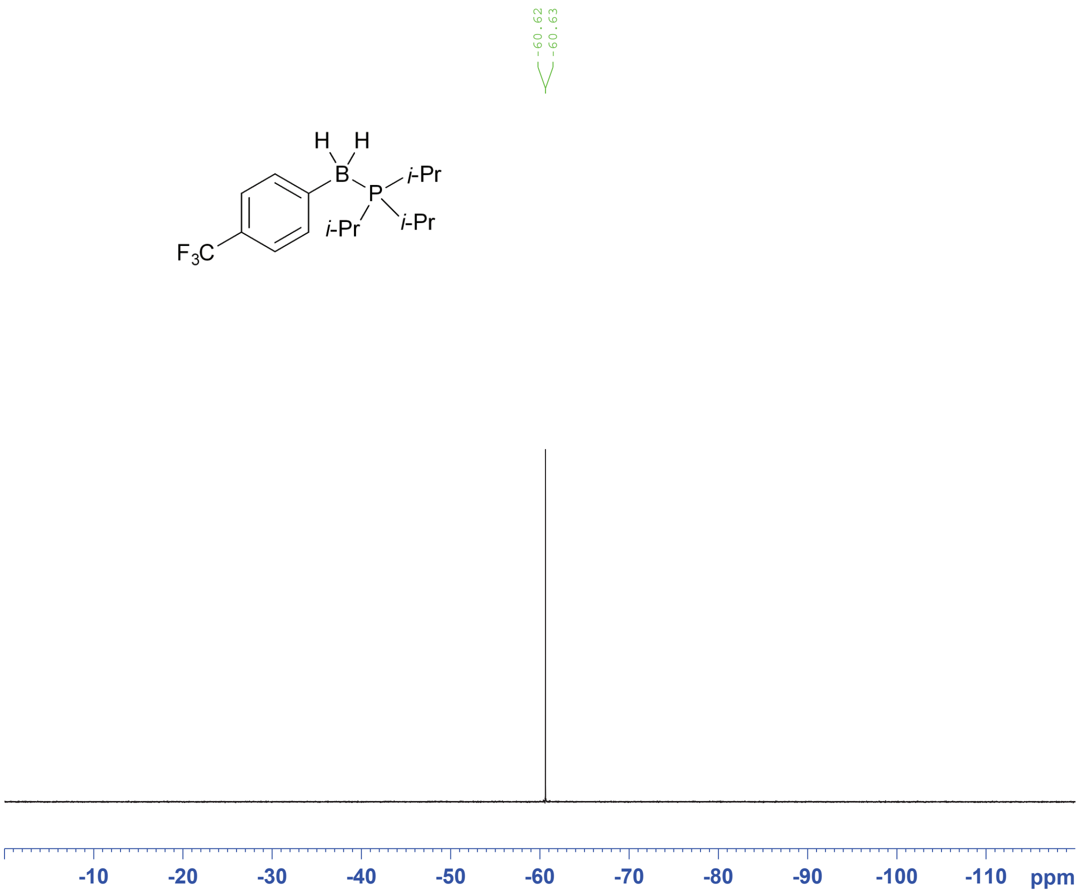


```
NAME           May27-2022
EXPNO                   2
PROCNO                  1
Date_            20220527
Time                23.39
INSTRUM             av400
PROBHD   5 mm PABBO BB-
PULPROG          zgfhigqn
TD                 131072
SOLVENT              DMSO
NS                      8
DS                      4
SWH             89285.711 Hz
FIDRES           0.681196 Hz
AQ              0.7340532 sec
RG                    203
DW                  5.600 usec
DE                   6.50 usec
TE                  297.2 K
D1             1.00000000 sec
D11            0.03000000 sec
D12            0.00002000 sec
TD0                     1

======== CHANNEL f1 ========
NUC1                  19F
P1                  19.50 usec
PL1                 -4.00 dB
PL1W          16.40489006 W
SFO1          376.4607164 MHz

======== CHANNEL f2 ========
CPDPRG2           waltz16
NUC2                   1H
PCPD2              112.00 usec
PL2                 -4.00 dB
PL12                18.00 dB
PL2W          24.60733604 W
PL12W          0.15526180 W
SFO2          400.1316005 MHz
SI                  65536
SF            376.4984617 MHz
WDW                    EM
SSB                     0
LB                   0.30 Hz
GB                      0
PC                   1.00
```

4CF3PhBH2P(i-Pr)3

IBB-nmr Analysis

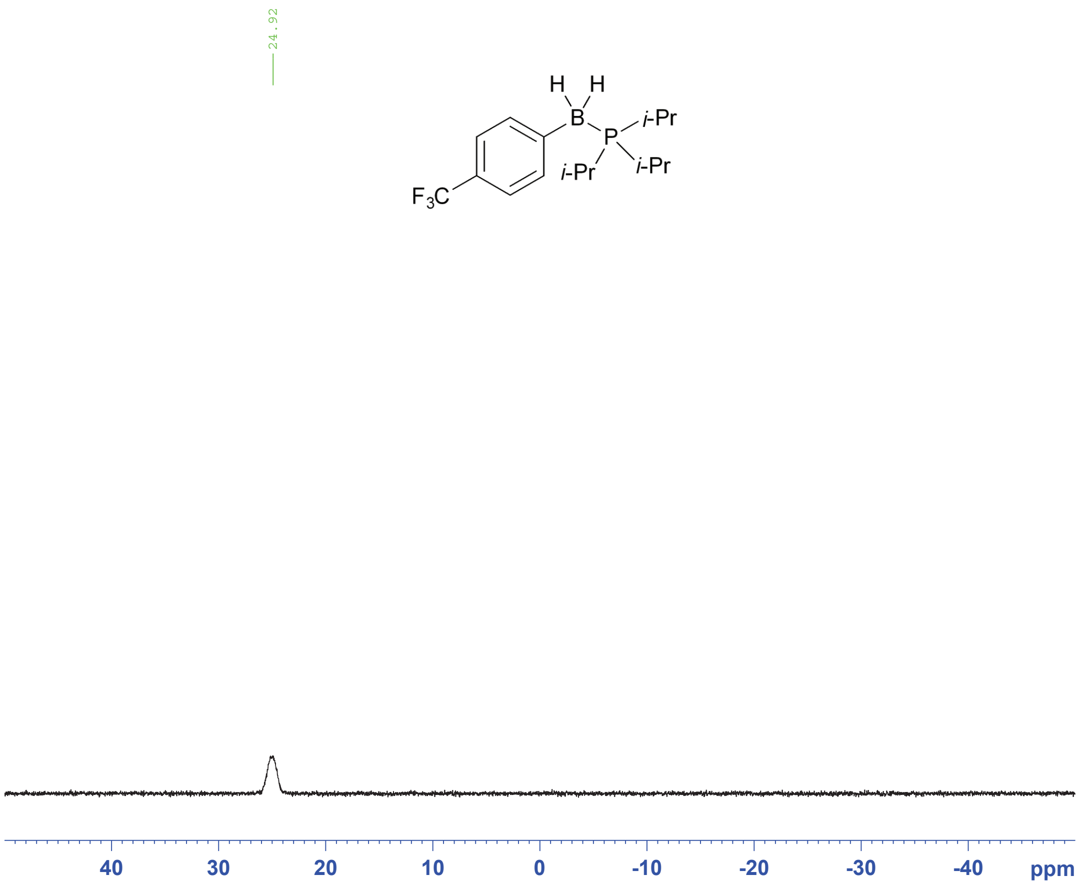


```
NAME           Apr10-2022
EXPNO                   7
PROCNO                  1
Date_            20220410
Time                19.55
INSTRUM             av400
PROBHD   5 mm PABBO BB-
PULPROG              zg30
TD                  65536
SOLVENT              DMSO
NS                     16
DS                      4
SWH             64102.563 Hz
FIDRES           0.978127 Hz
AQ              0.5112308 sec
RG                    203
DW                  7.800 usec
DE                   6.50 usec
TE                  296.0 K
D1             2.00000000 sec
TD0                     1

======== CHANNEL f1 ========
NUC1                  31P
P1                  10.00 usec
PL1                 -0.70 dB
PL1W          30.10233498 W
SFO1          161.9674942 MHz
SI                  32768
SF            161.9754962 MHz
WDW                    EM
SSB                     0
LB                   1.00 Hz
GB                      0
PC                   1.40
```

4CF3PhBH2PPh(i-Pr)2 B-4-(Trifluoromethyl)phenyl diisopropyl(phenyl)phosphine–borane (33)

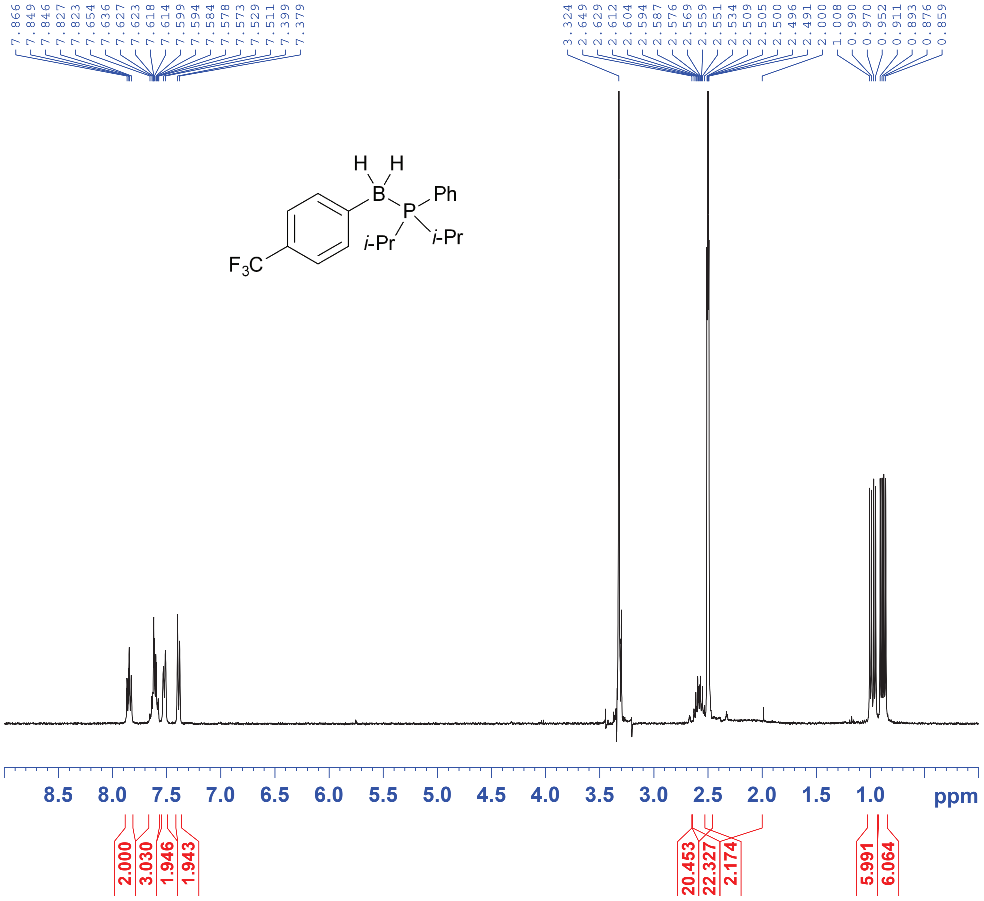

IBB-nmr Analysis

```
NAME           May28-2022
EXPNO                   1
PROCNO                  1
Date_            20220528
Time                19.10
INSTRUM             av400
PROBHD    5 mm PABBO BB-
PULPROG              zg30
TD                  65536
SOLVENT              DMSO
NS                     16
DS                      2
SWH              8223.685 Hz
FIDRES           0.125483 Hz
AQ              3.9846387 sec
RG                    203
DW                 60.800 usec
DE                   6.50 usec
TE                  297.1 K
D1             1.00000000 sec
TD0                     1

======== CHANNEL f1 ========
NUC1                   1H
P1                  14.00 usec
PL1                 -1.80 dB
PL1W          14.82738590 W
SFO1          400.1324710 MHz
SI                  32768
SF            400.1300029 MHz
WDW                    EM
SSB                     0
LB                   0.30 Hz
GB                      0
PC                   1.00
```

4CF3PhBH2PPh(i-Pr)2

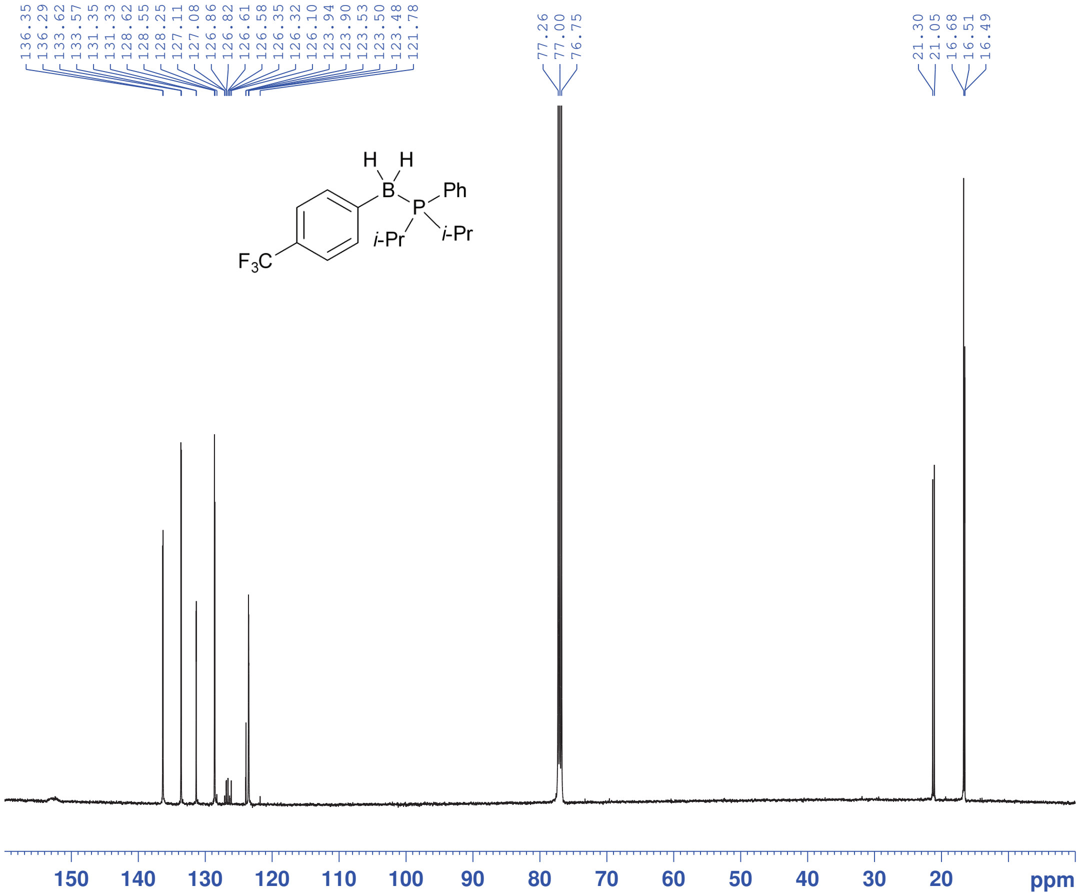

IBB-nmr Analysis

```
NAME           Mar06-2024
EXPNO                  13
PROCNO                  1
Date_            20240306
Time                20.54
INSTRUM             av500
PROBHD   5 mm CPDCH 13C
PULPROG           zgpg30
TD                  65536
SOLVENT             CDCl3
NS                   1024
DS                      4
SWH             30030.029 Hz
FIDRES           0.458222 Hz
AQ              1.0912410 sec
RG                   35.9
DW                 16.650 usec
DE                  20.00 usec
TE                  297.9 K
D1             2.00000000 sec
D11            0.03000000 sec
TD0                     1

======== CHANNEL f1 ========
NUC1                  13C
P1                  10.00 usec
PL1                 -5.00 dB
PL1W          14.29790783 W
SFO1          125.7703643 MHz

======== CHANNEL f2 ========
CPDPRG2           waltz16
NUC2                   1H
PCPD2               80.00 usec
PL2                 -4.00 dB
PL12                14.06 dB
PL13                17.00 dB
PL2W           6.30957365 W
PL12W          0.09862794 W
PL13W          0.05011872 W
SFO2          500.1320005 MHz
SI                  32768
SF            125.7577918 MHz
WDW                    EM
SSB                     0
LB                   1.00 Hz
GB                      0
PC                   0.30
```

4CF3PhBH2PPh(i-Pr)2

IBB-nmr Analysis

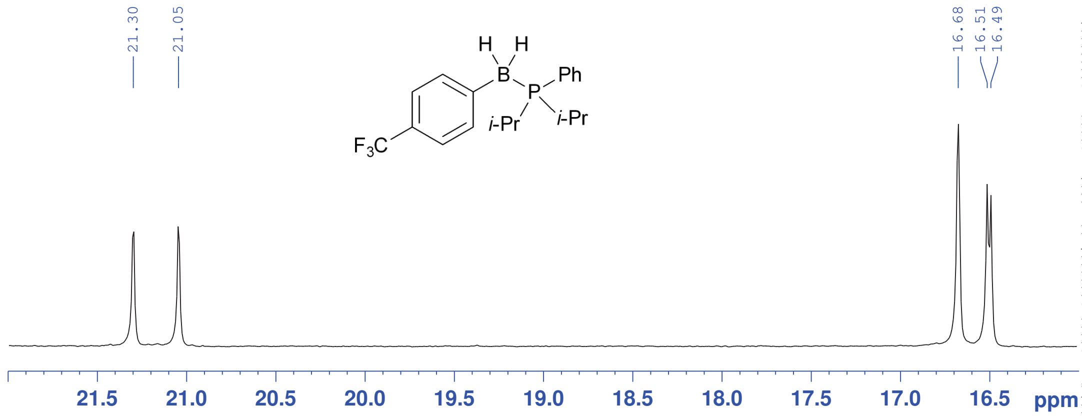

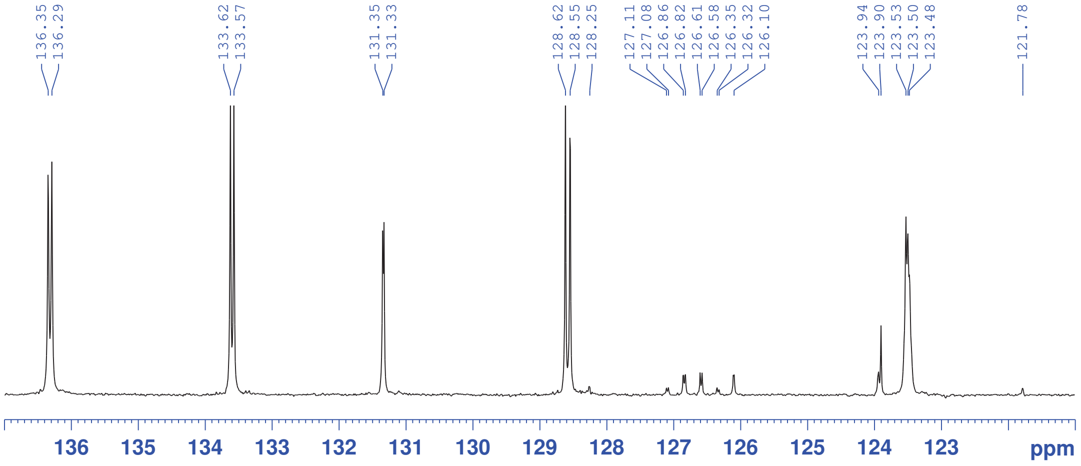


```
NAME           Mar06-2024
EXPNO                  13
PROCNO                  1
Date_            20240306
Time                20.54
INSTRUM             av500
PROBHD    5 mm CPDCH 13C
PULPROG           zgpg30
TD                  65536
SOLVENT             CDCl3
NS                   1024
DS                      4
SWH             30030.029 Hz
FIDRES           0.458222 Hz
AQ              1.0912410 sec
RG                   35.9
DW                 16.650 usec
DE                  20.00 usec
TE                  297.9 K
D1             2.00000000 sec
D11            0.03000000 sec
TD0                     1

======== CHANNEL f1 ========
NUC1                  13C
P1                  10.00 usec
PL1                 -5.00 dB
PL1W          14.29790783 W
SFO1         125.7703643 MHz

======== CHANNEL f2 ========
CPDPRG2           waltz16
NUC2                   1H
PCPD2               80.00 usec
PL2                 -4.00 dB
PL12                14.06 dB
PL13                17.00 dB
PL2W           6.30957365 W
PL12W          0.09862794 W
PL13W          0.05011872 W
SFO2         500.1320005 MHz
SI                  32768
SF           125.7577918 MHz
WDW                    EM
SSB                     0
LB                   1.00 Hz
GB                      0
PC                   0.30
```

4CF3PhBH2PPh(i-Pr)2

IBB-nmr Analysis

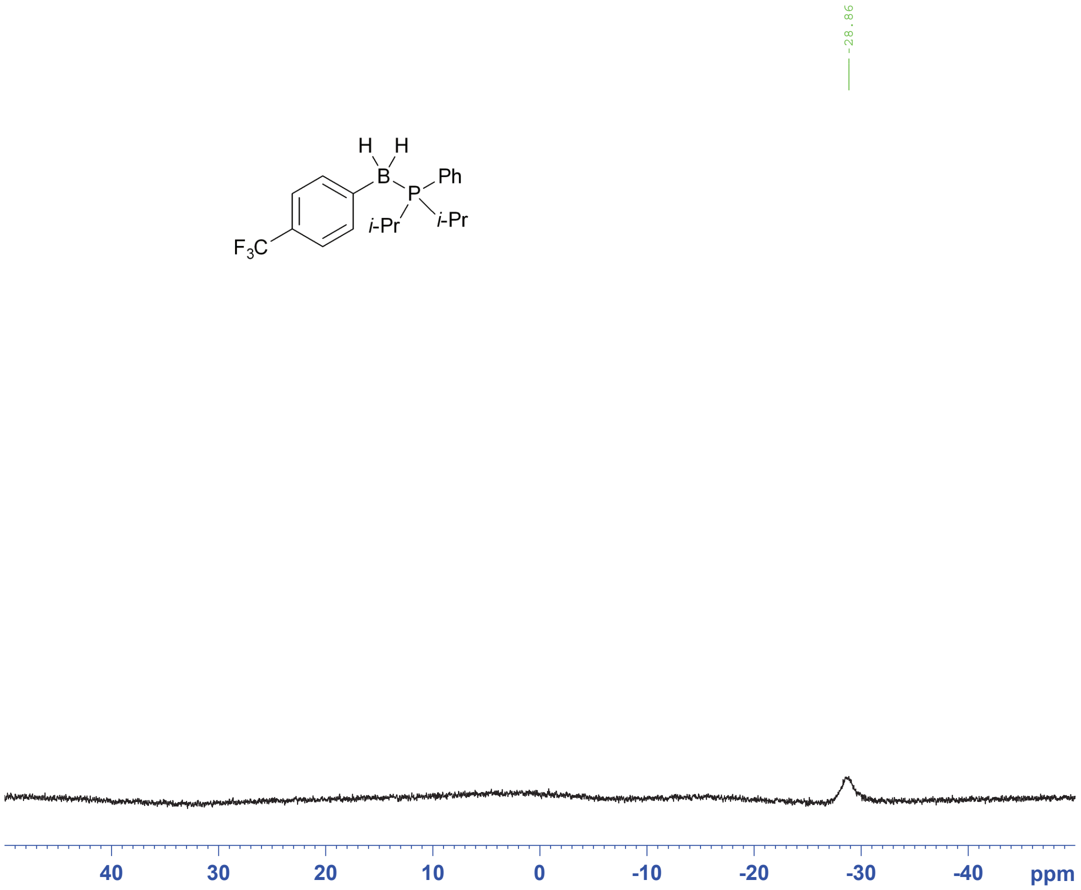


```
NAME           May28-2022
EXPNO                   5
PROCNO                  1
Date_            20220528
Time                19.30
INSTRUM             av400
PROBHD    5 mm PABBO BB-
PULPROG              zgig
TD                  32768
SOLVENT              DMSO
NS                    128
DS                      4
SWH             20000.000 Hz
FIDRES           0.610352 Hz
AQ              0.8192500 sec
RG                    203
DW                 25.000 usec
DE                  20.00 usec
TE                  297.8 K
D1             1.00000000 sec
D11            0.03000000 sec
TD0                     1

======== CHANNEL f1 ========
NUC1                  11B
P1                   6.30 usec
PL1                 -2.00 dB
SFO1         128.3776050 MHz

======== CHANNEL f2 ========
CPDPRG2           waltz16
NUC2                   1H
PCPD2               80.00 usec
PL2                 -1.70 dB
PL12                13.80 dB
PL2W          14.48987389 W
PL12W          0.40838012 W
SFO2         400.1316005 MHz
SI                  32768
SF           128.3776183 MHz
WDW                    EM
SSB                     0
LB                   2.00 Hz
GB                      0
PC                   1.40
```

4CF3PhBH2PPh(i-Pr)2

IBB-nmr Analysis

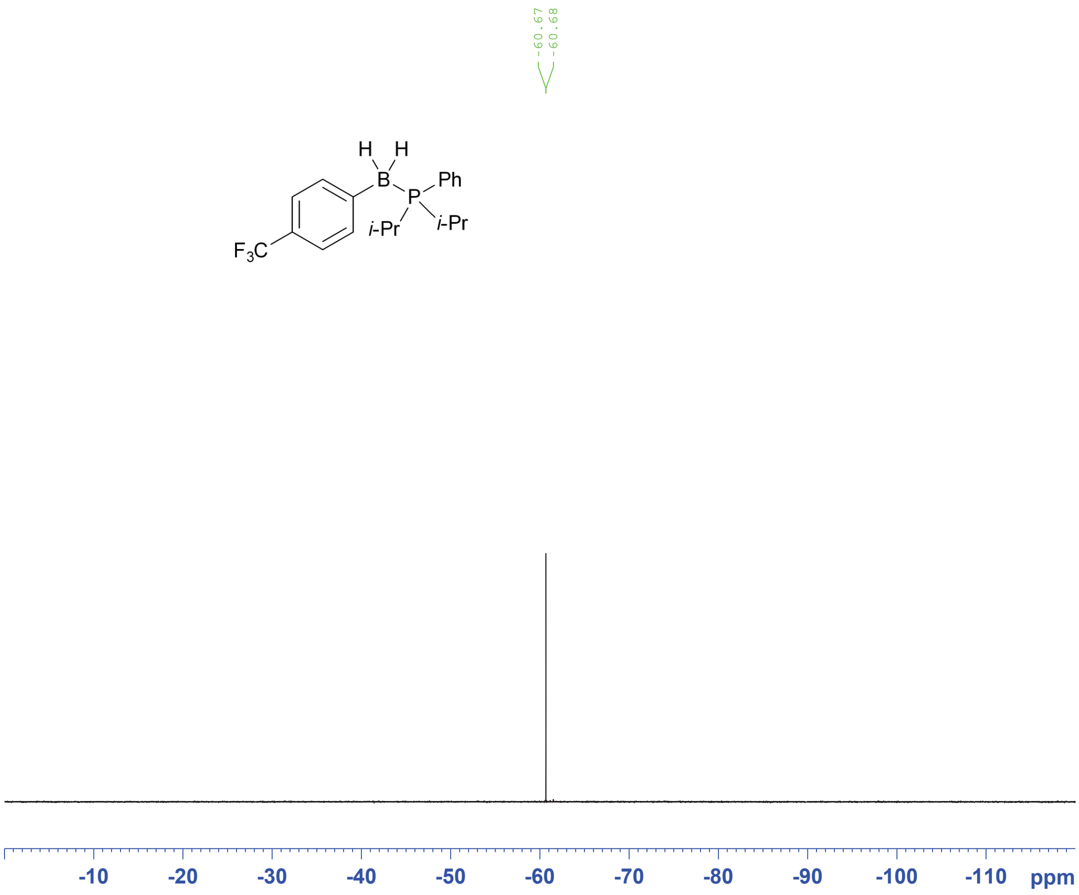

NAME	May28-2022
EXPNO	4
PROCNO	1
Date_	20220528
Time	19.23
INSTRUM	av400
PROBHD	5 mm PABBO BB-
PULPROG	zgfhigqn
TD	131072
SOLVENT	DMSO
NS	8
DS	4
SWH	89285.711 Hz
FIDRES	0.681196 Hz
AQ	0.7340532 sec
RG	203
DW	5.600 usec
DE	6.50 usec
TE	297.2 K
D1	1.00000000 sec
D11	0.03000000 sec
D12	0.00002000 sec
TD0	1
======== CHANNEL f1 ========	
NUC1	19F
P1	19.50 usec
PL1	-4.00 dB
PL1W	16.40489006 W
SFO1	376.4607164 MHz
======== CHANNEL f2 ========	
CPDPRG2	waltz16
NUC2	1H
PCPD2	112.00 usec
PL2	-4.00 dB
PL12	18.00 dB
PL2W	24.60733604 W
PL12W	0.15526180 W
SFO2	400.1316005 MHz
SI	65536
SF	376.4984617 MHz
WDW	EM
SSB	0
LB	0.30 Hz
GB	0
PC	1.00

4CF3PhBH2PPh(i-Pr)2

IBB-nmr Analysis

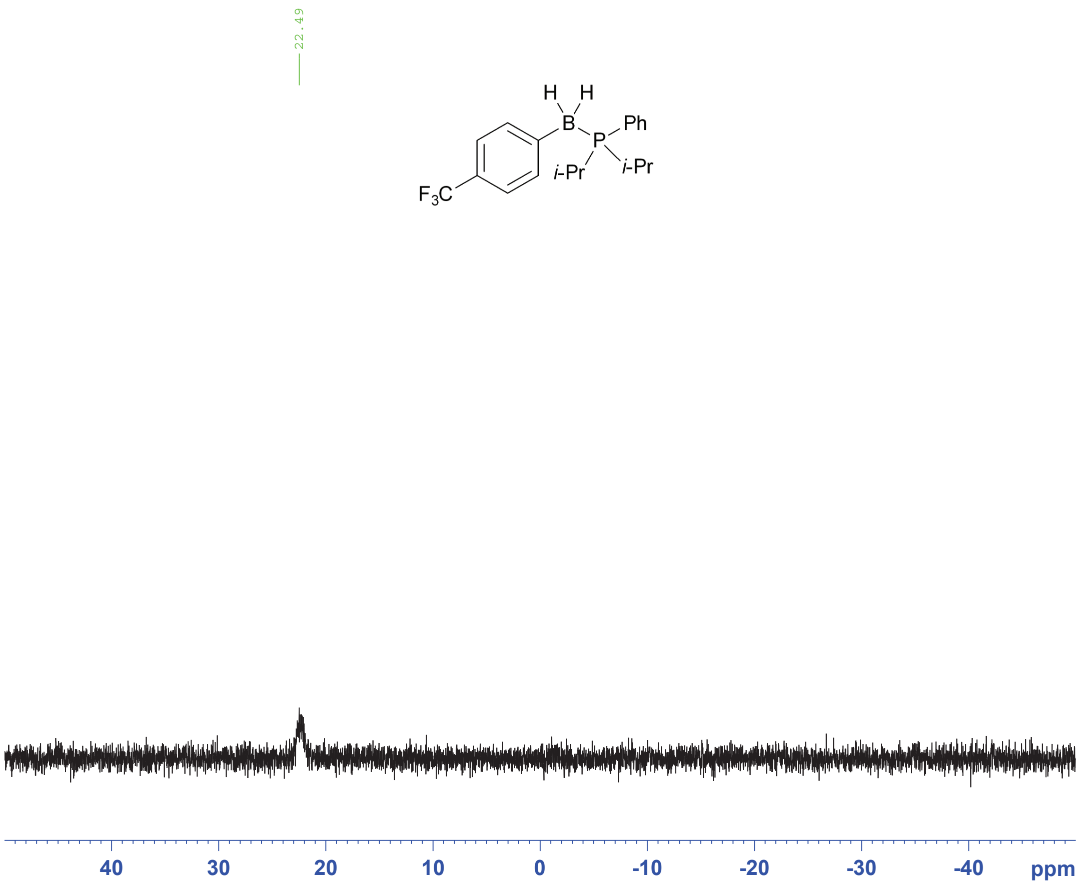

NAME	May28-2022
EXPNO	3
PROCNO	1
Date_	20220528
Time	19.20
INSTRUM	av400
PROBHD	5 mm PABBO BB-
PULPROG	zg30
TD	65536
SOLVENT	DMSO
NS	128
DS	4
SWH	64102.563 Hz
FIDRES	0.978127 Hz
AQ	0.5112308 sec
RG	203
DW	7.800 usec
DE	6.50 usec
TE	297.1 K
D1	2.00000000 sec
TD0	1
======== CHANNEL f1 ========	
NUC1	31P
P1	10.00 usec
PL1	-0.70 dB
PL1W	30.10233498 W
SFO1	161.9674942 MHz
SI	32768
SF	161.9754962 MHz
WDW	EM
SSB	0
LB	1.00 Hz
GB	0
PC	1.40

4CF3PhBH2P(cyclopropyl)3 8-15Fr B-4-(Trifluoromethyl)phenyl tricyclopropylphosphine–borane (34)

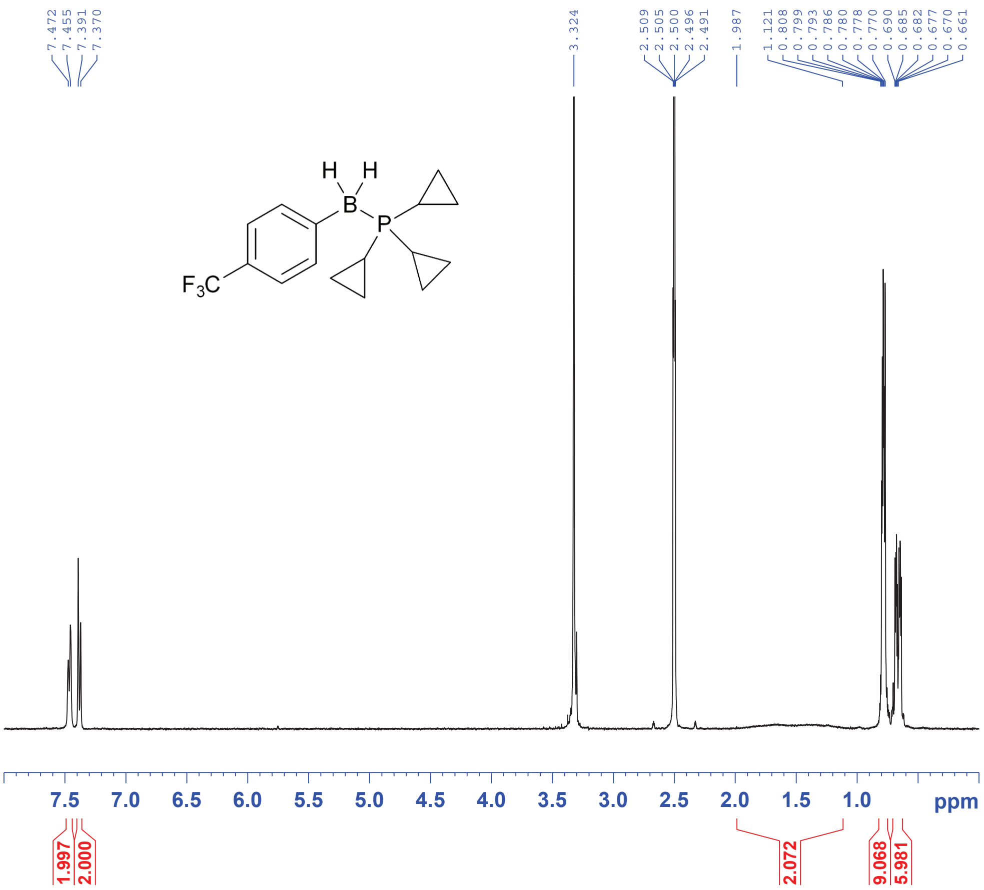

IBB-nmr Analysis

NAME	July14-2022
EXPNO	1
PROCNO	1
Date_	20220713
Time	23.54
INSTRUM	av400
PROBHD	5 mm PABBO BB-
PULPROG	zg30
TD	65536
SOLVENT	DMSO
NS	16
DS	2
SWH	8223.685 Hz
FIDRES	0.125483 Hz
AQ	3.9846387 sec
RG	203
DW	60.800 usec
DE	6.50 usec
TE	297.1 K
D1	1.00000000 sec
TD0	1
======== CHANNEL f1 ========	
NUC1	1H
P1	14.00 usec
PL1	-1.80 dB
PL1W	14.82738590 W
SFO1	400.1324710 MHz
SI	32768
SF	400.1300029 MHz
WDW	EM
SSB	0
LB	0.30 Hz
GB	0
PC	1.00

4CF3PhBH2P(cyclopropyl)3

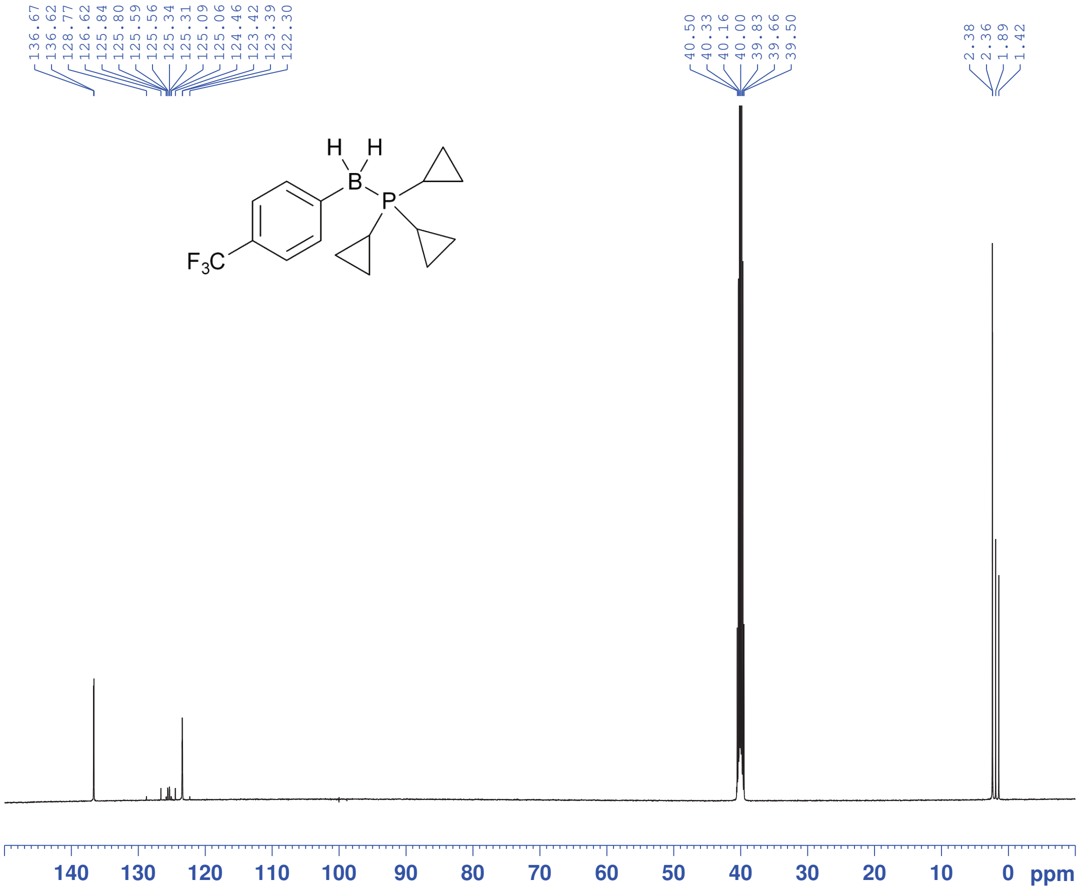

IBB-nmr Analysis

NAME	Mar06-2024
EXPNO	5
PROCNO	1
Date_	20240306
Time	1.42
INSTRUM	av500
PROBHD	5 mm CPDCH 13C
PULPROG	zgpg30
TD	65536
SOLVENT	DMSO
NS	256
DS	4
SWH	30030.029 Hz
FIDRES	0.458222 Hz
AQ	1.0912410 sec
RG	35.9
DW	16.650 usec
DE	20.00 usec
TE	298.0 K
D1	2.00000000 sec
D11	0.03000000 sec
TD0	1
======== CHANNEL f1 ========	
NUC1	13C
P1	10.00 usec
PL1	-5.00 dB
PL1W	14.29790783 W
SFO1	125.7703643 MHz
======== CHANNEL f2 ========	
CPDPRG2	waltz16
NUC2	1H
PCPD2	80.00 usec
PL2	-4.00 dB
PL12	14.06 dB
PL13	17.00 dB
PL2W	6.30957365 W
PL12W	0.09862794 W
PL13W	0.05011872 W
SFO2	500.1320005 MHz
SI	32768
SF	125.7577875 MHz
WDW	EM
SSB	0
LB	1.00 Hz
GB	0
PC	0.30

4CF3PhBH2P(cyclopropyl)3

IBB-nmr Analysis

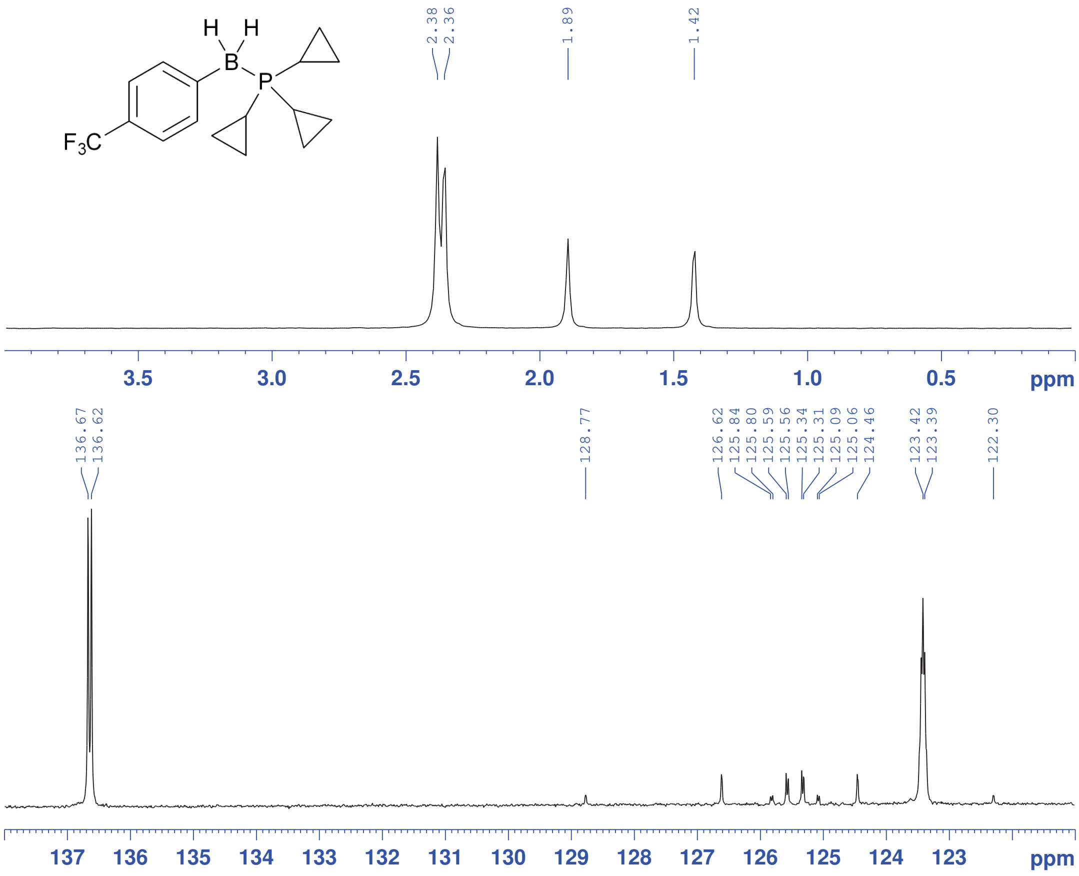

NAME	Mar06-2024
EXPNO	5
PROCNO	1
Date_	20240306
Time	1.42
INSTRUM	av500
PROBHD	5 mm CPDCH 13C
PULPROG	zgpg30
TD	65536
SOLVENT	DMSO
NS	256
DS	4
SWH	30030.029 Hz
FIDRES	0.458222 Hz
AQ	1.0912410 sec
RG	35.9
DW	16.650 usec
DE	20.00 usec
TE	298.0 K
D1	2.00000000 sec
D11	0.03000000 sec
TD0	1
======== CHANNEL f1 ========	
NUC1	13C
P1	10.00 usec
PL1	-5.00 dB
PL1W	14.29790783 W
SFO1	125.7703643 MHz
======== CHANNEL f2 ========	
CPDPRG2	waltz16
NUC2	1H
PCPD2	80.00 usec
PL2	-4.00 dB
PL12	14.06 dB
PL13	17.00 dB
PL2W	6.30957365 W
PL12W	0.09862794 W
PL13W	0.05011872 W
SFO2	500.1320005 MHz
SI	32768
SF	125.7577875 MHz
WDW	EM
SSB	0
LB	1.00 Hz
GB	0
PC	0.30

4CF3PhBH2P(cyclopropyl)3 8-15Fr

IBB-nmr Analysis

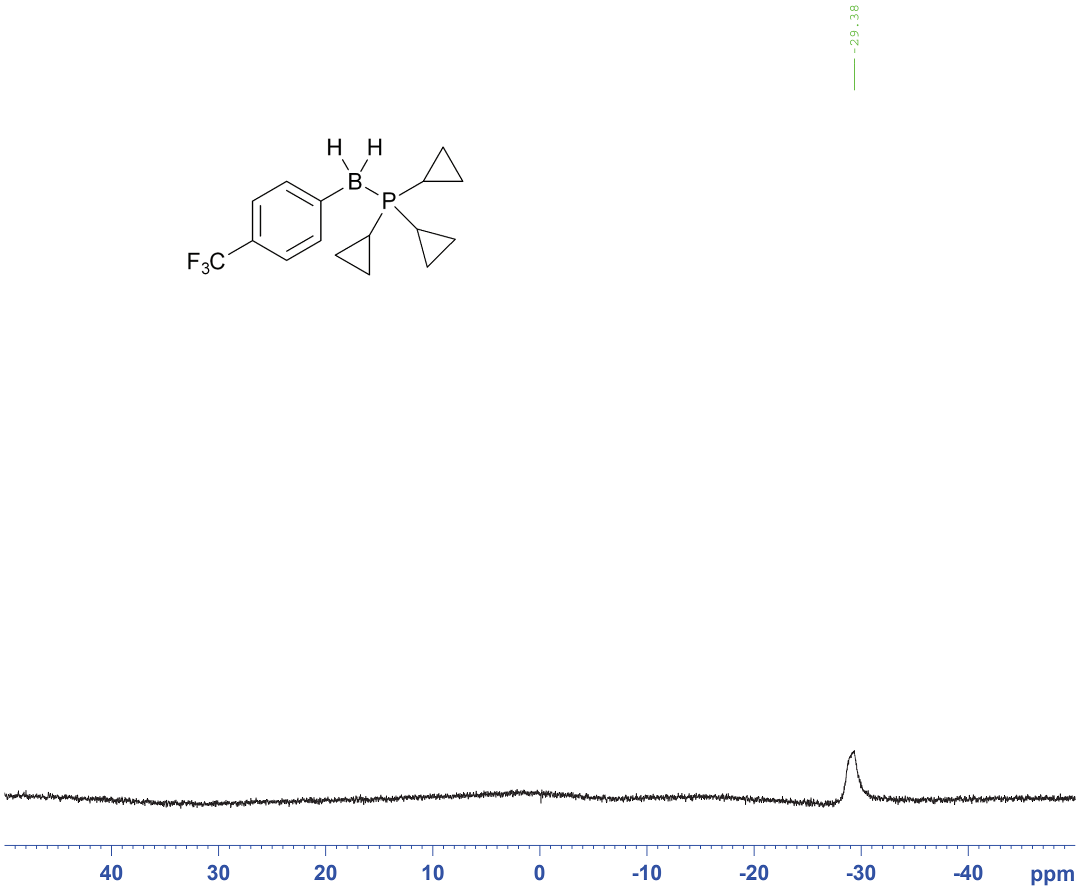

NAME	July14-2022
EXPNO	4
PROCNO	1
Date_	20220714
Time	0.10
INSTRUM	av400
PROBHD	5 mm PABBO BB-
PULPROG	zgig
TD	32768
SOLVENT	DMSO
NS	128
DS	4
SWH	20000.000 Hz
FIDRES	0.610352 Hz
AQ	0.8192500 sec
RG	203
DW	25.000 usec
DE	20.00 usec
TE	297.8 K
D1	1.00000000 sec
D11	0.03000000 sec
TD0	1
======== CHANNEL f1 ========	
NUC1	11B
P1	6.30 usec
PL1	-2.00 dB
SFO1	128.3776050 MHz
======== CHANNEL f2 ========	
CPDPRG2	waltz16
NUC2	1H
PCPD2	80.00 usec
PL2	-1.70 dB
PL12	13.80 dB
PL2W	14.48987389 W
PL12W	0.40838012 W
SFO2	400.1316005 MHz
SI	32768
SF	128.3776183 MHz
WDW	EM
SSB	0
LB	2.00 Hz
GB	0
PC	1.40

4CF3PhBH2P(cyclopropyl)3 8-15Fr

IBB-nmr Analysis

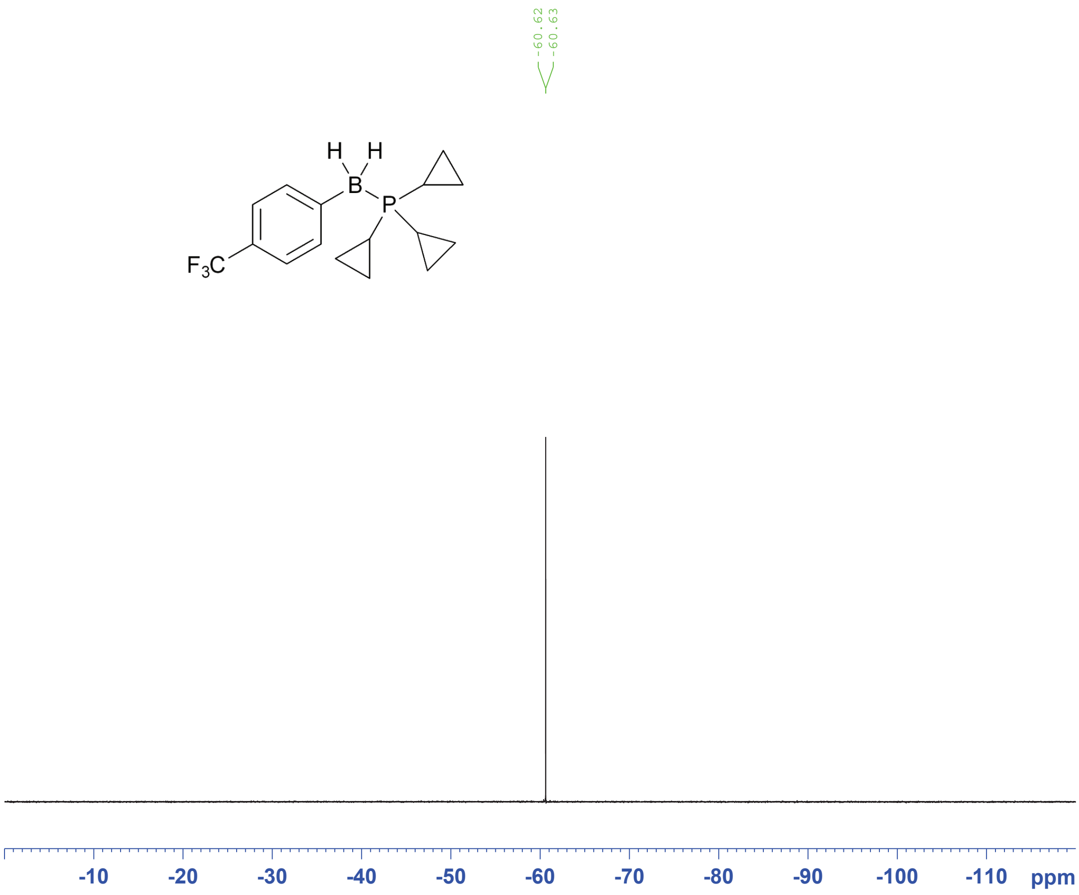

NAME	July14-2022
EXPNO	2
PROCNO	1
Date_	20220713
Time	23.57
INSTRUM	av400
PROBHD	5 mm PABBO BB-
PULPROG	zgfhigqn
TD	131072
SOLVENT	DMSO
NS	8
DS	4
SWH	89285.711 Hz
FIDRES	0.681196 Hz
AQ	0.7340532 sec
RG	203
DW	5.600 usec
DE	6.50 usec
TE	297.2 K
D1	1.00000000 sec
D11	0.03000000 sec
D12	0.00002000 sec
TD0	1

======== CHANNEL f1 ========

NUC1	19F
P1	19.50 usec
PL1	-4.00 dB
PL1W	16.40489006 W
SFO1	376.4607164 MHz

======== CHANNEL f2 ========

CPDPRG2	waltz16
NUC2	1H
PCPD2	112.00 usec
PL2	-4.00 dB
PL12	18.00 dB
PL2W	24.60733604 W
PL12W	0.15526180 W
SFO2	400.1316005 MHz
SI	65536
SF	376.4984617 MHz
WDW	EM
SSB	0
LB	0.30 Hz
GB	0
PC	1.00

4CF3PhBH2P(cyclopropyl)3 8-15Fr

IBB-nmr Analysis

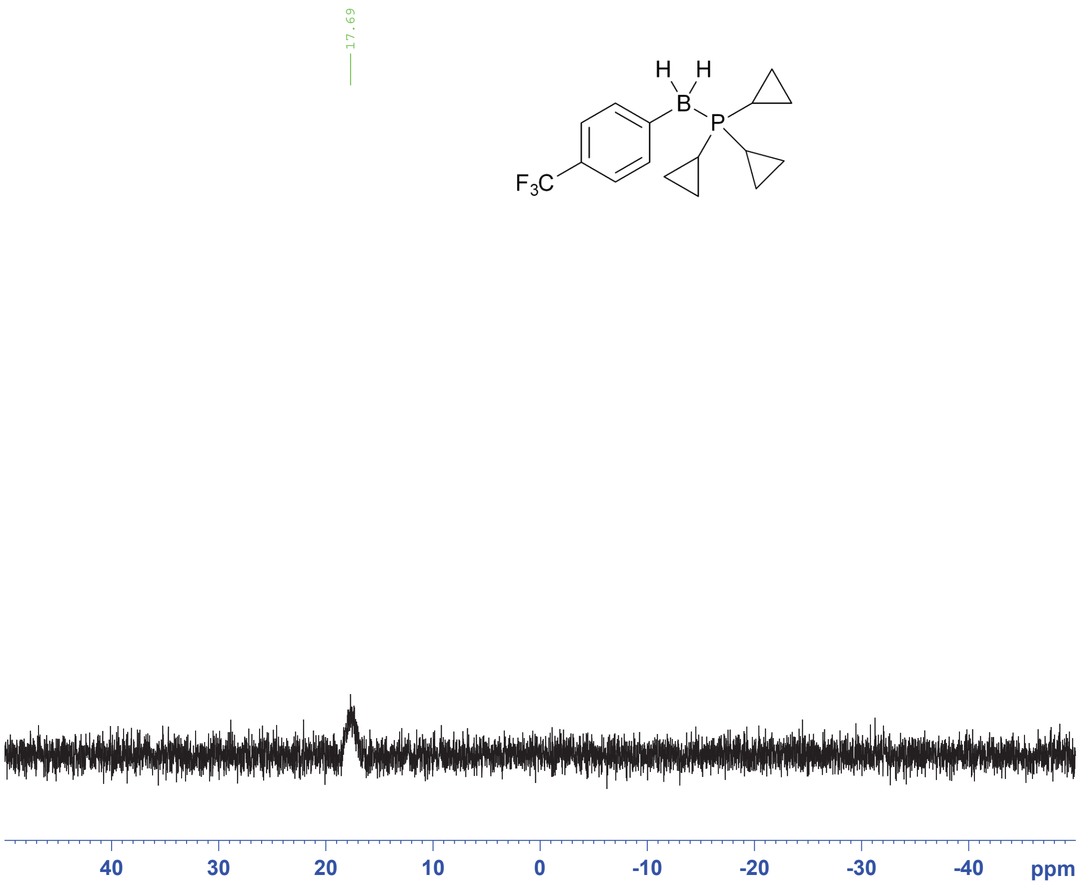

NAME	July14-2022
EXPNO	3
PROCNO	1
Date_	20220714
Time	0.03
INSTRUM	av400
PROBHD	5 mm PABBO BB-
PULPROG	zg30
TD	65536
SOLVENT	DMSO
NS	64
DS	4
SWH	64102.563 Hz
FIDRES	0.978127 Hz
AQ	0.5112308 sec
RG	203
DW	7.800 usec
DE	6.50 usec
TE	297.1 K
D1	2.00000000 sec
TD0	1

======== CHANNEL f1 ========

NUC1	31P
P1	10.00 usec
PL1	-0.70 dB
PL1W	30.10233498 W
SFO1	161.9674942 MHz
SI	32768
SF	161.9754962 MHz
WDW	EM
SSB	0
LB	1.00 Hz
GB	0
PC	1.40

4CF3PhBH2P(n-Bu)3 recolumn

B-4-(Trifluoromethyl)phenyl tri-n-butylphosphine–borane (35)

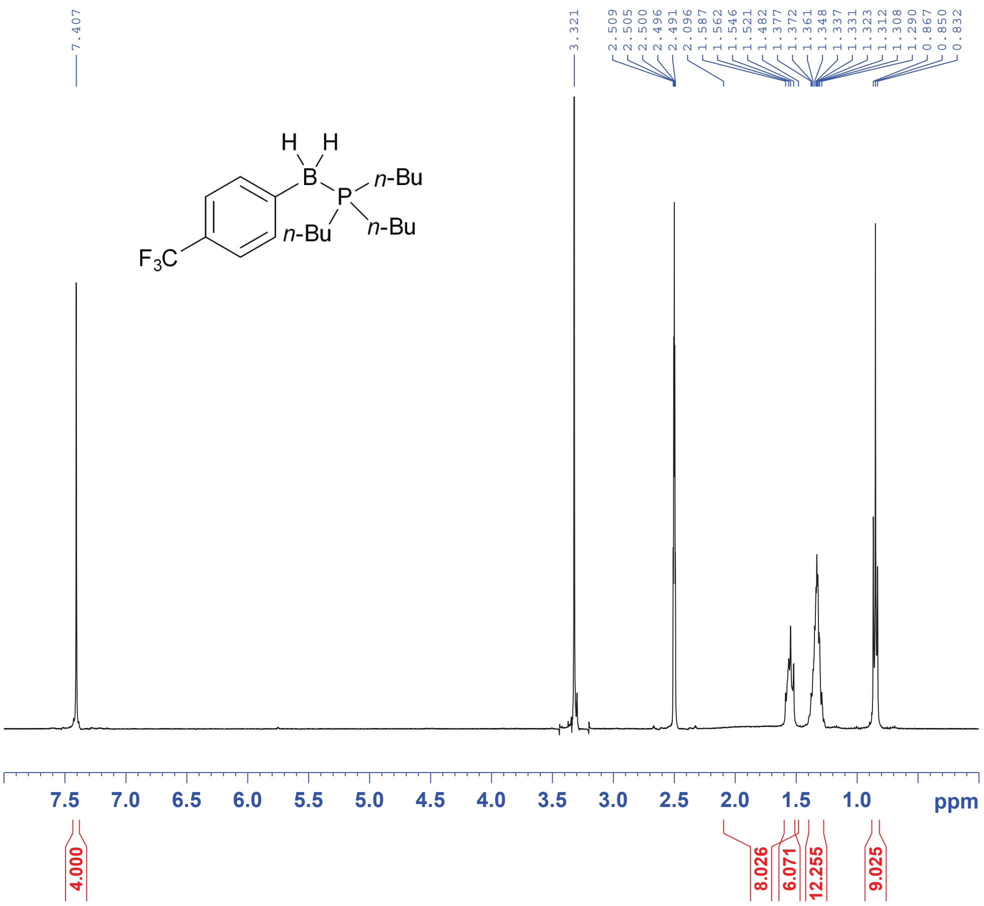

IBB-nmr Analysis

NAME	July26-2022
EXPNO	1
PROCNO	1
Date_	20220726
Time	21.28
INSTRUM	av400
PROBHD	5 mm PABBO BB-
PULPROG	zg30
TD	65536
SOLVENT	DMSO
NS	16
DS	2
SWH	8223.685 Hz
FIDRES	0.125483 Hz
AQ	3.9846387 sec
RG	203
DW	60.800 usec
DE	6.50 usec
TE	297.8 K
D1	1.00000000 sec
TD0	1
======== CHANNEL f1 ========	
NUC1	1H
P1	14.00 usec
PL1	-1.80 dB
PL1W	14.82738590 W
SFO1	400.1324710 MHz
SI	32768
SF	400.1300029 MHz
WDW	EM
SSB	0
LB	0.30 Hz
GB	0
PC	1.00

4CF3PhBH2P(n-Bu)3 recolumn

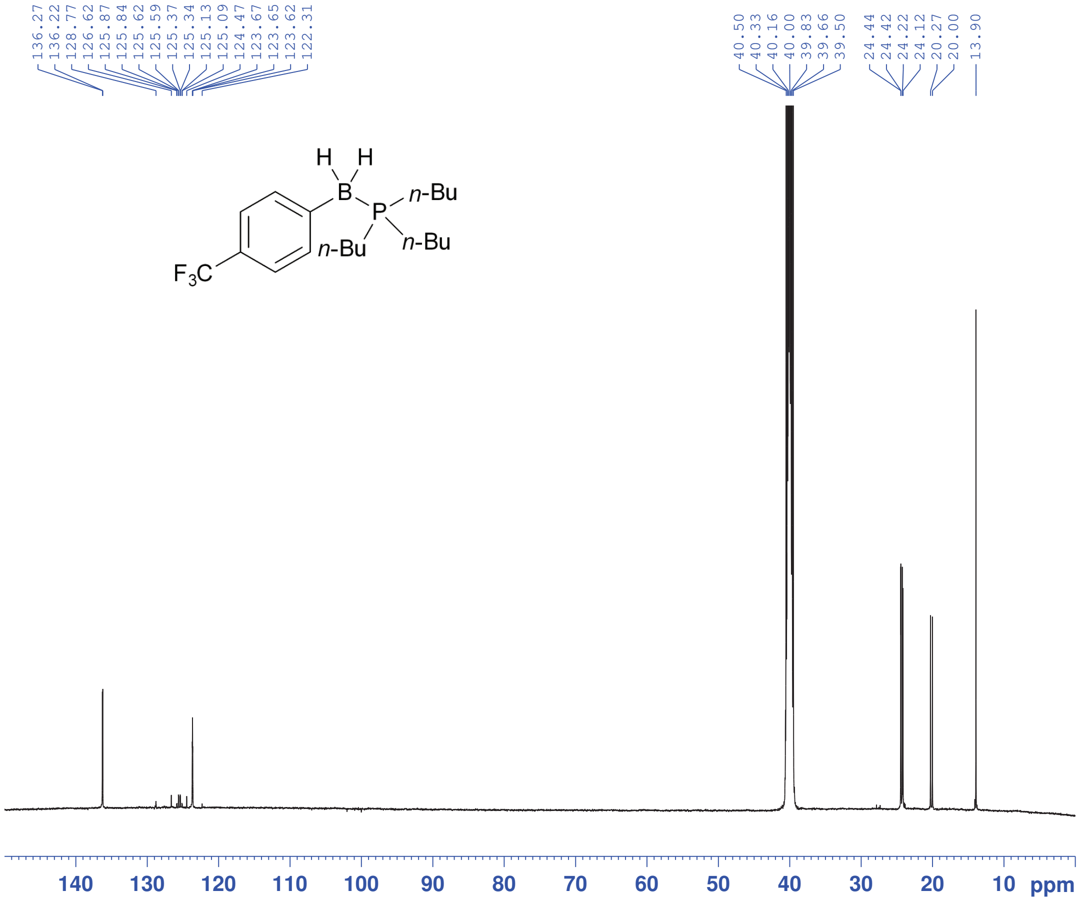

IBB-nmr Analysis

NAME	July28-2022
EXPNO	2
PROCNO	1
Date_	20220729
Time	5.28
INSTRUM	av500
PROBHD	5 mm CPDCH 13C
PULPROG	zgpg30
TD	65536
SOLVENT	DMSO
NS	8192
DS	4
SWH	30030.029 Hz
FIDRES	0.458222 Hz
AQ	1.0912410 sec
RG	35.9
DW	16.650 usec
DE	20.00 usec
TE	298.0 K
D1	2.00000000 sec
D11	0.03000000 sec
TD0	1
======== CHANNEL f1 ========	
NUC1	13C
P1	10.00 usec
PL1	-5.00 dB
PL1W	14.29790783 W
SFO1	125.7703643 MHz
======== CHANNEL f2 ========	
CPDPRG2	waltz16
NUC2	1H
PCPD2	80.00 usec
PL2	-4.00 dB
PL12	14.06 dB
PL13	17.00 dB
PL2W	6.30957365 W
PL12W	0.09862794 W
PL13W	0.05011872 W
SFO2	500.1320005 MHz
SI	32768
SF	125.7577866 MHz
WDW	EM
SSB	0
LB	1.00 Hz
GB	0
PC	0.30

4CF3PhBH2P(n-Bu)3 recolumn

IBB-nmr Analysis

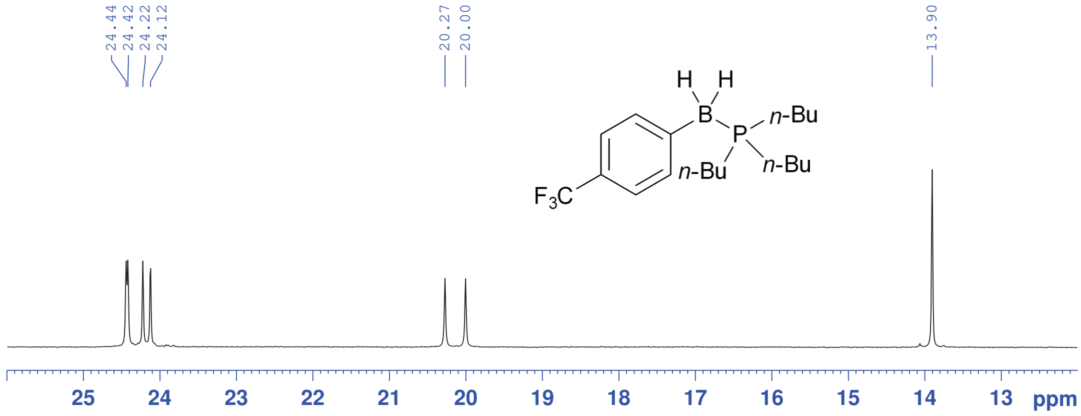

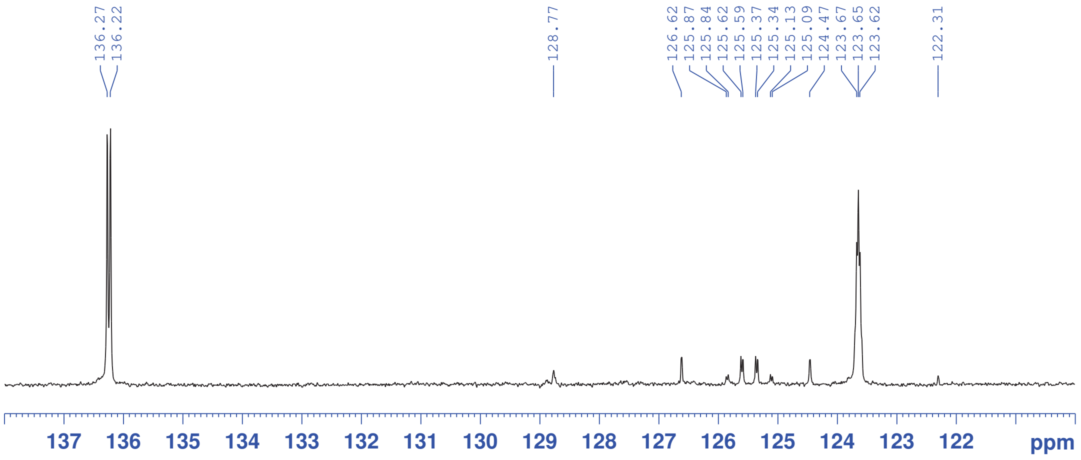

NAME	July28-2022
EXPNO	2
PROCNO	1
Date_	20220729
Time	5.28
INSTRUM	av500
PROBHD	5 mm CPDCH 13C
PULPROG	zgpg30
TD	65536
SOLVENT	DMSO
NS	8192
DS	4
SWH	30030.029 Hz
FIDRES	0.458222 Hz
AQ	1.0912410 sec
RG	35.9
DW	16.650 usec
DE	20.00 usec
TE	298.0 K
D1	2.00000000 sec
D11	0.03000000 sec
TD0	1

======== CHANNEL f1 ========

NUC1	13C
P1	10.00 usec
PL1	-5.00 dB
PL1W	14.29790783 W
SFO1	125.7703643 MHz

======== CHANNEL f2 ========

CPDPRG2	waltz16
NUC2	1H
PCPD2	80.00 usec
PL2	-4.00 dB
PL12	14.06 dB
PL13	17.00 dB
PL2W	6.30957365 W
PL12W	0.09862794 W
PL13W	0.05011872 W
SFO2	500.1320005 MHz
SI	32768
SF	125.7577866 MHz
WDW	EM
SSB	0
LB	1.00 Hz
GB	0
PC	0.30

4CF3PhBH2P(n-Bu)3 recolumn

IBB-nmr Analysis

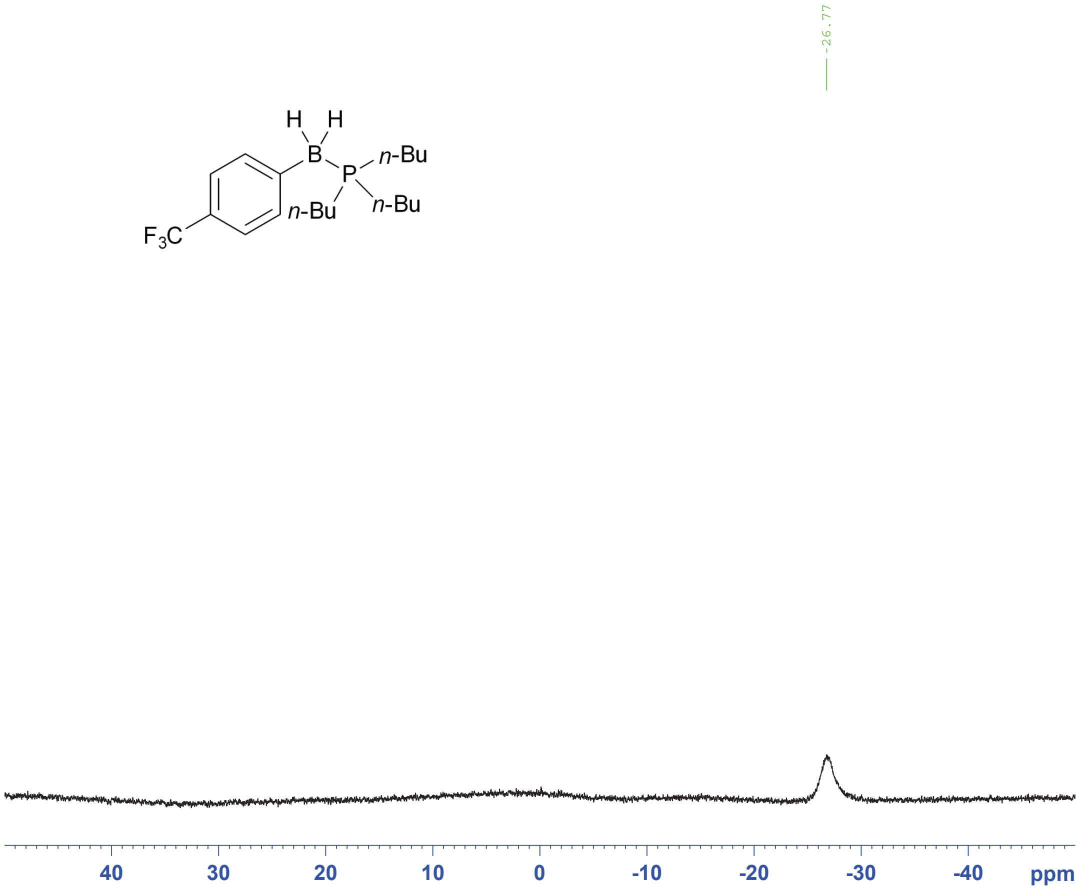

NAME	July26-2022
EXPNO	3
PROCNO	1
Date_	20220726
Time	21.39
INSTRUM	av400
PROBHD	5 mm PABBO BB-
PULPROG	zgig
TD	32768
SOLVENT	DMSO
NS	128
DS	4
SWH	20000.000 Hz
FIDRES	0.610352 Hz
AQ	0.8192500 sec
RG	203
DW	25.000 usec
DE	20.00 usec
TE	298.4 K
D1	1.00000000 sec
D11	0.03000000 sec
TD0	1

======== CHANNEL f1 ========

NUC1	11B
P1	6.30 usec
PL1	-2.00 dB
SFO1	128.3776050 MHz

======== CHANNEL f2 ========

CPDPRG2	waltz16
NUC2	1H
PCPD2	80.00 usec
PL2	-1.70 dB
PL12	13.80 dB
PL2W	14.48987389 W
PL12W	0.40838012 W
SFO2	400.1316005 MHz
SI	32768
SF	128.3776183 MHz
WDW	EM
SSB	0
LB	2.00 Hz
GB	0
PC	1.40

4CF3PhBH2P(n-Bu)3 recolumn

IBB-nmr Analysis

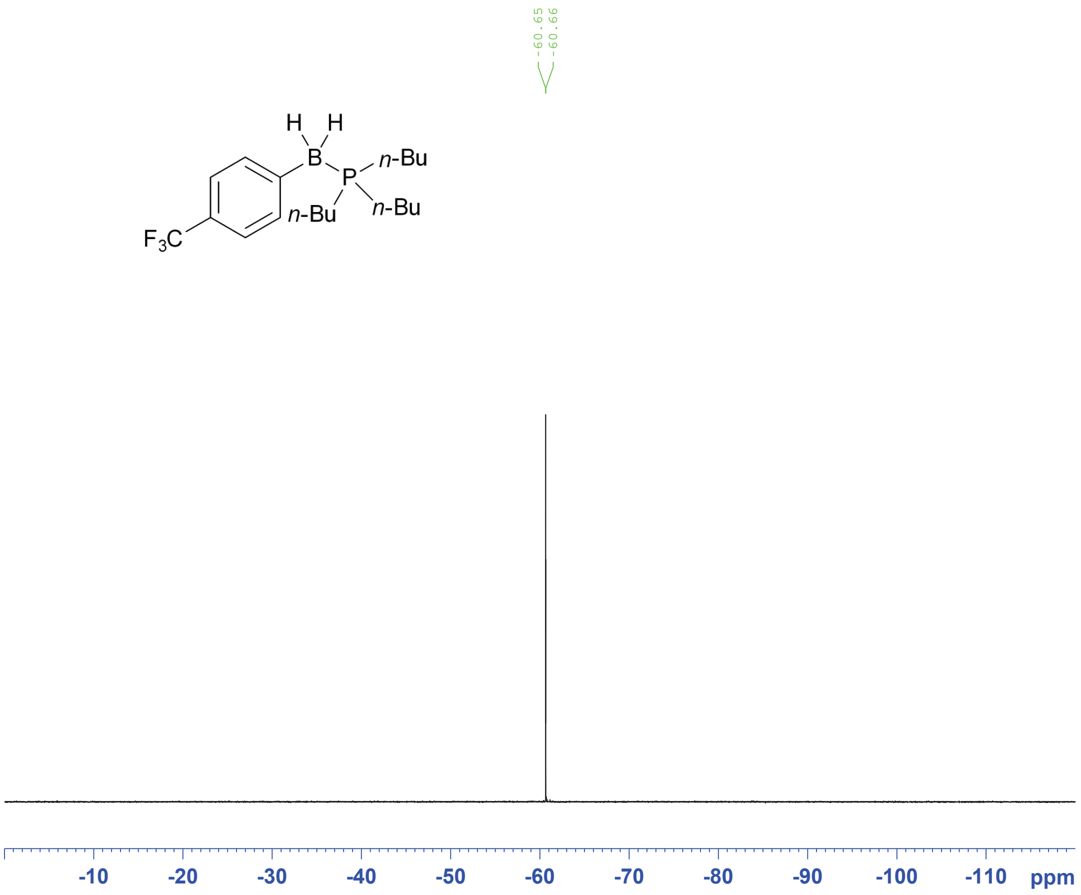

NAME	July26-2022
EXPNO	2
PROCNO	1
Date_	20220726
Time	21.32
INSTRUM	av400
PROBHD	5 mm PABBO BB-
PULPROG	zgfhigqn
TD	131072
SOLVENT	DMSO
NS	8
DS	4
SWH	89285.711 Hz
FIDRES	0.681196 Hz
AQ	0.7340532 sec
RG	203
DW	5.600 usec
DE	6.50 usec
TE	297.9 K
D1	1.00000000 sec
D11	0.03000000 sec
D12	0.00002000 sec
TD0	1
======== CHANNEL f1 ========	
NUC1	19F
P1	19.50 usec
PL1	-4.00 dB
PL1W	16.40489006 W
SFO1	376.4607164 MHz
======== CHANNEL f2 ========	
CPDPRG2	waltz16
NUC2	1H
PCPD2	112.00 usec
PL2	-4.00 dB
PL12	18.00 dB
PL2W	24.60733604 W
PL12W	0.15526180 W
SFO2	400.1316005 MHz
SI	65536
SF	376.4984617 MHz
WDW	EM
SSB	0
LB	0.30 Hz
GB	0
PC	1.00

4CF3PhBH2P(n-Bu)3 recolumn

IBB-nmr Analysis

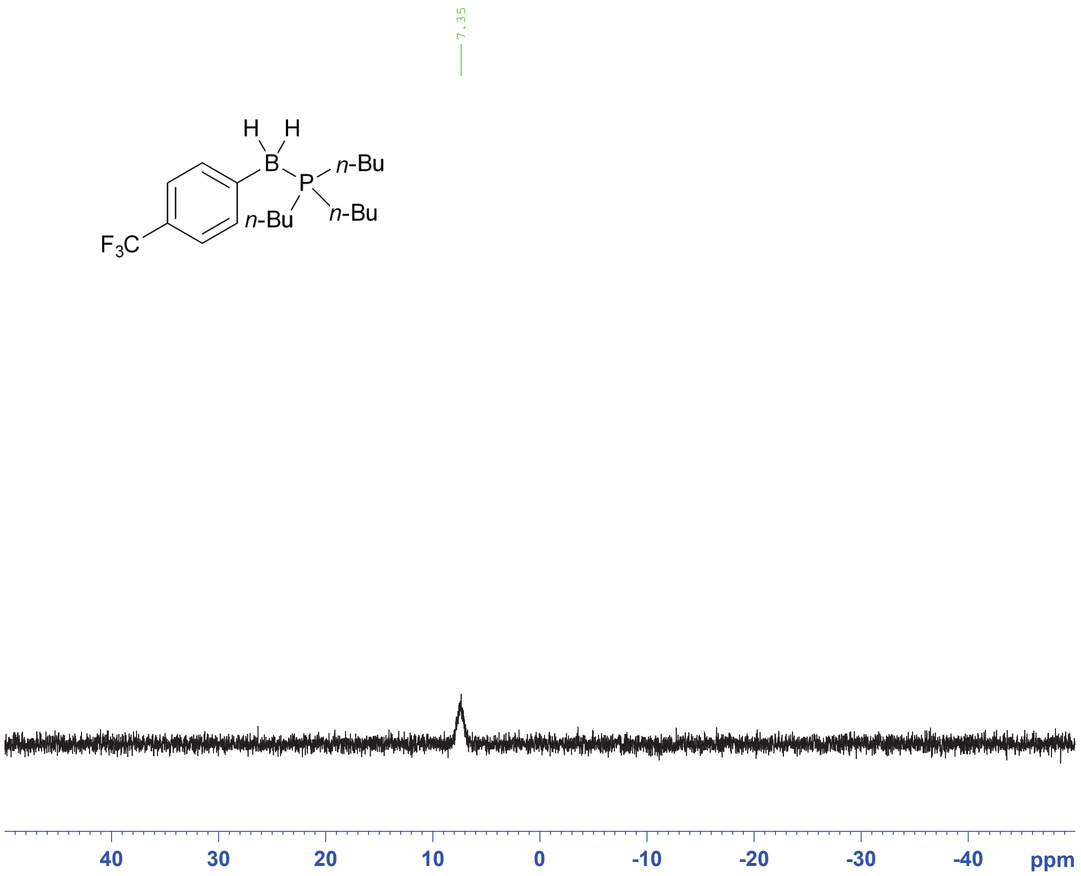

NAME	July26-2022
EXPNO	4
PROCNO	1
Date_	20220726
Time	21.45
INSTRUM	av400
PROBHD	5 mm PABBO BB-
PULPROG	zg30
TD	65536
SOLVENT	DMSO
NS	64
DS	4
SWH	64102.563 Hz
FIDRES	0.978127 Hz
AQ	0.5112308 sec
RG	203
DW	7.800 usec
DE	6.50 usec
TE	297.8 K
D1	2.00000000 sec
TD0	1
======== CHANNEL f1 ========	
NUC1	31P
P1	10.00 usec
PL1	-0.70 dB
PL1W	30.10233498 W
SFO1	161.9674942 MHz
SI	32768
SF	161.9754962 MHz
WDW	EM
SSB	0
LB	1.00 Hz
GB	0
PC	1.40

4-CF3PhBH2P(cyclopentyl)3

B-4-(Trifluoromethyl)phenyl tricyclopentylphosphine–borane (36)

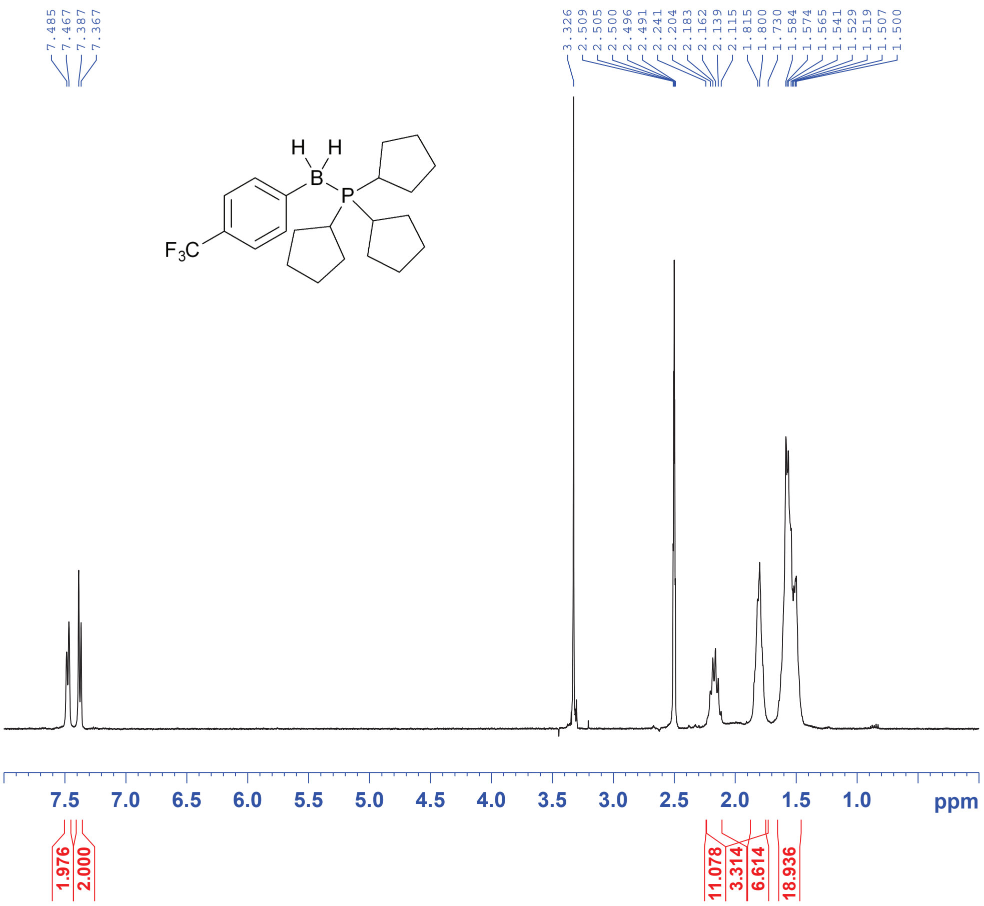

IBB-nmr Analysis

NAME	Feb17-2022
EXPNO	2
PROCNO	1
Date_	20220217
Time	22.09
INSTRUM	av400
PROBHD	5 mm PABBO BB-
PULPROG	zg30
TD	65536
SOLVENT	DMSO
NS	4
DS	2
SWH	8223.685 Hz
FIDRES	0.125483 Hz
AQ	3.9846387 sec
RG	203
DW	60.800 usec
DE	6.50 usec
TE	296.2 K
D1	1.00000000 sec
TD0	1
======== CHANNEL f1 ========	
NUC1	1H
P1	14.00 usec
PL1	-1.80 dB
PL1W	14.82738590 W
SFO1	400.1324710 MHz
SI	32768
SF	400.1300031 MHz
WDW	EM
SSB	0
LB	0.30 Hz
GB	0
PC	1.00

4CF3PhBH2P(cyclopentyl)3

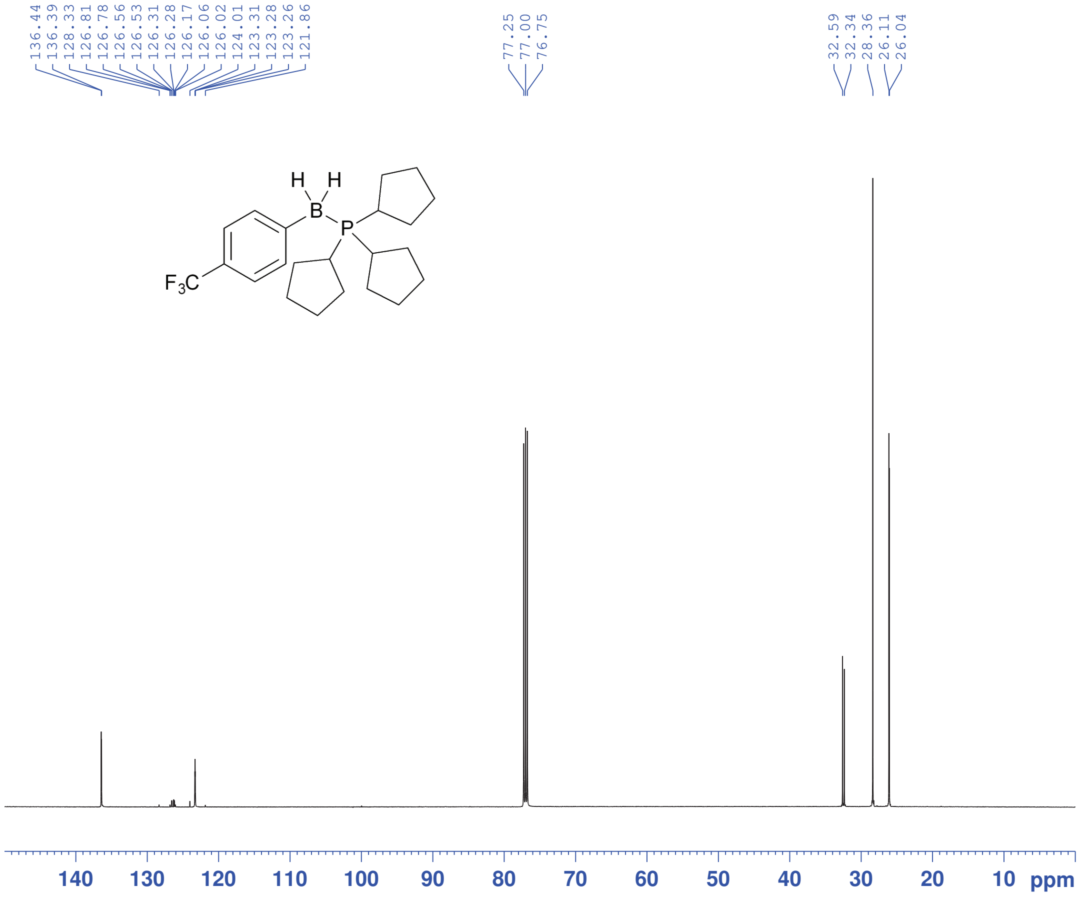

IBB-nmr Analysis

NAME	Mar08-2024
EXPNO	13
PROCNO	1
Date_	20240308
Time	7.10
INSTRUM	av500
PROBHD	5 mm CPDCH 13C
PULPROG	zgpg30
TD	65536
SOLVENT	CDCl3
NS	512
DS	4
SWH	30030.029 Hz
FIDRES	0.458222 Hz
AQ	1.0912410 sec
RG	35.9
DW	16.650 usec
DE	20.00 usec
TE	298.1 K
D1	2.00000000 sec
D11	0.03000000 sec
TD0	1
======== CHANNEL f1 ========	
NUC1	13C
P1	10.00 usec
PL1	-5.00 dB
PL1W	14.29790783 W
SFO1	125.7703643 MHz
======== CHANNEL f2 ========	
CPDPRG2	waltz16
NUC2	1H
PCPD2	80.00 usec
PL2	-4.00 dB
PL12	14.06 dB
PL13	17.00 dB
PL2W	6.30957365 W
PL12W	0.09862794 W
PL13W	0.05011872 W
SFO2	500.1320005 MHz
SI	32768
SF	125.7577928 MHz
WDW	EM
SSB	0
LB	1.00 Hz
GB	0
PC	0.30

4CF3PhBH2P(cyclopentyl)3

IBB-nmr Analysis

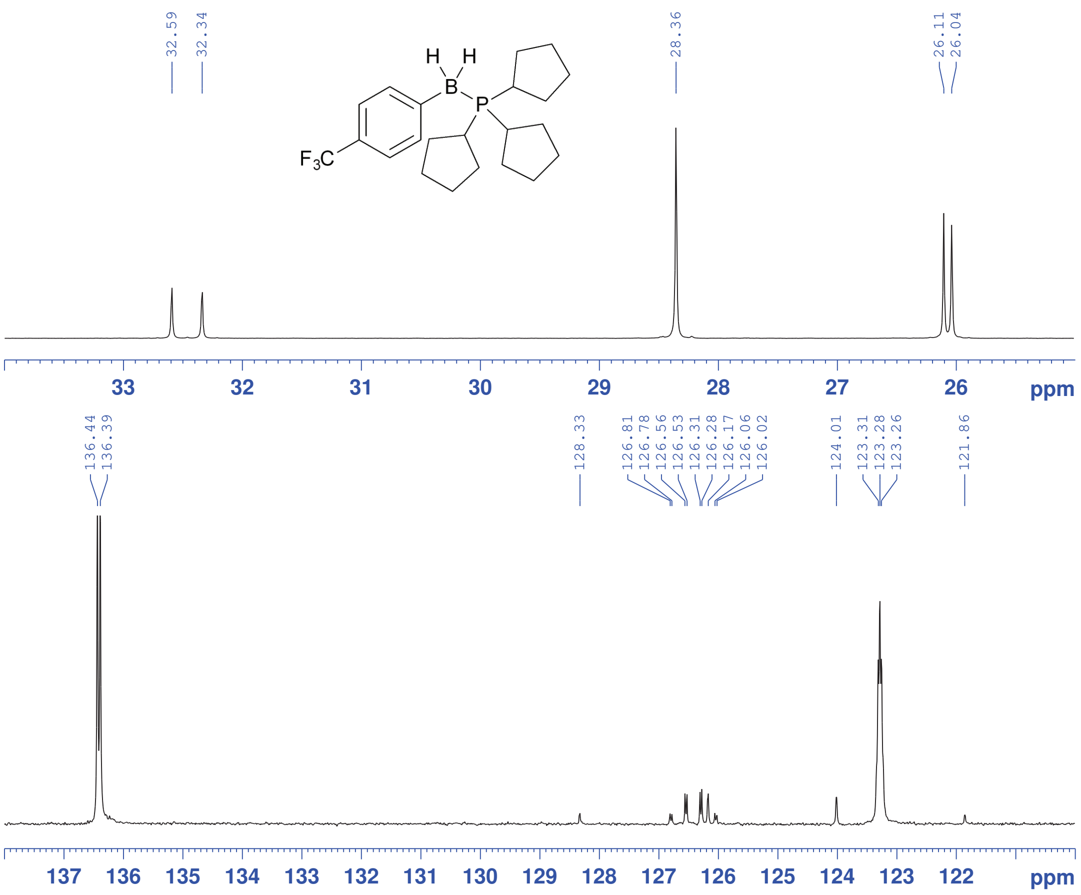

NAME	Mar08-2024
EXPNO	13
PROCNO	1
Date_	20240308
Time	7.10
INSTRUM	av500
PROBHD	5 mm CPDCH 13C
PULPROG	zgpg30
TD	65536
SOLVENT	CDCl3
NS	512
DS	4
SWH	30030.029 Hz
FIDRES	0.458222 Hz
AQ	1.0912410 sec
RG	35.9
DW	16.650 usec
DE	20.00 usec
TE	298.1 K
D1	2.00000000 sec
D11	0.03000000 sec
TD0	1
======== CHANNEL f1 ========	
NUC1	13C
P1	10.00 usec
PL1	-5.00 dB
PL1W	14.29790783 W
SFO1	125.7703643 MHz
======== CHANNEL f2 ========	
CPDPRG2	waltz16
NUC2	1H
PCPD2	80.00 usec
PL2	-4.00 dB
PL12	14.06 dB
PL13	17.00 dB
PL2W	6.30957365 W
PL12W	0.09862794 W
PL13W	0.05011872 W
SFO2	500.1320005 MHz
SI	32768
SF	125.7577928 MHz
WDW	EM
SSB	0
LB	1.00 Hz
GB	0
PC	0.30

4-CF3PhBH2P(cyclopentyl)3

IBB-nmr Analysis

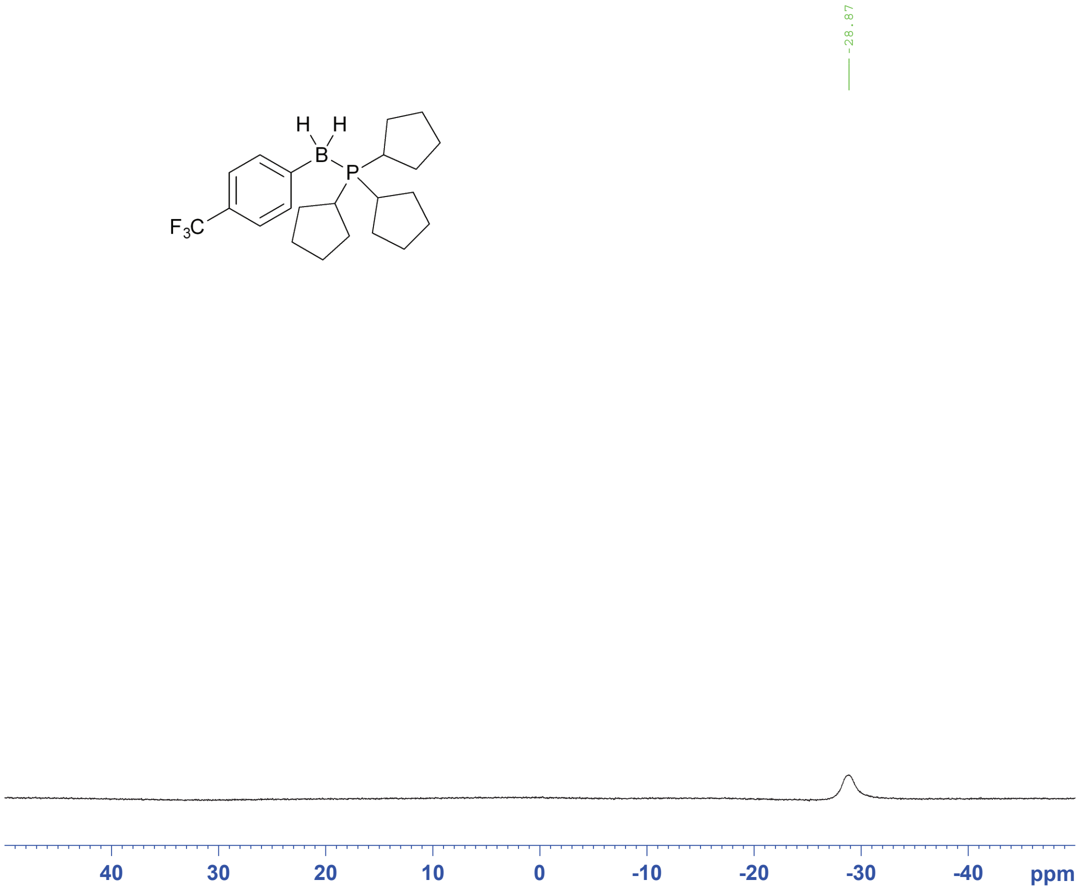

NAME	Feb17-2022
EXPNO	4
PROCNO	1
Date_	20220217
Time	22.20
INSTRUM	av400
PROBHD	5 mm PABBO BB-
PULPROG	zgig
TD	32768
SOLVENT	DMSO
NS	128
DS	4
SWH	20000.000 Hz
FIDRES	0.610352 Hz
AQ	0.8192500 sec
RG	203
DW	25.000 usec
DE	20.00 usec
TE	296.8 K
D1	1.00000000 sec
D11	0.03000000 sec
TD0	1
======== CHANNEL f1 ========	
NUC1	11B
P1	6.30 usec
PL1	-2.00 dB
SFO1	128.3776050 MHz
======== CHANNEL f2 ========	
CPDPRG2	waltz16
NUC2	1H
PCPD2	80.00 usec
PL2	-1.70 dB
PL12	13.80 dB
PL2W	14.48987389 W
PL12W	0.40838012 W
SFO2	400.1316005 MHz
SI	32768
SF	128.3776183 MHz
WDW	EM
SSB	0
LB	2.00 Hz
GB	0
PC	1.40

4-CF3PhBH2P(cyclopentyl)3

IBB-nmr Analysis

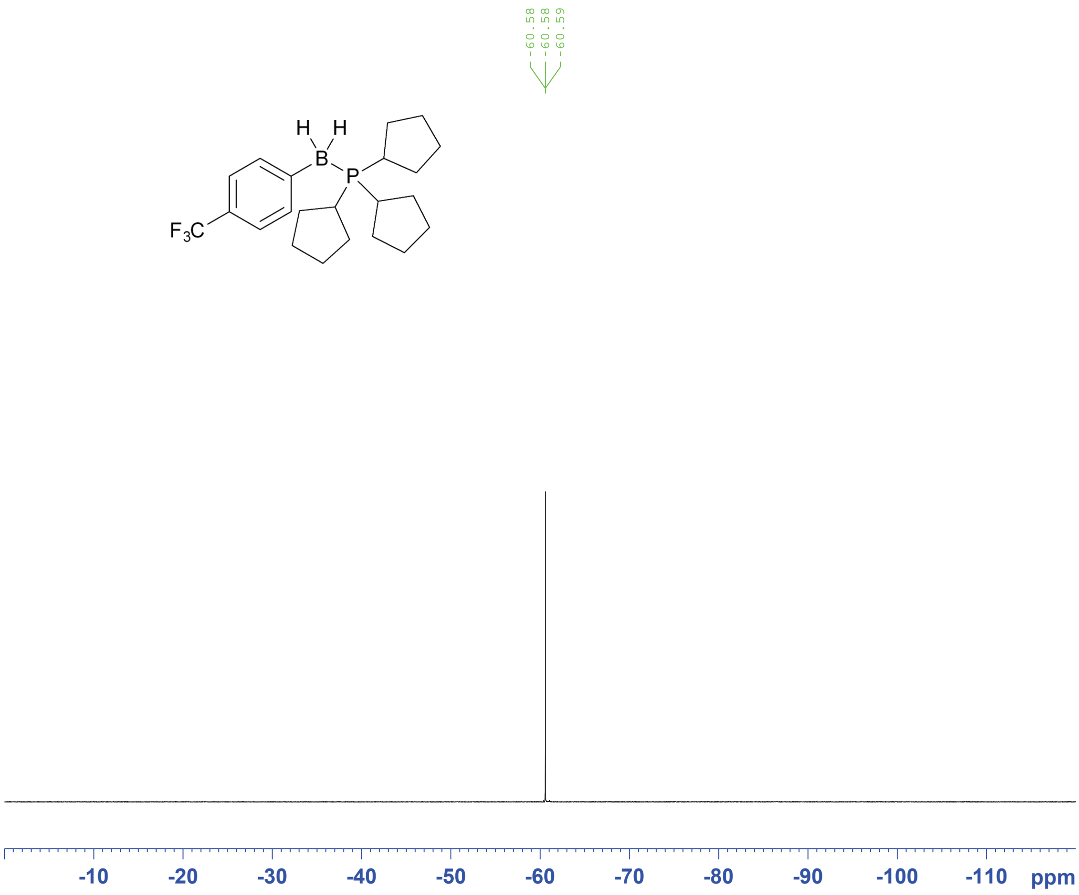

NAME	Feb17-2022	
EXPNO	5	
PROCNO	1	
Date_	20220217	
Time	22.23	
INSTRUM	av400	
PROBHD	5 mm PABBO BB-	
PULPROG	zgfhigqn	
TD	131072	
SOLVENT	DMSO	
NS	8	
DS	4	
SWH	89285.711	Hz
FIDRES	0.681196	Hz
AQ	0.7340532	sec
RG	203	
DW	5.600	usec
DE	6.50	usec
TE	296.4	K
D1	1.00000000	sec
D11	0.03000000	sec
D12	0.00002000	sec
TD0	1	
======== CHANNEL f1 ========		
NUC1	19F	
P1	19.50	usec
PL1	-4.00	dB
PL1W	16.40489006	W
SFO1	376.4607164	MHz
======== CHANNEL f2 ========		
CPDPRG2	waltz16	
NUC2	1H	
PCPD2	112.00	usec
PL2	-4.00	dB
PL12	18.00	dB
PL2W	24.60733604	W
PL12W	0.15526180	W
SFO2	400.1316005	MHz
SI	65536	
SF	376.4984617	MHz
WDW	EM	
SSB	0	
LB	0.30	Hz
GB	0	
PC	1.00	

4-CF3PhBH2P(cyclopentyl)3

IBB-nmr Analysis

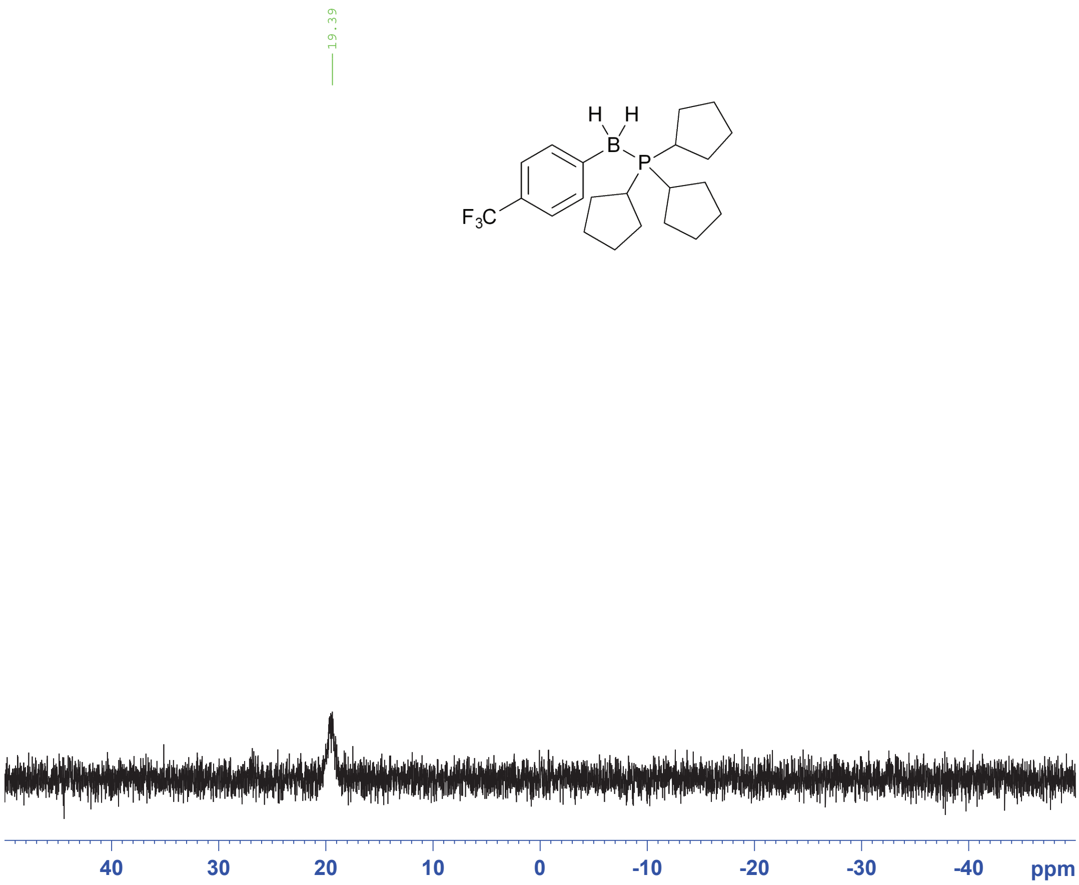

NAME	Feb17-2022	
EXPNO	3	
PROCNO	1	
Date_	20220217	
Time	22.12	
INSTRUM	av400	
PROBHD	5 mm PABBO BB-	
PULPROG	zg30	
TD	65536	
SOLVENT	DMSO	
NS	8	
DS	4	
SWH	64102.563	Hz
FIDRES	0.978127	Hz
AQ	0.5112308	sec
RG	203	
DW	7.800	usec
DE	6.50	usec
TE	296.3	K
D1	2.00000000	sec
TD0	1	
======== CHANNEL f1 ========		
NUC1	31P	
P1	10.00	usec
PL1	-0.70	dB
PL1W	30.10233498	W
SFO1	161.9674942	MHz
SI	32768	
SF	161.9754962	MHz
WDW	EM	
SSB	0	
LB	1.00	Hz
GB	0	
PC	1.40	

B-4-(Trifluoromethyl)phenyl tricyclohexylphosphine–borane (37)

4CF3PhBH2P(cyclohexyl)3

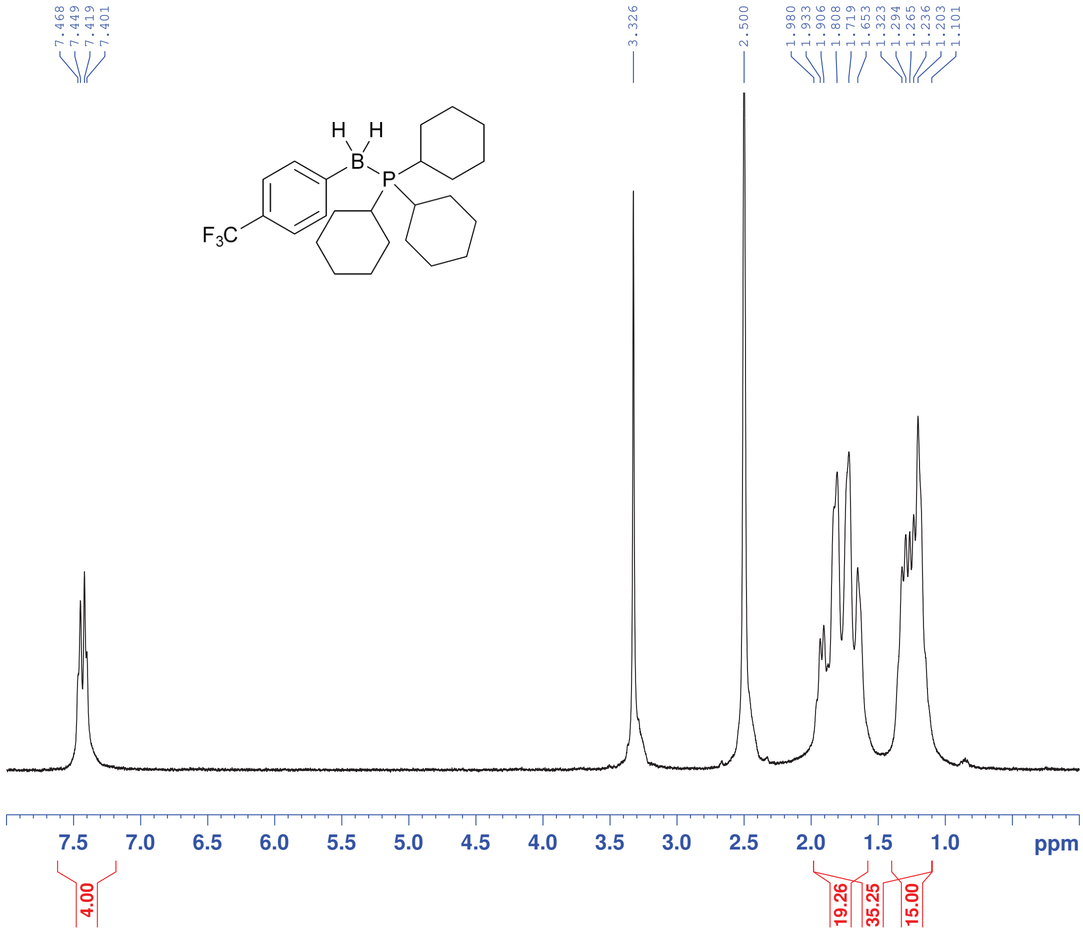

IBB-nmr Analysis

```
NAME          Apr07-2022
EXPNO                 10
PROCNO                 1
Date_           20220407
Time               23.20
INSTRUM            av400
PROBHD   5 mm PABBO BB-
PULPROG             zg30
TD                 65536
SOLVENT             DMSO
NS                    16
DS                     2
SWH             8223.685 Hz
FIDRES          0.125483 Hz
AQ             3.9846387 sec
RG                   203
DW                60.800 usec
DE                  6.50 usec
TE                 296.1 K
D1            1.00000000 sec
TD0                    1

======== CHANNEL f1 ========
NUC1                  1H
P1                 14.00 usec
PL1                -1.80 dB
PL1W         14.82738590 W
SFO1         400.1324710 MHz
SI                 32768
SF           400.1300035 MHz
WDW                   EM
SSB                    0
LB                  0.30 Hz
GB                     0
PC                  1.00
```

4CF3PhBH2P(cyclohexyl)3

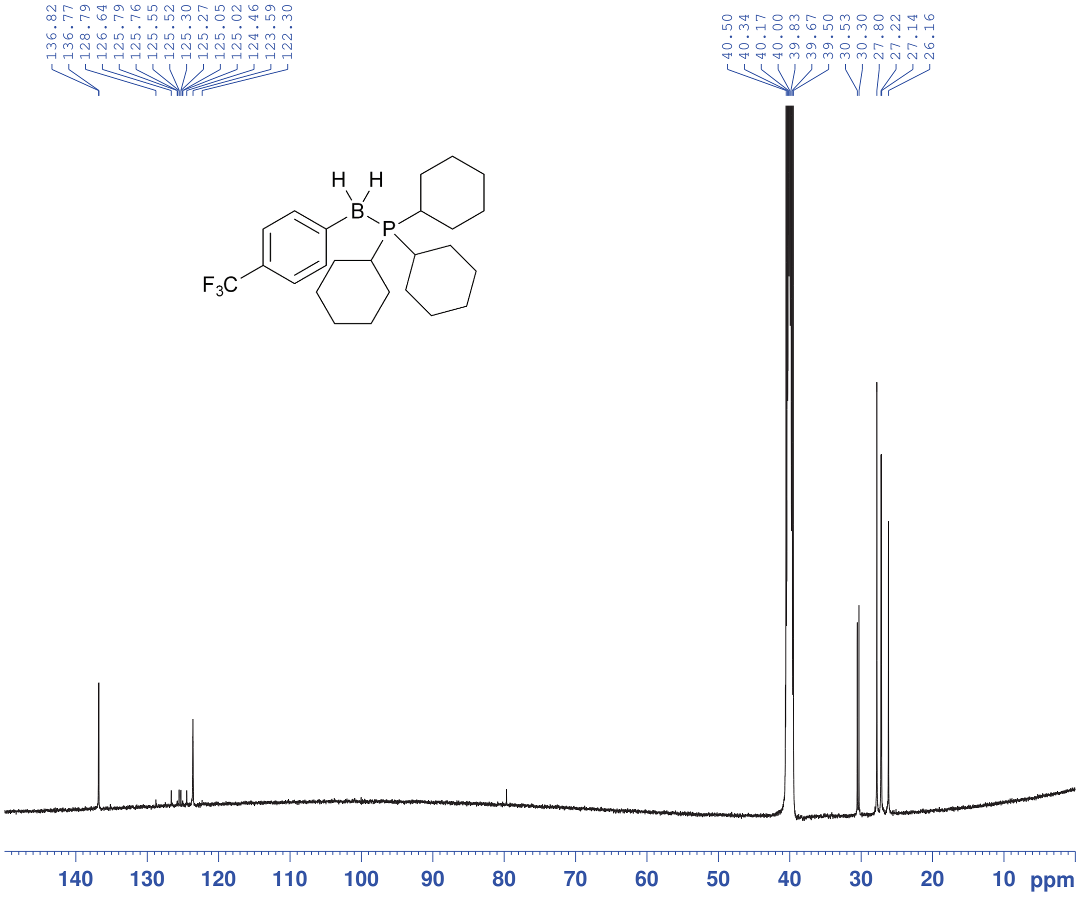

IBB-nmr Analysis

```
NAME          Mar06-2024
EXPNO                  9
PROCNO                 1
Date_           20240306
Time                5.01
INSTRUM            av500
PROBHD   5 mm CPDCH 13C
PULPROG           zgpg30
TD                 65536
SOLVENT             DMSO
NS                  2048
DS                     4
SWH            30030.029 Hz
FIDRES          0.458222 Hz
AQ             1.0912410 sec
RG                    57
DW                16.650 usec
DE                 20.00 usec
TE                 297.9 K
D1            2.00000000 sec
D11           0.03000000 sec
TD0                    1

======== CHANNEL f1 ========
NUC1                 13C
P1                 10.00 usec
PL1                -5.00 dB
PL1W         14.29790783 W
SFO1         125.7703643 MHz

======== CHANNEL f2 ========
CPDPRG2          waltz16
NUC2                  1H
PCPD2              80.00 usec
PL2                -4.00 dB
PL12               14.06 dB
PL13               17.00 dB
PL2W          6.30957365 W
PL12W         0.09862794 W
PL13W         0.05011872 W
SFO2         500.1320005 MHz
SI                 32768
SF           125.7577867 MHz
WDW                   EM
SSB                    0
LB                  1.00 Hz
GB                     0
PC                  0.30
```

4CF3PhBH2P(cyclohexyl)3

IBB-nmr Analysis

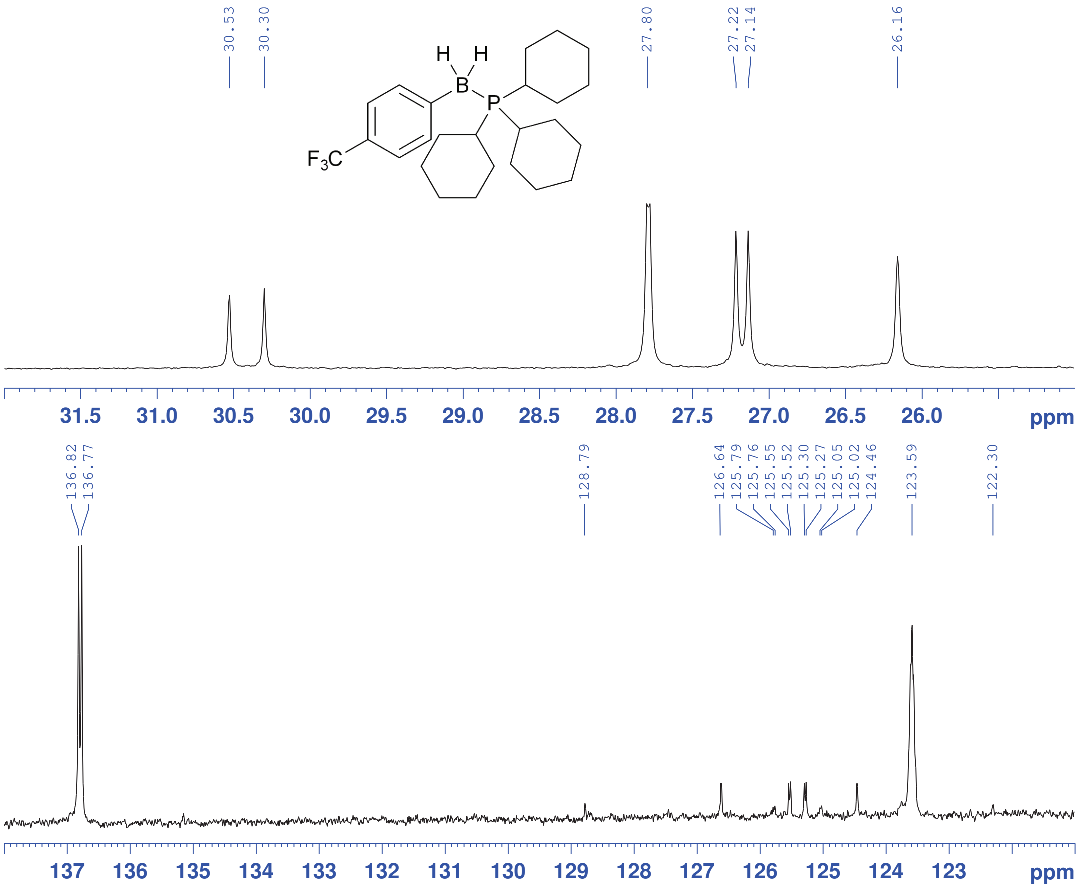

NAME	Mar06-2024
EXPNO	9
PROCNO	1
Date_	20240306
Time	5.01
INSTRUM	av500
PROBHD	5 mm CPDCH 13C
PULPROG	zgpg30
TD	65536
SOLVENT	DMSO
NS	2048
DS	4
SWH	30030.029 Hz
FIDRES	0.458222 Hz
AQ	1.0912410 sec
RG	57
DW	16.650 usec
DE	20.00 usec
TE	297.9 K
D1	2.00000000 sec
D11	0.03000000 sec
TD0	1
======== CHANNEL f1 ========	
NUC1	13C
P1	10.00 usec
PL1	-5.00 dB
PL1W	14.29790783 W
SFO1	125.7703643 MHz
======== CHANNEL f2 ========	
CPDPRG2	waltz16
NUC2	1H
PCPD2	80.00 usec
PL2	-4.00 dB
PL12	14.06 dB
PL13	17.00 dB
PL2W	6.30957365 W
PL12W	0.09862794 W
PL13W	0.05011872 W
SFO2	500.1320005 MHz
SI	32768
SF	125.7577867 MHz
WDW	EM
SSB	0
LB	1.00 Hz
GB	0
PC	0.30

4CF3PhBH2P(cyclohexyl)3

IBB-nmr Analysis

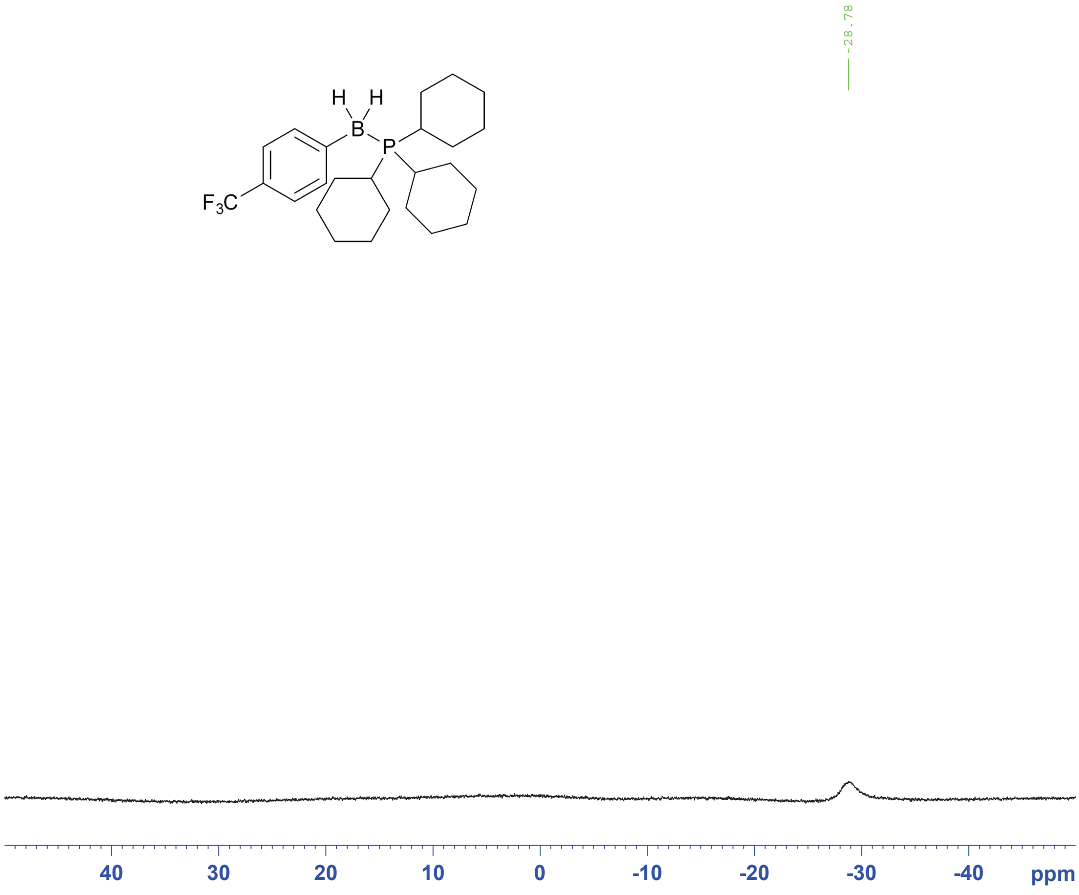

NAME	Mar27-2022
EXPNO	5
PROCNO	1
Date_	20220327
Time	22.54
INSTRUM	av400
PROBHD	5 mm PABBO BB-
PULPROG	zgig
TD	32768
SOLVENT	DMSO
NS	128
DS	4
SWH	20000.000 Hz
FIDRES	0.610352 Hz
AQ	0.8192500 sec
RG	203
DW	25.000 usec
DE	20.00 usec
TE	296.1 K
D1	1.00000000 sec
D11	0.03000000 sec
TD0	1
======== CHANNEL f1 ========	
NUC1	11B
P1	6.30 usec
PL1	-2.00 dB
SFO1	128.3776050 MHz
======== CHANNEL f2 ========	
CPDPRG2	waltz16
NUC2	1H
PCPD2	80.00 usec
PL2	-1.70 dB
PL12	13.80 dB
PL2W	14.48987389 W
PL12W	0.40838012 W
SFO2	400.1316005 MHz
SI	32768
SF	128.3776183 MHz
WDW	EM
SSB	0
LB	2.00 Hz
GB	0
PC	1.40

4CF3PhBH2P(cyclohexyl)3

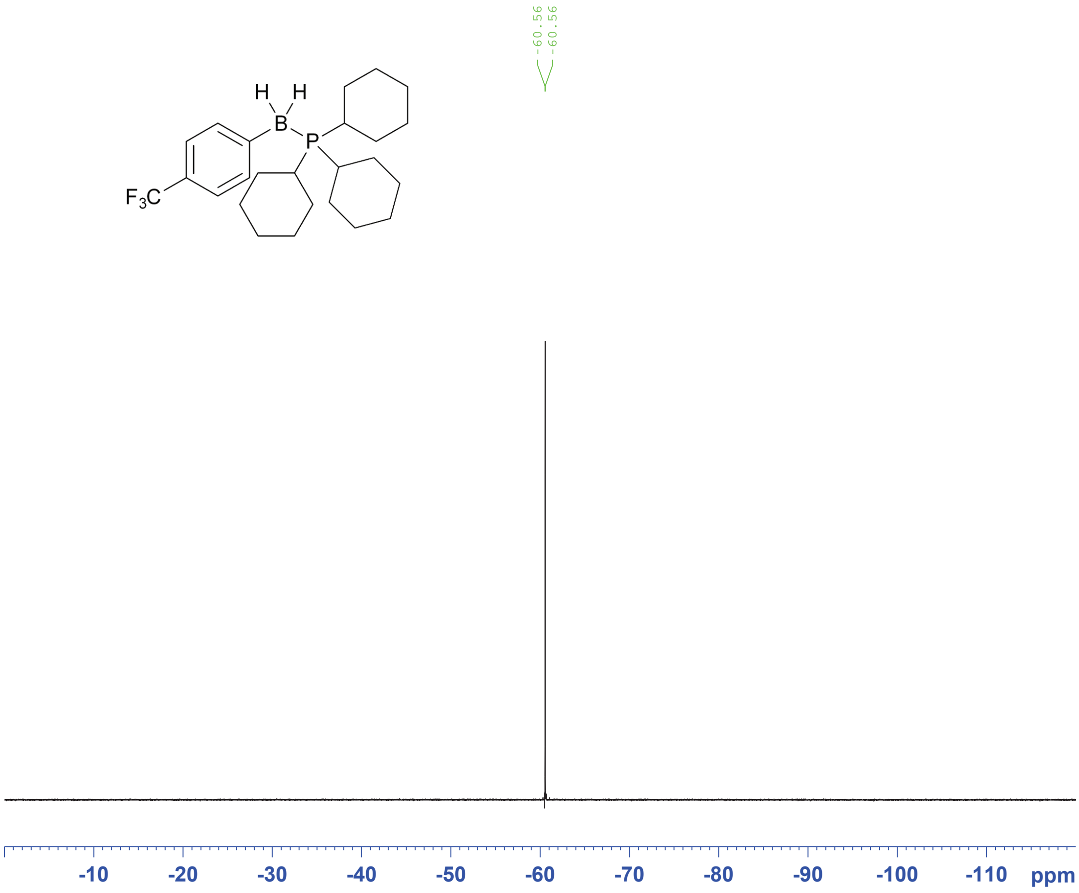

IBB-nmr Analysis

NAME	Mar27-2022	
EXPNO	3	
PROCNO	1	
Date_	20220327	
Time	22.42	
INSTRUM	av400	
PROBHD	5 mm PABBO BB-	
PULPROG	zgfhigqn	
TD	131072	
SOLVENT	DMSO	
NS	8	
DS	4	
SWH	89285.711	Hz
FIDRES	0.681196	Hz
AQ	0.7340532	sec
RG	203	
DW	5.600	usec
DE	6.50	usec
TE	295.6	K
D1	1.00000000	sec
D11	0.03000000	sec
D12	0.00002000	sec
TD0	1	

======== CHANNEL f1 ========

NUC1	19F	
P1	19.50	usec
PL1	-4.00	dB
PL1W	16.40489006	W
SFO1	376.4607164	MHz

======== CHANNEL f2 ========

CPDPRG2	waltz16	
NUC2	1H	
PCPD2	112.00	usec
PL2	-4.00	dB
PL12	18.00	dB
PL2W	24.60733604	W
PL12W	0.15526180	W
SFO2	400.1316005	MHz
SI	65536	
SF	376.4984617	MHz
WDW	EM	
SSB	0	
LB	0.30	Hz
GB	0	
PC	1.00	

4CF3PhBH2P(cyclohexyl)3

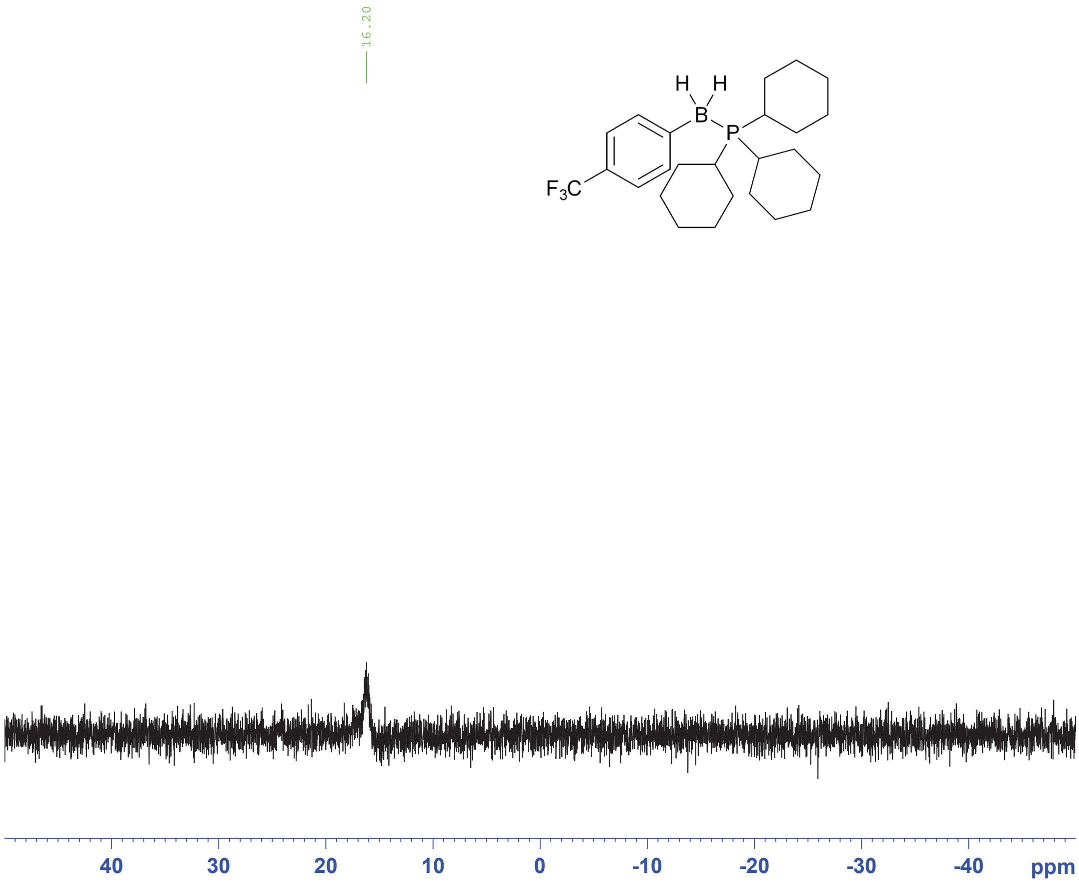

IBB-nmr Analysis

NAME	Mar27-2022	
EXPNO	4	
PROCNO	1	
Date_	20220327	
Time	22.46	
INSTRUM	av400	
PROBHD	5 mm PABBO BB-	
PULPROG	zg30	
TD	65536	
SOLVENT	DMSO	
NS	16	
DS	4	
SWH	64102.563	Hz
FIDRES	0.978127	Hz
AQ	0.5112308	sec
RG	203	
DW	7.800	usec
DE	6.50	usec
TE	295.5	K
D1	2.00000000	sec
TD0	1	

======== CHANNEL f1 ========

NUC1	31P	
P1	10.00	usec
PL1	-0.70	dB
PL1W	30.10233498	W
SFO1	161.9674942	MHz
SI	32768	
SF	161.9754962	MHz
WDW	EM	
SSB	0	
LB	1.00	Hz
GB	0	
PC	1.40	

B-4-(Trifluoromethyl)phenyl dicyclohexylphosphine–borane (38)

4-CF3PhBH2PH(cyclohexyl)2

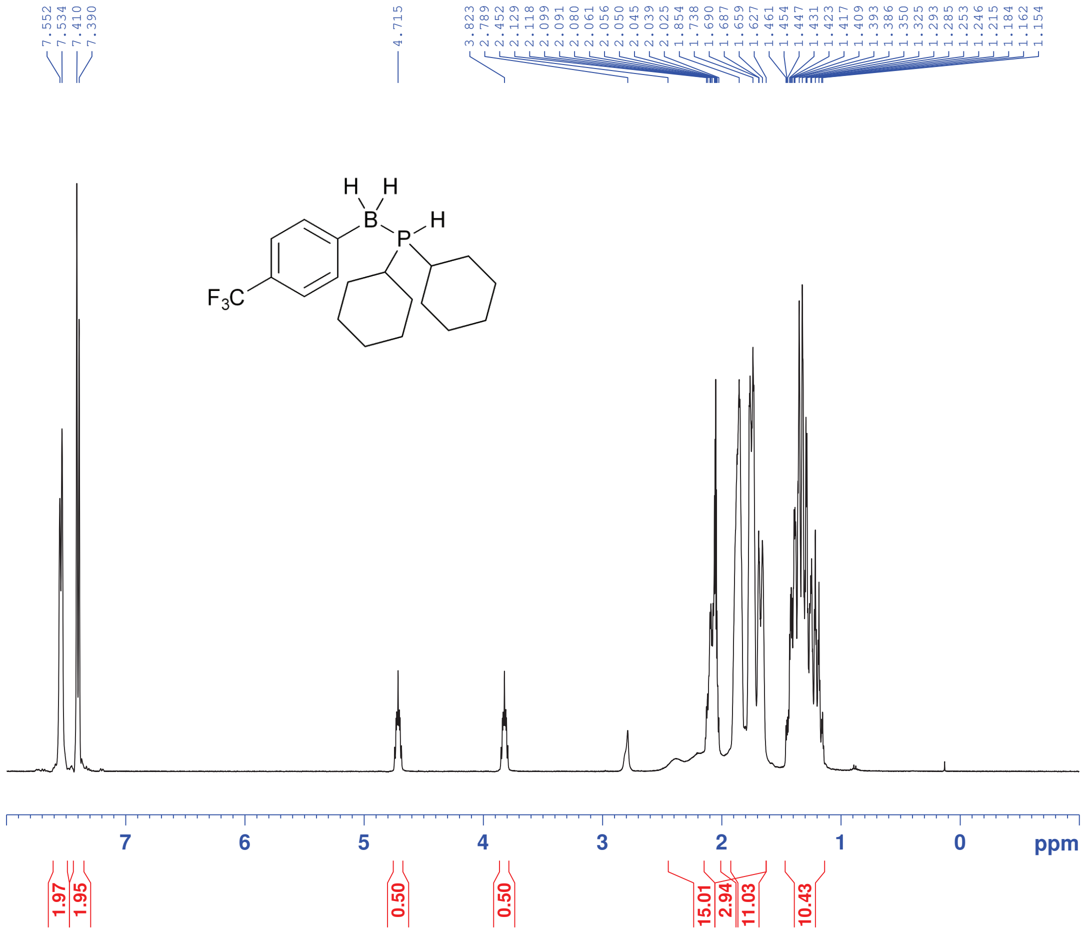

IBB-nmr Analysis

NAME	Aug8-2023
EXPNO	1
PROCNO	1
Date_	20230808
Time	8.22
INSTRUM	av400
PROBHD	5 mm PABBO BB-
PULPROG	zg30
TD	65536
SOLVENT	Acetone
NS	4
DS	2
SWH	8223.685 Hz
FIDRES	0.125483 Hz
AQ	3.9846387 sec
RG	57
DW	60.800 usec
DE	6.50 usec
TE	297.4 K
D1	1.00000000 sec
TD0	1
======== CHANNEL f1 ========	
NUC1	1H
P1	14.00 usec
PL1	-1.80 dB
PL1W	14.82738590 W
SFO1	400.1324710 MHz
SI	32768
SF	400.1300067 MHz
WDW	EM
SSB	0
LB	0.30 Hz
GB	0
PC	1.00

4CF3PhBH2PH(cyclohexyl)2

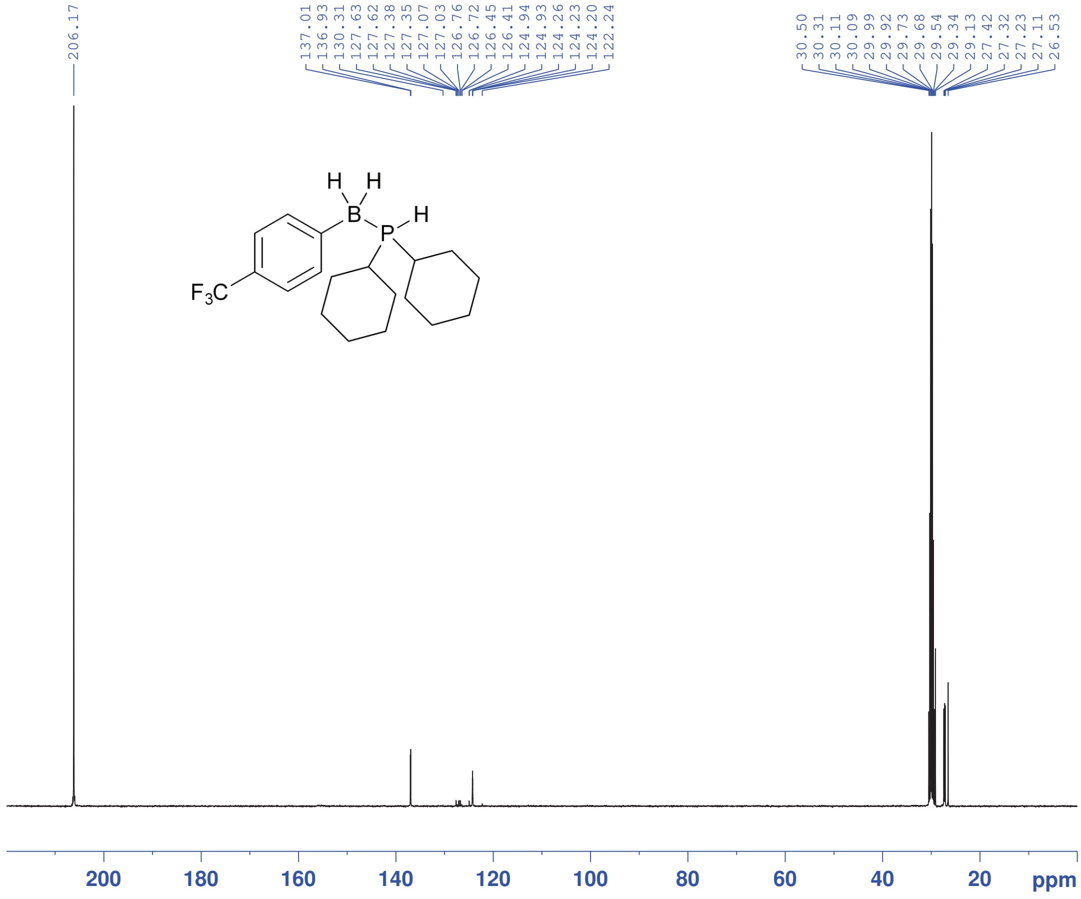

IBB-nmr Analysis

NAME	Aug8-2023
EXPNO	2
PROCNO	1
Date_	20230808
Time	9.27
INSTRUM	av400
PROBHD	5 mm PABBO BB-
PULPROG	zgpg30
TD	65536
SOLVENT	Acetone
NS	1024
DS	2
SWH	24038.461 Hz
FIDRES	0.366798 Hz
AQ	1.3631988 sec
RG	128
DW	20.800 usec
DE	6.50 usec
TE	298.7 K
D1	2.00000000 sec
D11	0.03000000 sec
TD0	1
======== CHANNEL f1 ========	
NUC1	13C
P1	9.00 usec
PL1	-3.00 dB
PL1W	73.17333984 W
SFO1	100.6228298 MHz
======== CHANNEL f2 ========	
CPDPRG2	waltz16
NUC2	1H
PCPD2	80.00 usec
PL2	-1.70 dB
PL12	13.80 dB
PL13	13.80 dB
PL2W	14.48987389 W
PL12W	0.40838012 W
PL13W	0.40838012 W
SFO2	400.1316005 MHz
SI	32768
SF	100.6126702 MHz
WDW	EM
SSB	0
LB	1.00 Hz
GB	0
PC	0.30

4CF3PhBH2PH(cyclohexyl)2

IBB-nmr Analysis

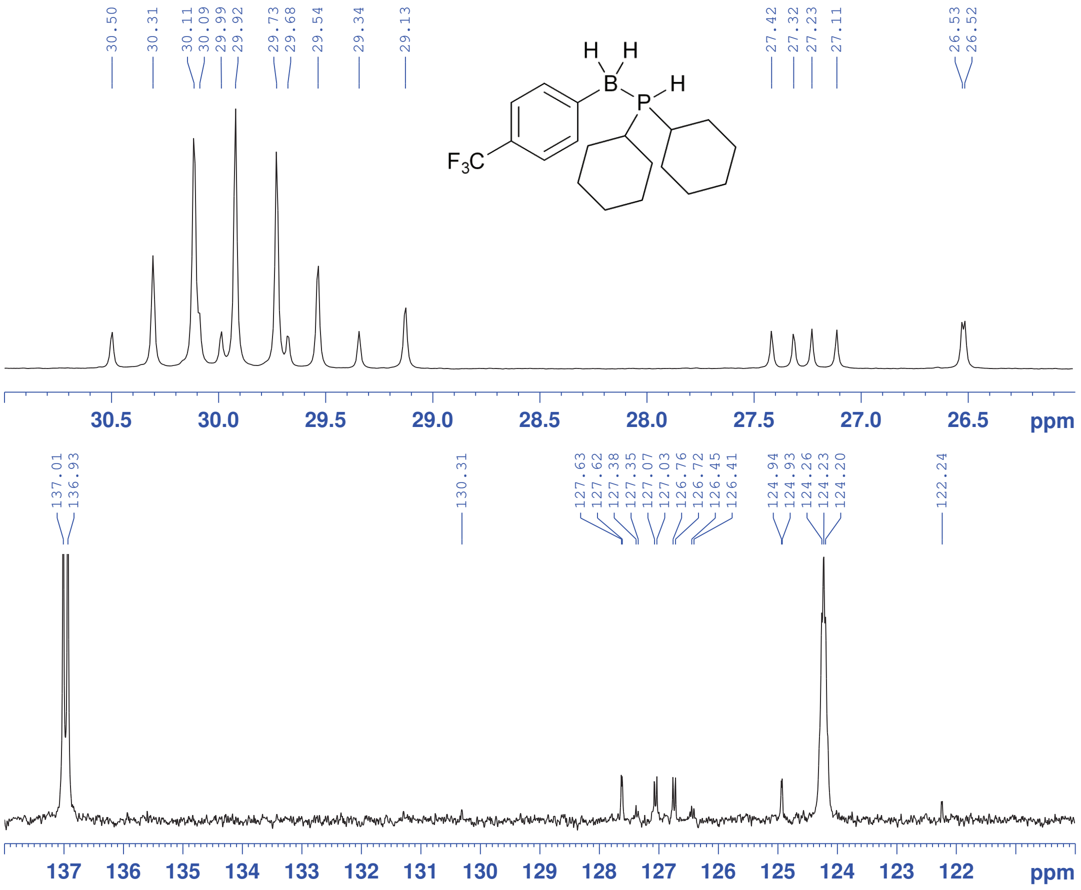

NAME	Aug8-2023
EXPNO	2
PROCNO	1
Date_	20230808
Time	9.27
INSTRUM	av400
PROBHD	5 mm PABBO BB-
PULPROG	zgpg30
TD	65536
SOLVENT	Acetone
NS	1024
DS	2
SWH	24038.461 Hz
FIDRES	0.366798 Hz
AQ	1.3631988 sec
RG	128
DW	20.800 usec
DE	6.50 usec
TE	298.7 K
D1	2.00000000 sec
D11	0.03000000 sec
TD0	1

======== CHANNEL f1 ========

NUC1	13C
P1	9.00 usec
PL1	-3.00 dB
PL1W	73.17333984 W
SFO1	100.6228298 MHz

======== CHANNEL f2 ========

CPDPRG2	waltz16
NUC2	1H
PCPD2	80.00 usec
PL2	-1.70 dB
PL12	13.80 dB
PL13	13.80 dB
PL2W	14.48987389 W
PL12W	0.40838012 W
PL13W	0.40838012 W
SFO2	400.1316005 MHz
SI	32768
SF	100.6126702 MHz
WDW	EM
SSB	0
LB	1.00 Hz
GB	0
PC	0.30

4CF3PhBH2PH(cyclohexyl)2

IBB-nmr Analysis

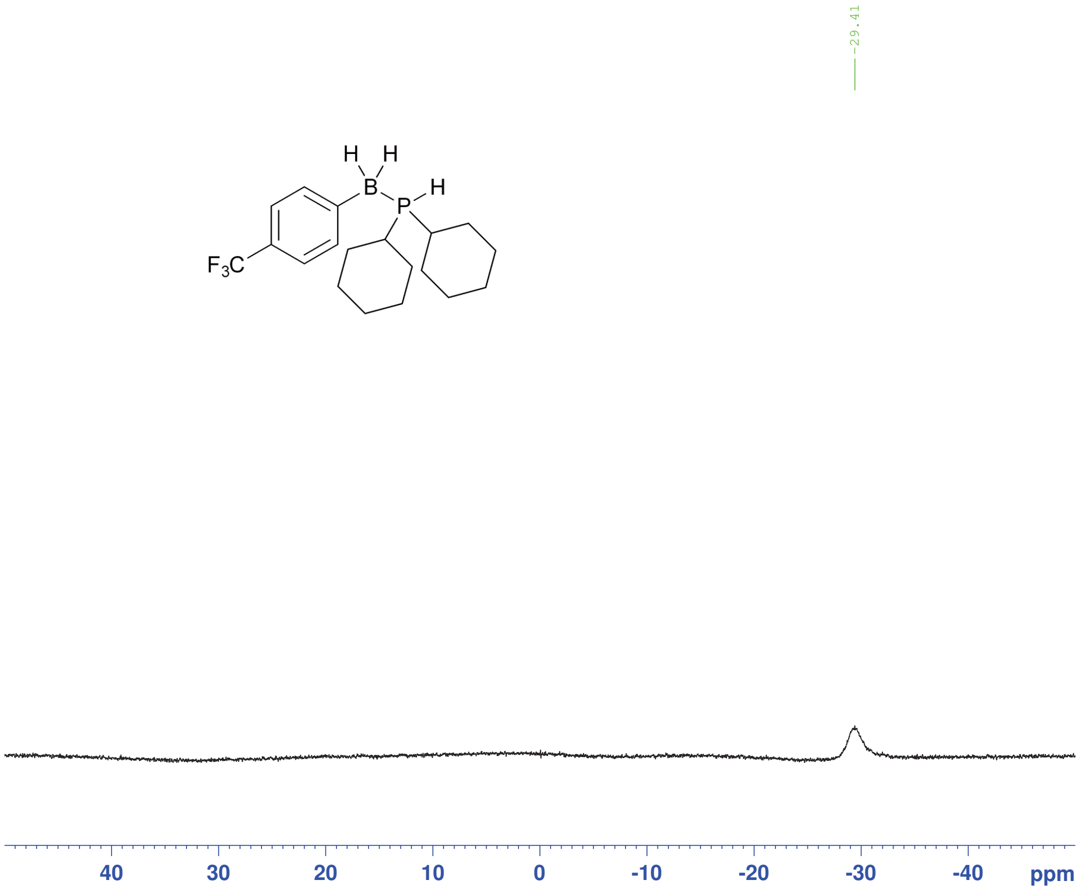

NAME	Jul22-2023
EXPNO	5
PROCNO	1
Date_	20230722
Time	16.33
INSTRUM	av400
PROBHD	5 mm PABBO BB-
PULPROG	zgig
TD	32768
SOLVENT	DMSO
NS	128
DS	4
SWH	20000.000 Hz
FIDRES	0.610352 Hz
AQ	0.8192500 sec
RG	203
DW	25.000 usec
DE	20.00 usec
TE	297.5 K
D1	1.00000000 sec
D11	0.03000000 sec
TD0	1

======== CHANNEL f1 ========

NUC1	11B
P1	6.30 usec
PL1	-2.00 dB
SFO1	128.3776050 MHz

======== CHANNEL f2 ========

CPDPRG2	waltz16
NUC2	1H
PCPD2	80.00 usec
PL2	-1.70 dB
PL12	13.80 dB
PL2W	14.48987389 W
PL12W	0.40838012 W
SFO2	400.1316005 MHz
SI	32768
SF	128.3776183 MHz
WDW	EM
SSB	0
LB	2.00 Hz
GB	0
PC	1.40

4-CF3PhBH2PH(cyclohexyl)2

IBB-nmr Analysis

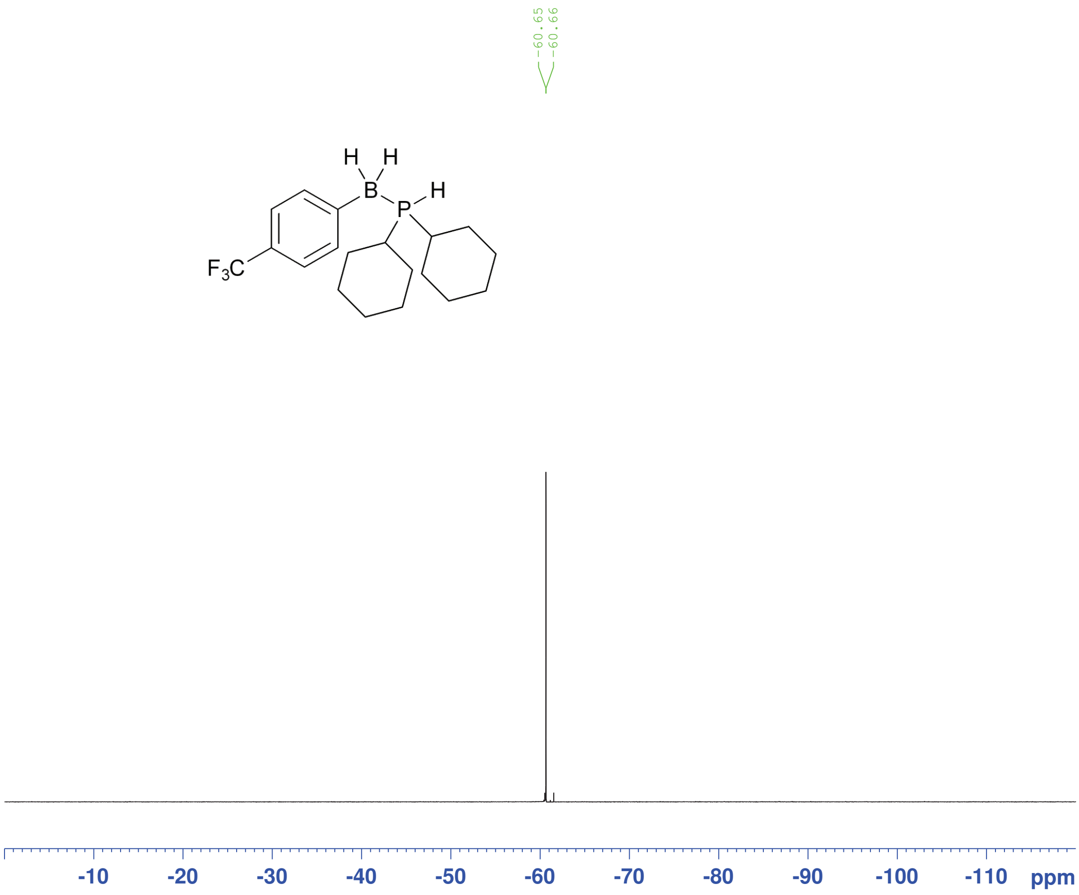


```
NAME            Aug7-2023
EXPNO                   2
PROCNO                  1
Date_            20230807
Time                 7.52
INSTRUM             av400
PROBHD   5 mm PABBO BB-
PULPROG          zgfhigqn
TD                 131072
SOLVENT              DMSO
NS                      8
DS                      4
SWH             89285.711 Hz
FIDRES           0.681196 Hz
AQ              0.7340532 sec
RG                    203
DW                  5.600 usec
DE                   6.50 usec
TE                  297.0 K
D1             1.00000000 sec
D11            0.03000000 sec
D12            0.00002000 sec
TD0                     1

======== CHANNEL f1 ========
NUC1                  19F
P1                  19.50 usec
PL1                 -4.00 dB
PL1W          16.40489006 W
SFO1          376.4607164 MHz

======== CHANNEL f2 ========
CPDPRG2          waltz16
NUC2                  1H
PCPD2             112.00 usec
PL2                -4.00 dB
PL12               18.00 dB
PL2W         24.60733604 W
PL12W         0.15526180 W
SFO2         400.1316005 MHz
SI                 65536
SF           376.4984617 MHz
WDW                   EM
SSB                    0
LB                  0.30 Hz
GB                     0
PC                  1.00
```

4CF3PhBH2PH(cyclohexyl)2

IBB-nmr Analysis

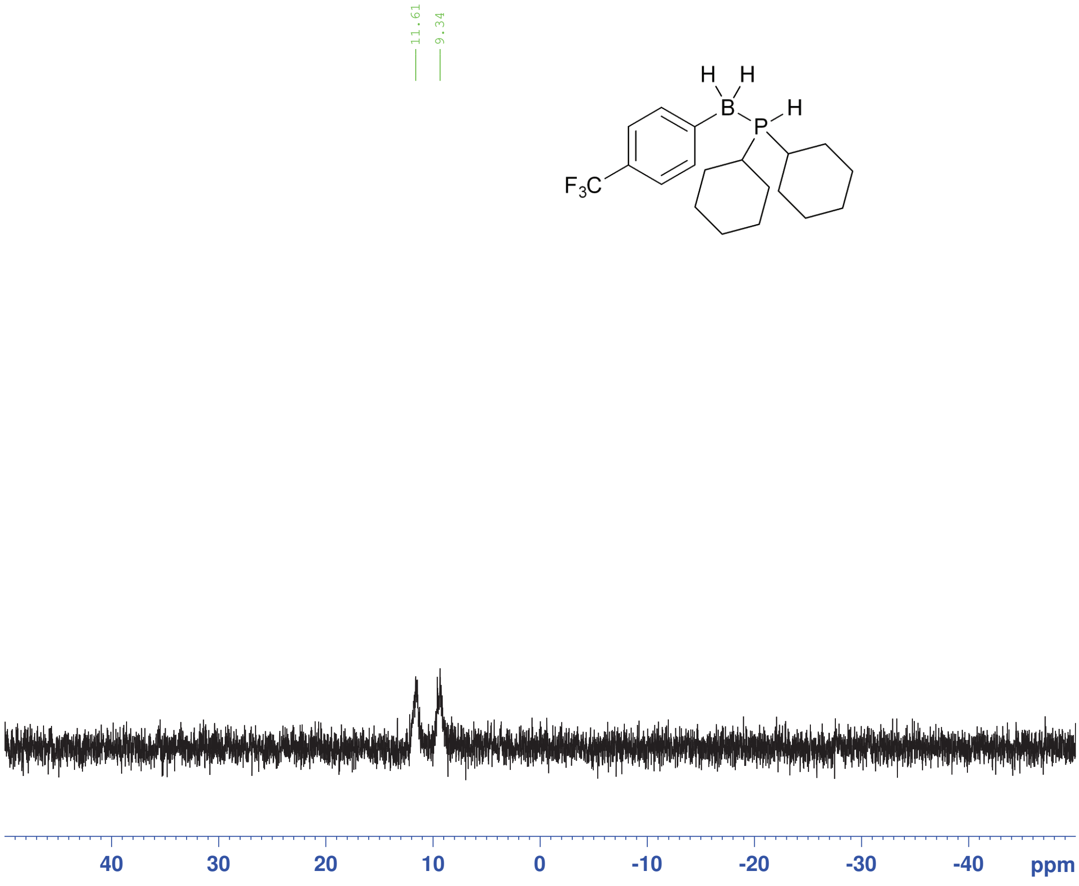


```
NAME            Jul22-2023
EXPNO                   4
PROCNO                  1
Date_            20230722
Time                16.26
INSTRUM             av400
PROBHD   5 mm PABBO BB-
PULPROG              zg30
TD                  65536
SOLVENT              DMSO
NS                     64
DS                      4
SWH             64102.563 Hz
FIDRES           0.978127 Hz
AQ              0.5112308 sec
RG                    203
DW                  7.800 usec
DE                   6.50 usec
TE                  296.9 K
D1             2.00000000 sec
TD0                     1

======== CHANNEL f1 ========
NUC1                  31P
P1                  10.00 usec
PL1                 -0.70 dB
PL1W          30.10233498 W
SFO1          161.9674942 MHz
SI                 32768
SF            161.9754962 MHz
WDW                   EM
SSB                    0
LB                  1.00 Hz
GB                     0
PC                  1.40
```

4CF3PhBH2P(NMe2)3

B-4-(Trifluoromethyl)phenyl tris(dimethylamino)phosphine–borane (39)

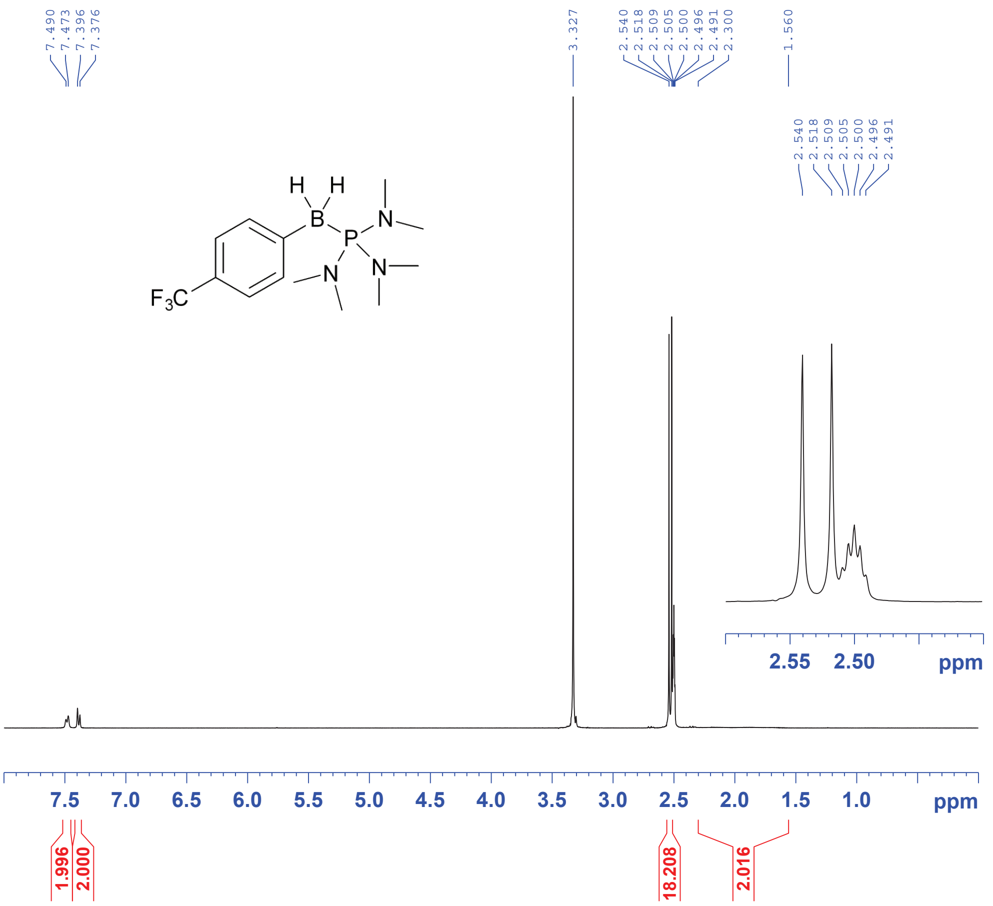

IBB-nmr Analysis

NAME	July17-2022
EXPNO	1
PROCNO	1
Date_	20220717
Time	17.57
INSTRUM	av400
PROBHD	5 mm PABBO BB-
PULPROG	zg30
TD	65536
SOLVENT	DMSO
NS	16
DS	2
SWH	8223.685 Hz
FIDRES	0.125483 Hz
AQ	3.9846387 sec
RG	203
DW	60.800 usec
DE	6.50 usec
TE	297.0 K
D1	1.00000000 sec
TD0	1

======== CHANNEL f1 ========

NUC1	1H
P1	14.00 usec
PL1	-1.80 dB
PL1W	14.82738590 W
SFO1	400.1324710 MHz
SI	32768
SF	400.1300036 MHz
WDW	EM
SSB	0
LB	0.30 Hz
GB	0
PC	1.00

4CF3PhBH2P(NMe2)3

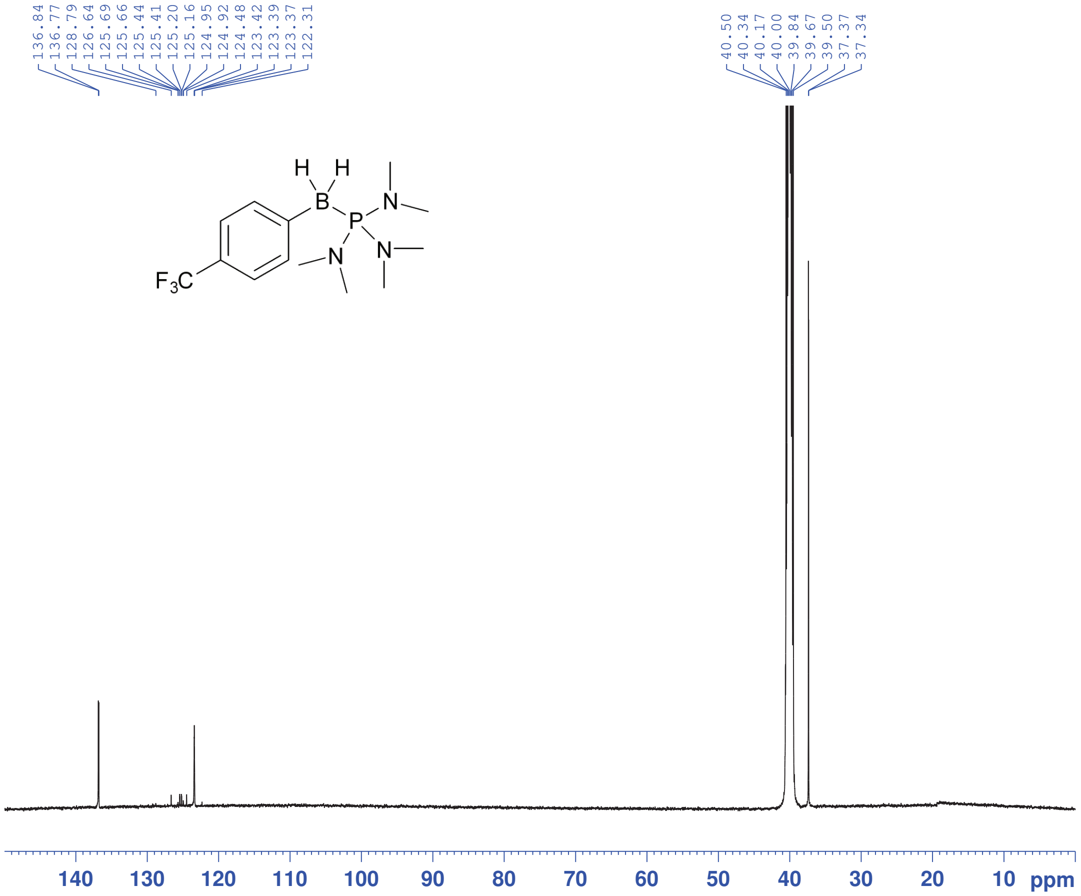

IBB-nmr Analysis

NAME	July18-2022
EXPNO	7
PROCNO	1
Date_	20220718
Time	15.23
INSTRUM	av500
PROBHD	5 mm CPDCH 13C
PULPROG	zgpg30
TD	65536
SOLVENT	DMSO
NS	4096
DS	4
SWH	30030.029 Hz
FIDRES	0.458222 Hz
AQ	1.0912410 sec
RG	90.5
DW	16.650 usec
DE	20.00 usec
TE	298.0 K
D1	2.00000000 sec
D11	0.03000000 sec
TD0	1

======== CHANNEL f1 ========

NUC1	13C
P1	10.00 usec
PL1	-5.00 dB
PL1W	14.29790783 W
SFO1	125.7703643 MHz

======== CHANNEL f2 ========

CPDPRG2	waltz16
NUC2	1H
PCPD2	80.00 usec
PL2	-4.00 dB
PL12	14.06 dB
PL13	17.00 dB
PL2W	6.30957365 W
PL12W	0.09862794 W
PL13W	0.05011872 W
SFO2	500.1320005 MHz
SI	32768
SF	125.7577857 MHz
WDW	EM
SSB	0
LB	1.00 Hz
GB	0
PC	0.30

4CF3PhBH2P(NMe2)3

IBB-nmr Analysis

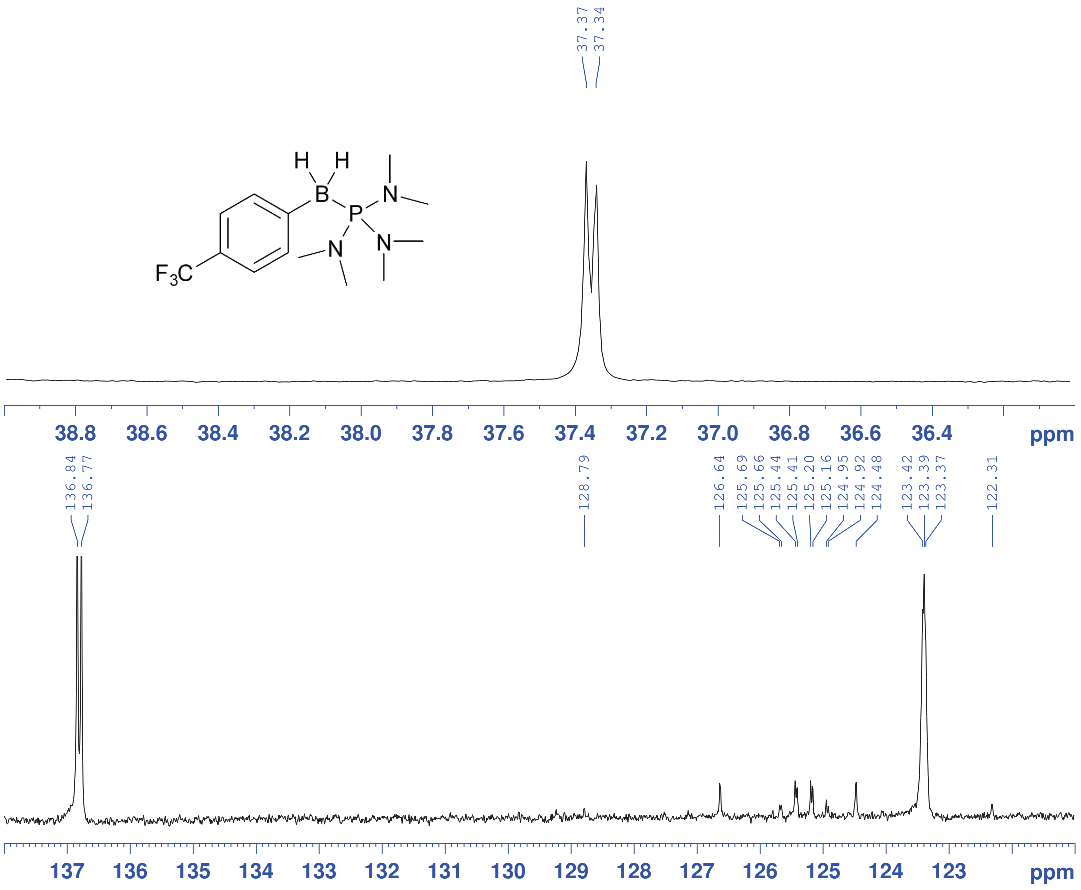


```
NAME          July18-2022
EXPNO                   7
PROCNO                  1
Date_            20220718
Time                15.23
INSTRUM             av500
PROBHD    5 mm CPDCH 13C
PULPROG           zgpg30
TD                  65536
SOLVENT              DMSO
NS                   4096
DS                      4
SWH             30030.029 Hz
FIDRES           0.458222 Hz
AQ              1.0912410 sec
RG                   90.5
DW                 16.650 usec
DE                  20.00 usec
TE                  298.0 K
D1             2.00000000 sec
D11            0.03000000 sec
TD0                     1

======== CHANNEL f1 ========
NUC1                  13C
P1                  10.00 usec
PL1                 -5.00 dB
PL1W          14.29790783 W
SFO1          125.7703643 MHz

======== CHANNEL f2 ========
CPDPRG2           waltz16
NUC2                   1H
PCPD2               80.00 usec
PL2                 -4.00 dB
PL12                14.06 dB
PL13                17.00 dB
PL2W           6.30957365 W
PL12W          0.09862794 W
PL13W          0.05011872 W
SFO2          500.1320005 MHz
SI                  32768
SF            125.7577857 MHz
WDW                    EM
SSB                     0
LB                   1.00 Hz
GB                      0
PC                   0.30
```

4CF3PhBH2P(NMe2)3

IBB-nmr Analysis

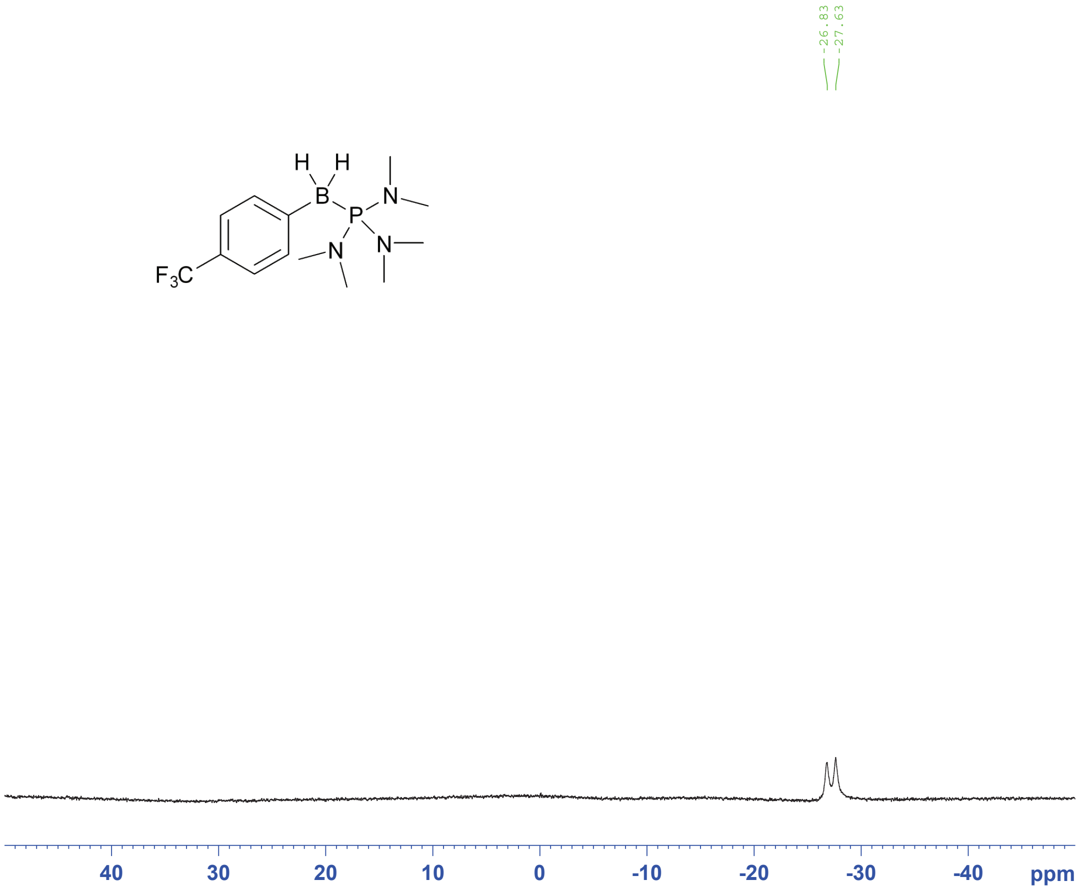


```
NAME          July17-2022
EXPNO                   3
PROCNO                  1
Date_            20220717
Time                18.08
INSTRUM             av400
PROBHD    5 mm PABBO BB-
PULPROG              zgig
TD                  32768
SOLVENT              DMSO
NS                    128
DS                      4
SWH             20000.000 Hz
FIDRES           0.610352 Hz
AQ              0.8192500 sec
RG                    203
DW                 25.000 usec
DE                  20.00 usec
TE                  297.6 K
D1             1.00000000 sec
D11            0.03000000 sec
TD0                     1

======== CHANNEL f1 ========
NUC1                  11B
P1                   6.30 usec
PL1                 -2.00 dB
SFO1          128.3776050 MHz

======== CHANNEL f2 ========
CPDPRG2           waltz16
NUC2                   1H
PCPD2               80.00 usec
PL2                 -1.70 dB
PL12                13.80 dB
PL2W          14.48987389 W
PL12W          0.40838012 W
SFO2          400.1316005 MHz
SI                  32768
SF            128.3776183 MHz
WDW                    EM
SSB                     0
LB                   2.00 Hz
GB                      0
PC                   1.40
```

4CF3PhBH2P(NMe2)3

IBB-nmr Analysis

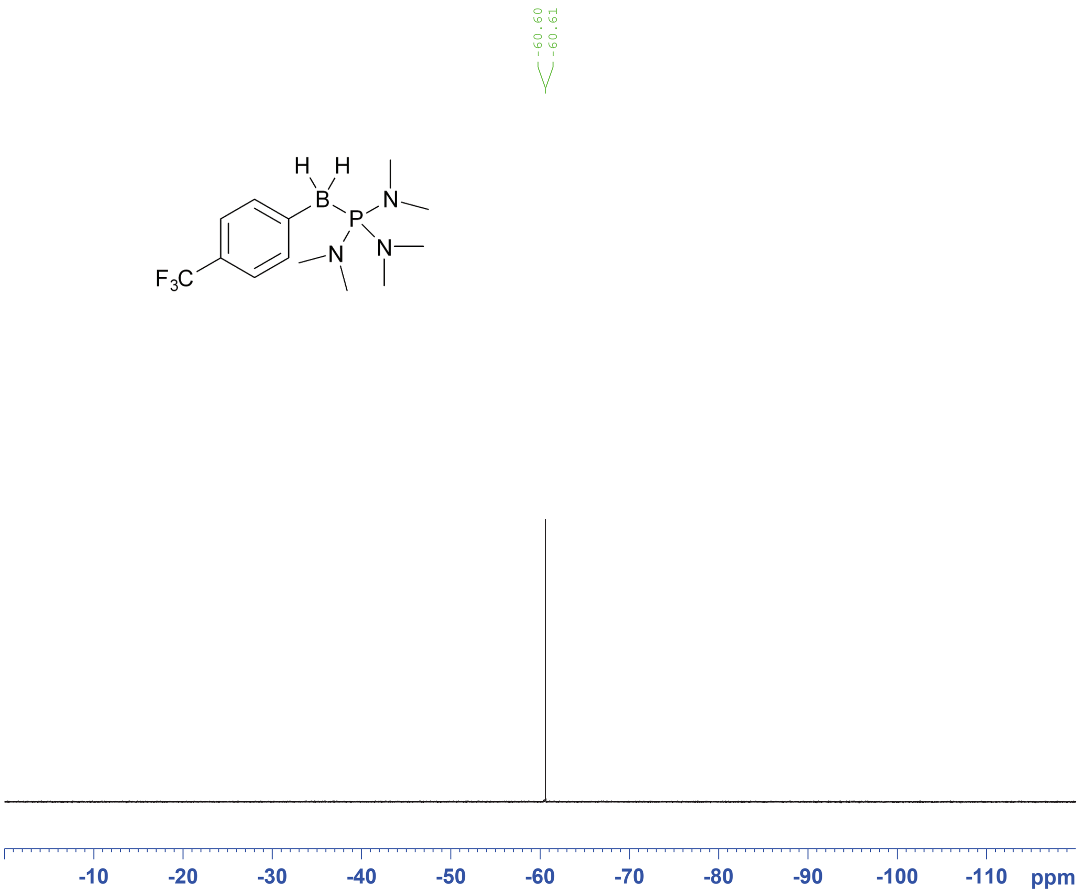

NAME	July17-2022
EXPNO	2
PROCNO	1
Date_	20220717
Time	18.01
INSTRUM	av400
PROBHD	5 mm PABBO BB-
PULPROG	zgfhigqn
TD	131072
SOLVENT	DMSO
NS	8
DS	4
SWH	89285.711 Hz
FIDRES	0.681196 Hz
AQ	0.7340532 sec
RG	203
DW	5.600 usec
DE	6.50 usec
TE	297.1 K
D1	1.00000000 sec
D11	0.03000000 sec
D12	0.00002000 sec
TD0	1
======== CHANNEL f1 ========	
NUC1	19F
P1	19.50 usec
PL1	-4.00 dB
PL1W	16.40489006 W
SFO1	376.4607164 MHz
======== CHANNEL f2 ========	
CPDPRG2	waltz16
NUC2	1H
PCPD2	112.00 usec
PL2	-4.00 dB
PL12	18.00 dB
PL2W	24.60733604 W
PL12W	0.15526180 W
SFO2	400.1316005 MHz
SI	65536
SF	376.4984617 MHz
WDW	EM
SSB	0
LB	0.30 Hz
GB	0
PC	1.00

4CF3PhBH2P(NMe2)3

IBB-nmr Analysis

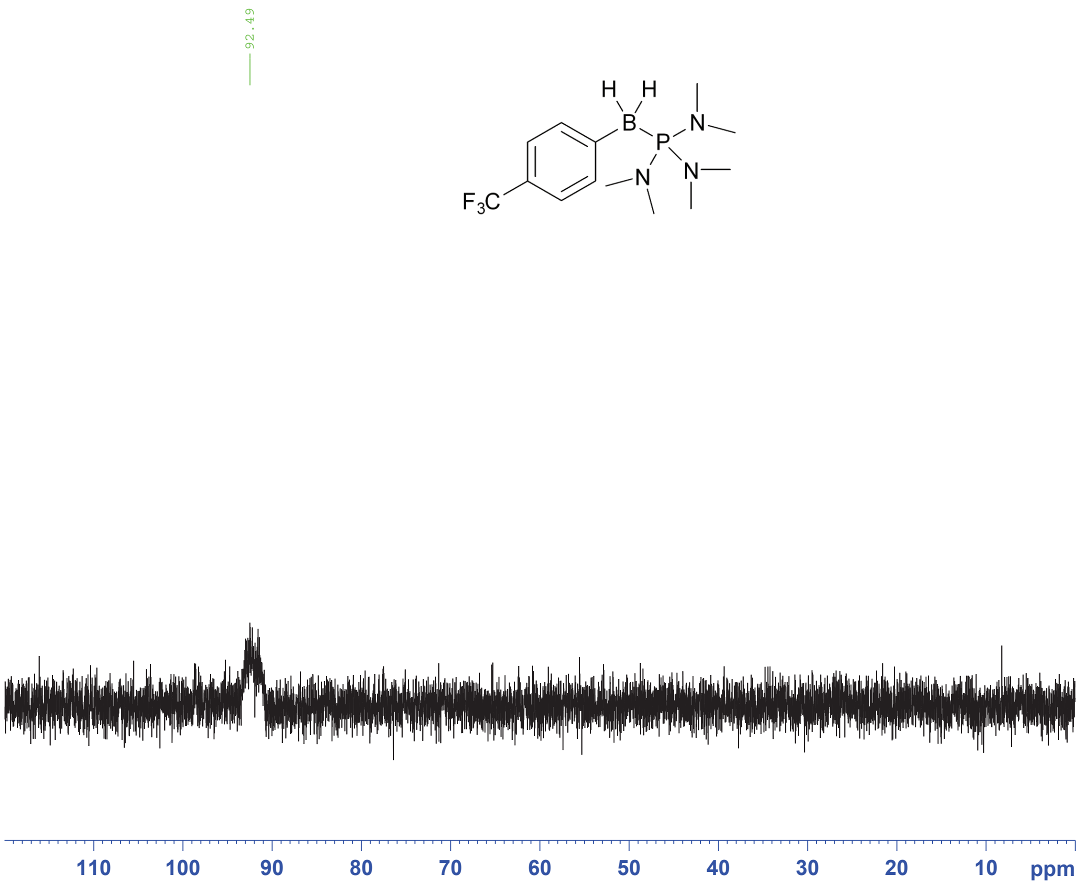

NAME	July17-2022
EXPNO	5
PROCNO	1
Date_	20220717
Time	18.18
INSTRUM	av400
PROBHD	5 mm PABBO BB-
PULPROG	zg30
TD	65536
SOLVENT	DMSO
NS	128
DS	4
SWH	64102.563 Hz
FIDRES	0.978127 Hz
AQ	0.5112308 sec
RG	203
DW	7.800 usec
DE	6.50 usec
TE	297.1 K
D1	2.00000000 sec
TD0	1
======== CHANNEL f1 ========	
NUC1	31P
P1	10.00 usec
PL1	-0.70 dB
PL1W	30.10233498 W
SFO1	161.9674942 MHz
SI	32768
SF	161.9754962 MHz
WDW	EM
SSB	0
LB	1.00 Hz
GB	0
PC	1.40
